# Life Satisfaction is More Strongly Correlated with Flourishing Than Cantril’s Ladder

**DOI:** 10.1007/s11482-026-10623-5

**Published:** 2026-06-22

**Authors:** R Noah Padgett, Chris Felton, Brendan Case, Tim Lomas, Byron Johnson, Tyler VanderWeele

**Affiliations:** 1 Department of Epidemiology, Harvard T.H. Chan School of Public Health, Boston, USA; 2 Human Flourishing Program, Harvard University, Cambridge, USA; 3 Institute for Studies of Religion, Baylor University, Waco, USA

**Keywords:** Evaluative well-being, Life satisfaction, Cantril's ladder, Well-being, Measurement

## Abstract

Policymakers and scholars alike seek an informative single-item measure of subjective well-being. While single-item measures are inherently limited, a single-item indicator can be time-sensitive, reliable, and valid if well-constructed for a particular purpose. Such as providing a population level summary of evaluative aspects of subjective well-being. We compare two measures of evaluative well-being: life satisfaction (*Overall*,* how satisfied are you with life as a whole these days? )* and Cantril’s ladder life evaluation using data in the Global Flourishing Study which includes over 200,000 participants in 22 countries. We find that life satisfaction is more strongly correlated with happiness, health, meaning, character, relationships, and financial security than life evaluation is. This result held in all 22 countries and with all dimensions of flourishing examined, including financial and material dimensions. While life evaluation is strongly related to financial aspects of well-being, life satisfaction provides a more comprehensive single-item assessment of overall subjective well-being.

**Supplementary Information:**

The online version contains supplementary material available at https://doi.org/10.1007/s11482-026-10623-5.

Subjective well-being has become an area of increasing interest in policy and academic study; it is important in its own right and also shapes subsequent health (Hülür et al., 2017; Kim et al., 2021; Helliwell, 2021). Large-scale social surveys, such as the Gallup World Poll and the European Values Survey, are regularly used to evaluate the distribution of and trends in subjective well-being. These surveys make a tradeoff between the breadth of the populations surveyed (number of countries and sample size within country) and the construct coverage (number of indicators used to capture well-being).

While in some contexts it may be desirable to carry out multidimensional assessments of subjective well-being (VanderWeele and Johnson 2025a), in a number of contexts—perhaps especially in policy settings—single-item assessments may be preferred due to the relatively concrete interpretation of scores, as argued by Helliwell and colleagues (2026). An open question in the field concerns which single-item assessment might be preferable as a population summary of subjective well-being (Lee et al., 2021; Layard and De Neve, 2023VanderWeele and Johnson 2025b).

One commonly employed assessment is Cantril’s ladder (Cantril, 1965) which is sometimes referred to as a measure of life evaluation. Hereafter, we use the term *life evaluation* (LE) to refer to the Cantril’s ladder assessment of life evaluation specifically. The wording of this question is typically given as follows:

Please imagine a ladder with steps numbered from zero at the bottom to ten at the top. Suppose we say that the top of the ladder represents the best possible life for you, and the bottom of the ladder represents the worst possible life for you. On which step of the ladder would you say you personally feel you stand at this time?

Cantril’s ladder LE has been used by the Gallup World Poll and the World Happiness Report, which since 2012 has ranked nations on this item (Helliwell et al., 2025). The indicator is conceptually appealing, widely used, and thought to be cross-culturally interpretable due to the “ladder” imagery. The imagery is hypothesized to help avoid many potential limitations of other subjective well-being measures because the item is anchored to a ladder and, thus, easier to translate. Cantril’s ladder LE has a strong country-level correlation with GDP per capita (Helliwell et al., 2025, see also Helliwell, 2008: 12).

Another commonly employed assessment approach is sometimes described as “life satisfaction.” Assessments of satisfaction with life have been used by psychologists since the 1960s (Andrews & Withey, 1976; Campbell, Converse, & Rodgers 1976), but the construct has achieved particular prominence through the five-item measure proposed by Diener (1984), which asks respondents to make overall judgments about their satisfaction with life. A single-item life satisfaction assessment has now also been used extensively. The item is often phrased as:

Overall, how satisfied are you with life as a whole these days?

Response options range from 0 (= “Not at all satisfied with your life”) to 10 (= “Completely satisfied with your life”). This single-item assessment has been used in numerous assessment contexts, including the UK’s Annual Survey, the OECD, and in the 2007 and 2008 iterations of the Gallup World Poll (OECD, 2013; Alan and Hand, 2017). The single-item assessment has shown strong correlations with multi-item assessments of life satisfaction (Cheung & Lucas, 2014) and has adequate test-retest reliability (Schimmack & Oishi, 2005). Hereafter, we use *life satisfaction* (LS) to specifically refer to the single-item measure.

LE and LS arguably constitute the two leading single-item assessments providing global evaluations about subjective well-being. There has been some prior discussion as to the relative merits of each. On the one hand, the LS question is shorter than the LE question, which may make the LS question easier for respondents to digest and takes less times. On the other hand, some have argued that the LE provides a conceptually broader framing than LS, soliciting a more cognitive – and so more properly global – evaluation of one’s life, rather than LS’s relatively tighter anchoring to (especially negative) affective well-being (Andrews & Withey, 1976: 91; Helliwell, 2021: 292 − 93Lee et al. 2021; VanderWeele and Johnson 2025a).

However, previous comparative research has found that LE may be more strongly associated with income than LS (Helliwell, 2008; Helliwell et al., 2021; Galbraith et al., 2024; Bjørnskov, 2010). Helliwell and colleagues (2010) speculated that this might in part be “due to the ladder framing of the Cantril Ladder question […] The vertical nature of the Ladder, with the best possible life being that on the top rung, might encourage respondents to think in more materialistic terms, and in a more relative way,” i.e., more in terms of social comparison (Helliwell et al., 2025: 292− 93). Additional confirmation of this hypothesis has been provided by recent qualitative work, which indicates that respondents to LE principally reflect on money and social status in framing their responses rather than on other aspects of well-being, such as relationships (Nilsson et al., 2024). In view of these differences, Helliwell et al. went so far as to propose that “an average of the individual- level answers to the life satisfaction and Ladder questions provides a measure that is more robust and informative than either on its own (Helliwell et al., 2021: 294).

In this study, we undertake a comparison of LE and LS which aims, not principally to explore their correlations with one another or with objective factors such as individual or national income, but rather their relative adequacy as single-item *global* measures of well-being. Such adequacy is not to be taken for granted in any single-item measure: fifty years ago, Campbell, Converse, & Rodgers noted that “we do not know at this stage how adept people are in stepping back from the specifics of their everyday experiences to make an overall evaluation of their lives” (1976: 61), and subsequent work by Schwartz & Strack (1991, 1999, see also Argyle, 2002) documented observational and experimental evidence for a range of cognitive biases and heuristics which can distort responses to items such as LS or LE; it would be naïve to assume that responses to either invariably reflect respondents’ considered judgments about their lives as a whole.

Previous scholarship has explored this question to a limited extent. For instance, Campbell, Converse, & Rodgers (1976) reported that LS was more strongly correlated than a question about one’s general “happiness” with a more extensive index of well-being items (1976: 43). However, they did not compare LS directly to LE on this score, and they employed data only from the United States. In this paper, we compare the correlations of LS and LE with the 12-item Secure Flourishing Index (SFI, VanderWeele, 2017), covering six domains of subjective well-being: happiness, health, meaning in life, character, social connectedness, and financial stability.^1^ We use the data from Wave 1 of the Global Flourishing Study, a large cross-national study of well-being with over 200,000 participants across 22 countries and territories, with nationally representative sampling (VanderWeele et al., 2025), to evaluate which single-item measure—LE or LS—is more strongly correlated with these other aspects of flourishing across the countries sampled. Specifically, we pre-registered the research question: are responses to LE or LS more strongly correlated with other indicators of flourishing? Based on the work of Nilsson et al. (2024), we hypothesized that LE, as measured using the Cantril Ladder, would be more strongly correlated with the financial indicators, whereas LS would be more strongly correlated with other indicators.

## Methods

### Data

Wave 1 of the Global Flourishing Study data (Johnson et al. 2024; VanderWeele et al., 2025) included 202,898 participants in the following countries and territories: Argentina, Australia, Brazil, Egypt, Germany, Hong Kong (Special Administrative Region of China), India, Indonesia, Israel, Japan, Kenya, Mexico, Nigeria, the Philippines, Poland, South Africa, Spain, Sweden, Tanzania, Turkey, the United Kingdom, and the United States. The countries were selected to (a) maximize coverage of the world’s population, (b) ensure geographic, cultural, and religious diversity, and (c) prioritize feasibility and existing data collection infrastructure. Data collection was carried out by Gallup Inc. The study protocol was approved by IRB review of Gallup and of Baylor University. Gallup secures in-country IRBs in countries where local authorities request one. Data for Wave 1 were collected principally during 2023, with some countries beginning data collection in late 2022 and exact dates varying by country. The precise sampling design varied by country to ensure nationally representative samples, and further details are available in Ritter et al. (2024) and Padgett et al. (2025). Self-report responses to the survey items included aspects of well-being such as happiness, health, meaning, character, relationships, and financial stability (Lomas et al. 2025a), along with other demographic, social, economic, political, religious, personality, childhood, community, health, and well-being variables. The data are available through the Center for Open Science (COS, https://doi.org/10.17605/osf.io/3jtz8; see also https://www.cos.io/gfs). The present analyses were pre-registered (https://doi.org/10.17605/osf.io/83nsh).

## Variables

For this study, we utilized LE and LS as assessed in the GFS using the wording above. We also used the other 11 items, beyond LS, from the flourishing index of VanderWeele (2017) covering happiness, self-rated physical and mental health, meaning, character, close social relationships, and financial and material stability. Each of these items was also self-rated 0–10. The word for these other items is as follows: happiness, “In general, how happy or unhappy do you usually feel?”; health, “In general, how would you rate your physical health?” and “How would you rate your overall mental health?”; meaning, “I understand my purpose in life” and “Overall, to what extent do you feel the things you do in your life are worthwhile?”; character, “I always act to promote good in all circumstances, even in difficult and challenging situations” and “I am always able to give up some happiness now for greater happiness later”; relationships, “I am content with my friendships and relationships” and “My relationships are as satisfying as I would want them to be”; and financial stability, “How often do you worry about being able to meet normal monthly living expenses?” and “How often do you worry about safety, food, or housing?” These items were chosen because they were available in the GFS questionnaire.

It is important to note that, in the GFS questionnaire, not only LE but also the entire SFI, including LS, was prefaced with the ladder framing developed by Cantril. Participants were read the following – or a closely similar statement – at four different points in the course of responding to the 12-item SFI: “For these next questions, think about the ladder once again with the top of the ladder at ten being the best possible state or arrangement that you would want and the bottom of the ladder at zero being the worst possible state or arrangement. You can use any number between 0 and 10” (Lomas et al. 2025a: Supplementary File 3). This means that any observed differences between LS and LE in the GFS data cannot be attributed to the ladder framing itself; indeed, if the ladder framing independently anchors respondents to income and status, then the GFS might reasonably be taken as setting a floor for the potential divergence in responses to those items both within individuals and across countries.

## Analytic Strategy

In each country, we computed the correlation between LE, LS, and the other 11 flourishing indicators to give a 13 × 13 correlation matrix. Within each country, we calculated the average correlation between LE and all other indicators and between LS and all other indicators. We evaluated in each of the 22 countries whether the average correlation with LS or LE was greater. Analyses were conducted by country to ensure the complex sampling design was appropriately accounted for in each country. Our hypothesis was tested using a Bernoulli test for the equality of the proportion of countries where the average correlation is greater for LE than LS. We also examined whether LS or LE had a higher average correlation across countries in each domain or for each indicator. Missing data were negligible for Wave 1 for all variables included in this study, and analyses were conducted based on the complete-case data, utilizing 197,036 of the 202,898 observations from Wave 1. See the supplemental material for more information on missing data (Table S25).

## Results

The average correlations of LS and LE with all other indicators are reported in Table 1. LS had a higher average correlation in all 22 countries included in the GFS (χ^2^(df = 1) = 20.0; *p* = 7.56e-06). The magnitude of the difference in average correlation ranged from 0.026 correlation units in Israel to 0.130 in the Philippines. The overall correlation matrix among the 13 indicators is provided in Table S1. When the correlations were averaged across countries for each of the domains of flourishing (Table 2), LS also had a higher correlation on average across countries (χ^2^(df = 1) = 65.5; *p* = 5.74e-16) for all six domains: happiness, self-rated physical and mental health, meaning, character, close social relationships, and financial and material stability. When examining the individual correlations of LS and LE with each of the 11 other flourishing indicators in each of the 22 countries, LS showed a stronger correlation in 217 of the 242 comparisons (89.6%). Of the 25 correlations where LE was stronger, 15 involved the financial and material indicators. For the nine non-financial/material indicators, LS had a stronger correlation than LE in 188 of the 198 comparisons (94.9%). When the financial and material indicators are analyzed separately from the other nine flourishing indicators, the average correlation across countries for the two financial and material indicators was stronger for LE in 8 of the 22 countries, and stronger for LS in 14 of the 22 countries (Table S2). The country-specific correlation matrices are provided in Tables S3-24. Results measuring the strength of non-linear associations are consistent with these results (Felton et al., in prep). These findings hold when analyzed separately by males or females (see Table S26).

**Table 1:** Average correlation of LS with all other flourishing indicators is greater than LE for all countries included in the GFS (*N* = 197,036)

Country	Life satisfaction	Life evaluation
Argentina	0.43 (0.04)	0.35 (0.04)
Australia	0.59 (0.04)	0.54 (0.04)
Brazil	0.48 (0.03)	0.39 (0.03)
Egypt	0.27 (0.04)	0.23 (0.04)
Germany	0.44 (0.03)	0.38 (0.03)
Hong Kong	0.72 (0.04)	0.67 (0.04)
India	0.33 (0.03)	0.24 (0.03)
Indonesia	0.39 (0.04)	0.27 (0.04)
Israel	0.45 (0.05)	0.42 (0.05)
Japan	0.67 (0.02)	0.64 (0.02)
Kenya	0.24 (0.03)	0.19 (0.03)
Mexico	0.44 (0.04)	0.35 (0.04)
Nigeria	0.30 (0.04)	0.21 (0.04)
Philippines	0.36 (0.04)	0.23 (0.04)
Poland	0.50 (0.04)	0.38 (0.04)
South Africa	0.33 (0.05)	0.28 (0.05)
Spain	0.45 (0.04)	0.42 (0.04)
Sweden	0.57 (0.03)	0.52 (0.03)
Tanzania	0.27 (0.04)	0.21 (0.04)
Turkey	0.46 (0.05)	0.39 (0.05)
United Kingdom	0.56 (0.04)	0.50 (0.04)
United States	0.60 (0.03)	0.55 (0.03)

Note. Reported in columns are average correlation (standard error of average correlation) of LE/LS with all other flourishing indicators

Omnibus Bernoulli test of proportions to test the H_0_: *p*_LE_= *p*_LS_; *p*_LE_, proportion of countries where the average correlation of LE is greater than the average correlation of LS with all other flourishing index indicators; *p*_LS_, proportion of countries where the average correlation of LS is greater than the average correlation of LE with all other flourishing index indicators

χ^2^(df = 1) = 20.0; *p* = 7.56e-06

**Table 2:** Average correlation across countries of LS and LE with flourishing domains (*N* = 197,036)

Flourishing domain	Life satisfaction	Life evaluation
Happiness*	0.71 (0.04)	0.59 (0.04)
Physical & Mental Health	0.46 (0.03)	0.38 (0.03)
Meaning & Purpose	0.52 (0.03)	0.41 (0.04)
Character & Virtue	0.30 (0.03)	0.26 (0.03)
Close social relationships	0.46 (0.03)	0.36 (0.04)
Financial & material stability	0.34 (0.03)	0.33 (0.03)

Note. Reported estimates are the average correlation across countries of LS/LE with the indicators in each flourishing domain

*LS*, life satisfaction; *LE*, life evaluation

Omnibus Bernoulli test of proportions to test the H_0_: *p*_LE_= *p*_LS_; *p*_LE_, proportion of countries where the average correlation of LE is greater than the average correlation of LS with the items within the flourishing domain; *p*_LS_, proportion of countries where the average correlation of LS is greater than the average correlation of LE with the items within the flourishing domain

χ^2^(df = 1) = 65.5; *p* = 5.74e-16

*The happiness domain comprises only a single item since LS is removed from it

## Discussion

LS and LE, while related, may differ in terms of their conceptual breadth and what individuals think of while responding, thus influencing their efficacy as standalone indicators of well-being (Kahneman & Deaton, 2010; Benjamin et al., 2023) and may also differ in their relations with other aspects of well-being (Holt-Lunstad et al., 2017; Chen et al., 2019; VanderWeele and Johnson 2025b). The current results provide an important contribution to this literature by providing multinational empirical evidence that self-rated life satisfaction is more strongly related to a wide range of indicators of subjective well-being or flourishing than is the Cantril ladder measure of life evaluation, which is the only global measure of well-being present in the Gallup World Poll.

As noted above, recent analyses have indicated that responses to the LE question may be particularly influenced by thoughts of attainment and accomplishment—perhaps elicited by the ladder imagery—especially in relation to money and status rather than aspects of well-being such as relational ties (Nilsson et al., 2024, see also Helliwell et al., 2021: 292 − 93). Our analyses corroborate these results, with LS consistently showing stronger associations with non-material indicators across countries. However, contrary to expectations, LS more often (in 14 of the 22 countries) had stronger correlations than LE with the financial/material indicators as well.

Taken as a whole, our results provide compelling evidence for preferring LS over LE as a single-item assessment of subjective well-being because LS correlated more strongly, on average, with all aspects of flourishing than LE did. Patterns were consistent across countries and the domains of flourishing. These data and analyses are perhaps not absolutely definitive, insofar as our sample only includes 22 countries, albeit with considerable geographical and cultural diversity. Moreover, these countries only comprise lower-middle-, upper-middle- and upper-income- countries (Padgett et al., 2025) with no low-income countries (although Tanzania, with a per capita Gross National Income of $1200/year, only narrowly exceeds the World Bank’s low-income threshold of $1145/year) (World Bank, 2024). It is also possible that some idiosyncrasy of the GFS survey or data collection process was partially responsible for these results. And of course, it is possible that some other question might constitute a yet better single-item assessment of subjective well-being than either LS or LE (see also the average correlations for other indicators reported in Table S1 of the Online Supplement). Nevertheless, the consistency of the results in favor of LS over LE, across the 22 countries, and across the domains of flourishing, is striking.

The implications of this work for the study and promotion of well-being and for policy are potentially far-reaching. If the results here do generalize to other countries, and to other survey and assessment modalities, this would provide a strong argument for replacing Cantril’s Ladder LE with LS as the primary self-report single-item assessment of well-being. Doing so would likely considerably alter the World Happiness Report rankings, which are based on LE data from the Gallup World Poll, which have consistently been dominated by the Nordic nations (with Finland top in 2026 for the ninth consecutive year) together with other affluent Western countries (Lomas et al., 2025b; Helliwell et al., 2025). A shift to LS might shift some of the attention of academics, pundits, and policymakers away from Scandinavian countries such as Sweden, which scores high on LE but somewhat lower on LS and composite flourishing (Helliwell et al., 2025; Lomas et al., 2025b; VanderWeele et al., 2025) and toward middle-income countries such as Indonesia or the Philippines, which have high scores both for LS and for composite flourishing (VanderWeele et al., 2025; Lomas et al., 2026) to discern clues about what promotes well-being in society, and indeed what a thriving society itself looks like.

A move from LE to LS could also alter our understanding of the current relations between economic development and well-being. LE is strongly albeit imperfectly associated with GDP per capita (Helliwell et al., 2025). However, other aspects of flourishing show weaker positive or even negative correlations with GDP/capita; aspects of meaning, relationship satisfaction and self-reported pro-social character traits seem to be higher in middle-income countries than high-income countries (VanderWeele et al., 2025; Kim et al., 2025; Oishi & Diener, 2014). If LE responses are indeed shaped preponderantly by thoughts of money and status (Nilsson et al., 2024), a focus on LE (instead of LS or composite flourishing) might inadvertently cause policymakers to neglect other central aspects of well-being. Insofar as we make economic growth or development the central policy priority, as has arguably already happened in many countries around the world, we might inadvertently sacrifice some of the “humanistic” aspects of well-being, such as meaning, character, or relationships. LE is arguably insufficient to provide a sufficiently meaningful corrective to this somewhat myopic focus on economic growth, given its strong correlations with GDP per capita, and the ladder possibly serving as a subjective gauge of such economic prosperity.

We arguably need further work on finding ways to carry out economic development in a manner that does not compromise meaning, pro-social character, or relationships (VanderWeele et al. 2025; VanderWeele and Johnson 2025b). Policymakers would ideally be guided by the goals of both promoting economic growth and improving non-material well-being (possibly measured by LS if a single-item assessment is needed). Seeking only to maximize economic growth alone, or even economic growth in conjunction with LE, may not be a fruitful path forward towards a flourishing society.

As such, while not definitive, our results suggest LS (ideally without any ladder framing) should be preferred over Cantril’s Ladder LE. The use of LE (over LS) in the World Happiness Report is effectively a consequence of this being the primary well-being measure in the Gallup World Poll. These results suggest it might be time to re-evaluate this preference for LE over LS, with a call to co-assess LE and LS to further study the potential consequences of how we select single-item well-being assessments. Our understanding of, and progress toward, well-being may depend upon it.

## Supplementary Information

Below is the link to the electronic supplementary material.

Supplementary Material 1 (PDF 30.1 MB)

Supplementary Material 2 (DOCX 161 KB)

## Data Availability

The non-sensitive data collected by Gallup for Wave 1 can be accessed through the Open Science Framework repository https://doi.org/10.17605/osf.io/c8hbk.

## Footnotes

1 The SFI includes LS; the correlations of LS and LE are with the index’s remaining 11 items.
