# Supplementary material for: Life Satisfaction is More Strongly Correlated with Flourishing Than Cantril’s Ladder
Source: Appl Res Qual Life. 2026 Jun 22;21(5):3337–48. doi: 10.1007/s11482-026-10623-5 (PMC13624039; doi:10.1007/s11482-026-10623-5)
Supplement: Supplementary file 1 [file 11482_2026_10623_MOESM1_ESM.pdf]

Online Supplement for “Life satisfaction is more strongly correlated with flourishing than Cantril’s Ladder"

Section 1

Measues of Non-Linear Assocation

In the main text of the paper, we report estimated correlations, but correlation is a measure of linear association. We are more broadly interested in the general associations of *Life Satisfaction* (LS) and *Cantril's Ladder* (CL) with various flourishing measures. This supplement first reports measures of non-linear association for each country and item pair. We then generate weighted scatterplots for each country and item pair along with superimposed non-linear curves. The results are consistent with the main results reported in the paper: LS is more strongly associated with flourishing than is CL.

1.1 Methodological Details

We are primarily interested in the associations of LS and CL with different flourishing items and less interested in associations between different flourishing items themselves. We therefore focus on associations of (i) LS and CL with (ii) the remaining 11 items of the VanderWeele Flourishing Index, resulting in $11 \times 2 = 22$ association measures and scatterplots for each country.

Each scatterplot superimposes a non-linear curve estimated using the data. In particular, we run a piecewise polynomial regression using B-splines for each pair of variables.[1]We then perform the modified cross-validation procedure developed by Wieczorek, Guerin and McMahon (2022) to account for the complex design of the GFS using 5 folds.[2]We report the cross-validation estimate of the model's root mean squared error

[1]More specifically, we use the `survey` package in conjunction with the `splines` package to account for the complex survey design of the GFS (Lumley, 2004). We specify cubic splines with 4 degrees of freedom, resulting in a single knot. While thin-plate splines as implemented in the `mgcv` package often perform better than B-splines in simple random samples, we opt for B-splines to more easily incorporate information about the complex survey design (Wood and Wood, 2015).

[2]For nine countries, this cross-validation procedure separates the data into 5 folds using a stratified cluster sample. That is, the sample itself is stratified, and a random sample of clusters is taken from each stratum to form 5 non-overlapping subsets. Measures of out-of-fold predictive accuracy use survey weights. For 13 countries, certain strata were so small that this procedure could not be performed. For these countries, we used cluster sampling to form the folds, but avoided stratifying

(RMSE), with lower RMSE indicating better fit to the data. These results are extremely similar to the correlation results reported in the main text. This is unsurprising, as the scatterplots show that the associations are fairly linear.

1.2 Departure from Pre-Registered Analysis

We departed from the pre-registered analysis, which specified using "a weighted P-spline regression, with weights given by the GFS survey weights, as implemented by `geom_smooth(aes(weight = ANNUAL_WEIGHT1), formula = y~s(x,bs='ps'))` in the `ggplot2` package in `R`."[3] After registering this analysis, but before estimating the cross-validated RMSE, we discovered this approach produced non-linear curves that clearly overfit the data (see Section 25). Under the hood, `geom_smooth()` uses the `gam` function from the `mgcv` package to fit curves. `gam` uses generalized cross-validation (GCV) to select smoothing parameters (Wickham, 2016; Wood and Wood, 2015). GCV, however, can produce poor estimates of prediction error in complex surveys, and in our case it appeared to lead to undersmoothing (Wieczorek, Guerin and McMahon, 2022). In Section 25, we show the initial plots we generated that alerted us to this overfitting problem. We subsequently decided to use the `survey` package in conjunction with the `splines` package to estimate curves that account for the complex structure of the GFS survey (Lumley, 2004). That said, the scatterplots show that associations are fairly linear.

1.3 A Note on Between-Country Comparisons

While tempting, we discourage between-country comparisons of scatterplots and fitted curves for two reasons. First, the scatterplots reveal that respondents from different countries likely interpret the 0–10 scale differently. Second, the scatterplots for one country—Israel—are sufficiently unusual that they suggest possible translation issues or inattentive responding. We briefly discuss these issues in turn.

First, scatterplots for some countries (e.g., Egypt and India, among others) reveal what we call the "window" pattern: the plotted data points create what looks like a four-pane window frame, with a border along the edges of the plot and vertical and horizontal bands of points that intersect in the center. In these "window" countries, respondents have a strong tendency to clump at three values—0, 5, and 10—suggesting the respondents may interpret the 0–10 scale differently from respondents in "non-window" countries. As a result, comparing a window country's scatterplot with a non-window country's scatterplot may tell us more about differences in interpretation than differences in association.

the data. Specifically, for Argentina, Brazil, Mexico, Egypt, India, Indonesia, Israel, Kenya, Nigeria, Philippines, South Africa, Tanzania, and Turkey, we used simple cluster sampling to create the folds. For all other countries, we used stratified cluster sampling.

[3]The pre-registration plan can be found here: `https://doi.org/10.17605/OSF.IO/83NSH`.

Second, Israel's scatterplots show surprising clusters of points: many respondents who report LS and CL values of 0 also report high levels of happiness, mental health, relational contentment, meaning in life, purpose in life, or promoting the good. Visually, this results in clusters of points in the top left corner of Israel's scatterplots. This sort of clustering is to be expected in window countries, but Israel's scatterplots are quite linear with the exception of these clusters. Several other non-window countries show similar clustering, but only for a small number of items. For instance, Mexico's plots for *Purpose in Life*, *Promoting the Good*, and *Delayed Gratification* contain clusters of points in the upper left, but its plot for *Happiness* does not, and its plot for *Mental Health* contains clusters in the top and bottom left. Israel is unusual in that all 22 plots contain top-left clusters of points, including the plots for *Happiness* and *Mental Health*. Although we cannot say so definitively, it is possible that this clustering reflects inattentive responding or translation issues, and thus the fitted curves (and plotted data) should not necessarily be taken at face value.

Section 2

CV RMSE Results

This section displays the estimated cross-validated root mean squared error (CV RMSE) for each of the 22 item pairs for each country, and the following sections display the scatterplots for each country. CV RMSE estimates the expected test set error—that is, how poorly the model would predict the outcome if trained and tested on new data drawn from the same population (in expectation across repeated samples) (Bates, Hastie and Tibshirani, 2024; Wieczorek, Guerin and McMahon, 2022).[4] A lower RMSE indicates better fit and thus a stronger association. We display estimated CV RMSEs in Tables 1–22. For each LS–CL comparison in each country, we bold the smaller CV RMSE.

Aggregating the results from all 22 countries, and looking across 11 flourishing indicators, we have 242 total comparisons between LS and CL. In 208 (86%) of them, LS exhibits a stronger association than CL; in 13 (5%), LS and CL are tied (rounding to the nearest 0.01); in 21 (9%), CL is more strongly associated. We can also further restrict the analysis to non-material flourishing indicators, which we hypothesized (prior to analyzing the data) would show stronger associations with LS than CL. Of these 198 comparisons, LS is more strongly associated than CL in 184 (93%); in 8 (4%), LS and CL are tied; and in 6 (3%), CL is more strongly associated than LS.

[4]More specifically, on new samples of the same size as the existing samples.

Flourishing Item	*Life Satisfaction*	*Cantril's Ladder*
Happiness	**1.47**	1.73
Physical Health	**1.99**	2.04
Mental Health	**1.88**	2.00
Meaning in Life	**1.70**	1.94
Purpose in Life	**2.07**	2.20
Promoting the Good	**1.55**	1.59
Delayed Gratification	**2.03**	2.06
Relational Contentment	**2.15**	2.24
Relationship Satisfaction	**2.15**	2.31
Financial Worry	3.57	**3.53**
Material Worry	3.66	**3.65**

Table 1: 5-Fold Cross-Validated RMSE for Argentina. Flourishing indicators are regressed, one at a time, on either LS or CL, using a piecewise polynomial regression with B-splines. Regressions incorporate survey weights, and the cross-validation procedure uses cluster sampling and survey weights to form each fold. Lower values indicate better fit and stronger association. See Section 1.1 for more details.

Flourishing Item	*Life Satisfaction*	*Cantril's Ladder*
Happiness	**1.05**	1.23
Physical Health	1.82	**1.79**
Mental Health	**1.51**	1.72
Meaning in Life	**1.40**	1.62
Purpose in Life	**2.04**	2.17
Promoting the Good	**1.49**	1.52
Delayed Gratification	**1.84**	1.85
Relational Contentment	**1.81**	1.92
Relationship Satisfaction	**1.93**	2.09
Financial Worry	2.65	**2.63**
Material Worry	2.44	**2.43**

Table 2: 5-Fold Cross-Validated RMSE for Australia. Flourishing indicators are regressed, one at a time, on either LS or CL, using a piecewise polynomial regression with B-splines. Regressions incorporate survey weights, and the cross-validation procedure uses cluster sampling and survey weights to form each fold. Lower values indicate better fit and stronger association. See Section 1.1 for more details.

Flourishing Item	*Life Satisfaction*	*Cantril's Ladder*
Happiness	**1.54**	1.86
Physical Health	**2.04**	2.12
Mental Health	**2.14**	2.34
Meaning in Life	**1.64**	1.95
Purpose in Life	**2.14**	2.29
Promoting the Good	**1.66**	1.72
Delayed Gratification	**2.17**	2.20
Relational Contentment	**2.27**	2.42
Relationship Satisfaction	**2.42**	2.60
Financial Worry	3.66	3.66
Material Worry	3.80	**3.78**

Table 3: 5-Fold Cross-Validated RMSE for Brazil. Flourishing indicators are regressed, one at a time, on either LS or CL, using a piecewise polynomial regression with B-splines. Regressions incorporate survey weights, and the cross-validation procedure uses cluster sampling and survey weights to form each fold. Lower values indicate better fit and stronger association. See Section 1.1 for more details.

Flourishing Item	*Life Satisfaction*	*Cantril's Ladder*
Happiness	2.64	2.64
Physical Health	2.70	2.70
Mental Health	**2.02**	2.06
Meaning in Life	**2.21**	2.33
Purpose in Life	**2.33**	2.36
Promoting the Good	**2.02**	2.05
Delayed Gratification	**2.95**	2.97
Relational Contentment	**2.61**	2.64
Relationship Satisfaction	**2.52**	2.54
Financial Worry	3.29	**3.26**
Material Worry	**3.26**	3.27

Table 4: 5-Fold Cross-Validated RMSE for Egypt. Flourishing indicators are regressed, one at a time, on either LS or CL, using a piecewise polynomial regression with B-splines. Regressions incorporate survey weights, and the cross-validation procedure uses cluster sampling and survey weights to form each fold. Lower values indicate better fit and stronger association. See Section 1.1 for more details.

Flourishing Item	*Life Satisfaction*	*Cantril's Ladder*
Happiness	**1.06**	1.41
Physical Health	1.97	**1.96**
Mental Health	**1.96**	2.07
Meaning in Life	**1.45**	1.73
Purpose in Life	**2.17**	2.24
Promoting the Good	**1.79**	1.80
Delayed Gratification	**2.10**	2.11
Relational Contentment	**2.01**	2.10
Relationship Satisfaction	**2.14**	2.24
Financial Worry	2.77	**2.75**
Material Worry	2.61	2.61

Table 5: 5-Fold Cross-Validated RMSE for Germany. Flourishing indicators are regressed, one at a time, on either LS or CL, using a piecewise polynomial regression with B-splines. Regressions incorporate survey weights, and the cross-validation procedure uses cluster sampling and survey weights to form each fold. Lower values indicate better fit and stronger association. See Section 1.1 for more details.

Flourishing Item	*Life Satisfaction*	*Cantril's Ladder*
Happiness	**1.05**	1.22
Physical Health	**1.35**	1.38
Mental Health	**1.37**	1.60
Meaning in Life	**1.18**	1.38
Purpose in Life	**1.53**	1.61
Promoting the Good	**1.34**	1.38
Delayed Gratification	**1.54**	1.61
Relational Contentment	**1.44**	1.60
Relationship Satisfaction	**1.49**	1.67
Financial Worry	2.11	**2.09**
Material Worry	**1.96**	2.01

Table 6: 5-Fold Cross-Validated RMSE for Hong Kong. Flourishing indicators are regressed, one at a time, on either LS or CL, using a piecewise polynomial regression with B-splines. Regressions incorporate survey weights, and the cross-validation procedure uses cluster sampling and survey weights to form each fold. Lower values indicate better fit and stronger association. See Section 1.1 for more details.

Flourishing Item	*Life Satisfaction*	*Cantril's Ladder*
Happiness	**2.89**	3.19
Physical Health	**3.15**	3.27
Mental Health	**2.94**	3.11
Meaning in Life	**2.97**	3.23
Purpose in Life	**2.83**	2.91
Promoting the Good	**2.53**	2.61
Delayed Gratification	**3.38**	3.42
Relational Contentment	**2.96**	3.05
Relationship Satisfaction	**2.92**	3.01
Financial Worry	**4.01**	4.02
Material Worry	**4.07**	4.08

Table 7: 5-Fold Cross-Validated RMSE for India. Flourishing indicators are regressed, one at a time, on either LS or CL, using a piecewise polynomial regression with B-splines. Regressions incorporate survey weights, and the cross-validation procedure uses cluster sampling and survey weights to form each fold. Lower values indicate better fit and stronger association. See Section 1.1 for more details.

Flourishing Item	*Life Satisfaction*	*Cantril's Ladder*
Happiness	**1.77**	1.95
Physical Health	**1.73**	1.86
Mental Health	**1.67**	1.81
Meaning in Life	**1.52**	1.74
Purpose in Life	**1.54**	1.62
Promoting the Good	**1.66**	1.73
Delayed Gratification	**1.85**	1.94
Relational Contentment	**1.67**	1.75
Relationship Satisfaction	**1.70**	1.82
Financial Worry	**3.24**	3.27
Material Worry	3.35	3.35

Table 8: 5-Fold Cross-Validated RMSE for Indonesia. Flourishing indicators are regressed, one at a time, on either LS or CL, using a piecewise polynomial regression with B-splines. Regressions incorporate survey weights, and the cross-validation procedure uses cluster sampling and survey weights to form each fold. Lower values indicate better fit and stronger association. See Section 1.1 for more details.

Flourishing Item	*Life Satisfaction*	*Cantril's Ladder*
Happiness	**1.23**	1.29
Physical Health	1.72	**1.69**
Mental Health	1.56	1.56
Meaning in Life	**1.58**	1.66
Purpose in Life	**1.65**	1.66
Promoting the Good	**1.46**	1.49
Delayed Gratification	**2.22**	2.25
Relational Contentment	**1.69**	1.75
Relationship Satisfaction	**1.92**	1.96
Financial Worry	2.32	2.32
Material Worry	**2.31**	2.34

Table 9: 5-Fold Cross-Validated RMSE for Israel. Flourishing indicators are regressed, one at a time, on either LS or CL, using a piecewise polynomial regression with B-splines. Regressions incorporate survey weights, and the cross-validation procedure uses cluster sampling and survey weights to form each fold. Lower values indicate better fit and stronger association. See Section 1.1 for more details.

Flourishing Item	*Life Satisfaction*	*Cantril's Ladder*
Happiness	**1.01**	1.21
Physical Health	**1.67**	1.71
Mental Health	**1.46**	1.59
Meaning in Life	**1.41**	1.56
Purpose in Life	**1.79**	1.84
Promoting the Good	**1.58**	1.63
Delayed Gratification	**1.90**	1.91
Relational Contentment	**1.68**	1.77
Relationship Satisfaction	**1.62**	1.72
Financial Worry	**2.46**	2.48
Material Worry	**2.18**	2.20

Table 10: 5-Fold Cross-Validated RMSE for Japan. Flourishing indicators are regressed, one at a time, on either LS or CL, using a piecewise polynomial regression with B-splines. Regressions incorporate survey weights, and the cross-validation procedure uses cluster sampling and survey weights to form each fold. Lower values indicate better fit and stronger association. See Section 1.1 for more details.

Flourishing Item	*Life Satisfaction*	*Cantril's Ladder*
Happiness	**2.72**	2.78
Physical Health	**2.57**	2.60
Mental Health	**2.22**	2.24
Meaning in Life	**2.89**	3.01
Purpose in Life	**2.48**	2.50
Promoting the Good	**2.63**	2.66
Delayed Gratification	**2.75**	2.76
Relational Contentment	**2.64**	2.67
Relationship Satisfaction	**2.84**	2.90
Financial Worry	**3.64**	3.68
Material Worry	**3.70**	3.72

Table 11: 5-Fold Cross-Validated RMSE for Kenya. Flourishing indicators are regressed, one at a time, on either LS or CL, using a piecewise polynomial regression with B-splines. Regressions incorporate survey weights, and the cross-validation procedure uses cluster sampling and survey weights to form each fold. Lower values indicate better fit and stronger association. See Section 1.1 for more details.

Flourishing Item	*Life Satisfaction*	*Cantril's Ladder*
Happiness	**1.39**	1.69
Physical Health	**1.71**	1.78
Mental Health	**1.55**	1.70
Meaning in Life	**1.40**	1.67
Purpose in Life	**1.72**	1.84
Promoting the Good	**1.47**	1.50
Delayed Gratification	**1.97**	1.98
Relational Contentment	**1.75**	1.87
Relationship Satisfaction	**1.84**	2.00
Financial Worry	3.56	**3.52**
Material Worry	3.69	**3.66**

Table 12: 5-Fold Cross-Validated RMSE for Mexico. Flourishing indicators are regressed, one at a time, on either LS or CL, using a piecewise polynomial regression with B-splines. Regressions incorporate survey weights, and the cross-validation procedure uses cluster sampling and survey weights to form each fold. Lower values indicate better fit and stronger association. See Section 1.1 for more details.

Flourishing Item	*Life Satisfaction*	*Cantril's Ladder*
Happiness	**2.20**	2.41
Physical Health	**2.09**	2.13
Mental Health	**1.99**	2.02
Meaning in Life	**2.30**	2.47
Purpose in Life	**2.01**	2.03
Promoting the Good	**2.03**	2.06
Delayed Gratification	**1.98**	2.02
Relational Contentment	**2.26**	2.30
Relationship Satisfaction	**2.29**	2.36
Financial Worry	**2.99**	3.09
Material Worry	**3.07**	3.13

Table 13: 5-Fold Cross-Validated RMSE for Nigeria. Flourishing indicators are regressed, one at a time, on either LS or CL, using a piecewise polynomial regression with B-splines. Regressions incorporate survey weights, and the cross-validation procedure uses cluster sampling and survey weights to form each fold. Lower values indicate better fit and stronger association. See Section 1.1 for more details.

Flourishing Item	*Life Satisfaction*	*Cantril's Ladder*
Happiness	**1.88**	2.18
Physical Health	**1.95**	2.06
Mental Health	**1.81**	1.96
Meaning in Life	**2.12**	2.28
Purpose in Life	**1.92**	2.00
Promoting the Good	**1.86**	1.93
Delayed Gratification	**1.87**	1.93
Relational Contentment	**1.88**	1.98
Relationship Satisfaction	**1.88**	2.00
Financial Worry	**3.19**	3.23
Material Worry	**3.18**	3.22

Table 14: 5-Fold Cross-Validated RMSE for Philippines. Flourishing indicators are regressed, one at a time, on either LS or CL, using a piecewise polynomial regression with B-splines. Regressions incorporate survey weights, and the cross-validation procedure uses cluster sampling and survey weights to form each fold. Lower values indicate better fit and stronger association. See Section 1.1 for more details.

Flourishing Item	*Life Satisfaction*	*Cantril's Ladder*
Happiness	**1.06**	1.31
Physical Health	**1.63**	1.78
Mental Health	**1.45**	1.70
Meaning in Life	**1.36**	1.57
Purpose in Life	**1.78**	1.90
Promoting the Good	**1.47**	1.55
Delayed Gratification	2.27	**2.25**
Relational Contentment	**1.53**	1.69
Relationship Satisfaction	**1.55**	1.72
Financial Worry	**2.19**	2.25
Material Worry	**2.18**	2.27

Table 15: 5-Fold Cross-Validated RMSE for Poland. Flourishing indicators are regressed, one at a time, on either LS or CL, using a piecewise polynomial regression with B-splines. Regressions incorporate survey weights, and the cross-validation procedure uses cluster sampling and survey weights to form each fold. Lower values indicate better fit and stronger association. See Section 1.1 for more details.

Flourishing Item	*Life Satisfaction*	*Cantril's Ladder*
Happiness	**2.29**	2.34
Physical Health	2.62	2.62
Mental Health	**2.41**	2.43
Meaning in Life	**2.45**	2.59
Purpose in Life	2.34	2.34
Promoting the Good	**2.26**	2.31
Delayed Gratification	**2.36**	2.40
Relational Contentment	**2.41**	2.44
Relationship Satisfaction	**2.51**	2.63
Financial Worry	**3.08**	3.09
Material Worry	**3.24**	3.30

Table 16: 5-Fold Cross-Validated RMSE for South Africa. Flourishing indicators are regressed, one at a time, on either LS or CL, using a piecewise polynomial regression with B-splines. Regressions incorporate survey weights, and the cross-validation procedure uses cluster sampling and survey weights to form each fold. Lower values indicate better fit and stronger association. See Section 1.1 for more details.

Flourishing Item	*Life Satisfaction*	*Cantril's Ladder*
Happiness	**1.14**	1.29
Physical Health	1.91	1.91
Mental Health	**1.91**	1.98
Meaning in Life	**1.40**	1.69
Purpose in Life	**2.03**	2.08
Promoting the Good	1.63	1.63
Delayed Gratification	**1.95**	1.96
Relational Contentment	**1.86**	1.92
Relationship Satisfaction	**2.08**	2.15
Financial Worry	3.16	**3.14**
Material Worry	3.18	3.18

Table 17: 5-Fold Cross-Validated RMSE for Spain. Flourishing indicators are regressed, one at a time, on either LS or CL, using a piecewise polynomial regression with B-splines. Regressions incorporate survey weights, and the cross-validation procedure uses cluster sampling and survey weights to form each fold. Lower values indicate better fit and stronger association. See Section 1.1 for more details.

Flourishing Item	*Life Satisfaction*	*Cantril's Ladder*
Happiness	**0.98**	1.30
Physical Health	1.83	**1.82**
Mental Health	**1.59**	1.74
Meaning in Life	**1.39**	1.63
Purpose in Life	**2.09**	2.18
Promoting the Good	**1.57**	1.58
Delayed Gratification	1.99	1.99
Relational Contentment	**1.72**	1.85
Relationship Satisfaction	**1.88**	2.02
Financial Worry	2.55	**2.54**
Material Worry	2.38	**2.37**

Table 18: 5-Fold Cross-Validated RMSE for Sweden. Flourishing indicators are regressed, one at a time, on either LS or CL, using a piecewise polynomial regression with B-splines. Regressions incorporate survey weights, and the cross-validation procedure uses cluster sampling and survey weights to form each fold. Lower values indicate better fit and stronger association. See Section 1.1 for more details.

Flourishing Item	*Life Satisfaction*	*Cantril's Ladder*
Happiness	**2.91**	3.05
Physical Health	**2.90**	2.94
Mental Health	**2.04**	2.08
Meaning in Life	**2.92**	3.09
Purpose in Life	**2.89**	2.91
Promoting the Good	**2.64**	2.67
Delayed Gratification	**3.16**	3.17
Relational Contentment	**2.96**	3.02
Relationship Satisfaction	**3.16**	3.25
Financial Worry	**3.49**	3.53
Material Worry	**3.53**	3.56

Table 19: 5-Fold Cross-Validated RMSE for Tanzania. Flourishing indicators are regressed, one at a time, on either LS or CL, using a piecewise polynomial regression with B-splines. Regressions incorporate survey weights, and the cross-validation procedure uses cluster sampling and survey weights to form each fold. Lower values indicate better fit and stronger association. See Section 1.1 for more details.

Flourishing Item	*Life Satisfaction*	*Cantril's Ladder*
Happiness	**1.92**	2.51
Physical Health	**2.57**	2.58
Mental Health	**2.53**	2.76
Meaning in Life	**2.52**	2.78
Purpose in Life	**2.71**	2.75
Promoting the Good	**2.26**	2.29
Delayed Gratification	**2.57**	2.59
Relational Contentment	**2.76**	2.78
Relationship Satisfaction	**2.70**	2.76
Financial Worry	**3.03**	3.17
Material Worry	**3.17**	3.33

Table 20: 5-Fold Cross-Validated RMSE for Turkey. Flourishing indicators are regressed, one at a time, on either LS or CL, using a piecewise polynomial regression with B-splines. Regressions incorporate survey weights, and the cross-validation procedure uses cluster sampling and survey weights to form each fold. Lower values indicate better fit and stronger association. See Section 1.1 for more details.

Flourishing Item	*Life Satisfaction*	*Cantril's Ladder*
Happiness	**1.11**	1.45
Physical Health	**2.05**	2.06
Mental Health	**1.92**	2.08
Meaning in Life	**1.40**	1.77
Purpose in Life	**2.16**	2.30
Promoting the Good	**1.66**	1.70
Delayed Gratification	**1.90**	1.93
Relational Contentment	**1.88**	2.02
Relationship Satisfaction	**2.02**	2.17
Financial Worry	**2.74**	2.79
Material Worry	**2.58**	2.62

Table 21: 5-Fold Cross-Validated RMSE for the United Kingdom. Flourishing indicators are regressed, one at a time, on either LS or CL, using a piecewise polynomial regression with B-splines. Regressions incorporate survey weights, and the cross-validation procedure uses cluster sampling and survey weights to form each fold. Lower values indicate better fit and stronger association. See Section 1.1 for more details.

Flourishing Item	*Life Satisfaction*	*Cantril's Ladder*
Happiness	**1.03**	1.36
Physical Health	1.68	**1.67**
Mental Health	**1.60**	1.80
Meaning in Life	**1.39**	1.67
Purpose in Life	**2.08**	2.21
Promoting the Good	**1.55**	1.56
Delayed Gratification	**1.82**	1.83
Relational Contentment	**1.81**	1.96
Relationship Satisfaction	**1.94**	2.11
Financial Worry	2.70	**2.63**
Material Worry	2.57	**2.52**

Table 22: 5-Fold Cross-Validated RMSE for the United States. Flourishing indicators are regressed, one at a time, on either LS or CL, using a piecewise polynomial regression with B-splines. Regressions incorporate survey weights, and the cross-validation procedure uses cluster sampling and survey weights to form each fold. Lower values indicate better fit and stronger association. See Section 1.1 for more details.

Section 3

Scatterplots for Argentina

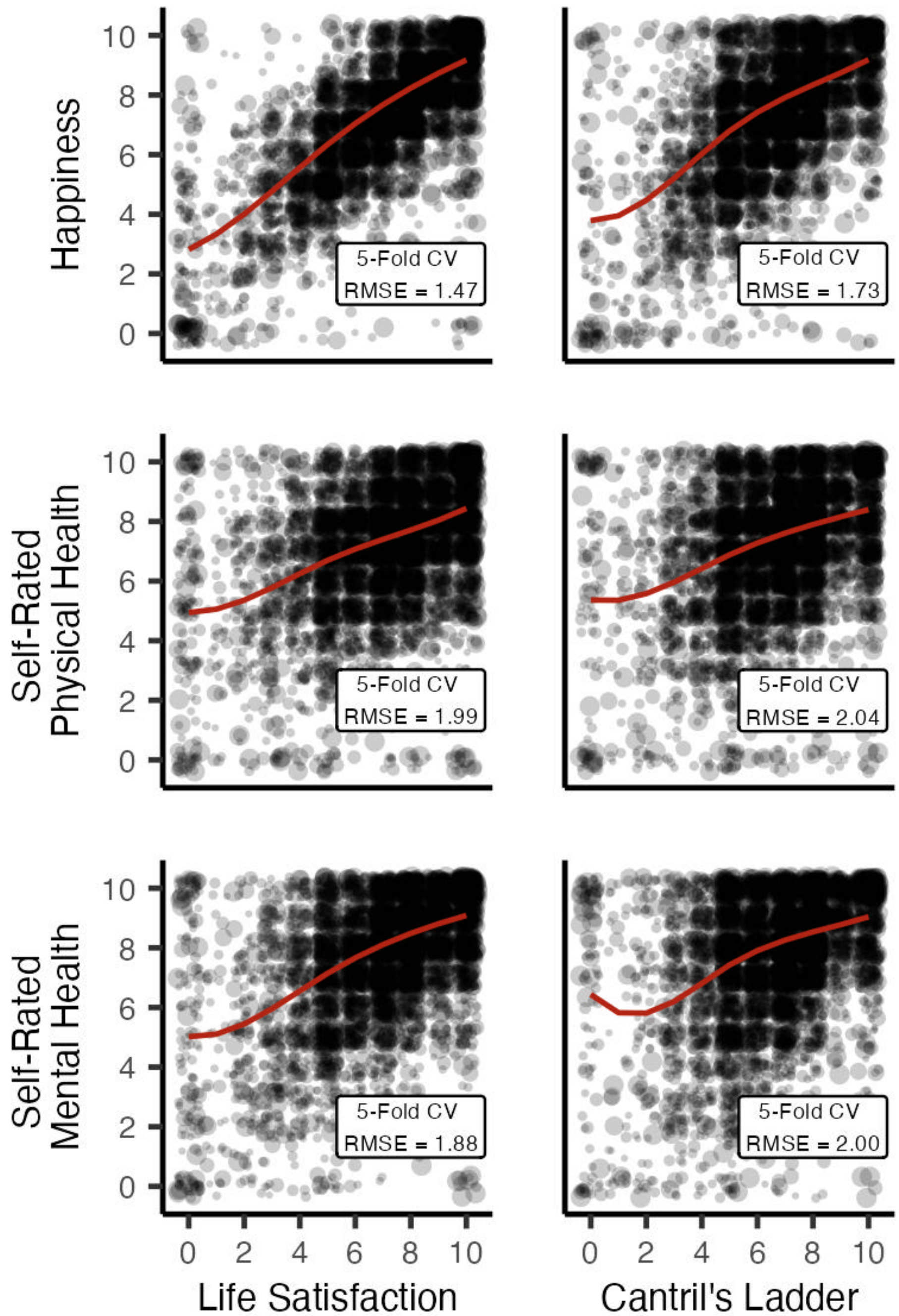

Figure 1: Scatterplots for Argentina, Panel 1. Curves fit using a piecewise polynomial regression with B-splines. Regressions incorporate survey weights, and the cross-validation procedure uses cluster sampling and survey weights to form each fold. See Section 1.1 for more details.

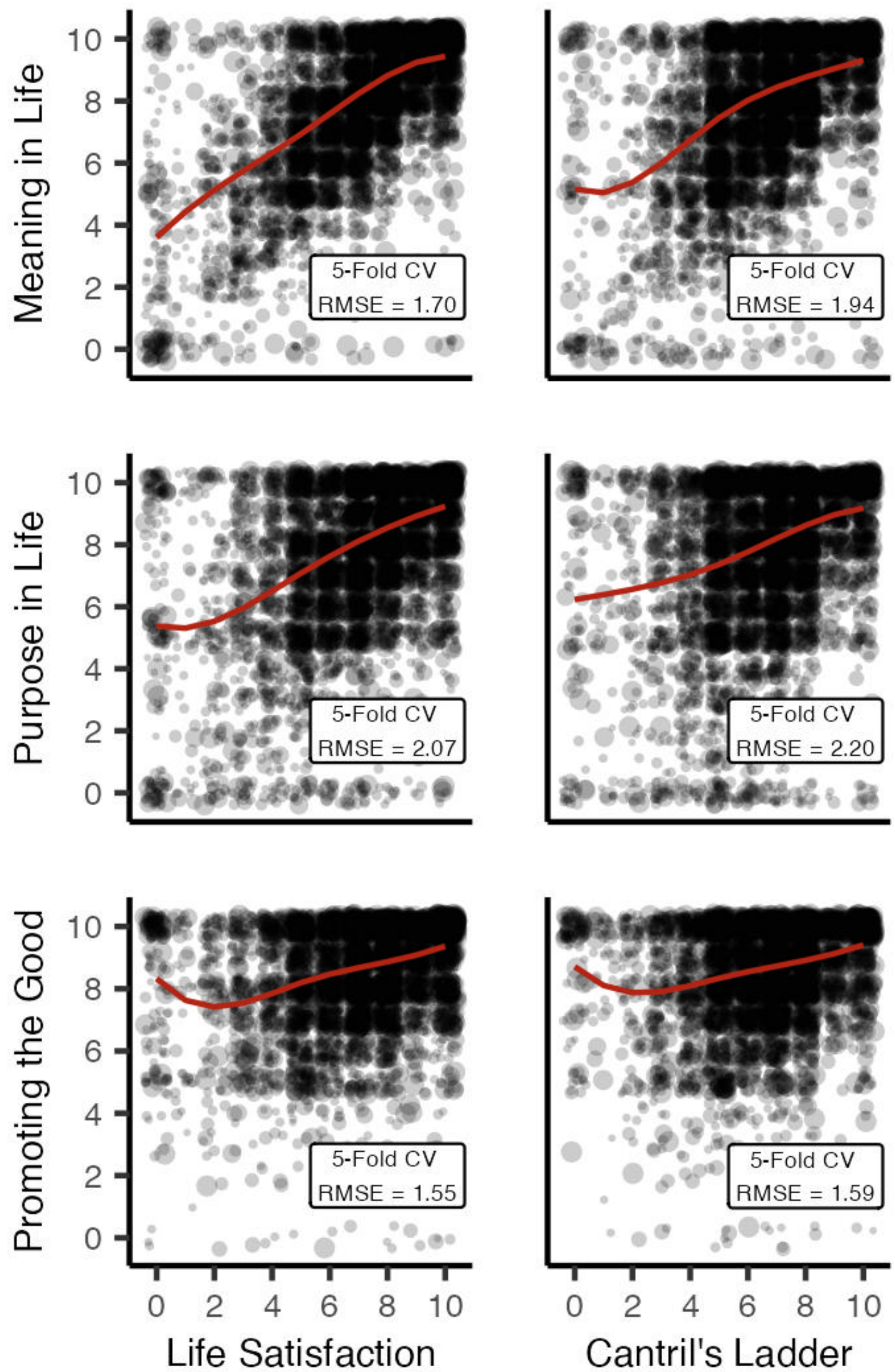

Figure 2: Scatterplots for Argentina, Panel 2. Curves fit using a piecewise polynomial regression with B-splines. Regressions incorporate survey weights, and the cross-validation procedure uses cluster sampling and survey weights to form each fold. See Section 1.1 for more details.

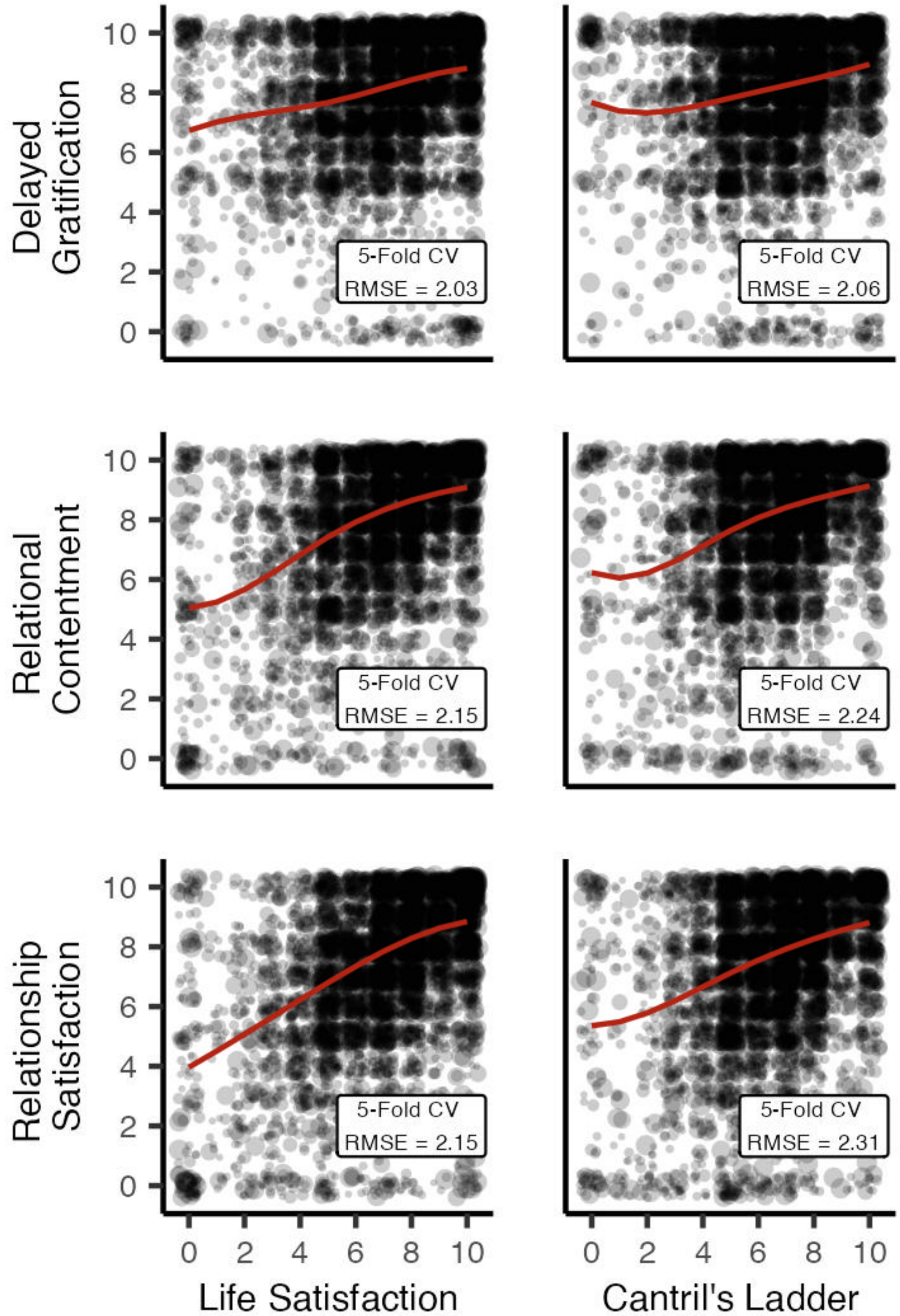

Figure 3: Scatterplots for Argentina, Panel 3. Curves fit using a piecewise polynomial regression with B-splines. Regressions incorporate survey weights, and the cross-validation procedure uses cluster sampling and survey weights to form each fold. See Section 1.1 for more details.

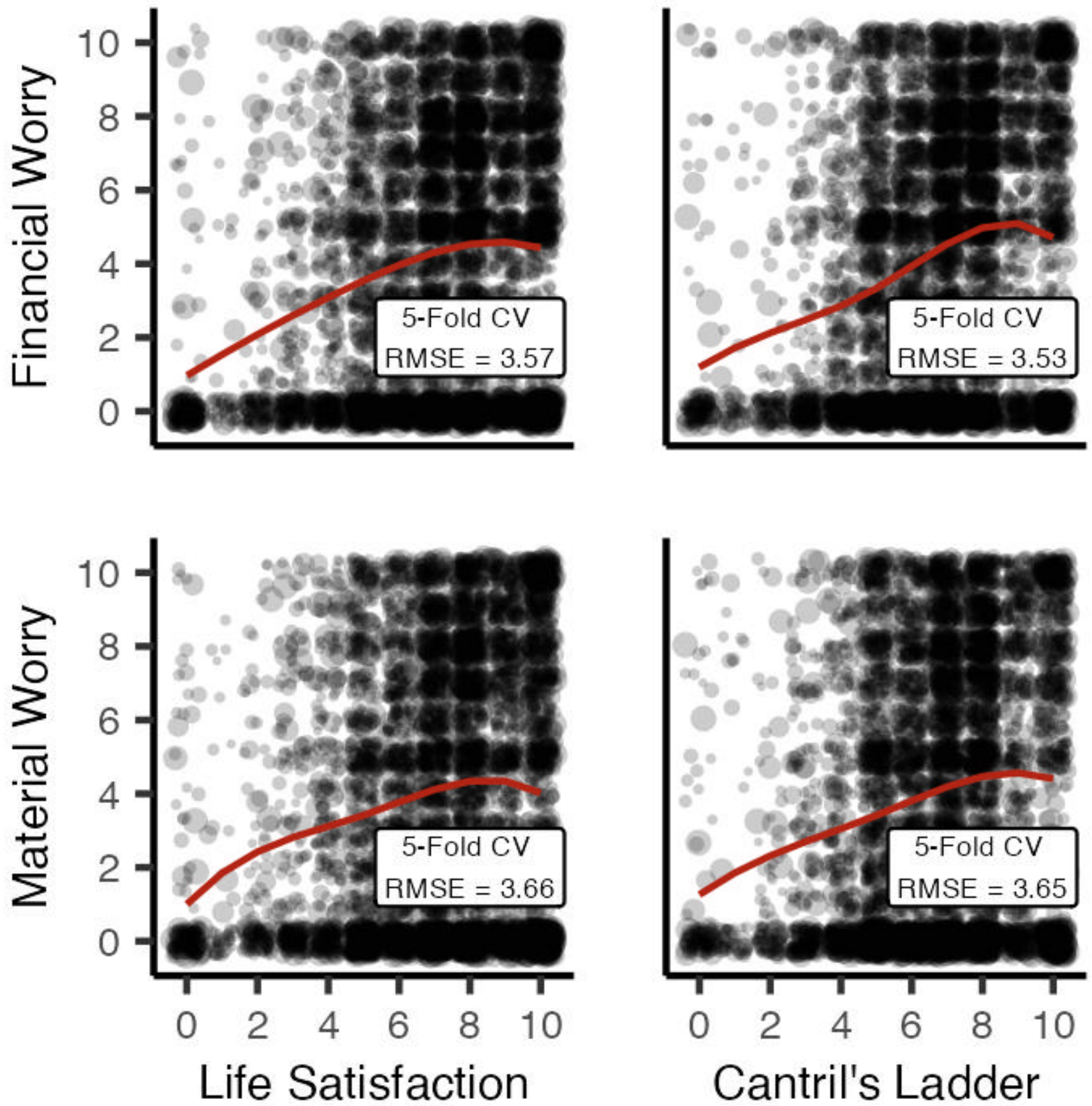

Figure 4: Scatterplots for Argentina, Panel 4. Curves fit using a piecewise polynomial regression with B-splines. Regressions incorporate survey weights, and the cross-validation procedure uses cluster sampling and survey weights to form each fold. See Section 1.1 for more details.

Section 4

Scatterplots for Australia

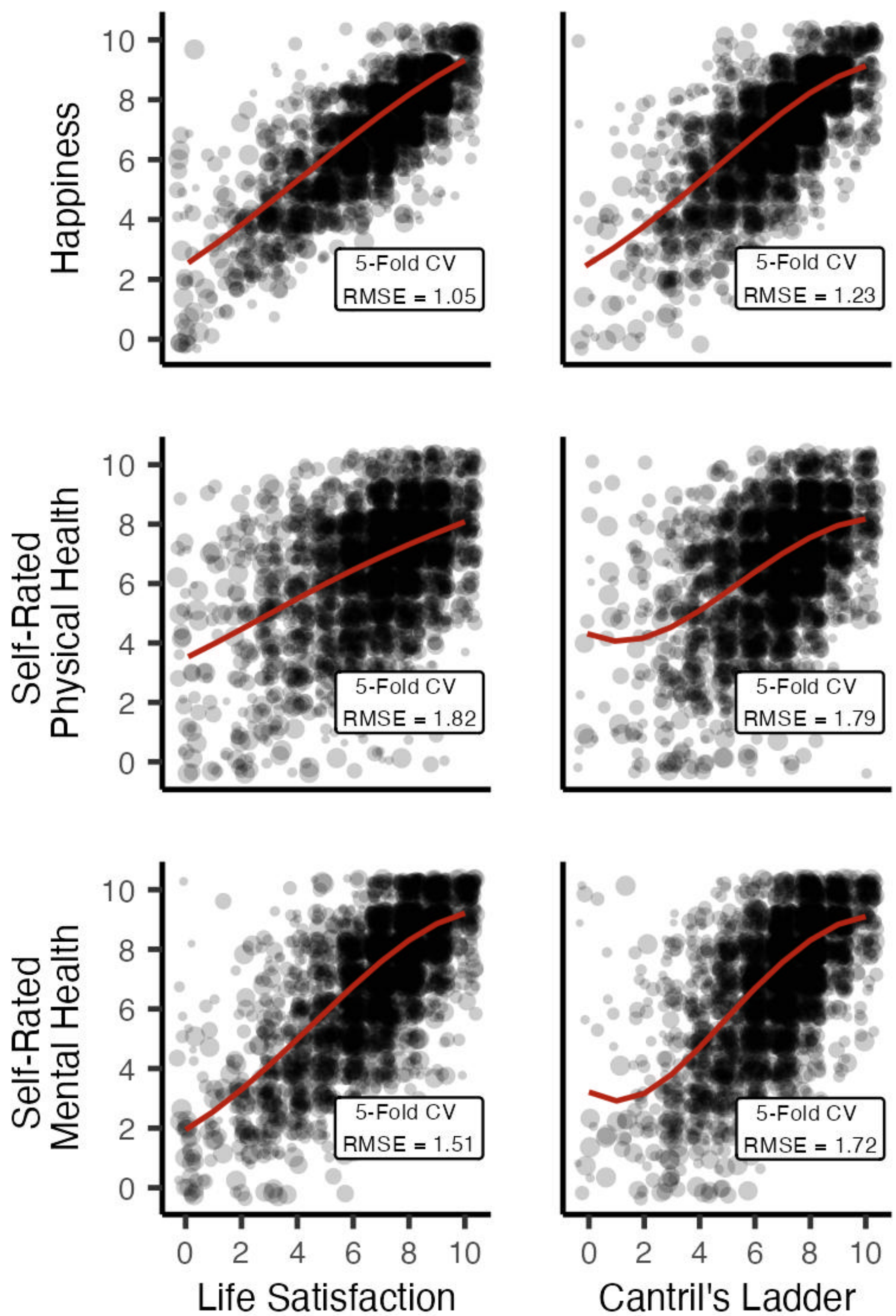

Figure 5: Scatterplots for Australia, Panel 1. Curves fit using a piecewise polynomial regression with B-splines. Regressions incorporate survey weights, and the cross-validation procedure uses cluster sampling and survey weights to form each fold. See Section 1.1 for more details.

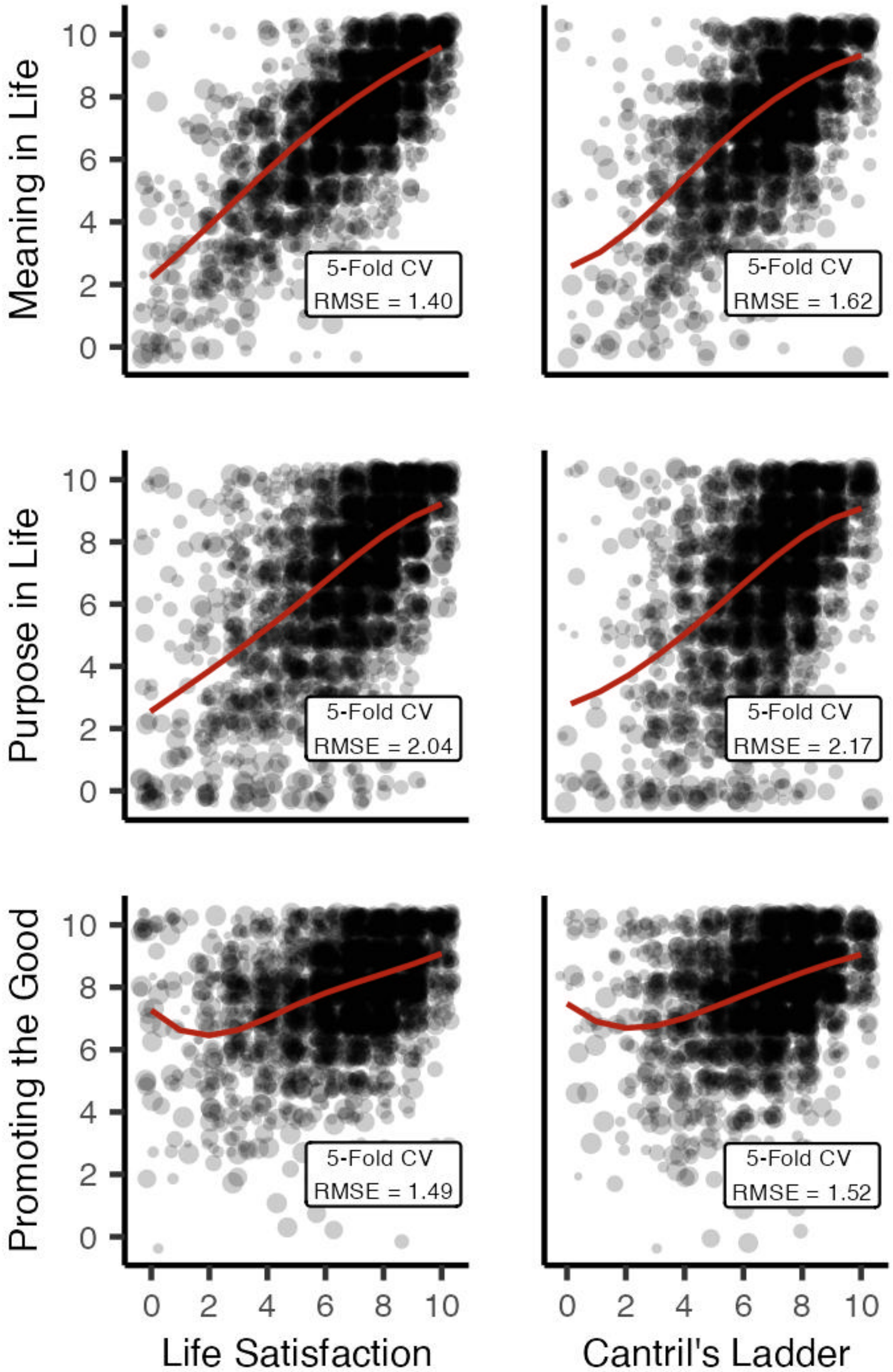

Figure 6: Scatterplots for Australia, Panel 2. Curves fit using a piecewise polynomial regression with B-splines. Regressions incorporate survey weights, and the cross-validation procedure uses cluster sampling and survey weights to form each fold. See Section 1.1 for more details.

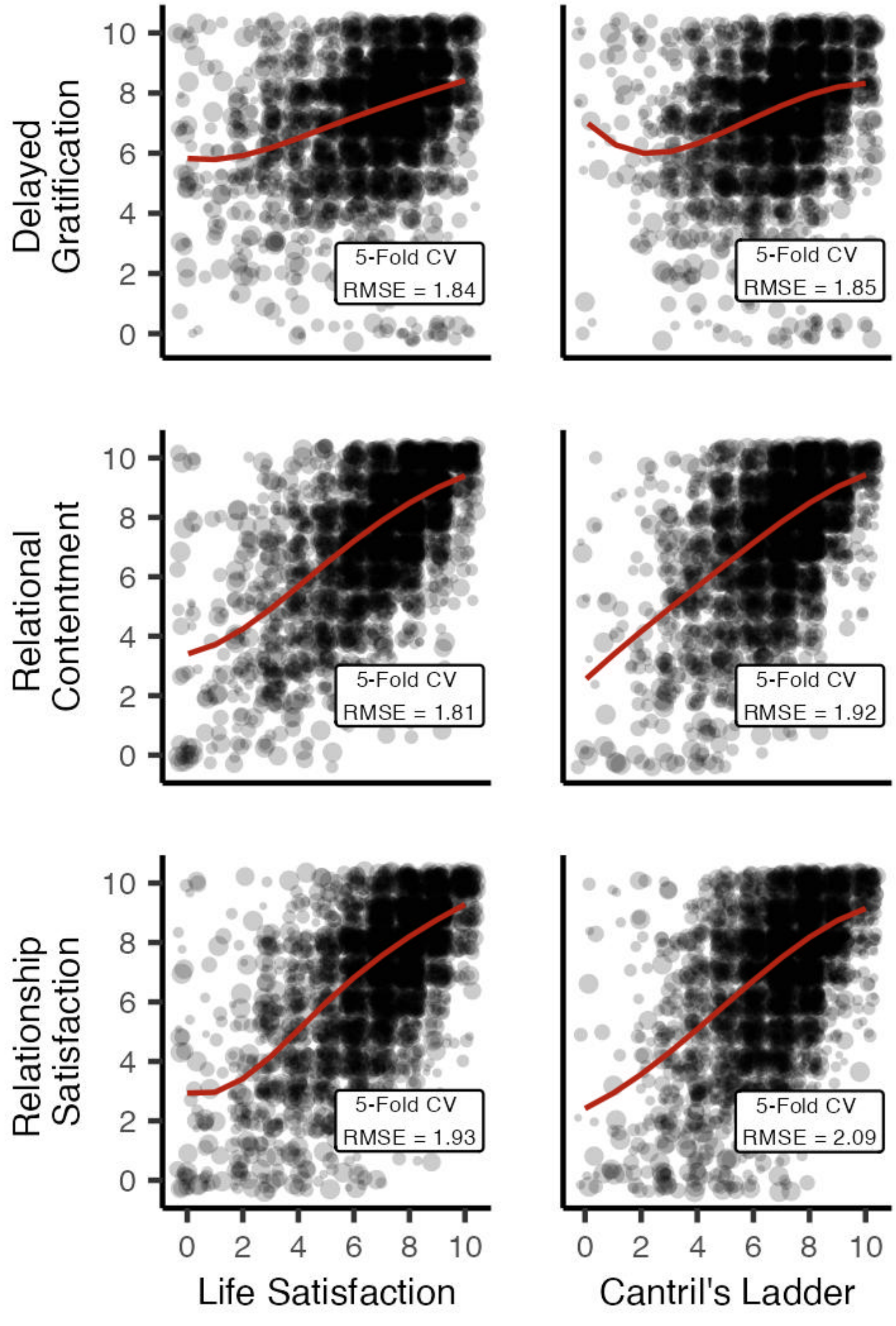

Figure 7: Scatterplots for Australia, Panel 3. Curves fit using a piecewise polynomial regression with B-splines. Regressions incorporate survey weights, and the cross-validation procedure uses cluster sampling and survey weights to form each fold. See Section 1.1 for more details.

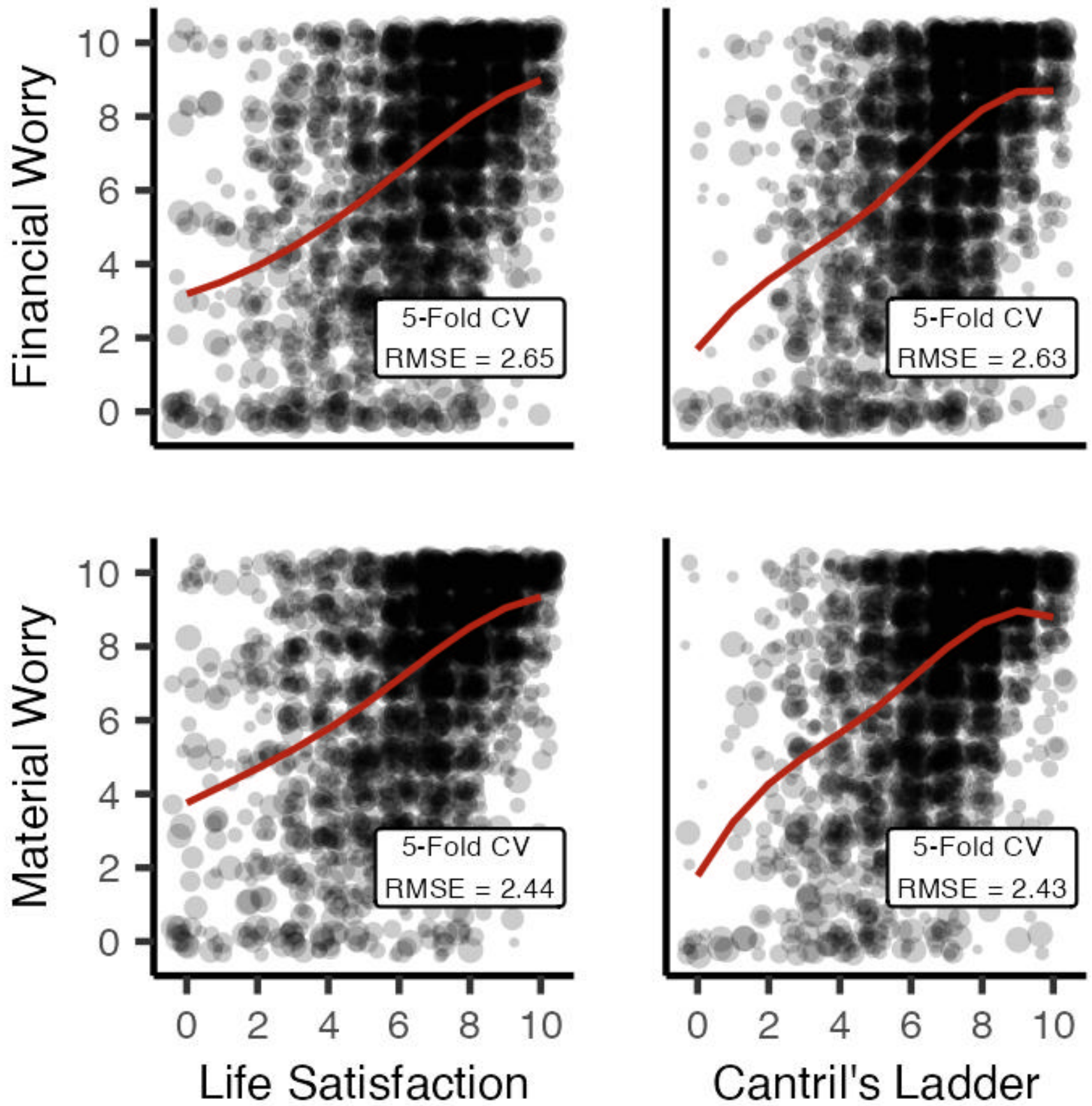

Figure 8: Scatterplots for Australia, Panel 4. Curves fit using a piecewise polynomial regression with B-splines. Regressions incorporate survey weights, and the cross-validation procedure uses cluster sampling and survey weights to form each fold. See Section 1.1 for more details.

Section 5

Scatterplots for Brazil

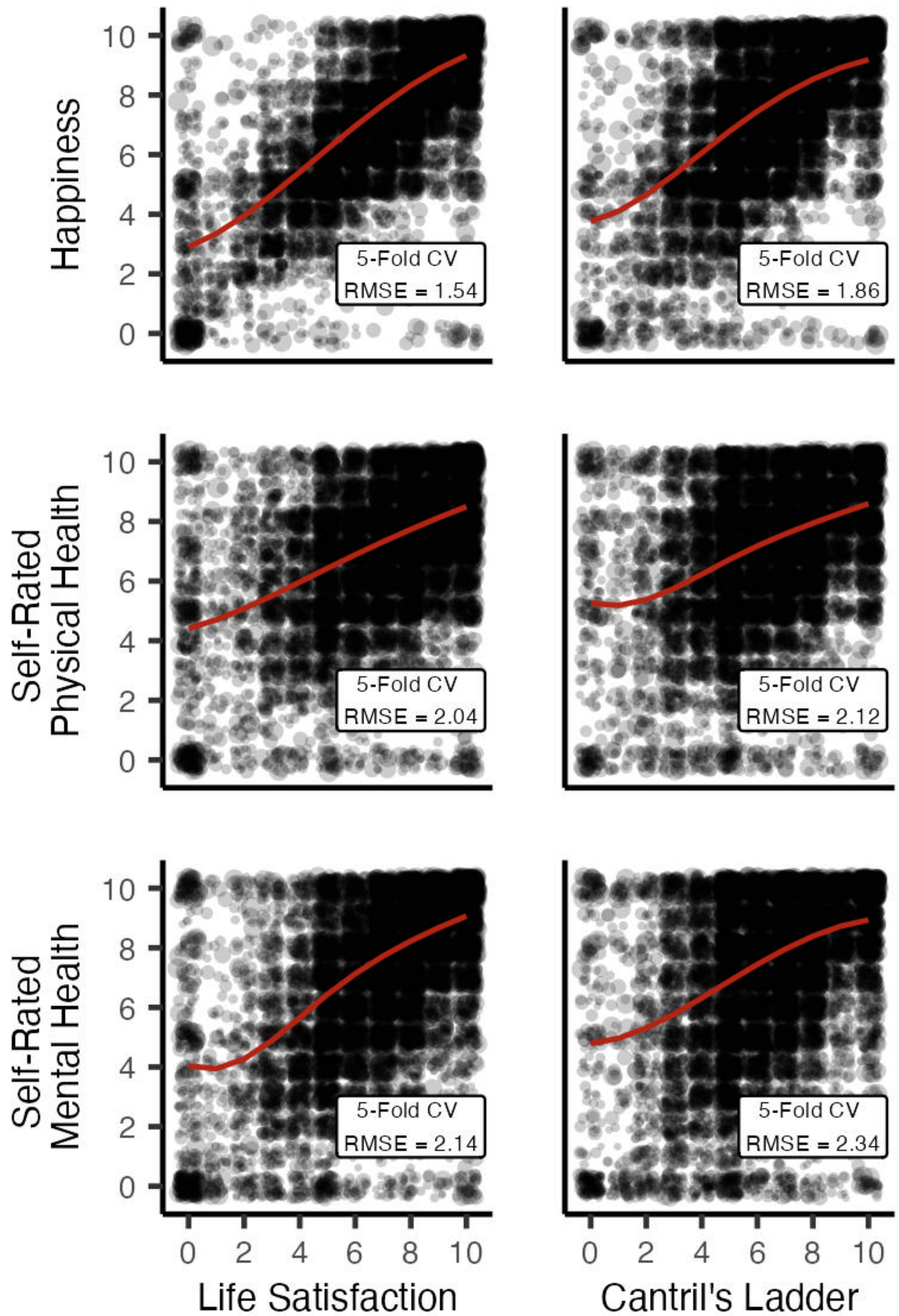

Figure 9: Scatterplots for Brazil, Panel 1. Curves fit using a piecewise polynomial regression with B-splines. Regressions incorporate survey weights, and the cross-validation procedure uses cluster sampling and survey weights to form each fold. See Section 1.1 for more details.

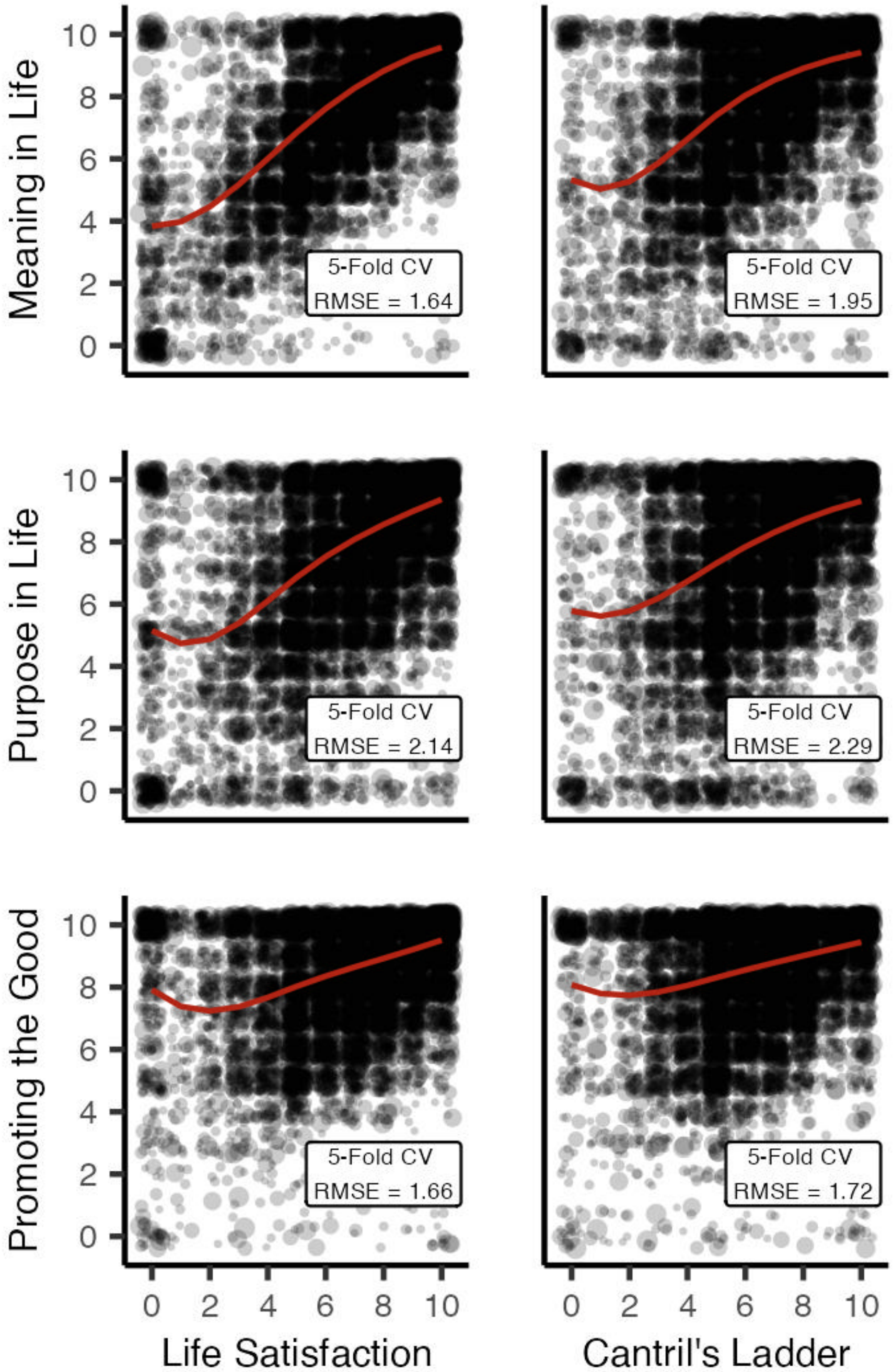

Figure 10: Scatterplots for Brazil, Panel 2. Curves fit using a piecewise polynomial regression with B-splines. Regressions incorporate survey weights, and the cross-validation procedure uses cluster sampling and survey weights to form each fold. See Section 1.1 for more details.

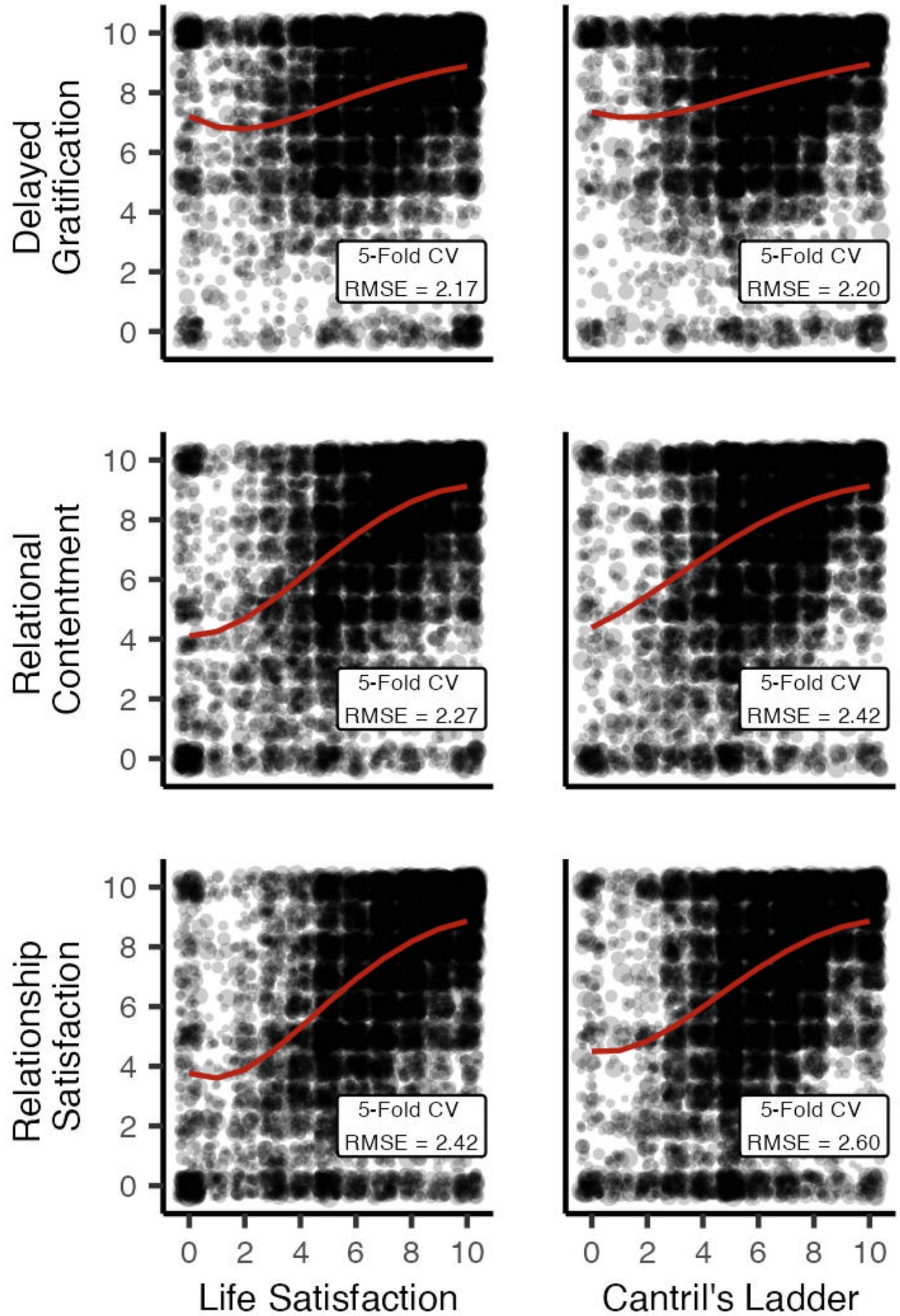

Figure 11: Scatterplots for Brazil, Panel 3. Curves fit using a piecewise polynomial regression with B-splines. Regressions incorporate survey weights, and the cross-validation procedure uses cluster sampling and survey weights to form each fold. See Section 1.1 for more details.

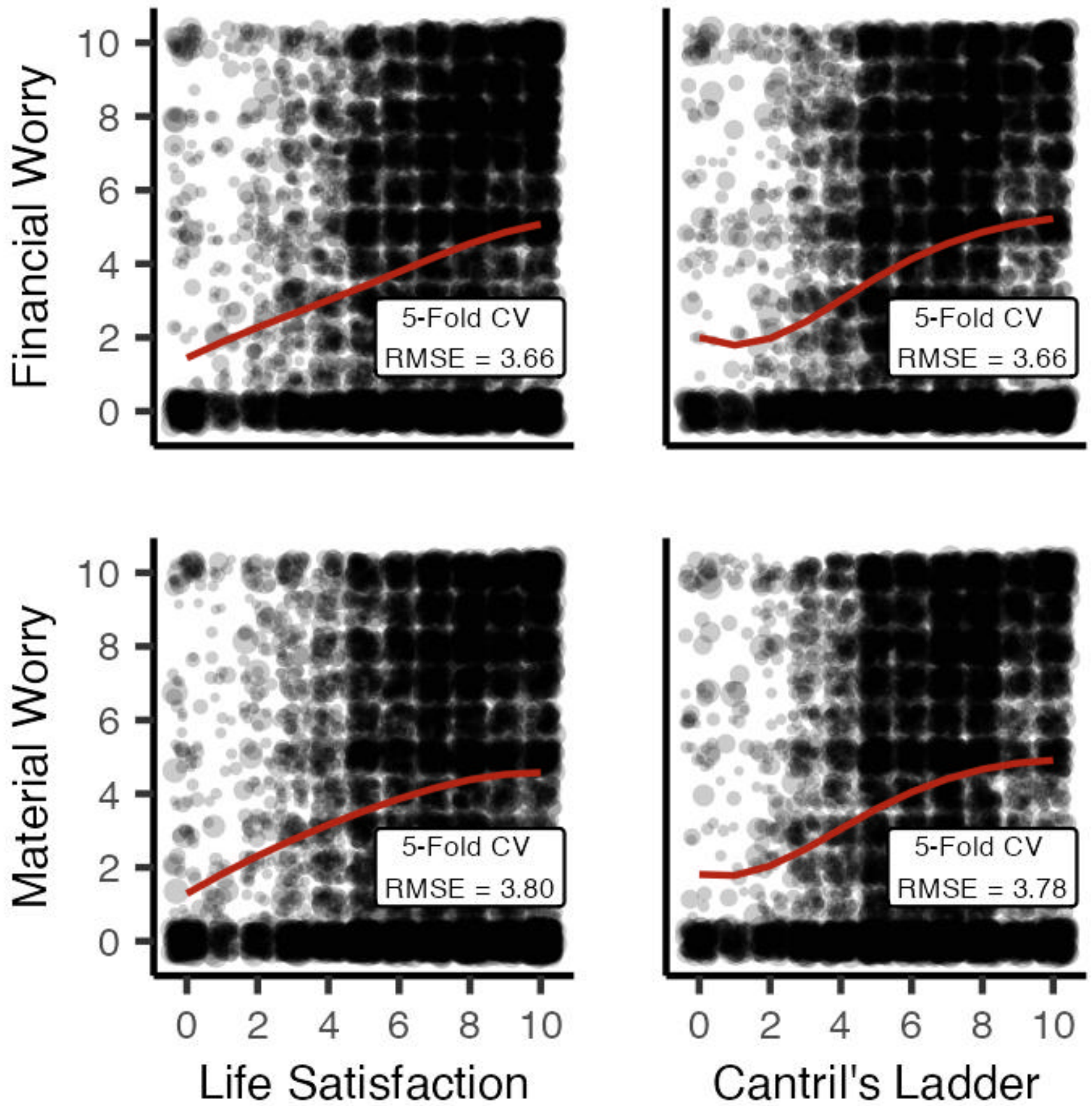

Figure 12: Scatterplots for Brazil, Panel 4. Curves fit using a piecewise polynomial regression with B-splines. Regressions incorporate survey weights, and the cross-validation procedure uses cluster sampling and survey weights to form each fold. See Section 1.1 for more details.

Section 6

Scatterplots for Egypt

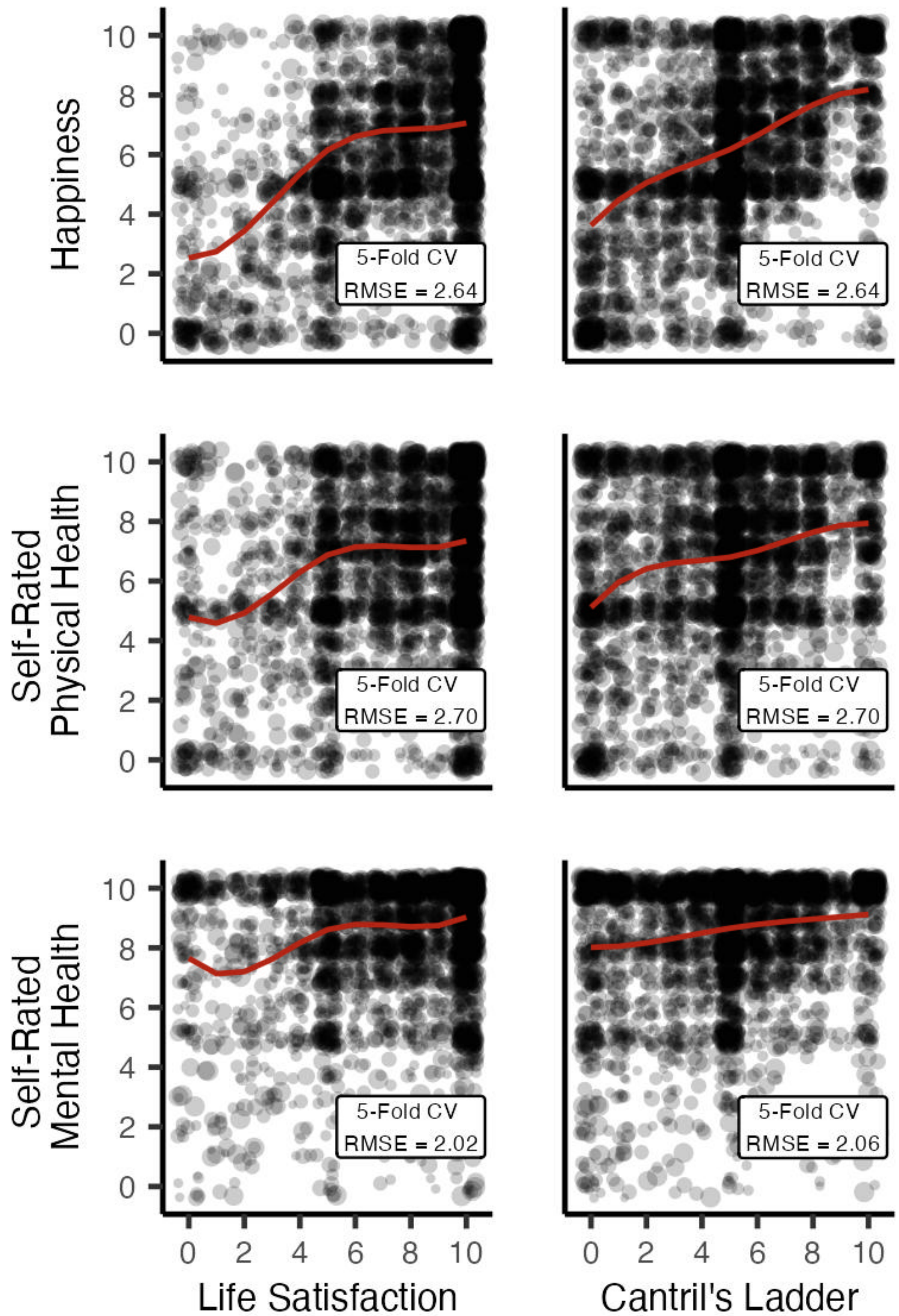

Figure 13: Scatterplots for Egypt, Panel 1. Curves fit using a piecewise polynomial regression with B-splines. Regressions incorporate survey weights, and the cross-validation procedure uses cluster sampling and survey weights to form each fold. See Section 1.1 for more details.

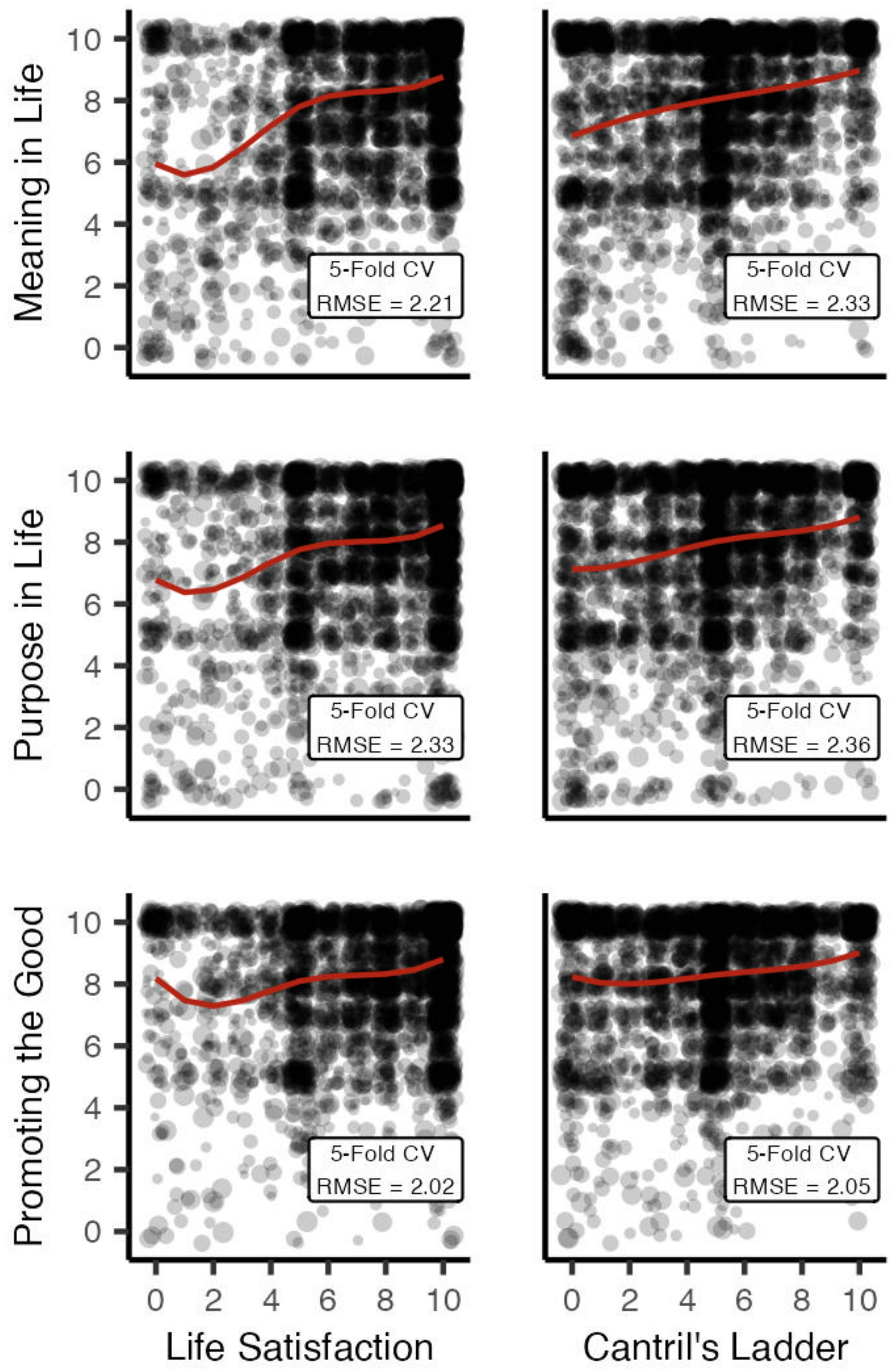

Figure 14: Scatterplots for Egypt, Panel 2. Curves fit using a piecewise polynomial regression with B-splines. Regressions incorporate survey weights, and the cross-validation procedure uses cluster sampling and survey weights to form each fold. See Section 1.1 for more details.

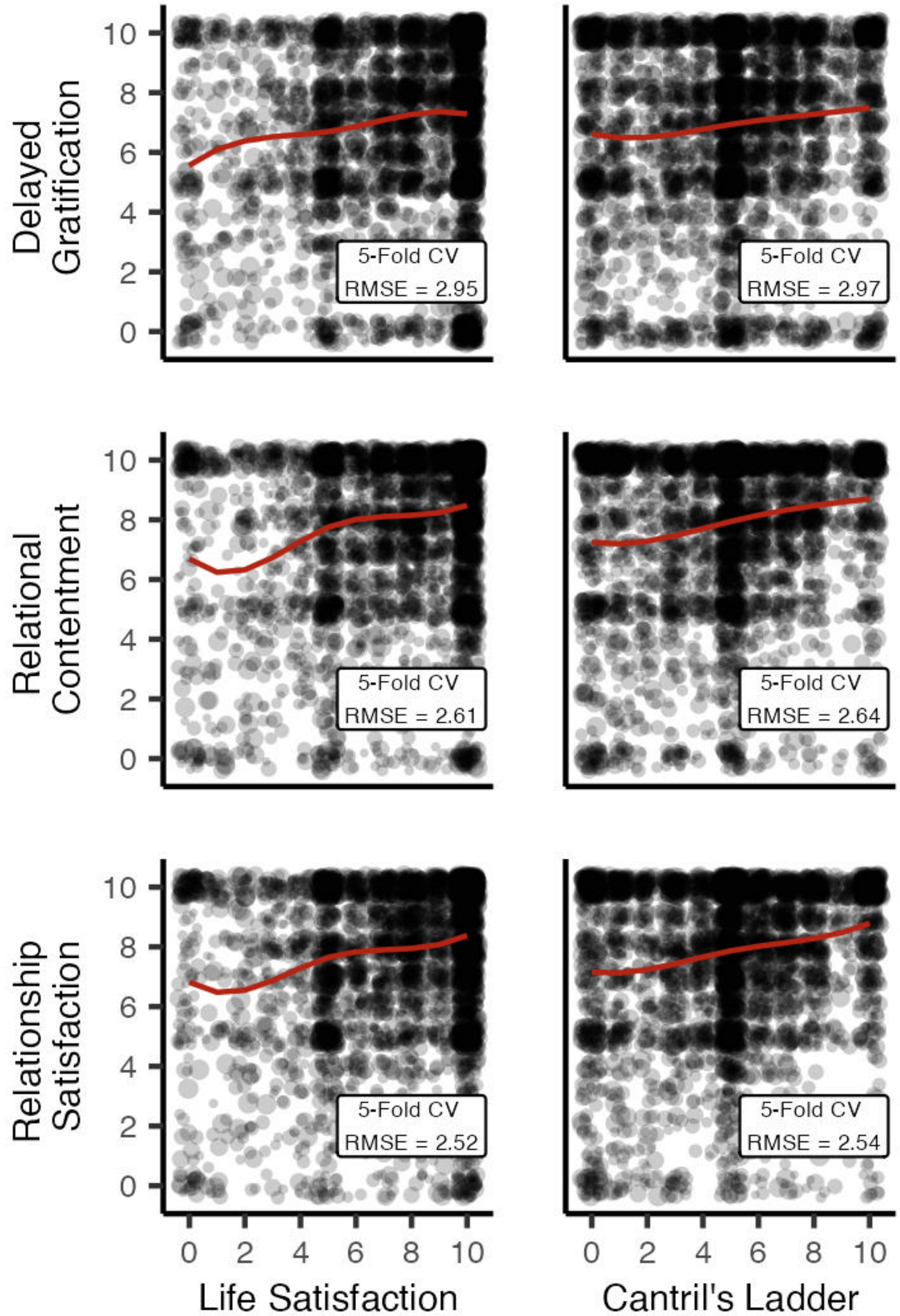

Figure 15: Scatterplots for Egypt, Panel 3. Curves fit using a piecewise polynomial regression with B-splines. Regressions incorporate survey weights, and the cross-validation procedure uses cluster sampling and survey weights to form each fold. See Section 1.1 for more details.

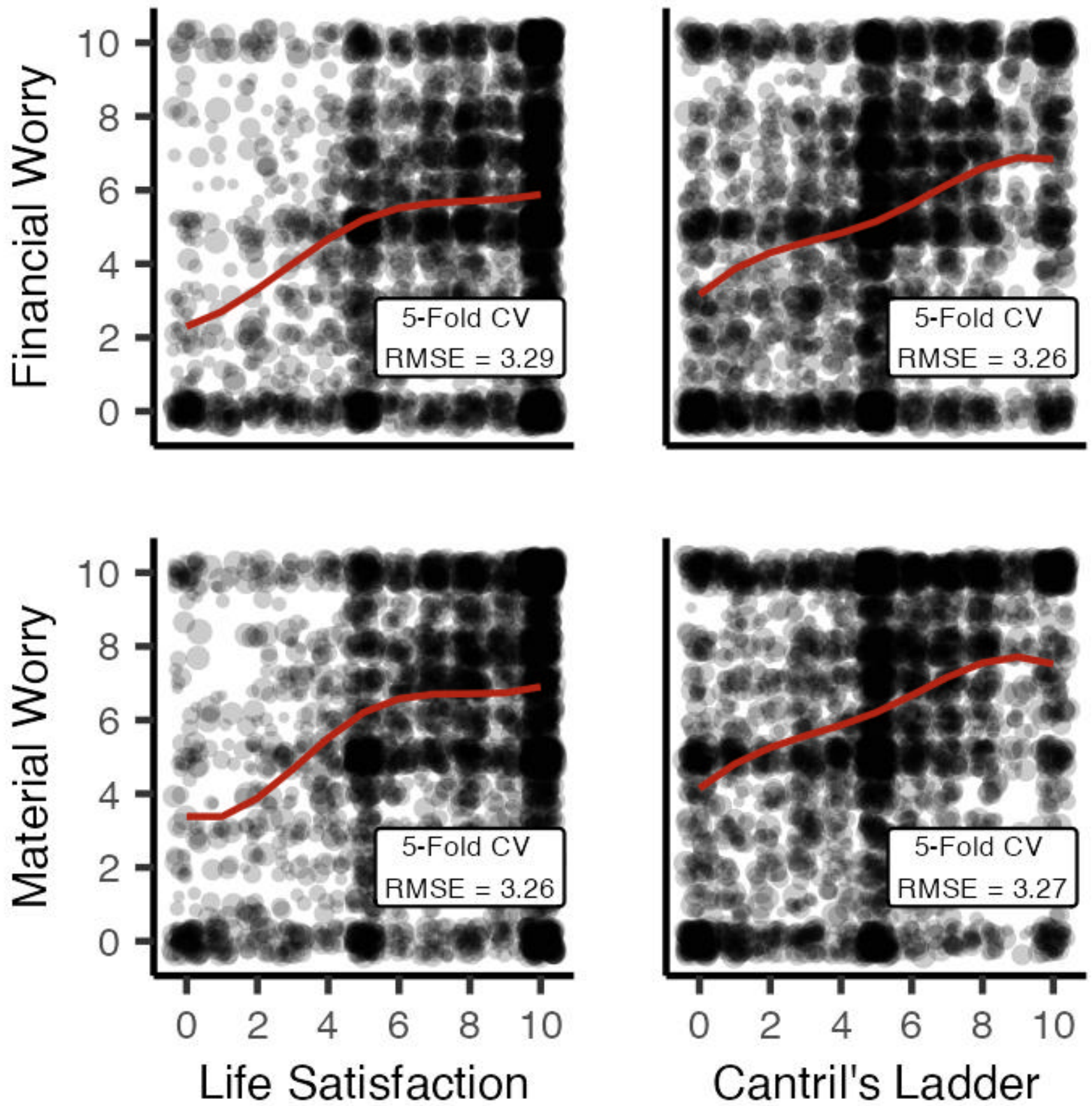

Figure 16: Scatterplots for Egypt, Panel 4. Curves fit using a piecewise polynomial regression with B-splines. Regressions incorporate survey weights, and the cross-validation procedure uses cluster sampling and survey weights to form each fold. See Section 1.1 for more details.

Section 7

Scatterplots for Germany

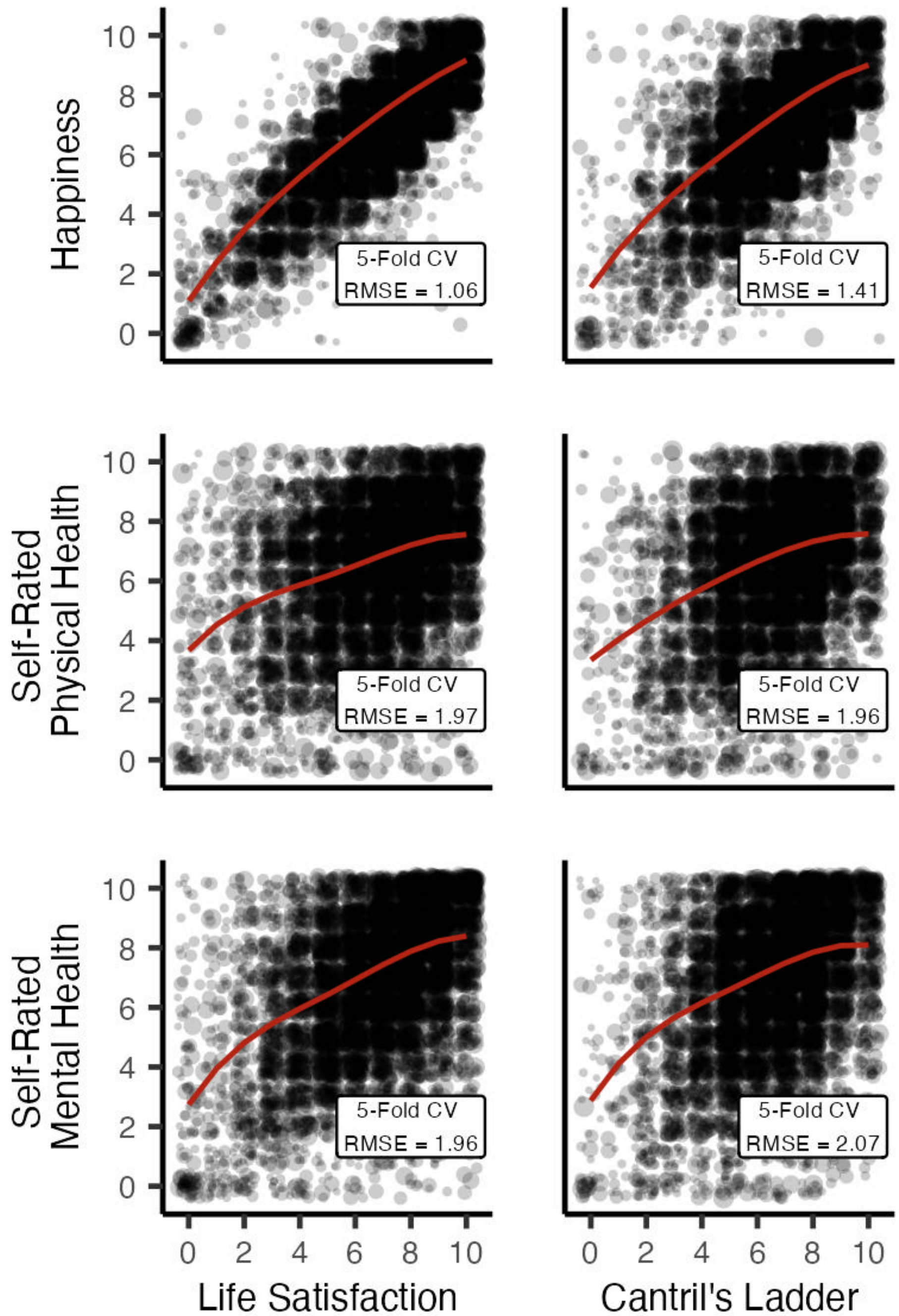

Figure 17: Scatterplots for Germany, Panel 1. Curves fit using a piecewise polynomial regression with B-splines. Regressions incorporate survey weights, and the cross-validation procedure uses cluster sampling and survey weights to form each fold. See Section 1.1 for more details.

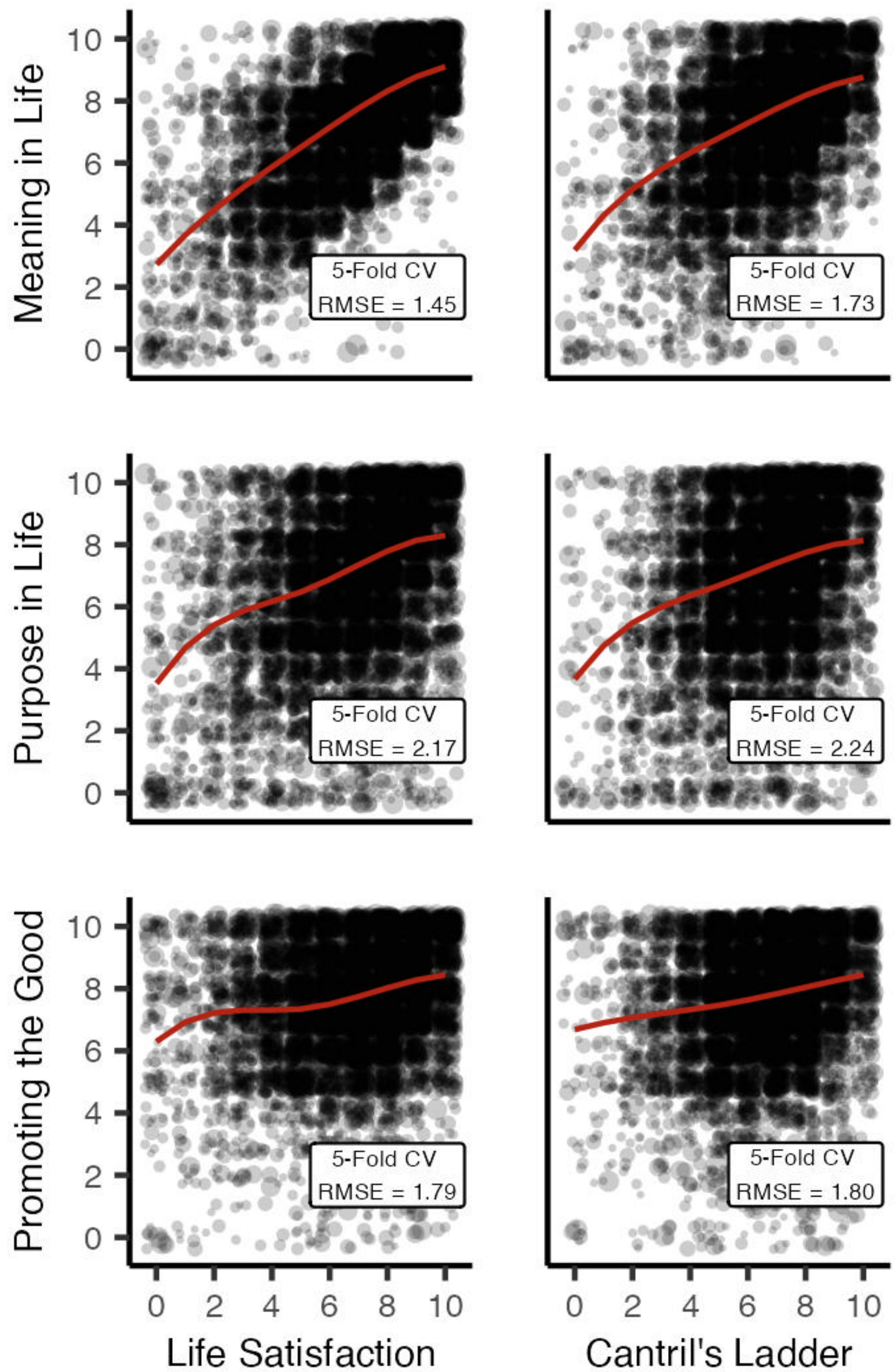

Figure 18: Scatterplots for Germany, Panel 2. Curves fit using a piecewise polynomial regression with B-splines. Regressions incorporate survey weights, and the cross-validation procedure uses cluster sampling and survey weights to form each fold. See Section 1.1 for more details.

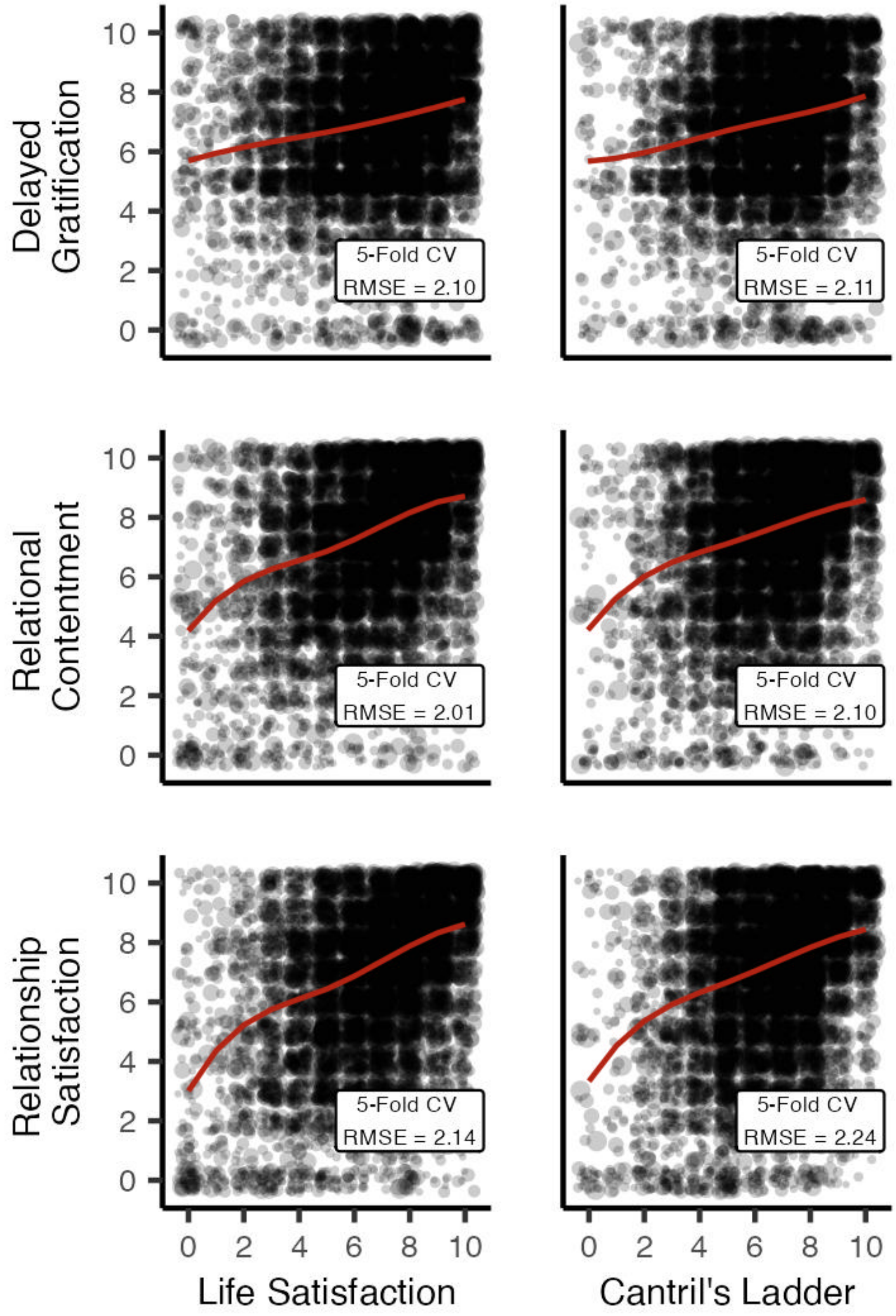

Figure 19: Scatterplots for Germany, Panel 3. Curves fit using a piecewise polynomial regression with B-splines. Regressions incorporate survey weights, and the cross-validation procedure uses cluster sampling and survey weights to form each fold. See Section 1.1 for more details.

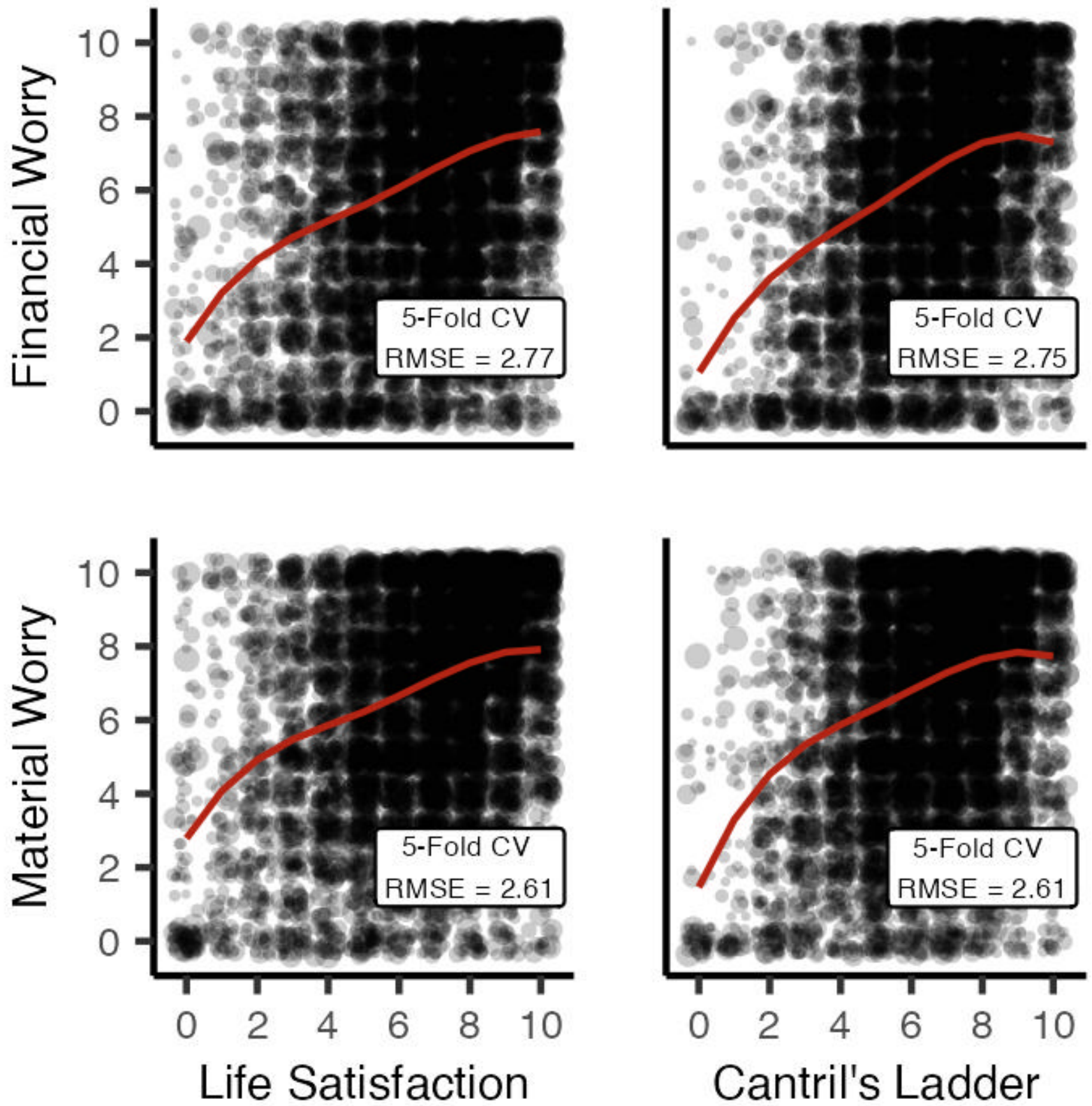

Figure 20: Scatterplots for Germany, Panel 4. Curves fit using a piecewise polynomial regression with B-splines. Regressions incorporate survey weights, and the cross-validation procedure uses cluster sampling and survey weights to form each fold. See Section 1.1 for more details.

Section 8

Scatterplots for Hong Kong

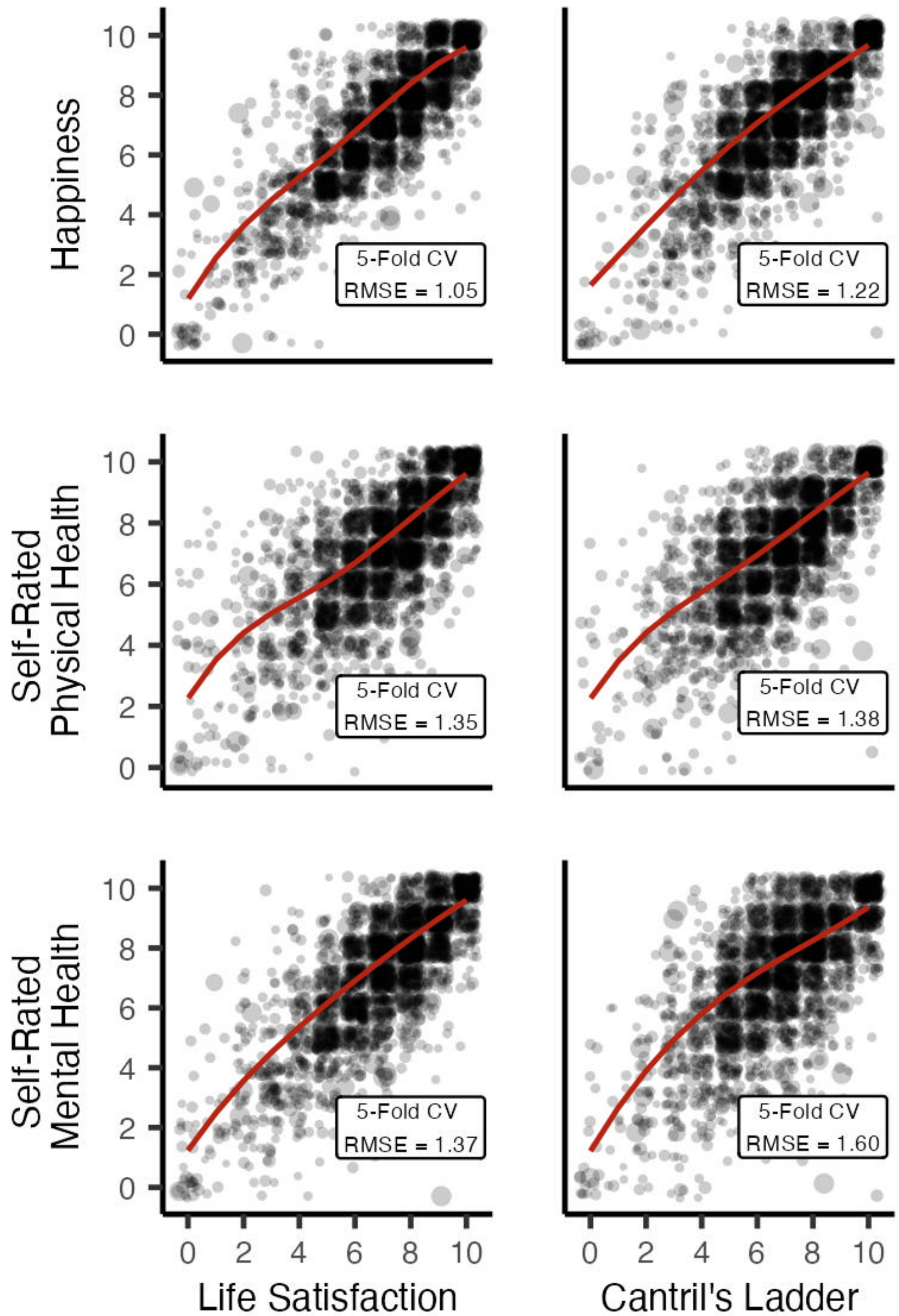

Figure 21: Scatterplots for Hong Kong, Panel 1. Curves fit using a piecewise polynomial regression with B-splines. Regressions incorporate survey weights, and the cross-validation procedure uses cluster sampling and survey weights to form each fold. See Section 1.1 for more details.

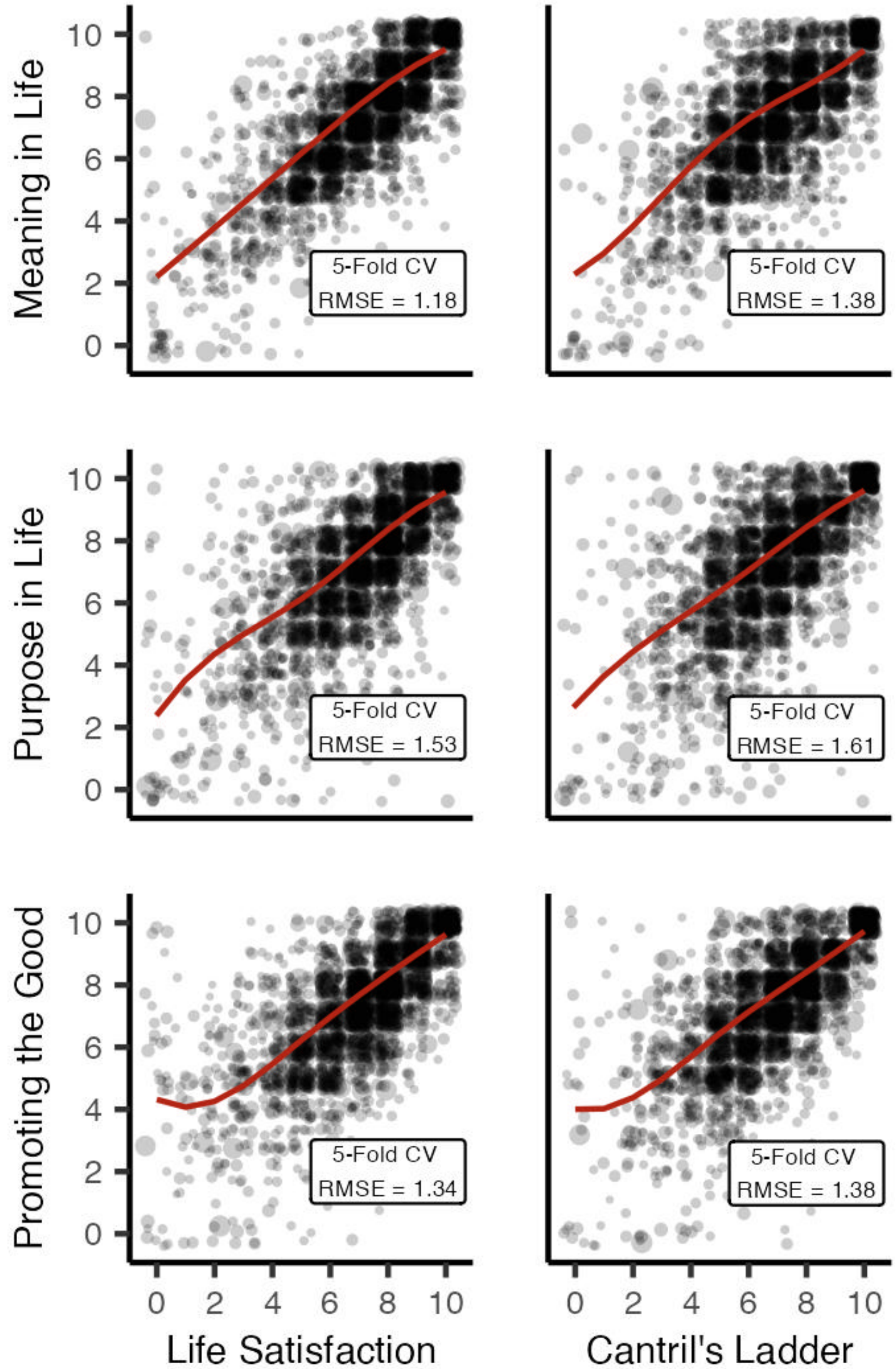

Figure 22: Scatterplots for Hong Kong, Panel 2. Curves fit using a piecewise polynomial regression with B-splines. Regressions incorporate survey weights, and the cross-validation procedure uses cluster sampling and survey weights to form each fold. See Section 1.1 for more details.

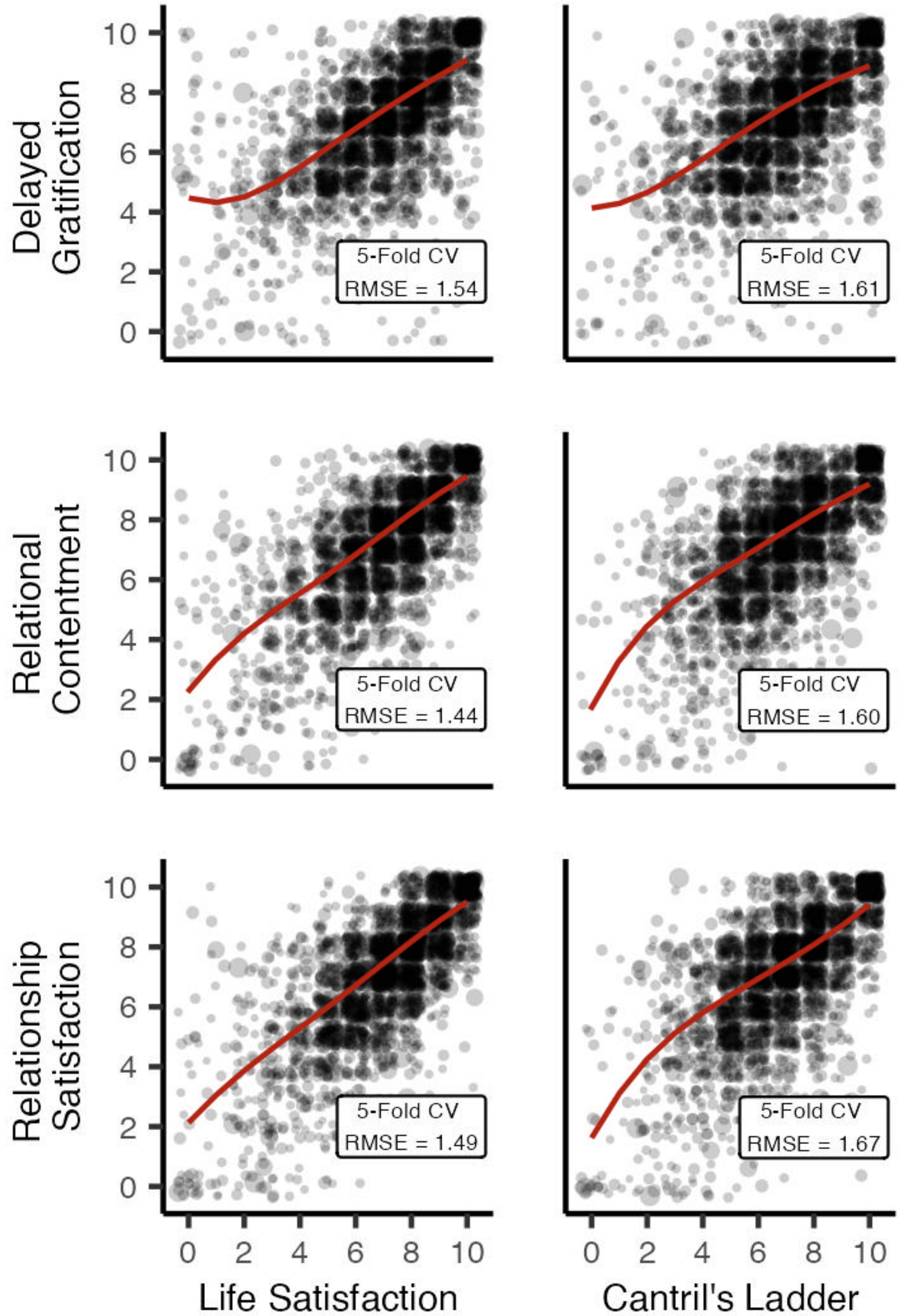

Figure 23: Scatterplots for Hong Kong, Panel 3. Curves fit using a piecewise polynomial regression with B-splines. Regressions incorporate survey weights, and the cross-validation procedure uses cluster sampling and survey weights to form each fold. See Section 1.1 for more details.

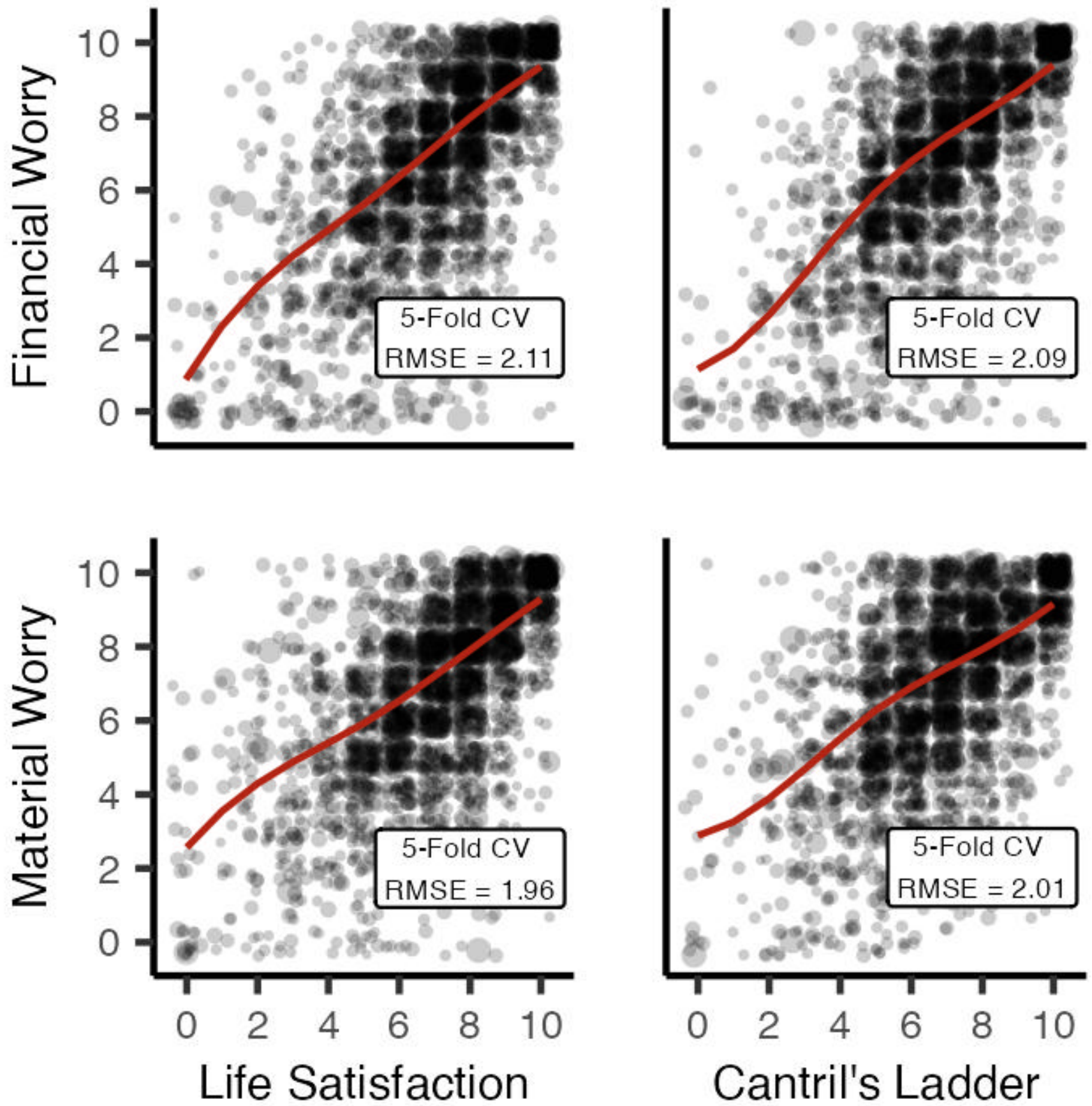

Figure 24: Scatterplots for Hong Kong, Panel 4. Curves fit using a piecewise polynomial regression with B-splines. Regressions incorporate survey weights, and the cross-validation procedure uses cluster sampling and survey weights to form each fold. See Section 1.1 for more details.

Section 9

Scatterplots for India

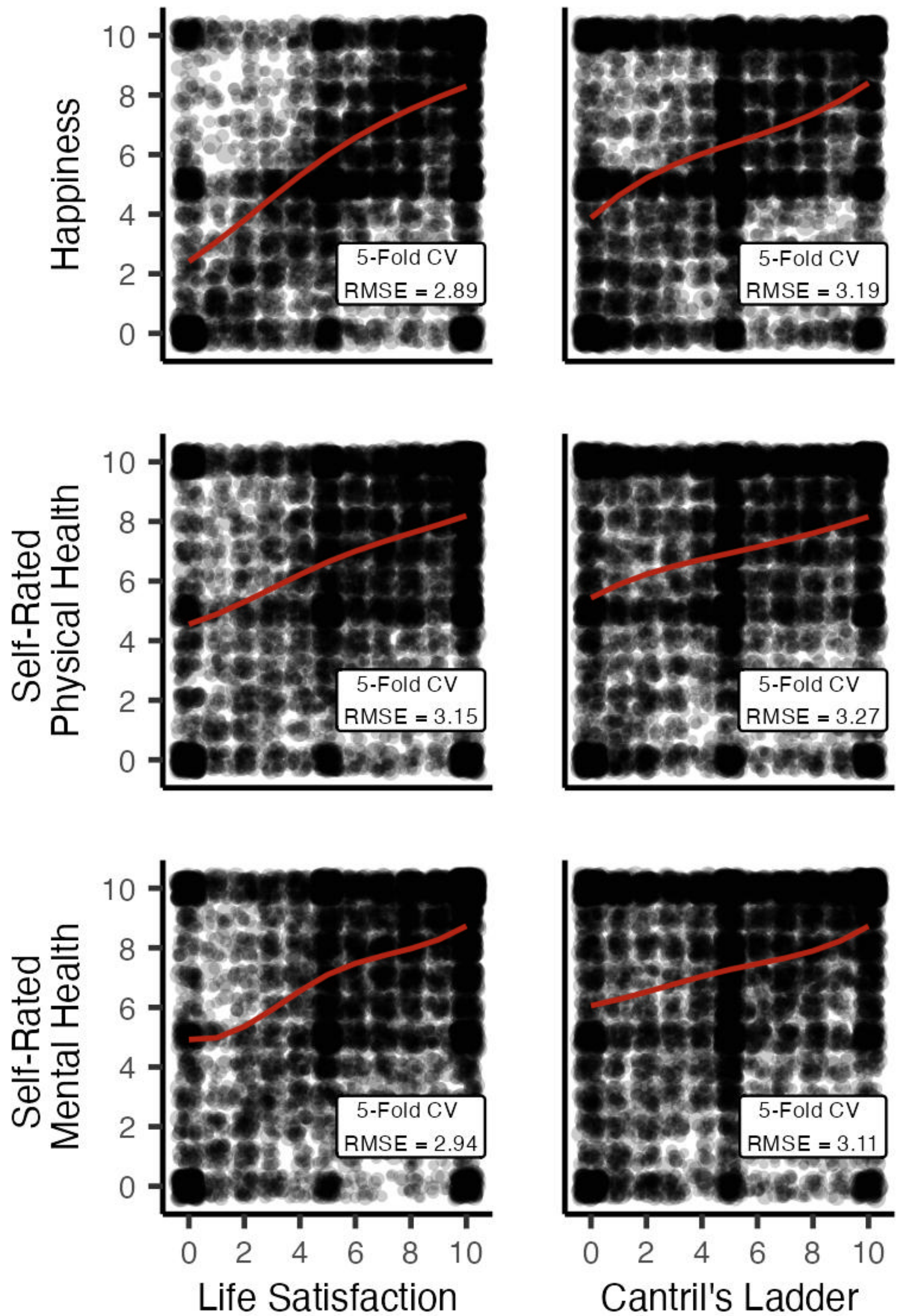

Figure 25: Scatterplots for India, Panel 1. Curves fit using a piecewise polynomial regression with B-splines. Regressions incorporate survey weights, and the cross-validation procedure uses cluster sampling and survey weights to form each fold. See Section 1.1 for more details.

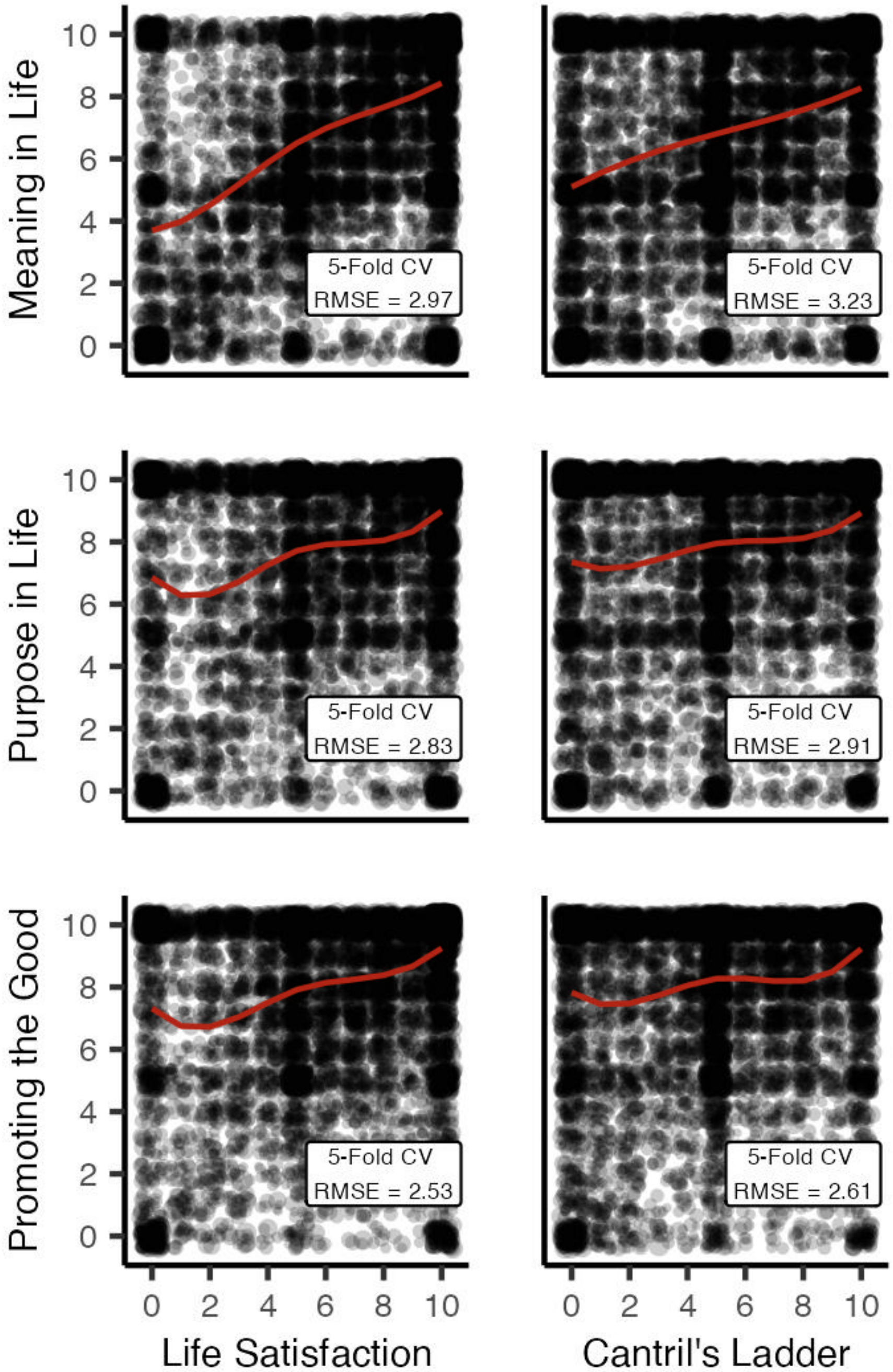

Figure 26: Scatterplots for India, Panel 2. Curves fit using a piecewise polynomial regression with B-splines. Regressions incorporate survey weights, and the cross-validation procedure uses cluster sampling and survey weights to form each fold. See Section 1.1 for more details.

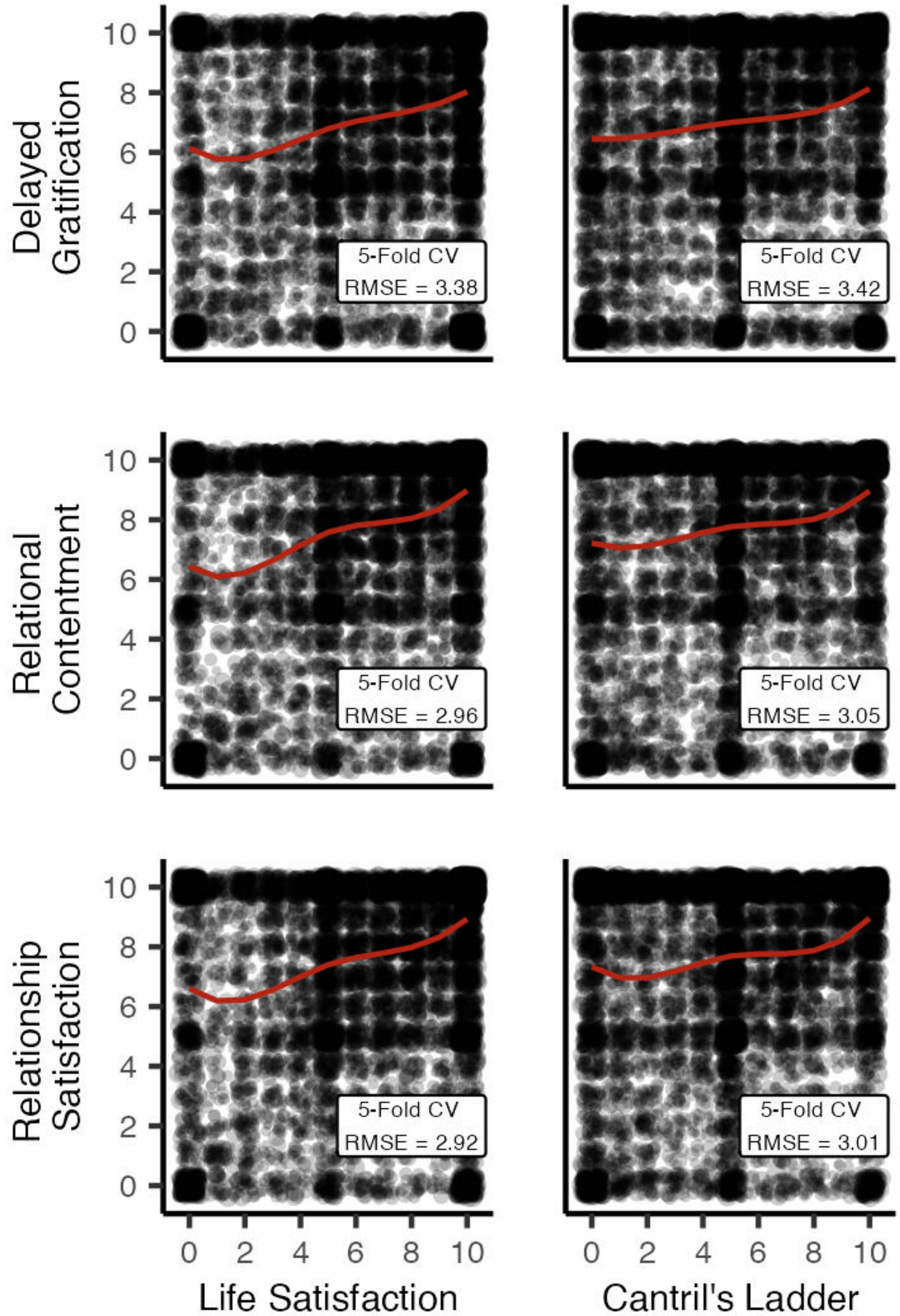

Figure 27: Scatterplots for India, Panel 3. Curves fit using a piecewise polynomial regression with B-splines. Regressions incorporate survey weights, and the cross-validation procedure uses cluster sampling and survey weights to form each fold. See Section 1.1 for more details.

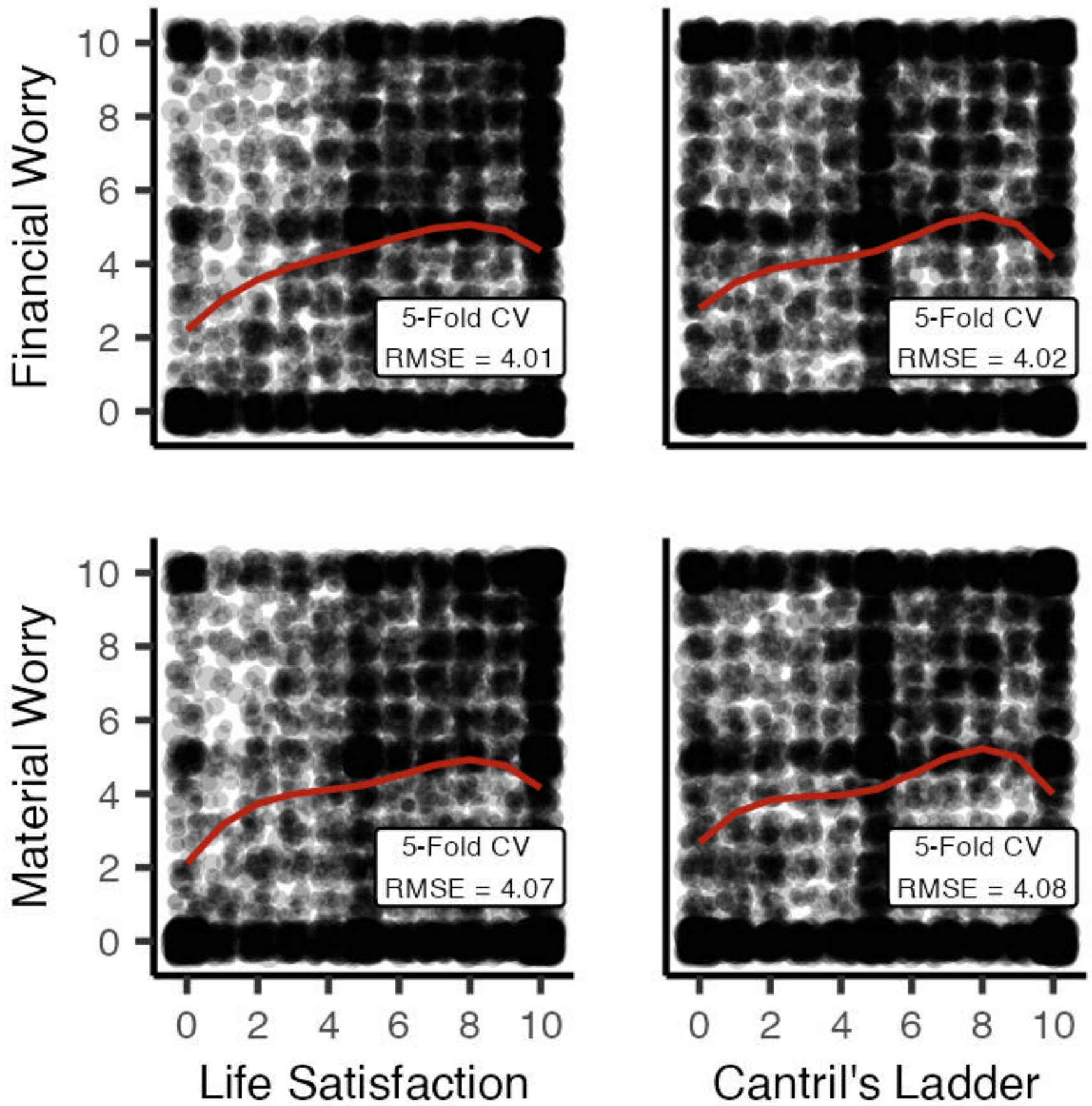

Figure 28: Scatterplots for India, Panel 4. Curves fit using a piecewise polynomial regression with B-splines. Regressions incorporate survey weights, and the cross-validation procedure uses cluster sampling and survey weights to form each fold. See Section 1.1 for more details.

Section 10

Scatterplots for Indonesia

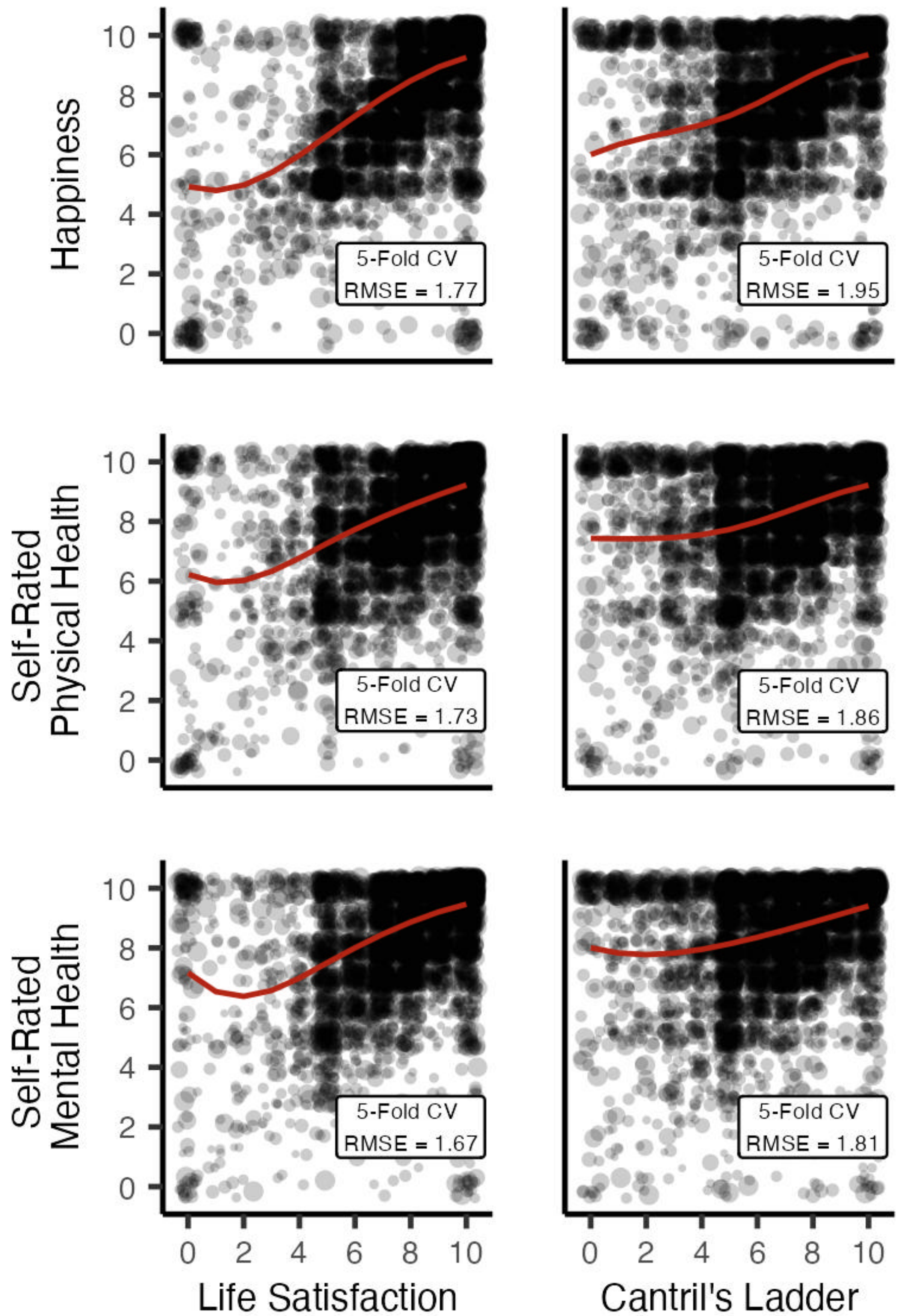

Figure 29: Scatterplots for Indonesia, Panel 1. Curves fit using a piecewise polynomial regression with B-splines. Regressions incorporate survey weights, and the cross-validation procedure uses cluster sampling and survey weights to form each fold. See Section 1.1 for more details.

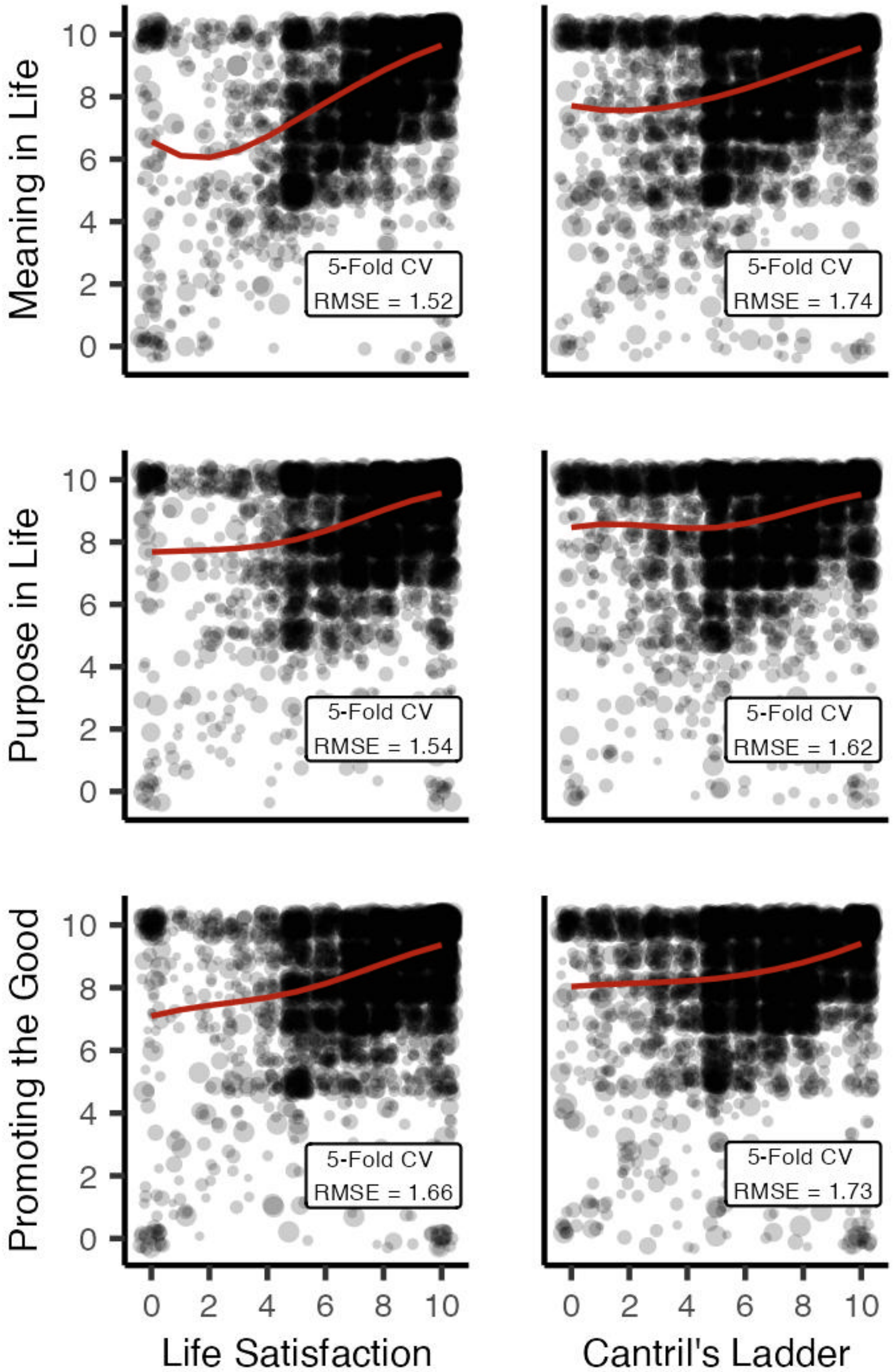

Figure 30: Scatterplots for Indonesia, Panel 2. Curves fit using a piecewise polynomial regression with B-splines. Regressions incorporate survey weights, and the cross-validation procedure uses cluster sampling and survey weights to form each fold. See Section 1.1 for more details.

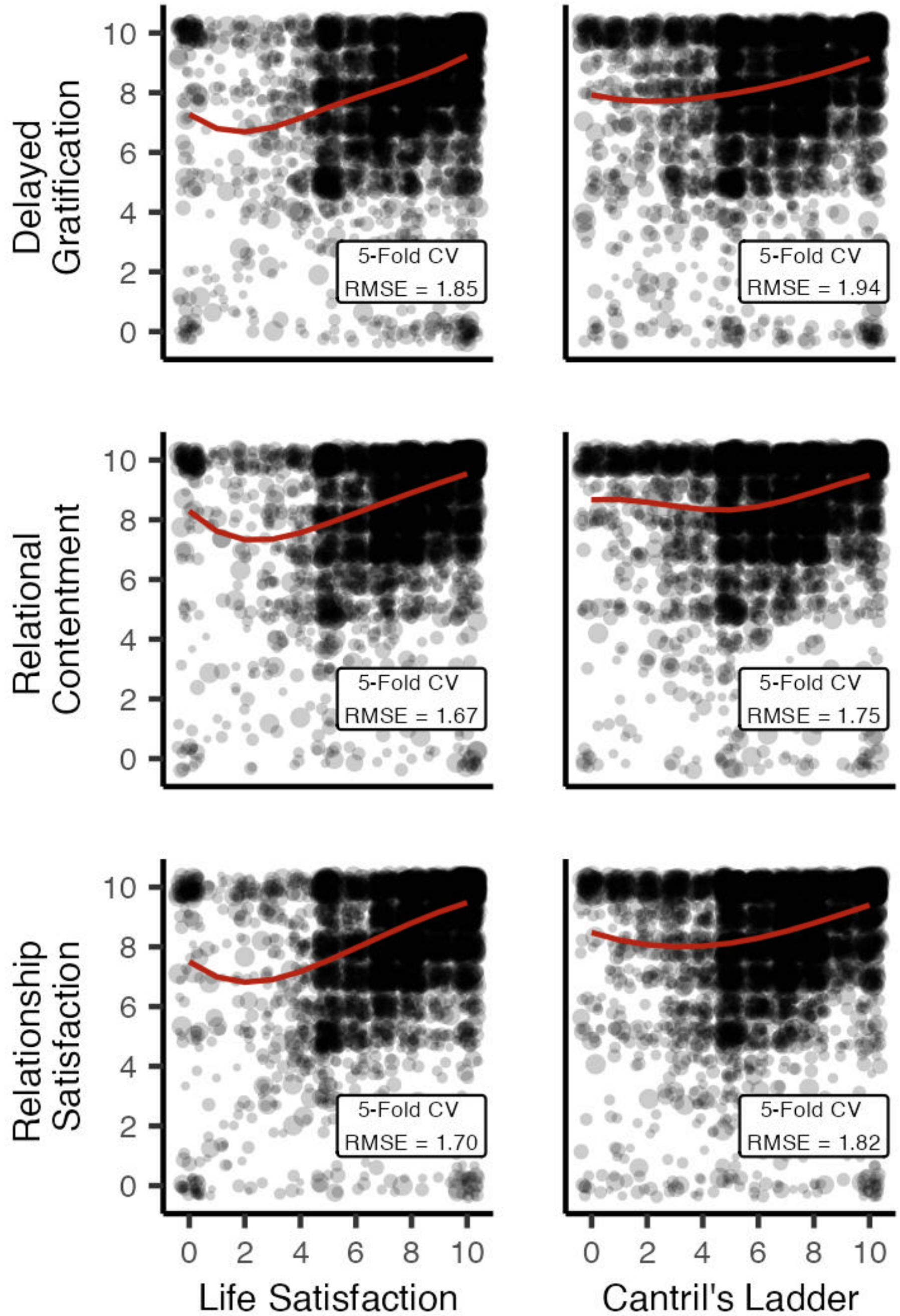

Figure 31: Scatterplots for Indonesia, Panel 3. Curves fit using a piecewise polynomial regression with B-splines. Regressions incorporate survey weights, and the cross-validation procedure uses cluster sampling and survey weights to form each fold. See Section 1.1 for more details.

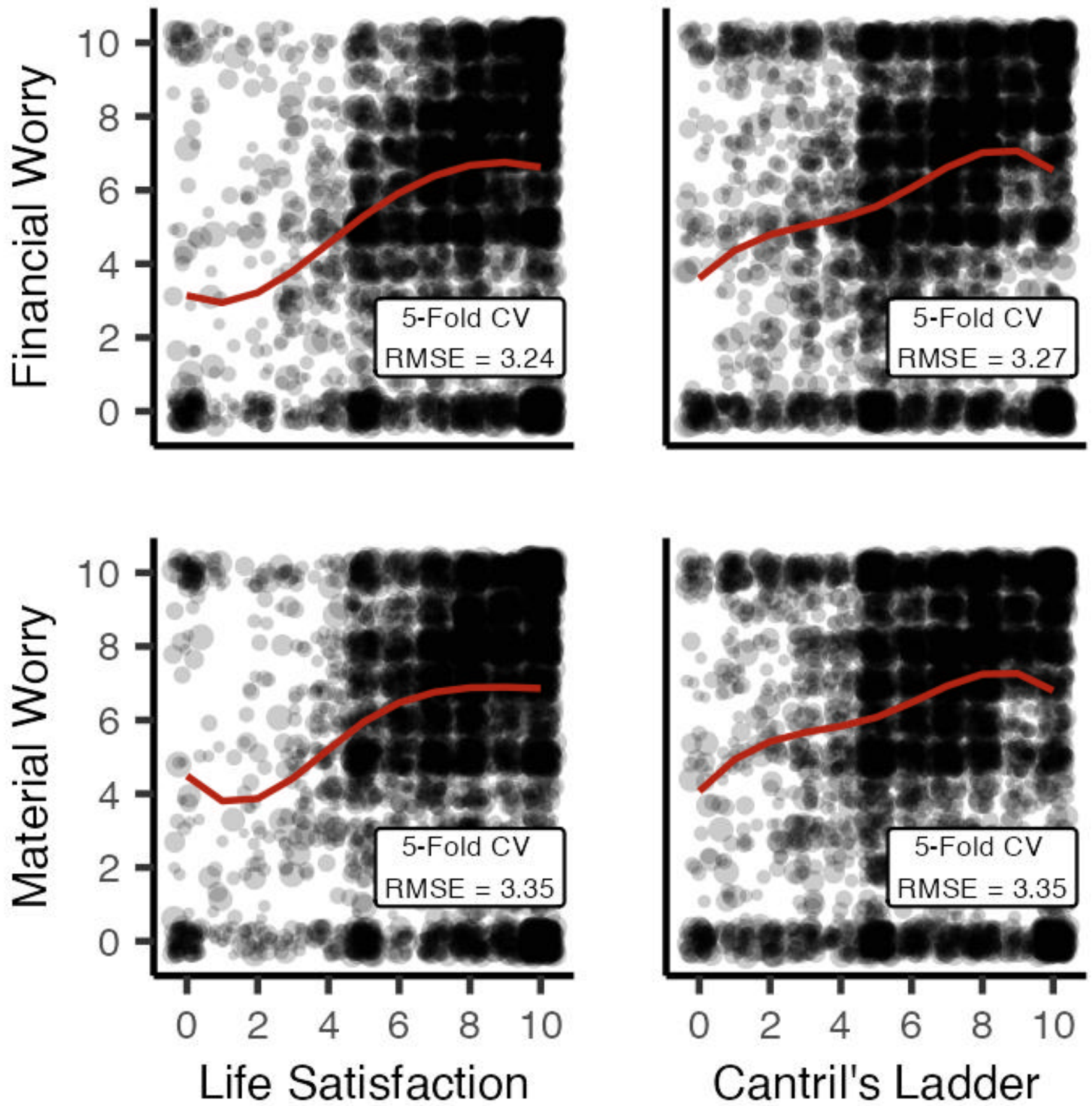

Figure 32: Scatterplots for Indonesia, Panel 4. Curves fit using a piecewise polynomial regression with B-splines. Regressions incorporate survey weights, and the cross-validation procedure uses cluster sampling and survey weights to form each fold. See Section 1.1 for more details.

Section 11

Scatterplots for Israel

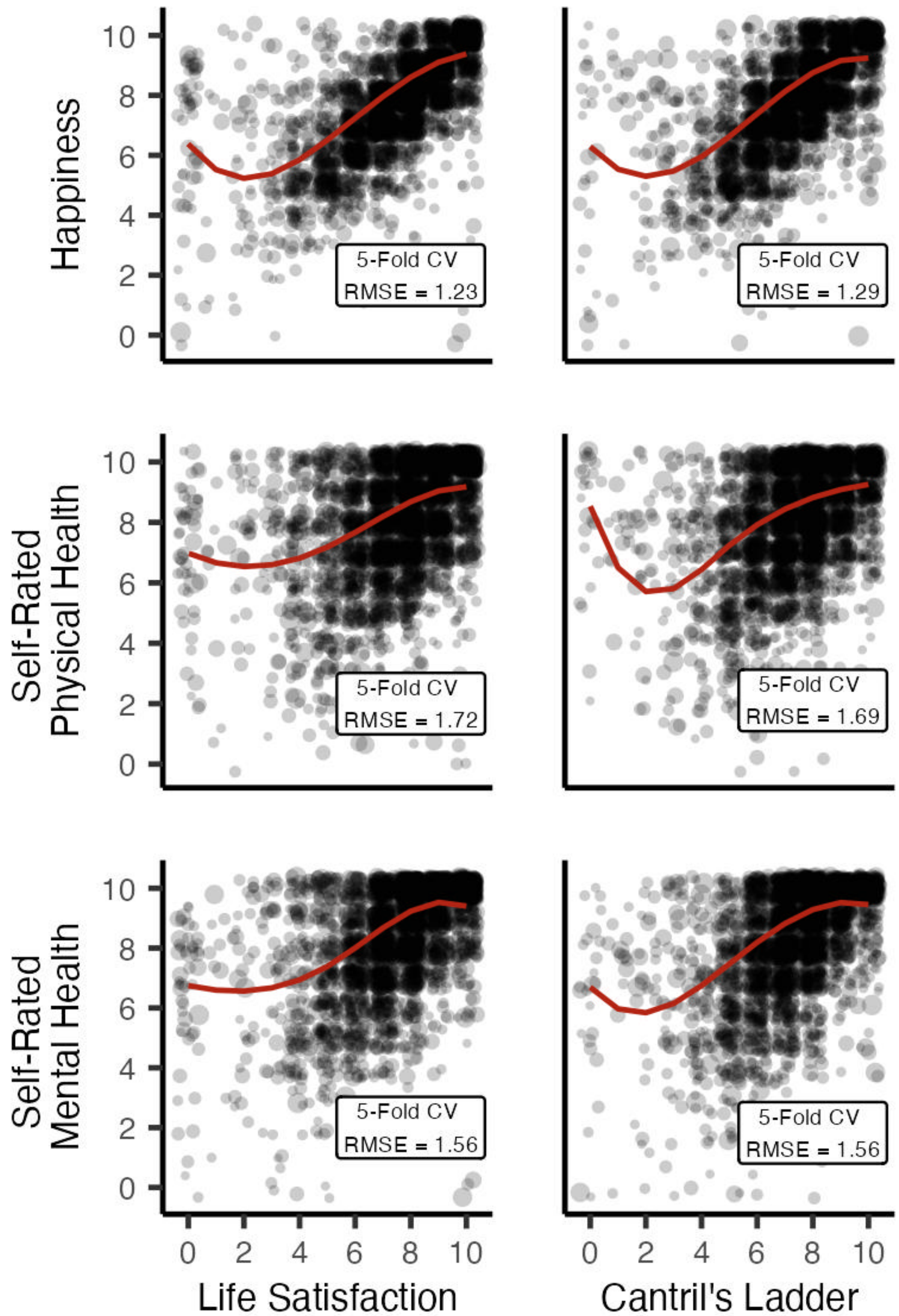

Figure 33: Scatterplots for Israel, Panel 1. Curves fit using a piecewise polynomial regression with B-splines. Regressions incorporate survey weights, and the cross-validation procedure uses cluster sampling and survey weights to form each fold. See Section 1.1 for more details.

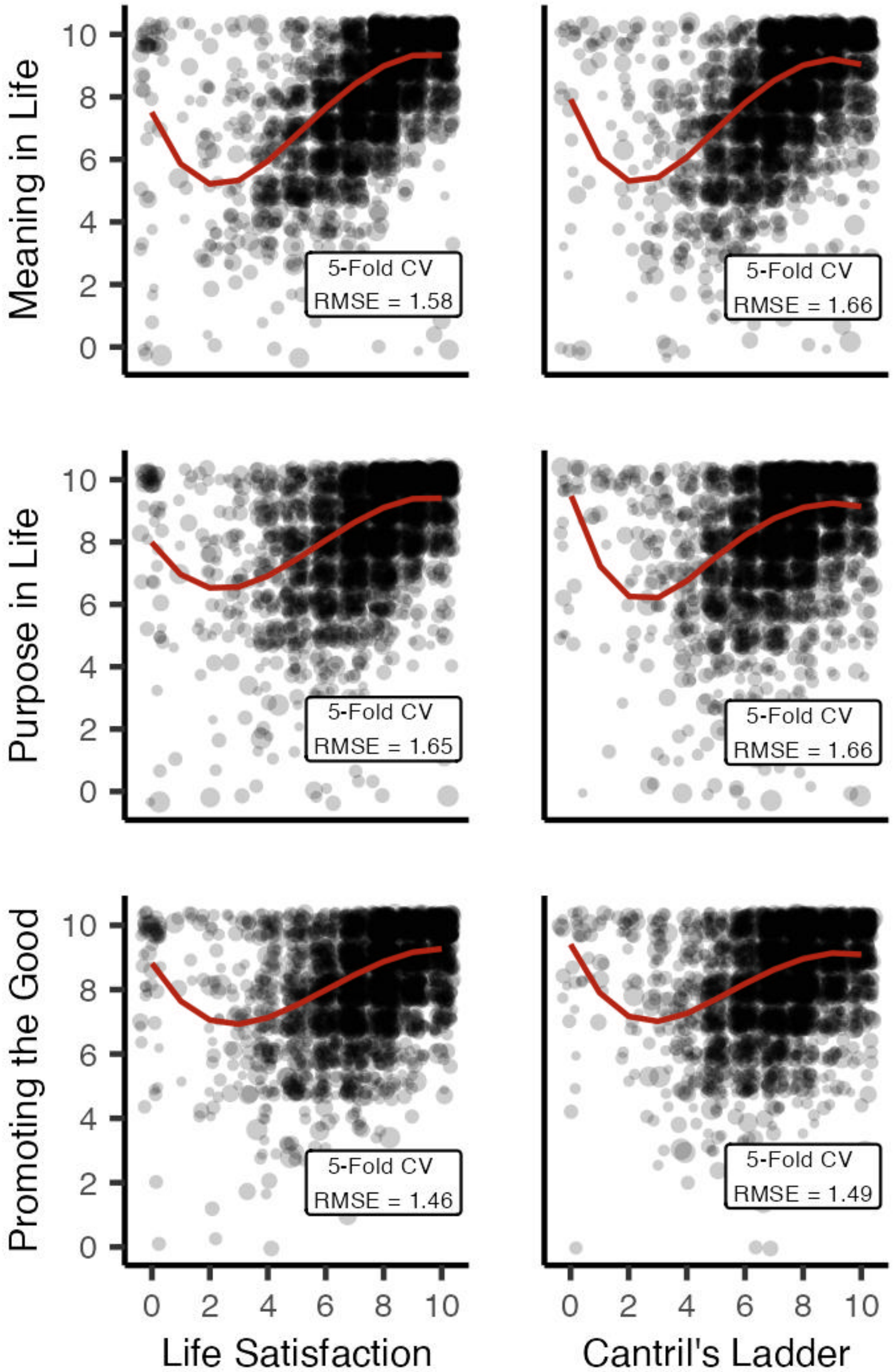

Figure 34: Scatterplots for Israel, Panel 2. Curves fit using a piecewise polynomial regression with B-splines. Regressions incorporate survey weights, and the cross-validation procedure uses cluster sampling and survey weights to form each fold. See Section 1.1 for more details.

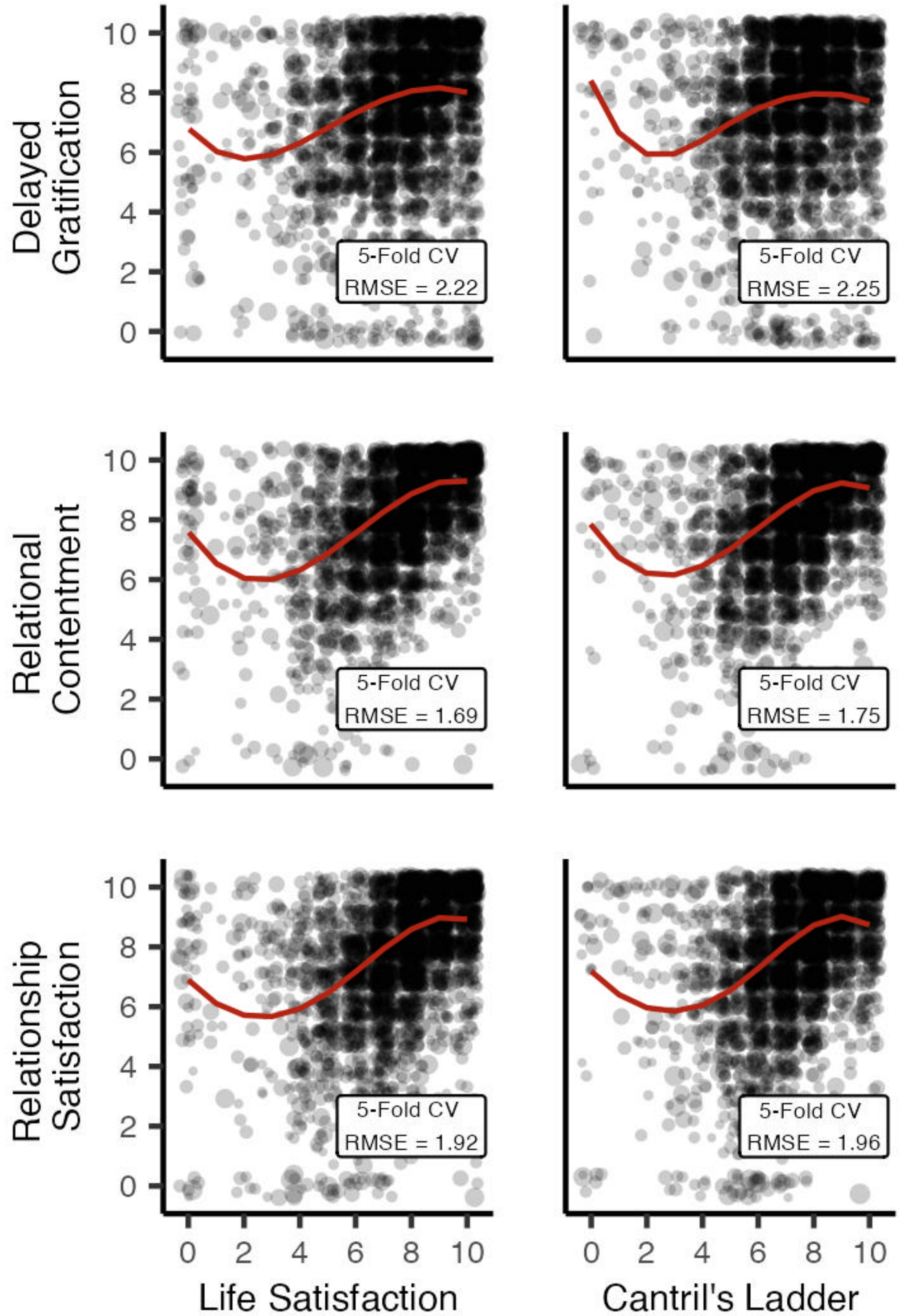

Figure 35: Scatterplots for Israel, Panel 3. Curves fit using a piecewise polynomial regression with B-splines. Regressions incorporate survey weights, and the cross-validation procedure uses cluster sampling and survey weights to form each fold. See Section 1.1 for more details.

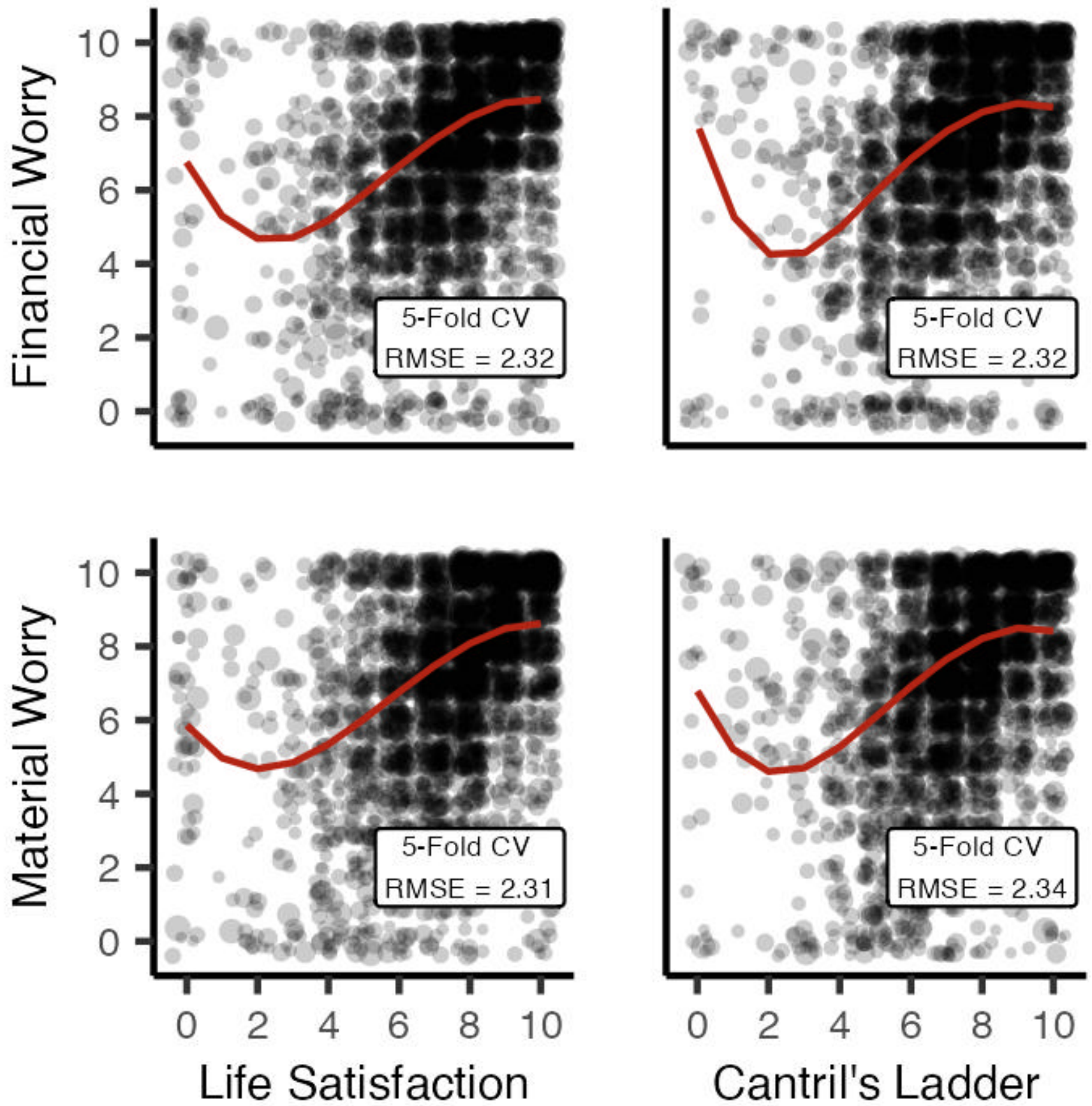

Figure 36: Scatterplots for Israel, Panel 4. Curves fit using a piecewise polynomial regression with B-splines. Regressions incorporate survey weights, and the cross-validation procedure uses cluster sampling and survey weights to form each fold. See Section 1.1 for more details.

Section 12

Scatterplots for Japan

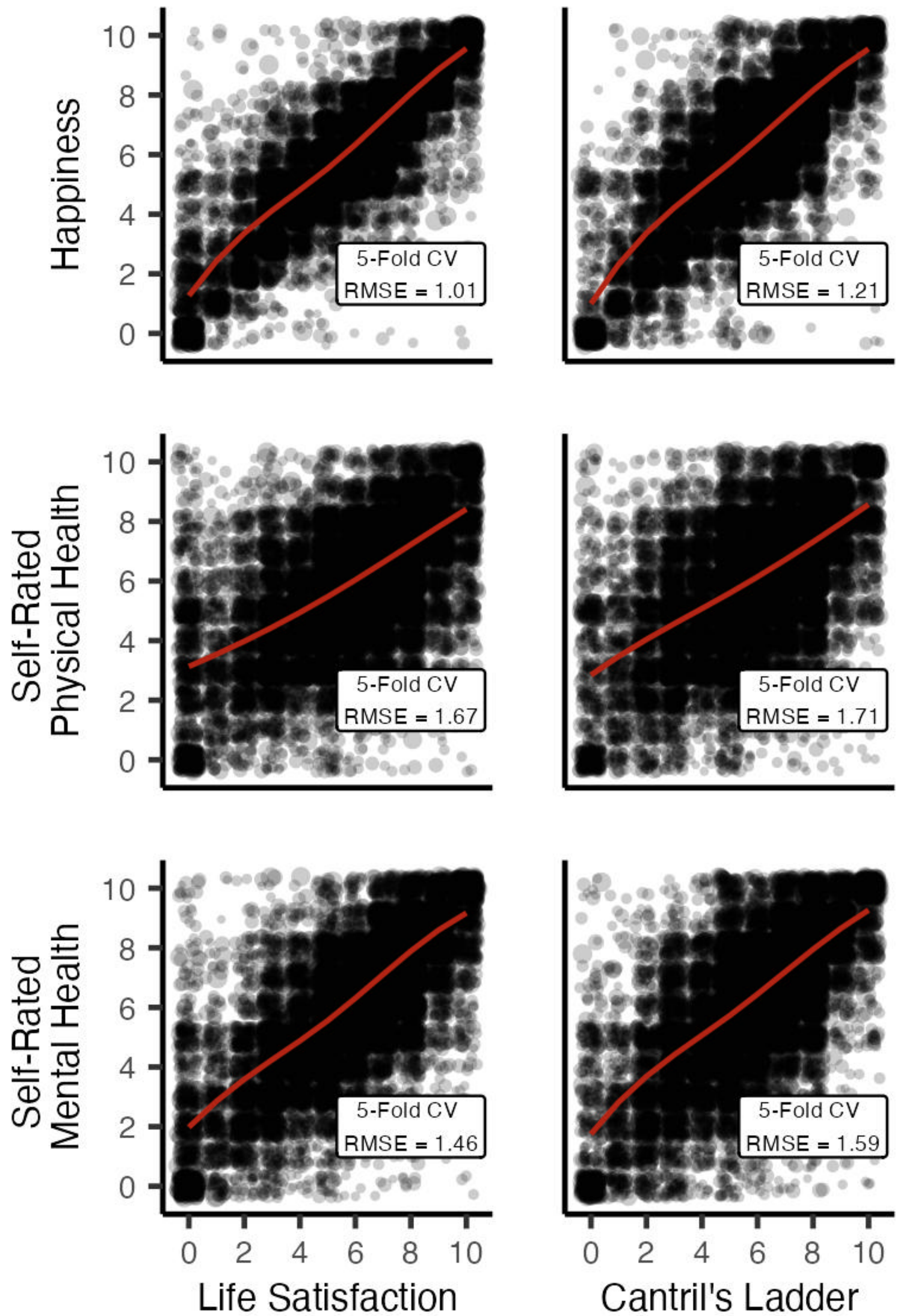

Figure 37: Scatterplots for Japan, Panel 1. Curves fit using a piecewise polynomial regression with B-splines. Regressions incorporate survey weights, and the cross-validation procedure uses cluster sampling and survey weights to form each fold. See Section 1.1 for more details.

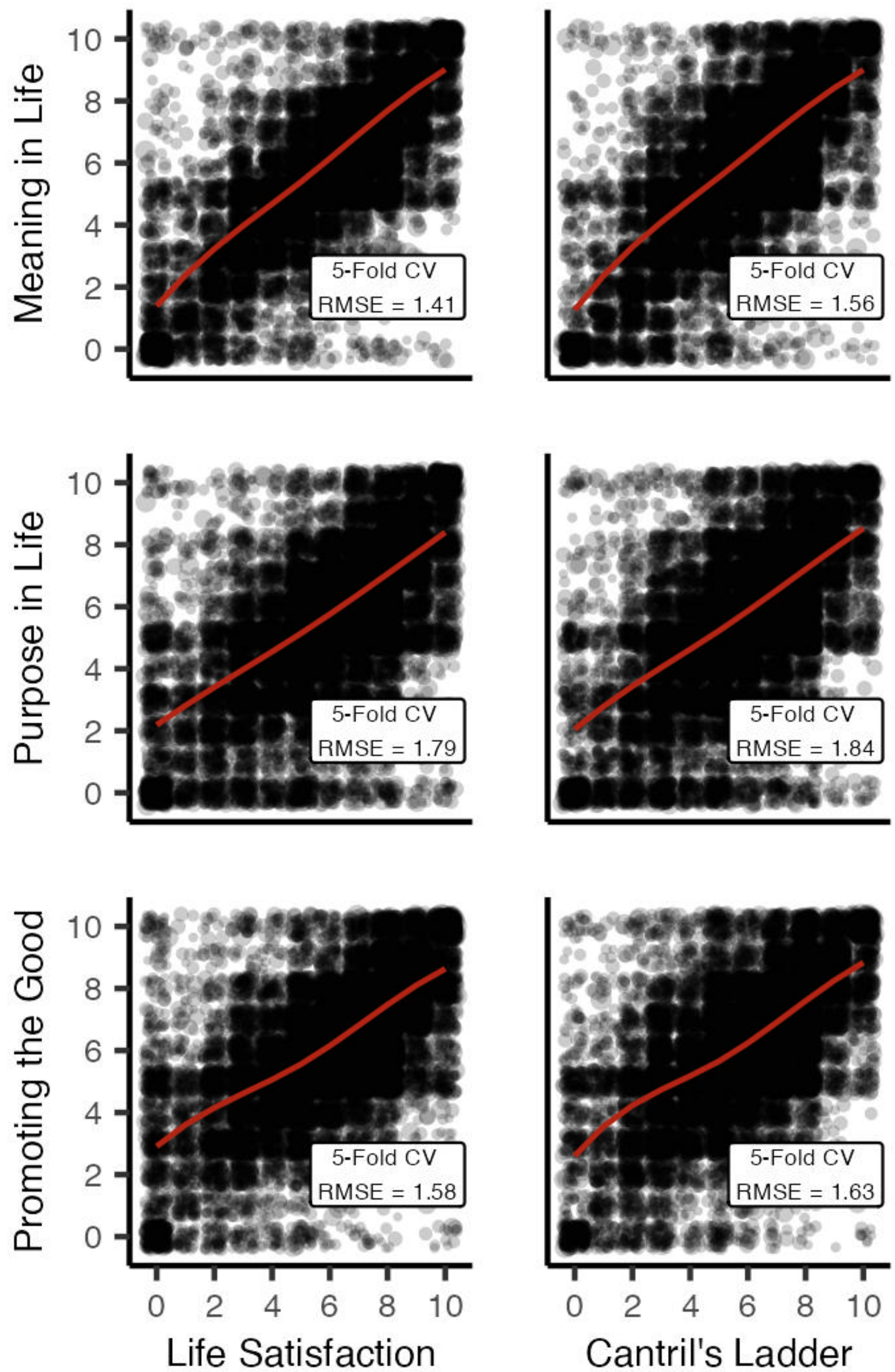

Figure 38: Scatterplots for Japan, Panel 2. Curves fit using a piecewise polynomial regression with B-splines. Regressions incorporate survey weights, and the cross-validation procedure uses cluster sampling and survey weights to form each fold. See Section 1.1 for more details.

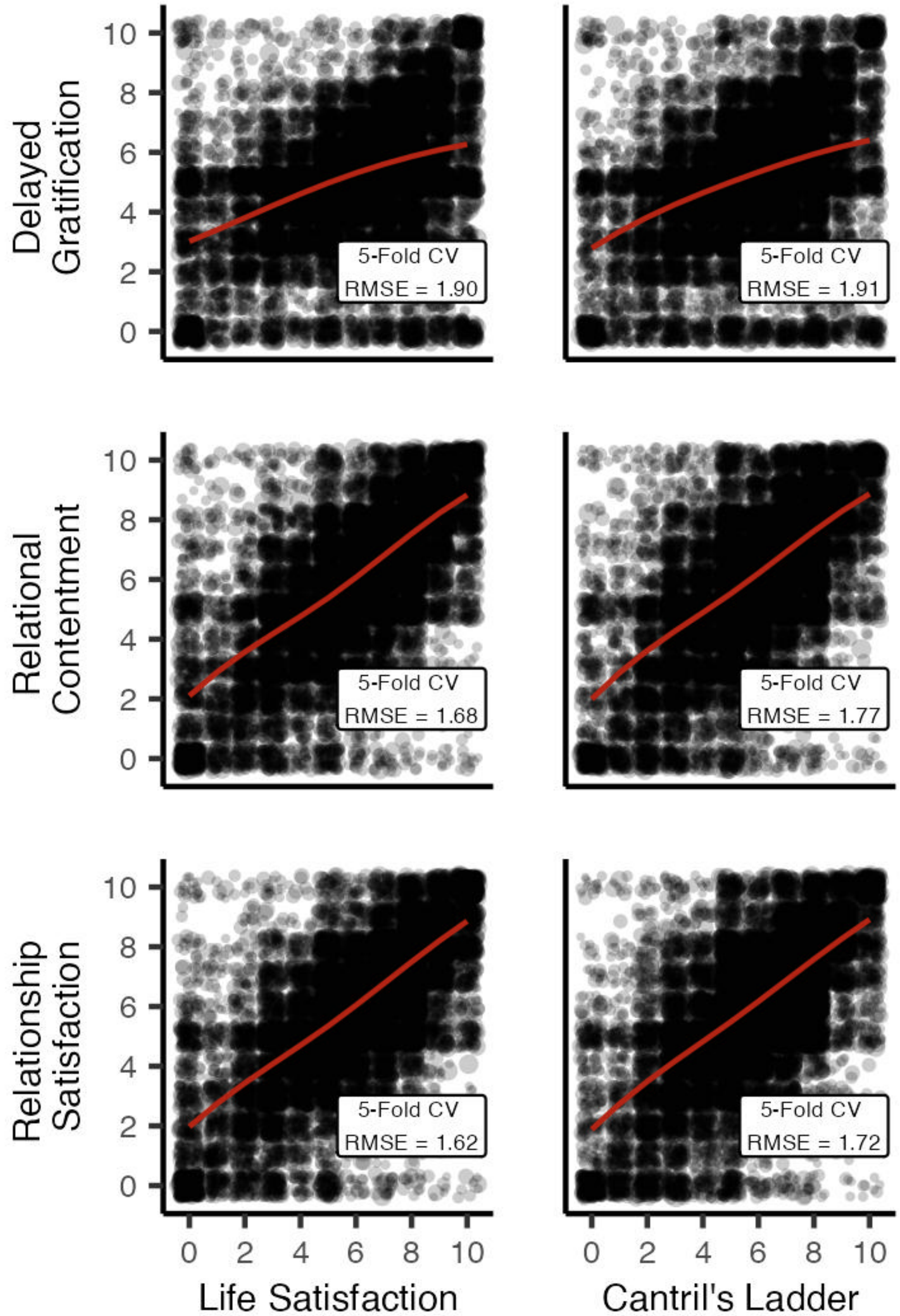

Figure 39: Scatterplots for Japan, Panel 3. Curves fit using a piecewise polynomial regression with B-splines. Regressions incorporate survey weights, and the cross-validation procedure uses cluster sampling and survey weights to form each fold. See Section 1.1 for more details.

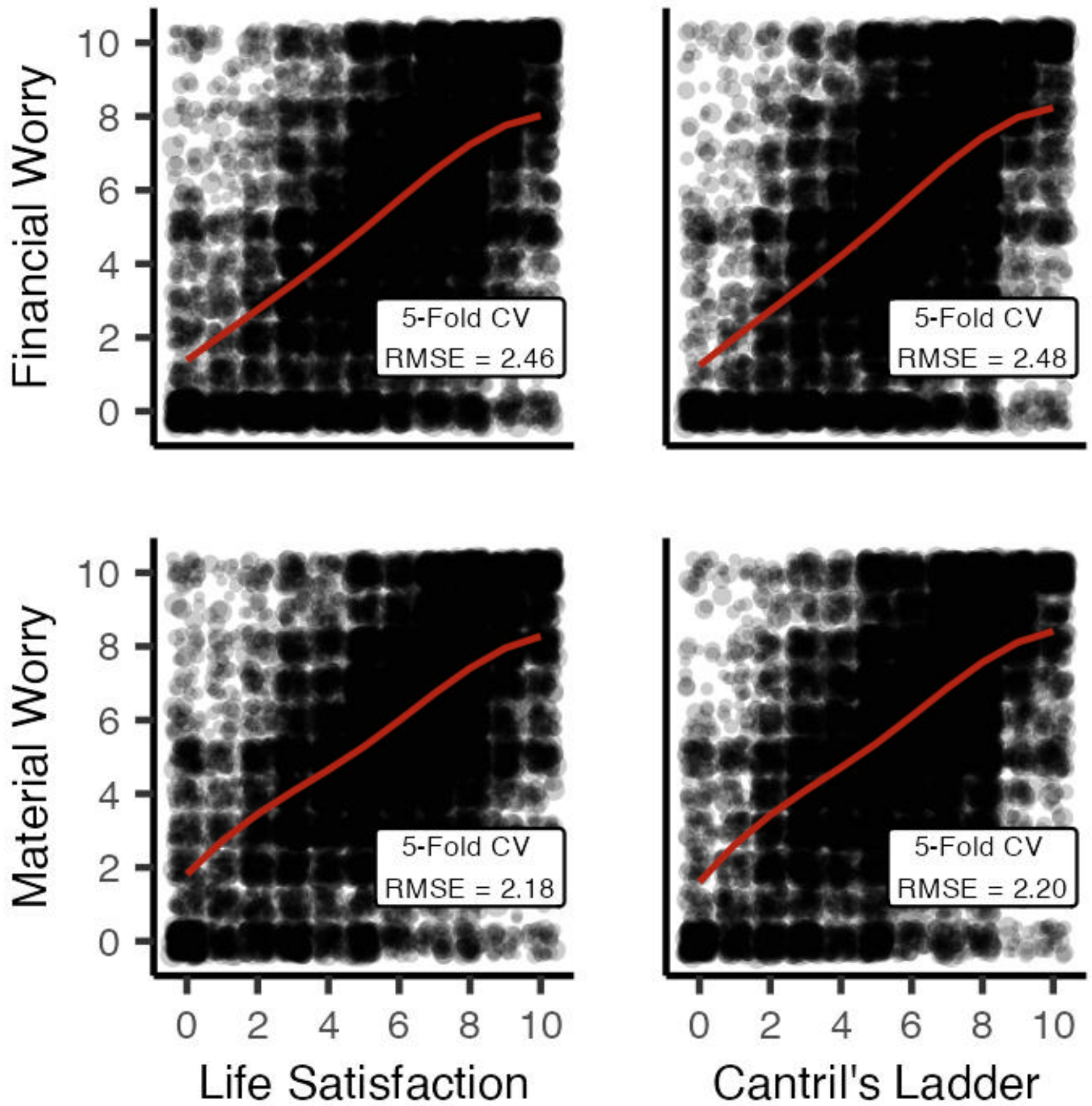

Figure 40: Scatterplots for Japan, Panel 4. Curves fit using a piecewise polynomial regression with B-splines. Regressions incorporate survey weights, and the cross-validation procedure uses cluster sampling and survey weights to form each fold. See Section 1.1 for more details.

Section 13

Scatterplots for Kenya

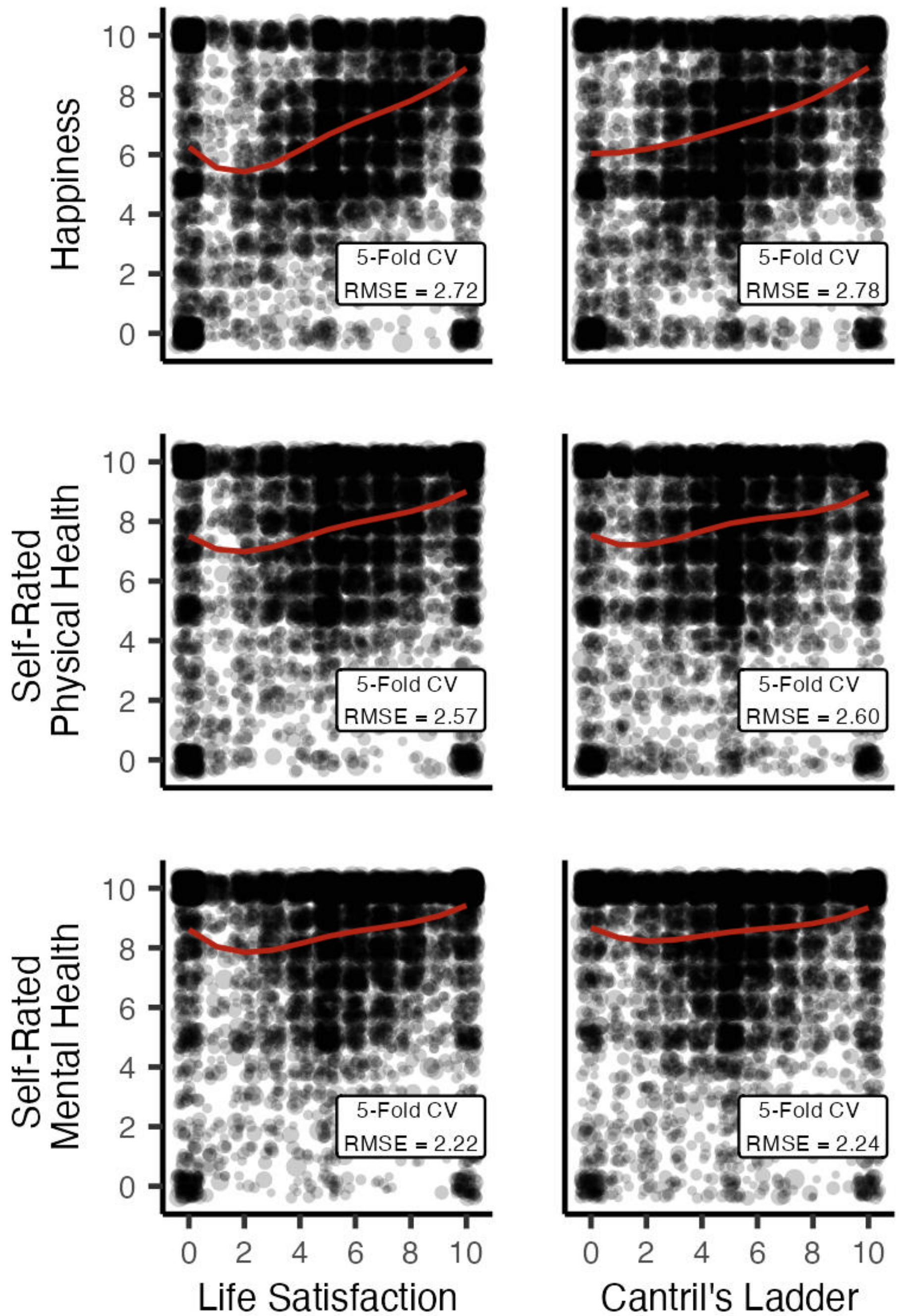

Figure 41: Scatterplots for Kenya, Panel 1. Curves fit using a piecewise polynomial regression with B-splines. Regressions incorporate survey weights, and the cross-validation procedure uses cluster sampling and survey weights to form each fold. See Section 1.1 for more details.

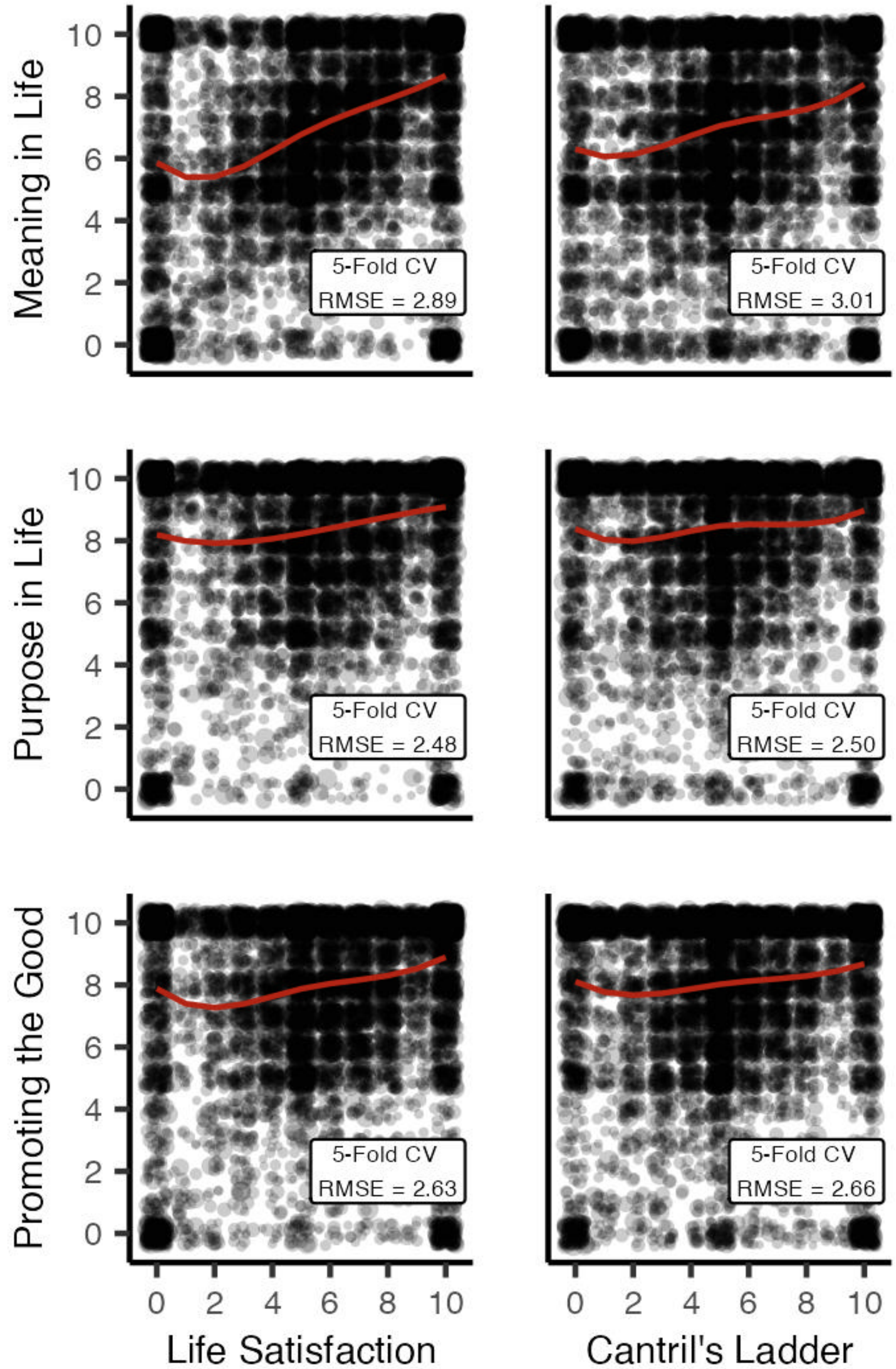

Figure 42: Scatterplots for Kenya, Panel 2. Curves fit using a piecewise polynomial regression with B-splines. Regressions incorporate survey weights, and the cross-validation procedure uses cluster sampling and survey weights to form each fold. See Section 1.1 for more details.

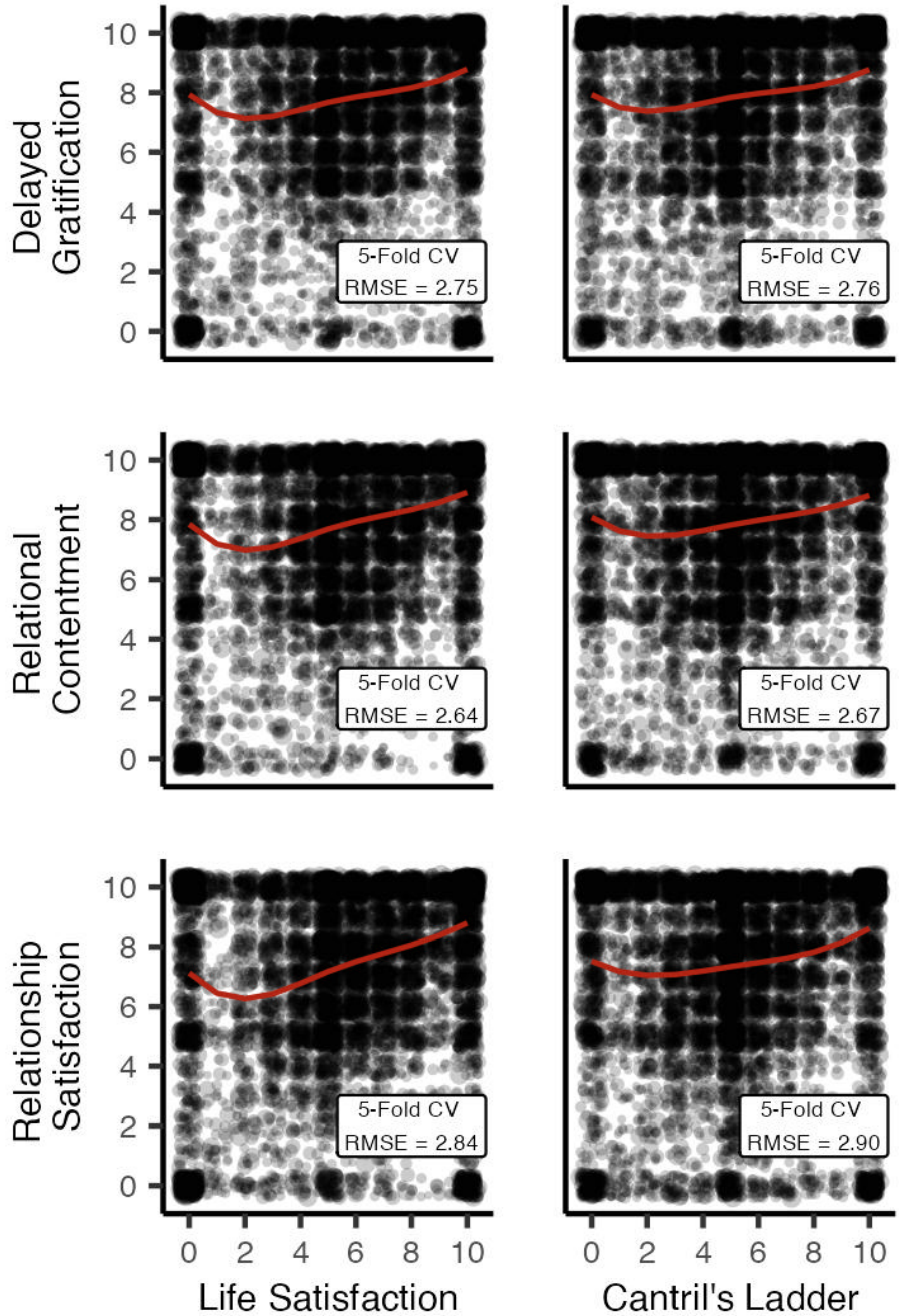

Figure 43: Scatterplots for Kenya, Panel 3. Curves fit using a piecewise polynomial regression with B-splines. Regressions incorporate survey weights, and the cross-validation procedure uses cluster sampling and survey weights to form each fold. See Section 1.1 for more details.

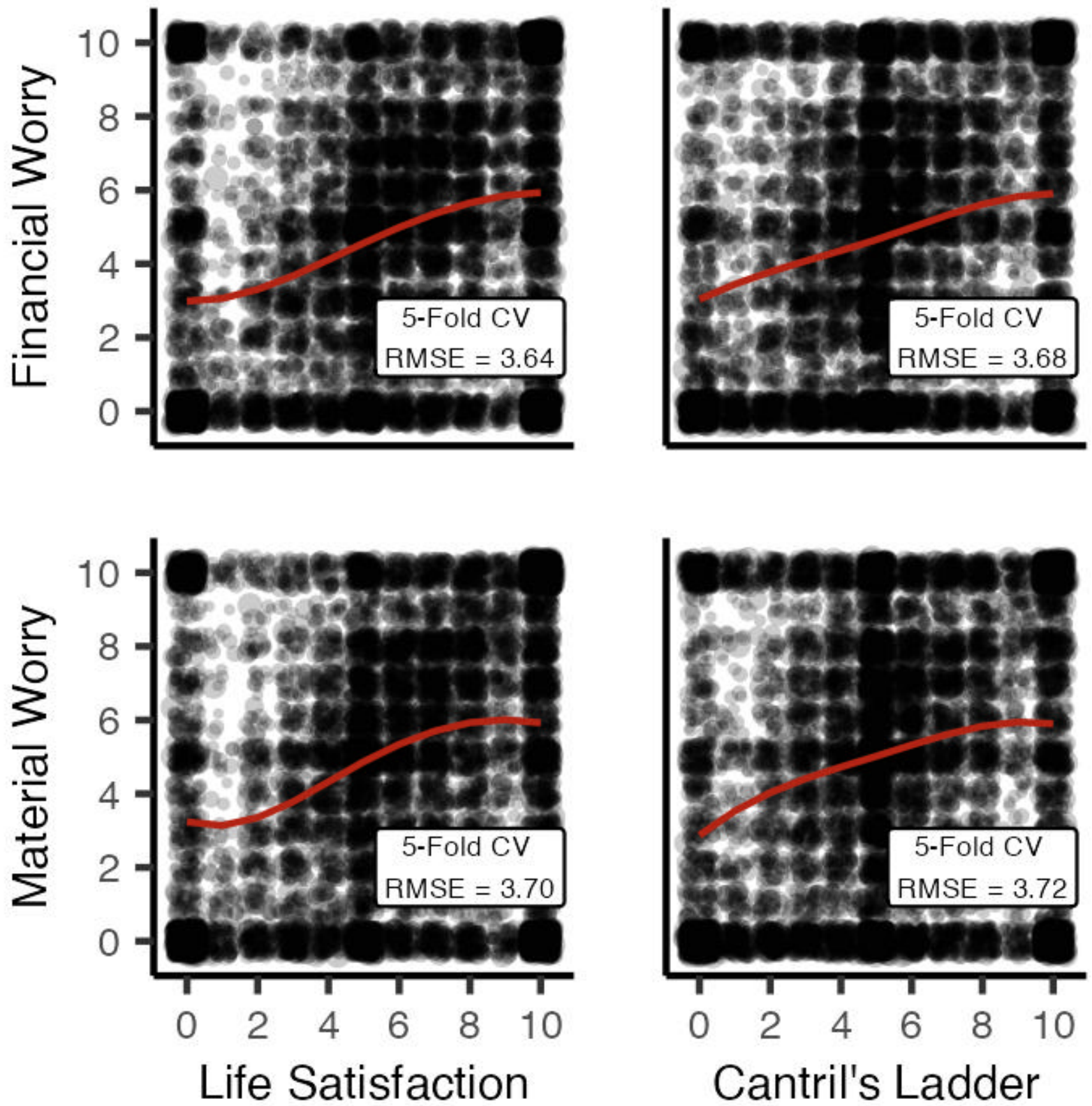

Figure 44: Scatterplots for Kenya, Panel 4. Curves fit using a piecewise polynomial regression with B-splines. Regressions incorporate survey weights, and the cross-validation procedure uses cluster sampling and survey weights to form each fold. See Section 1.1 for more details.

Section 14

Scatterplots for Mexico

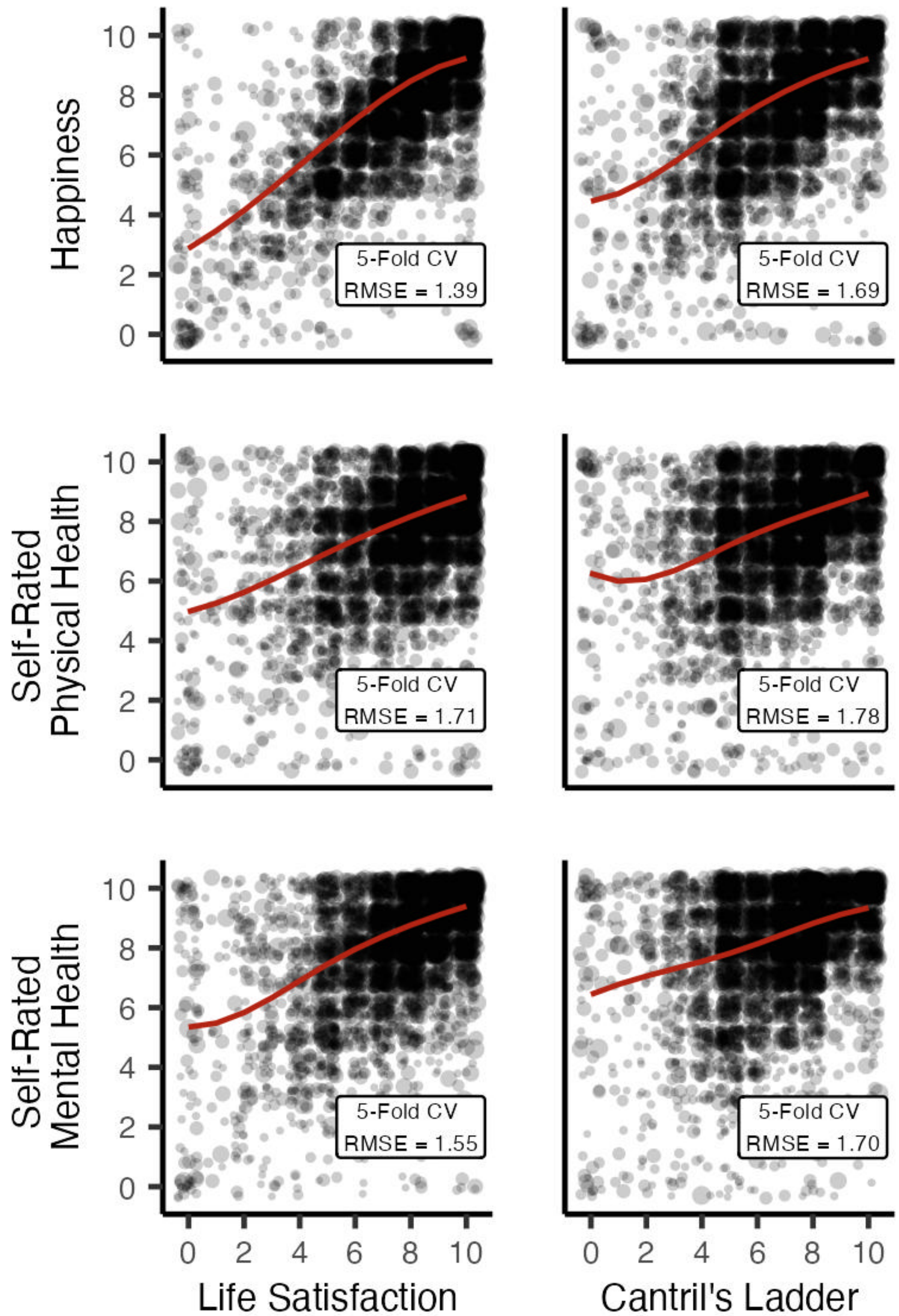

Figure 45: Scatterplots for Mexico, Panel 1. Curves fit using a piecewise polynomial regression with B-splines. Regressions incorporate survey weights, and the cross-validation procedure uses cluster sampling and survey weights to form each fold. See Section 1.1 for more details.

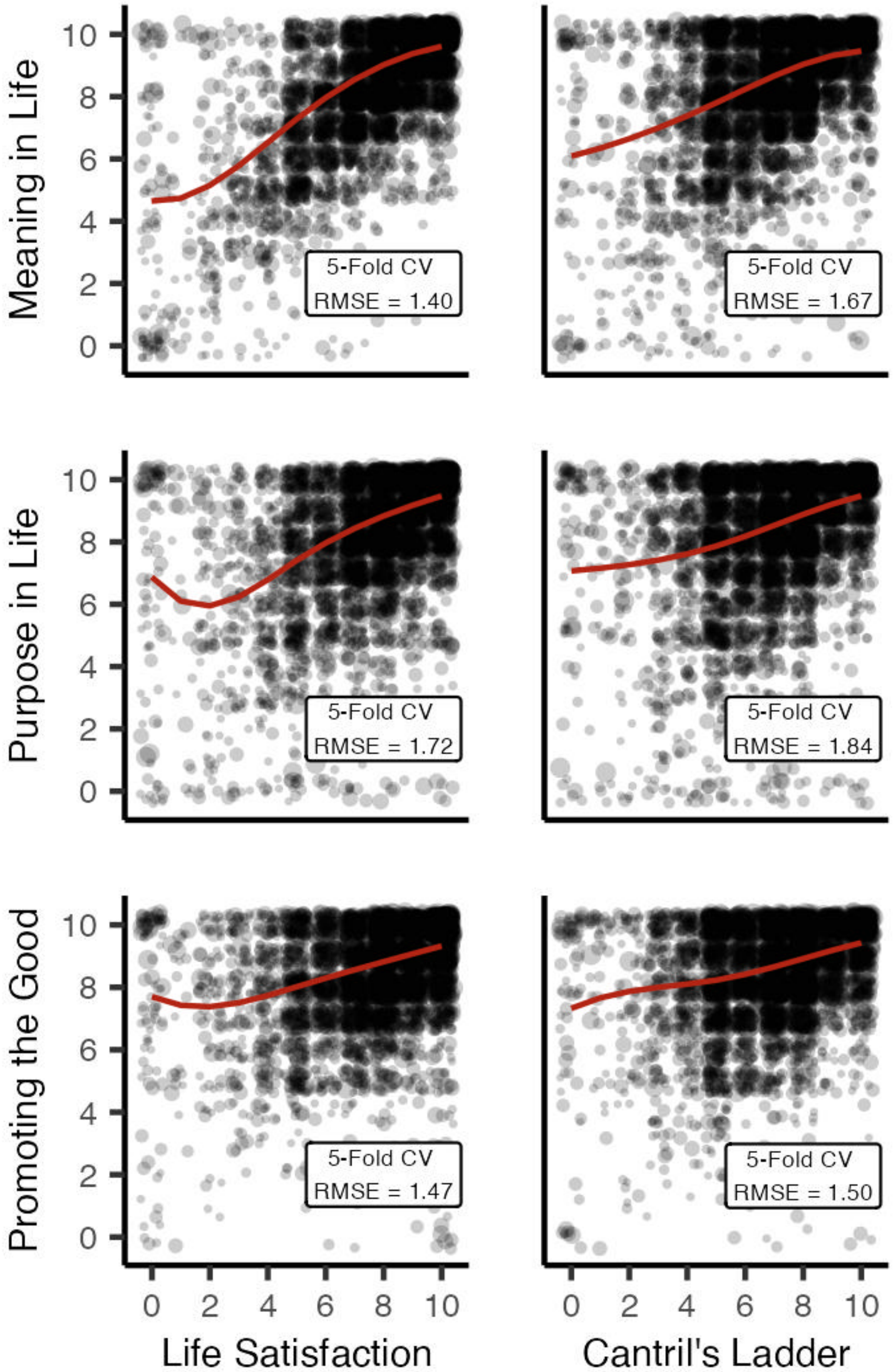

Figure 46: Scatterplots for Mexico, Panel 2. Curves fit using a piecewise polynomial regression with B-splines. Regressions incorporate survey weights, and the cross-validation procedure uses cluster sampling and survey weights to form each fold. See Section 1.1 for more details.

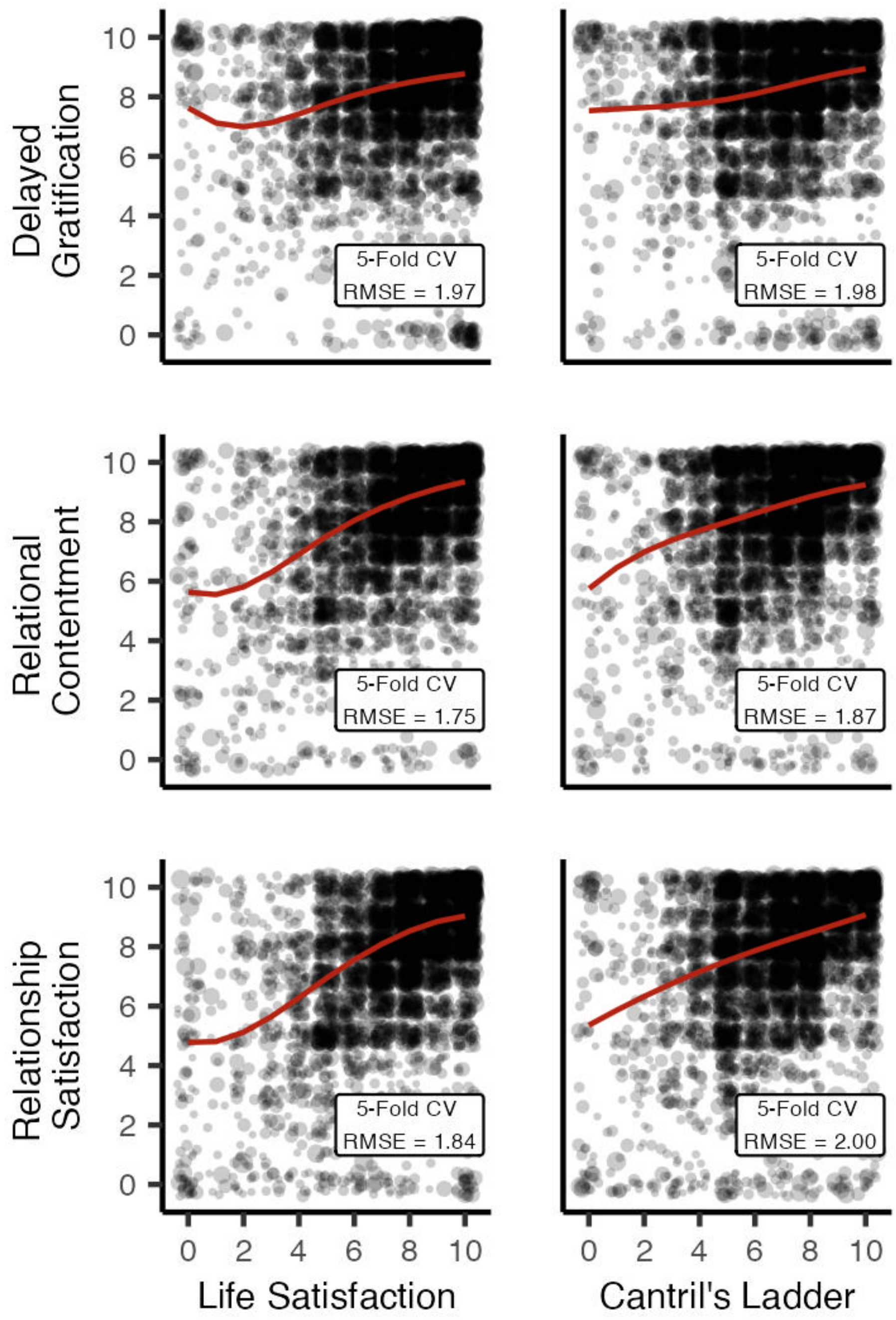

Figure 47: Scatterplots for Mexico, Panel 3. Curves fit using a piecewise polynomial regression with B-splines. Regressions incorporate survey weights, and the cross-validation procedure uses cluster sampling and survey weights to form each fold. See Section 1.1 for more details.

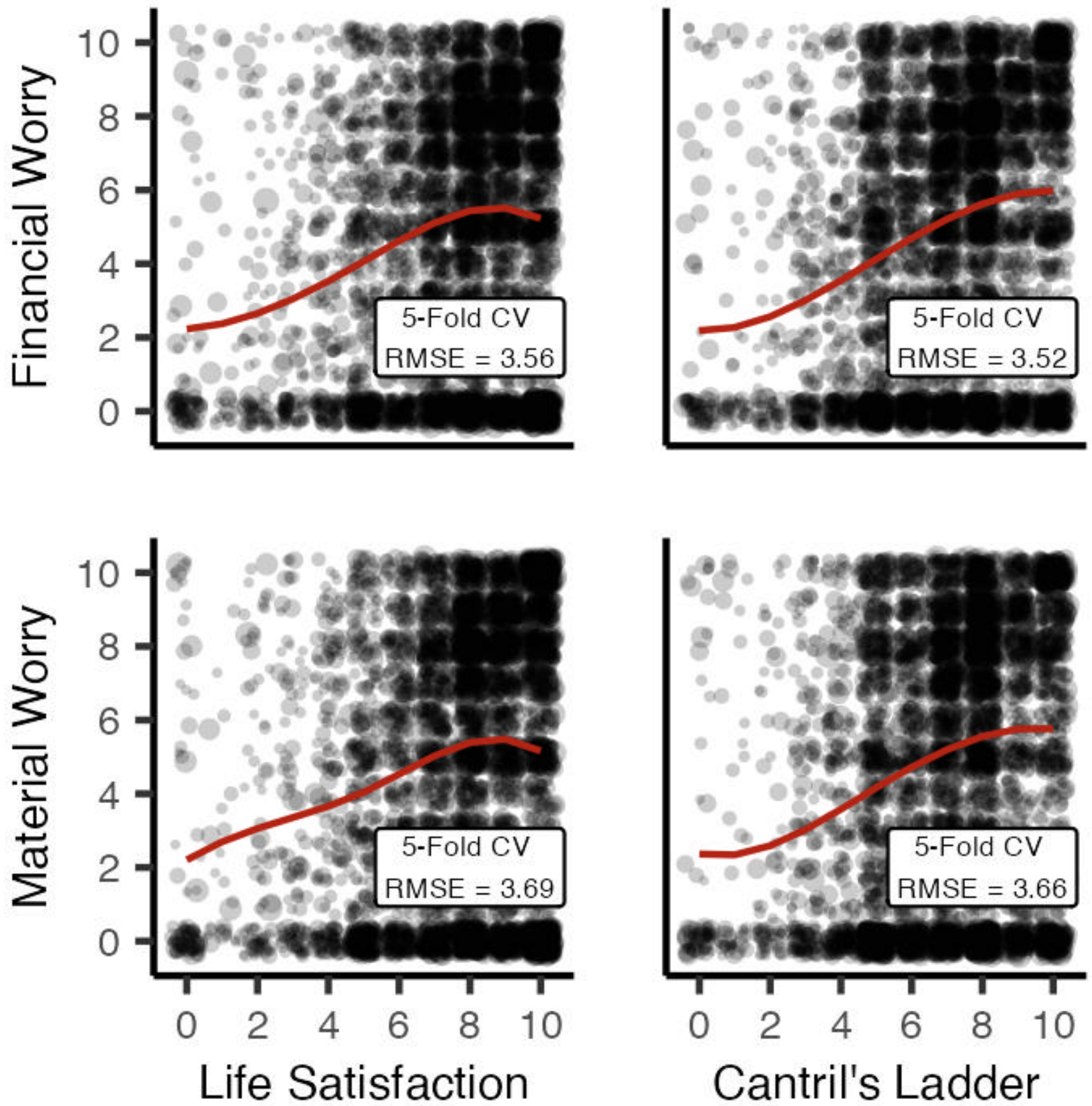

Figure 48: Scatterplots for Mexico, Panel 4. Curves fit using a piecewise polynomial regression with B-splines. Regressions incorporate survey weights, and the cross-validation procedure uses cluster sampling and survey weights to form each fold. See Section 1.1 for more details.

Section 15

Scatterplots for Nigeria

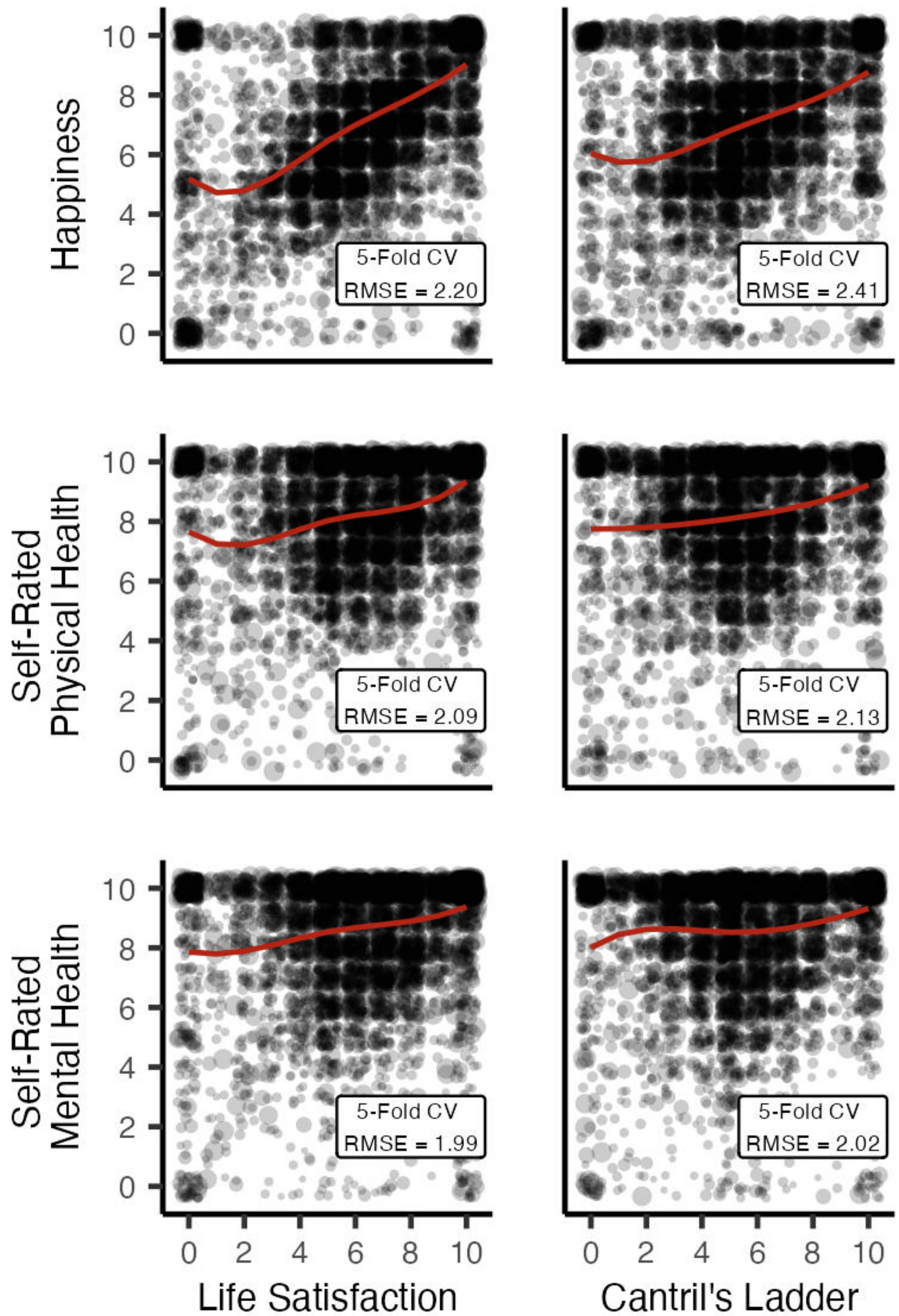

Figure 49: Scatterplots for Nigeria, Panel 1. Curves fit using a piecewise polynomial regression with B-splines. Regressions incorporate survey weights, and the cross-validation procedure uses cluster sampling and survey weights to form each fold. See Section 1.1 for more details.

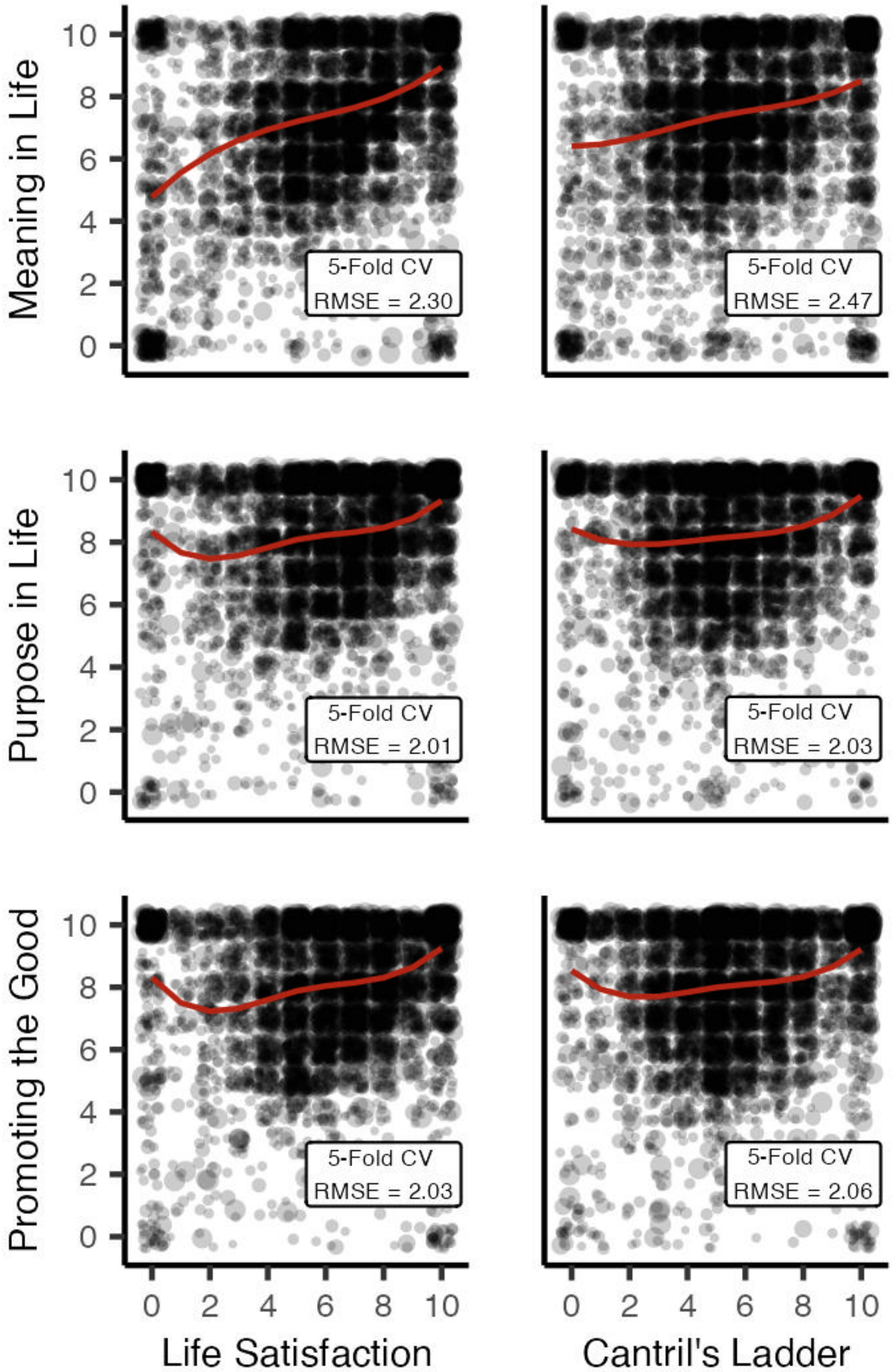

Figure 50: Scatterplots for Nigeria, Panel 2. Curves fit using a piecewise polynomial regression with B-splines. Regressions incorporate survey weights, and the cross-validation procedure uses cluster sampling and survey weights to form each fold. See Section 1.1 for more details.

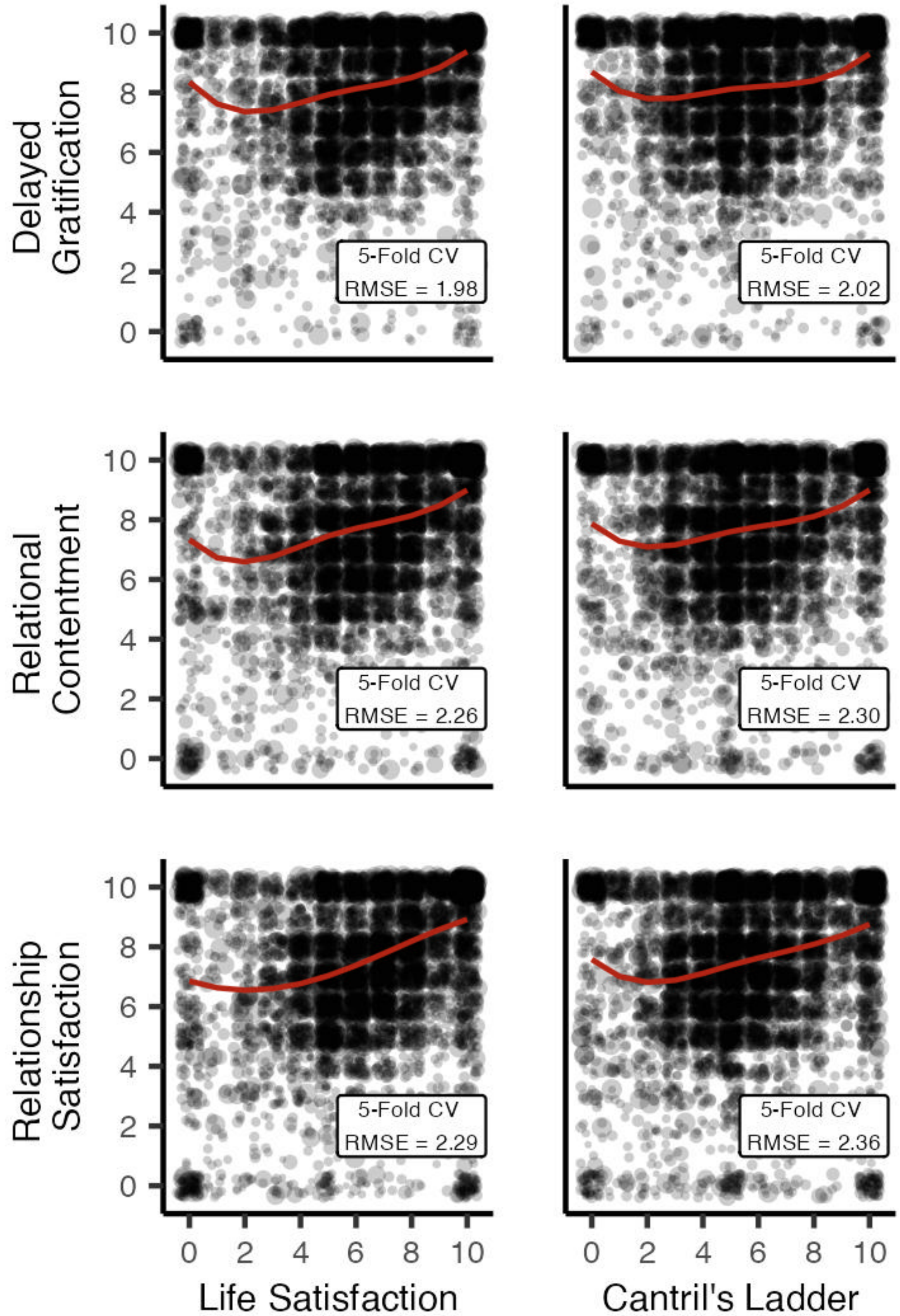

Figure 51: Scatterplots for Nigeria, Panel 3. Curves fit using a piecewise polynomial regression with B-splines. Regressions incorporate survey weights, and the cross-validation procedure uses cluster sampling and survey weights to form each fold. See Section 1.1 for more details.

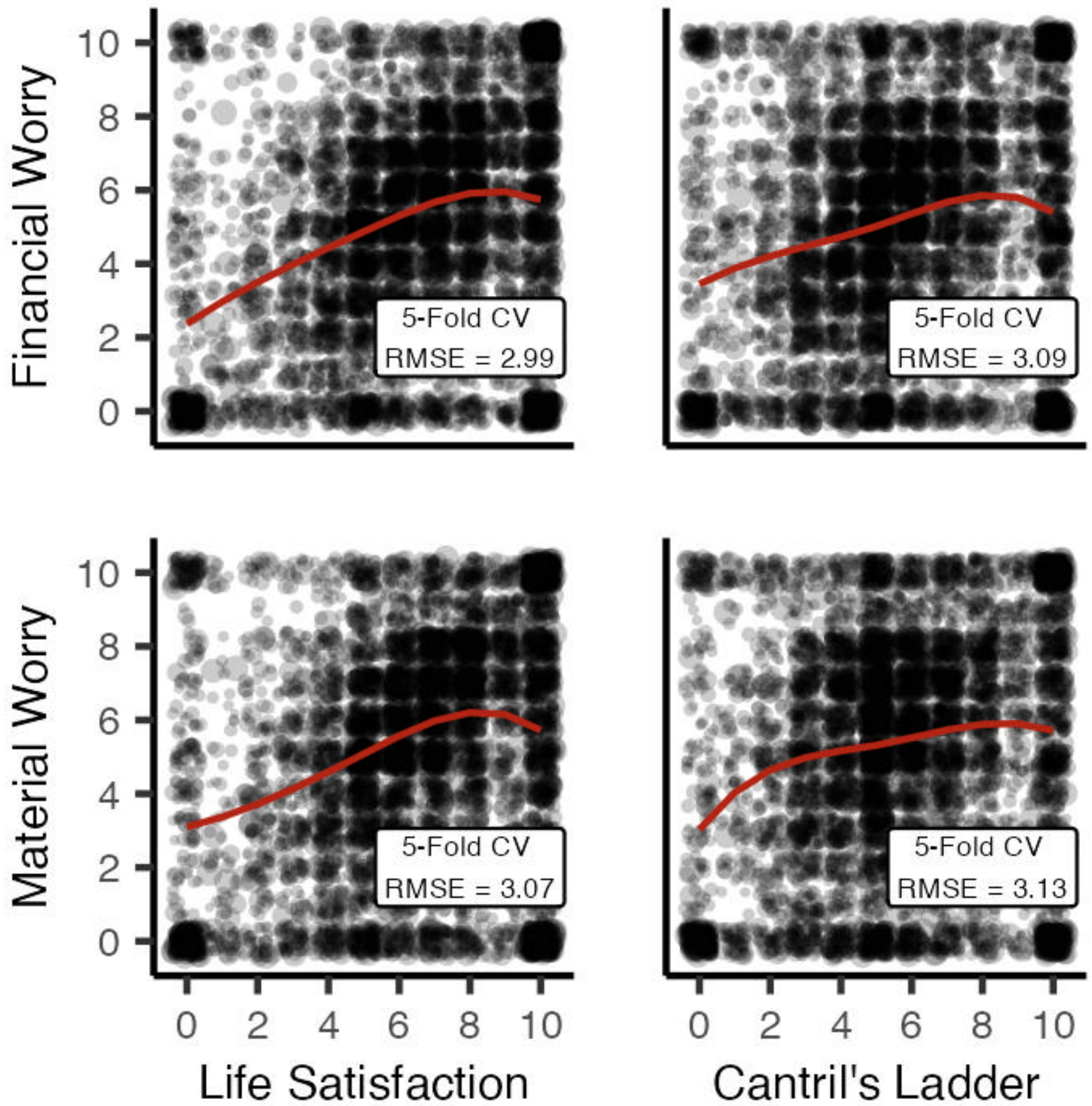

Figure 52: Scatterplots for Nigeria, Panel 4. Curves fit using a piecewise polynomial regression with B-splines. Regressions incorporate survey weights, and the cross-validation procedure uses cluster sampling and survey weights to form each fold. See Section 1.1 for more details.

Section 16

Scatterplots for Philippines

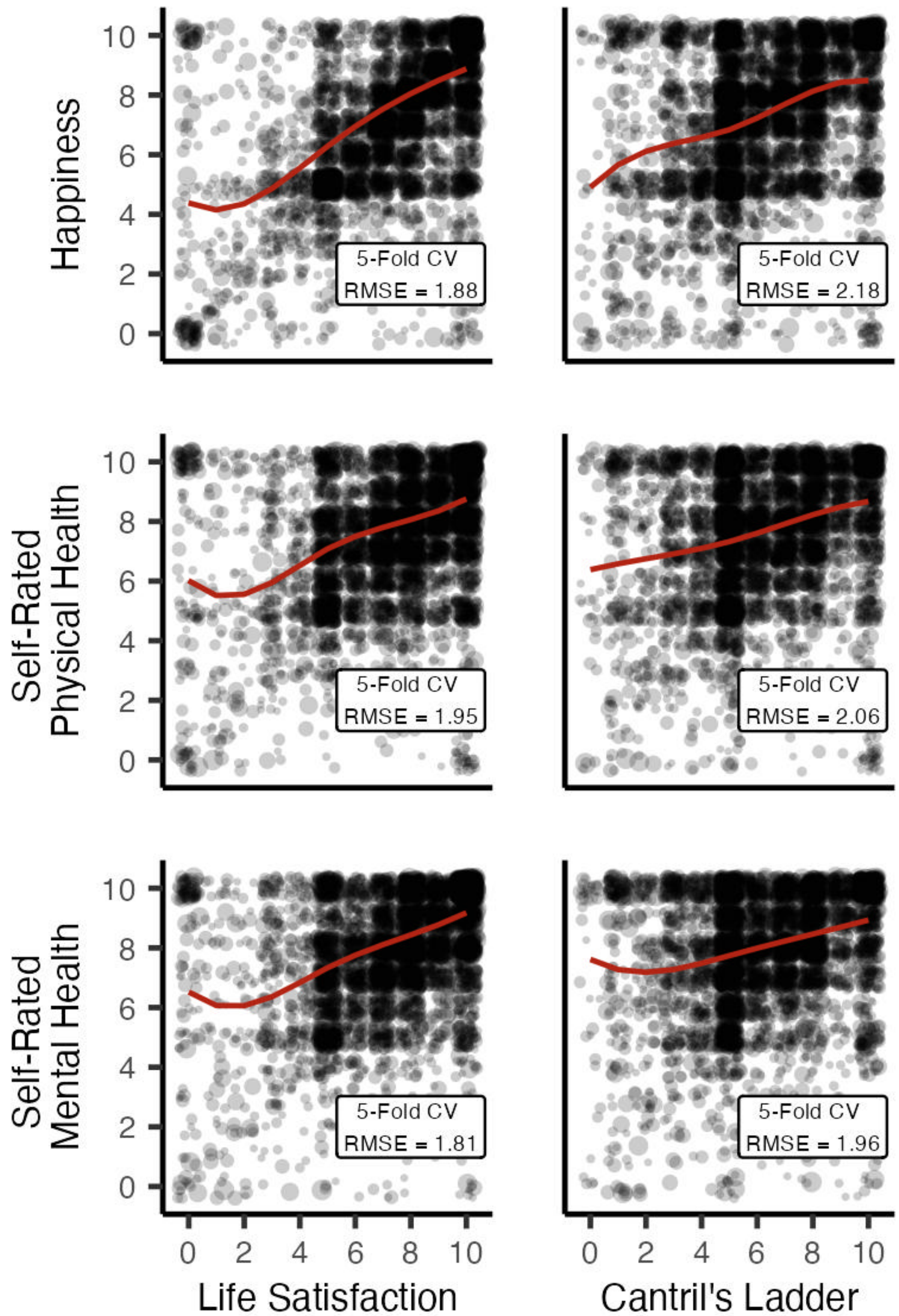

Figure 53: Scatterplots for Philippines, Panel 1. Curves fit using a piecewise polynomial regression with B-splines. Regressions incorporate survey weights, and the cross-validation procedure uses cluster sampling and survey weights to form each fold. See Section 1.1 for more details.

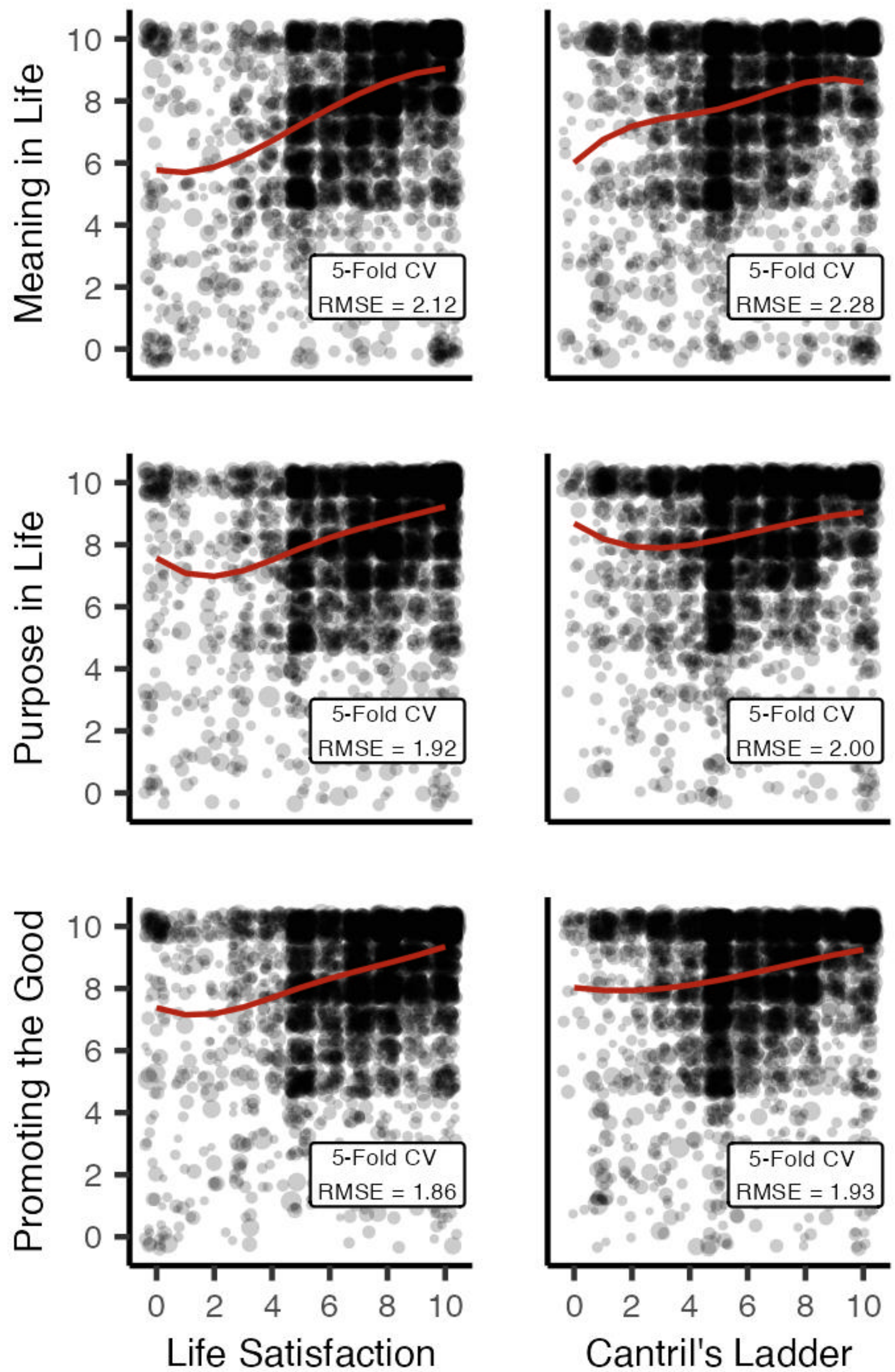

Figure 54: Scatterplots for Philippines, Panel 2. Curves fit using a piecewise polynomial regression with B-splines. Regressions incorporate survey weights, and the cross-validation procedure uses cluster sampling and survey weights to form each fold. See Section 1.1 for more details.

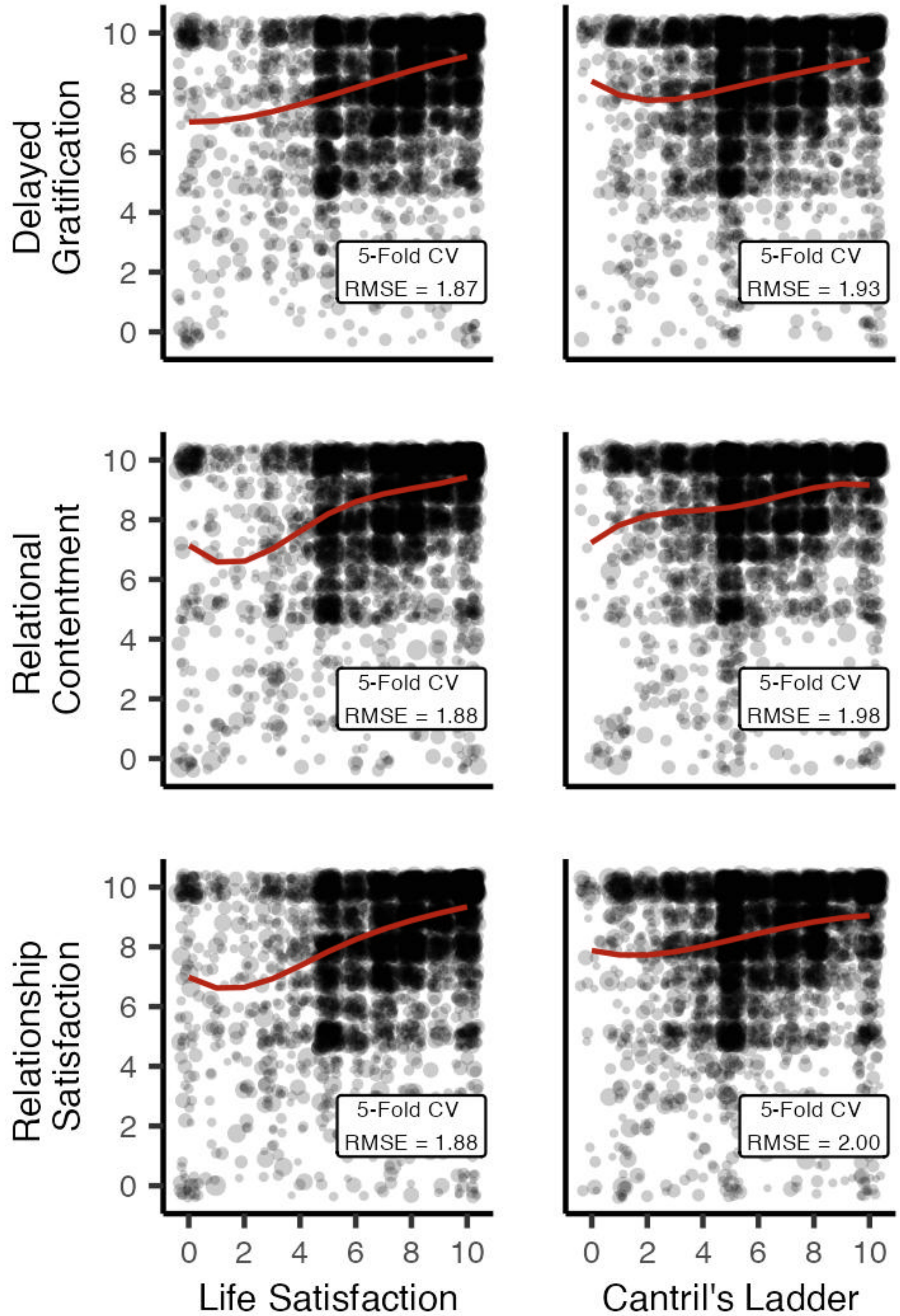

Figure 55: Scatterplots for Philippines, Panel 3. Curves fit using a piecewise polynomial regression with B-splines. Regressions incorporate survey weights, and the cross-validation procedure uses cluster sampling and survey weights to form each fold. See Section 1.1 for more details.

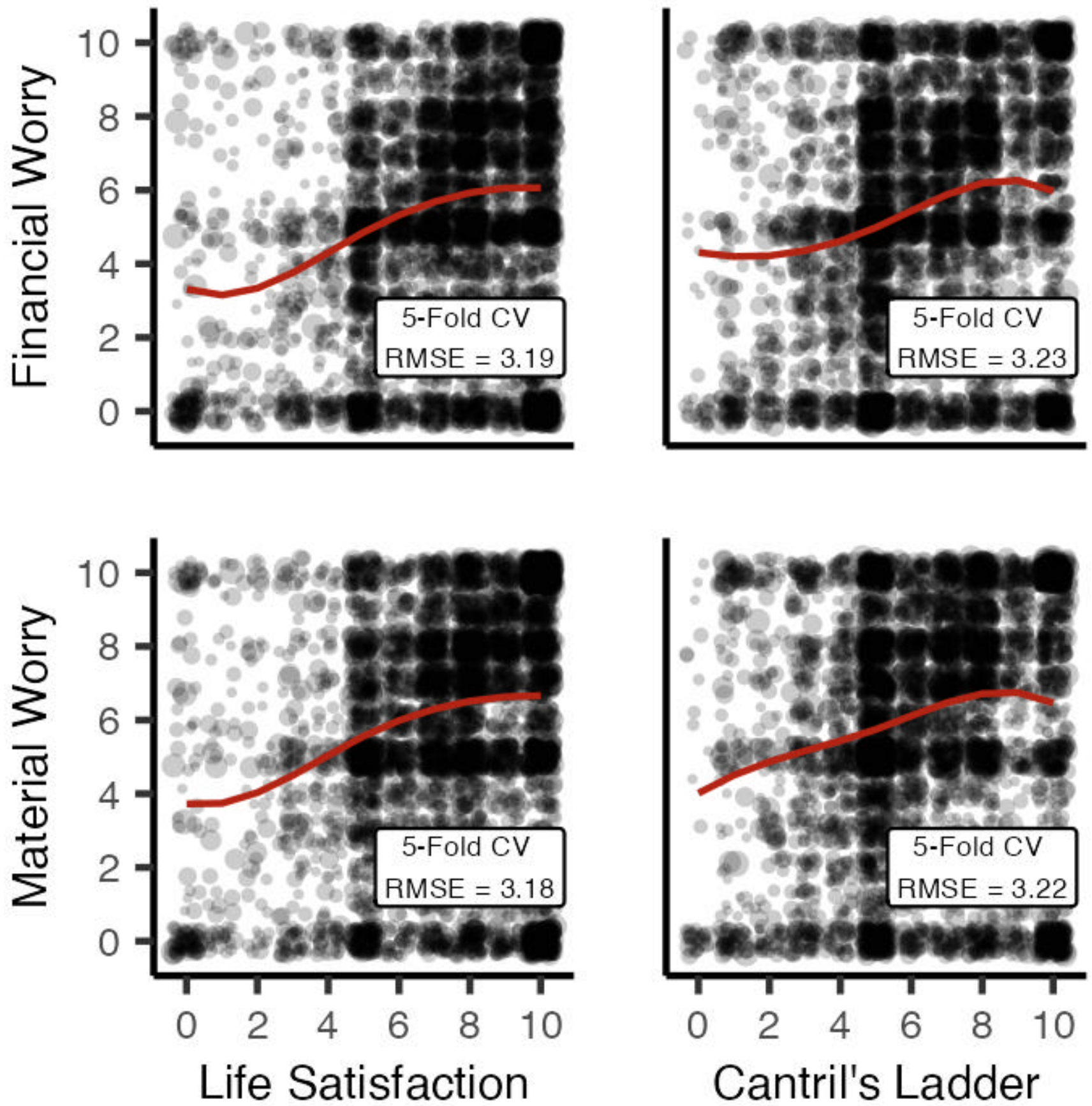

Figure 56: Scatterplots for Philippines, Panel 4. Curves fit using a piecewise polynomial regression with B-splines. Regressions incorporate survey weights, and the cross-validation procedure uses cluster sampling and survey weights to form each fold. See Section 1.1 for more details.

Section 17

Scatterplots for Poland

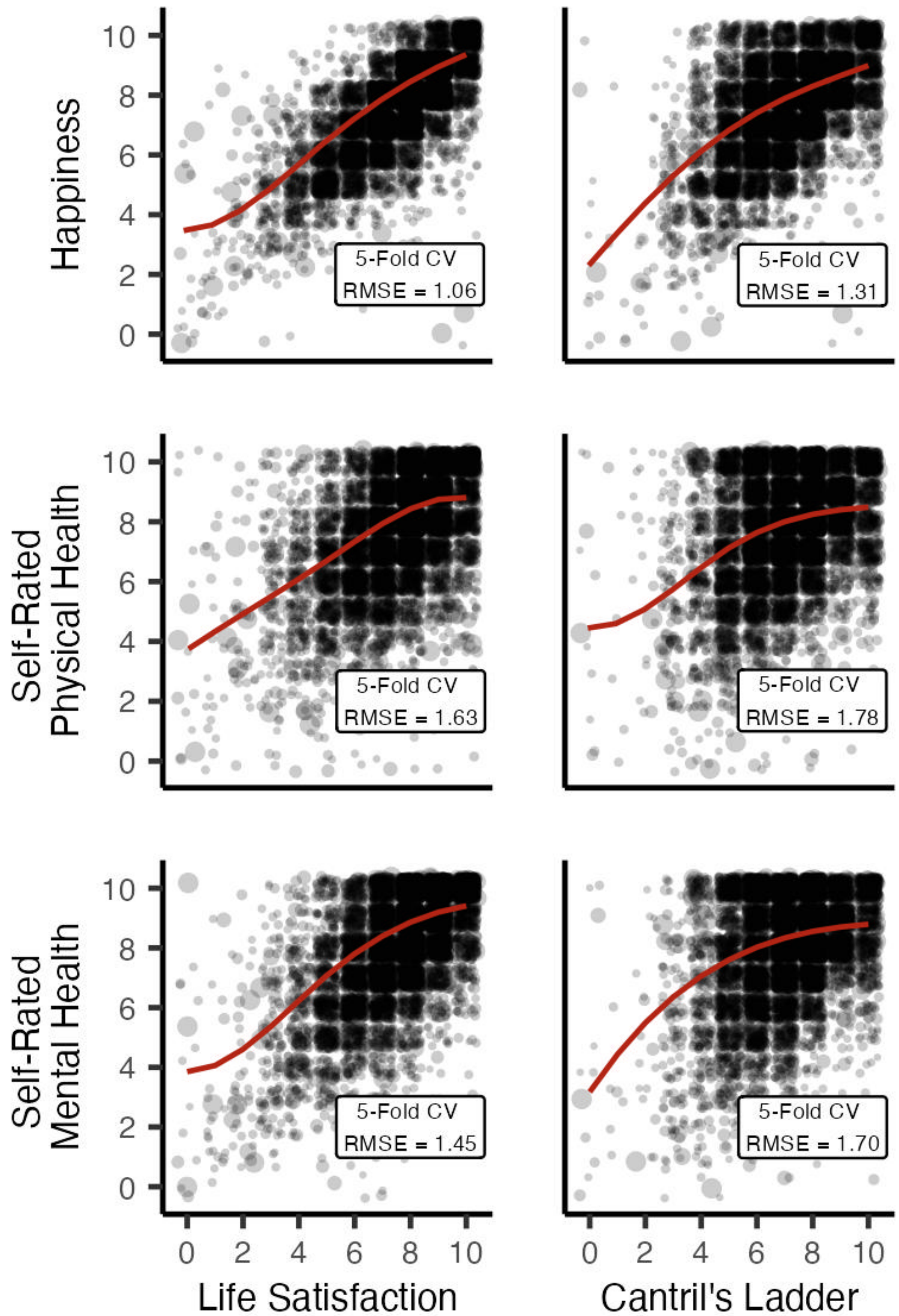

Figure 57: Scatterplots for Poland, Panel 1. Curves fit using a piecewise polynomial regression with B-splines. Regressions incorporate survey weights, and the cross-validation procedure uses cluster sampling and survey weights to form each fold. See Section 1.1 for more details.

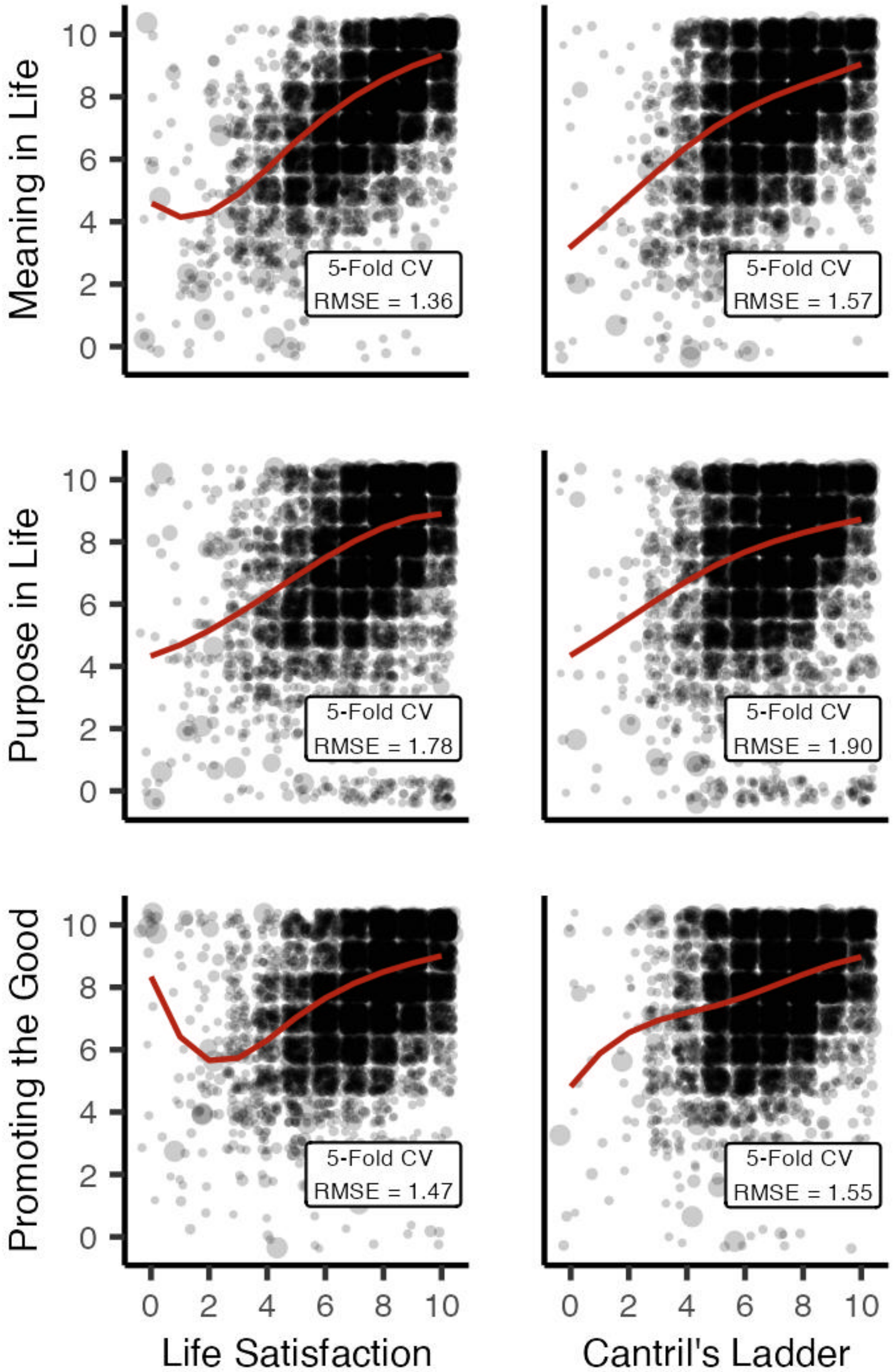

Figure 58: Scatterplots for Poland, Panel 2. Curves fit using a piecewise polynomial regression with B-splines. Regressions incorporate survey weights, and the cross-validation procedure uses cluster sampling and survey weights to form each fold. See Section 1.1 for more details.

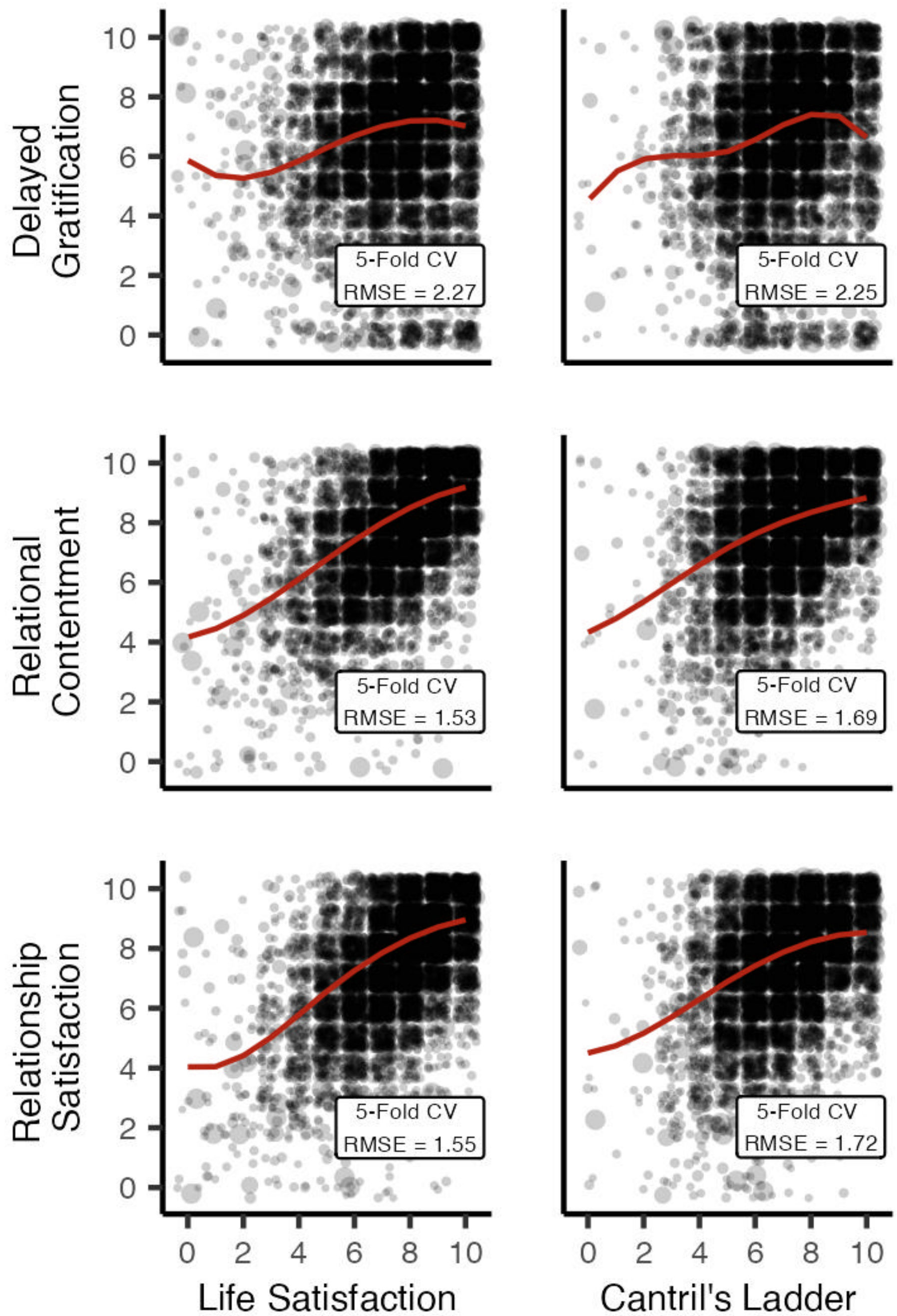

Figure 59: Scatterplots for Poland, Panel 3. Curves fit using a piecewise polynomial regression with B-splines. Regressions incorporate survey weights, and the cross-validation procedure uses cluster sampling and survey weights to form each fold. See Section 1.1 for more details.

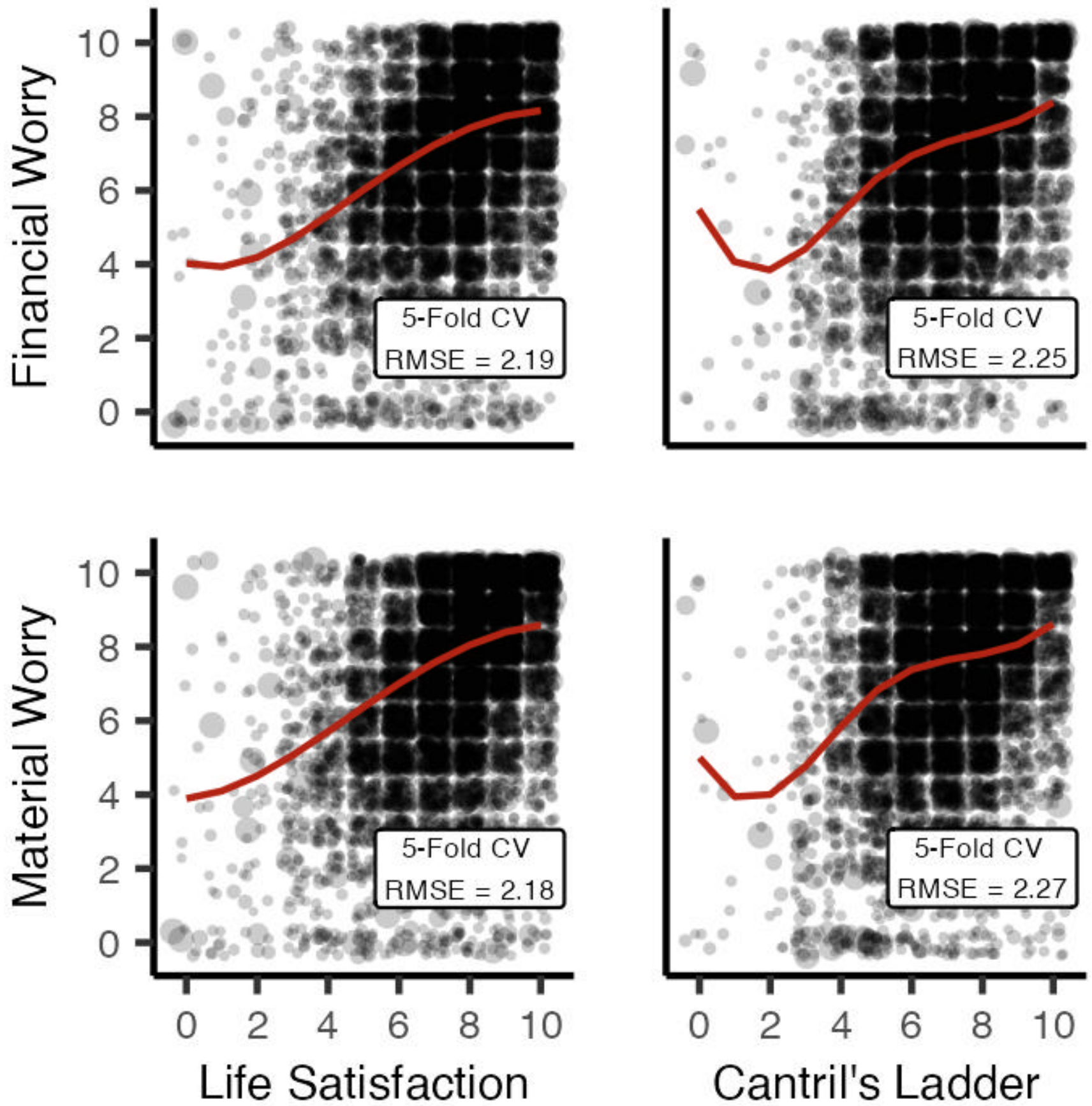

Figure 60: Scatterplots for Poland, Panel 4. Curves fit using a piecewise polynomial regression with B-splines. Regressions incorporate survey weights, and the cross-validation procedure uses cluster sampling and survey weights to form each fold. See Section 1.1 for more details.

Section 18

Scatterplots for South Africa

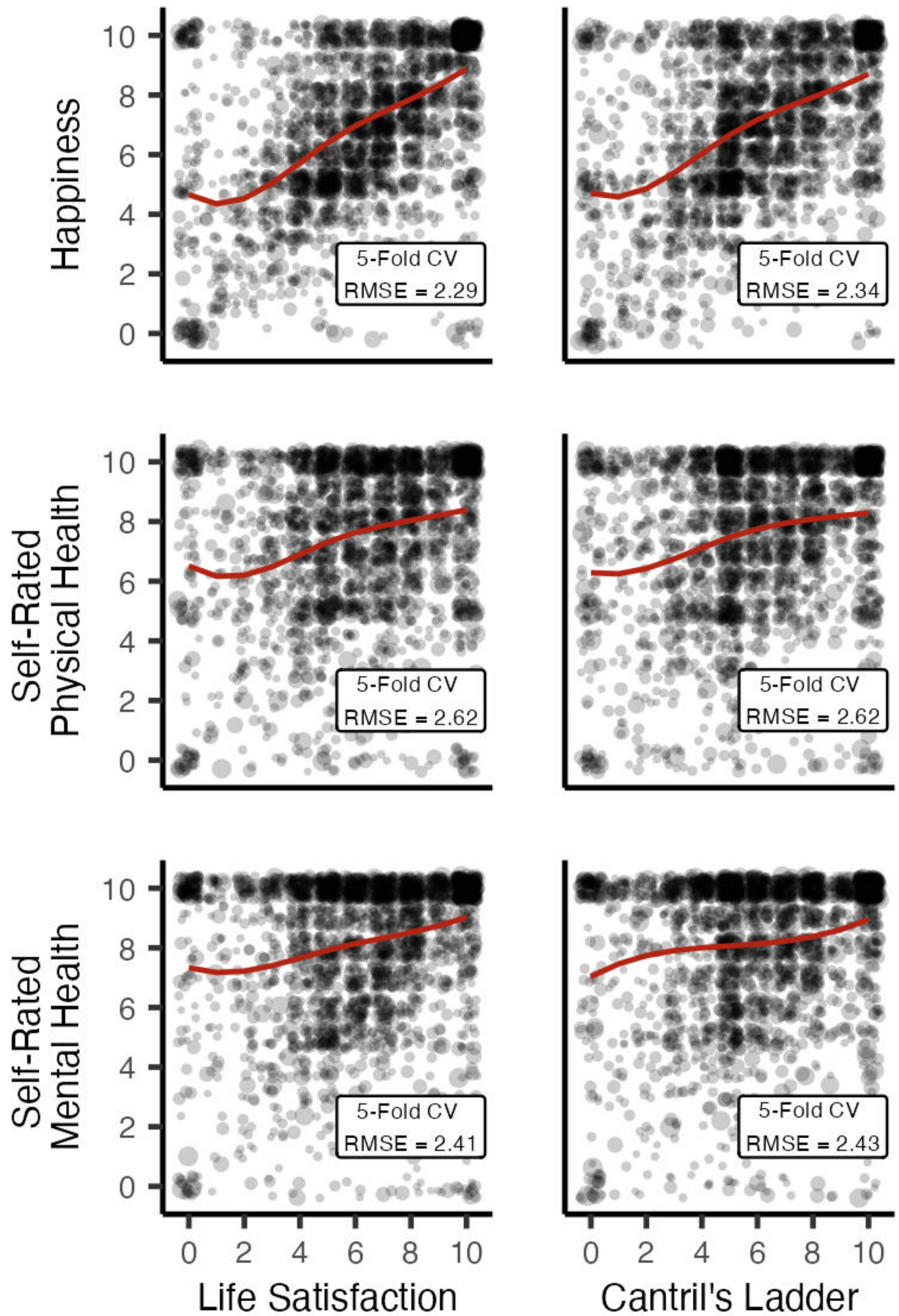

Figure 61: Scatterplots for South Africa, Panel 1. Curves fit using a piecewise polynomial regression with B-splines. Regressions incorporate survey weights, and the cross-validation procedure uses cluster sampling and survey weights to form each fold. See Section 1.1 for more details.

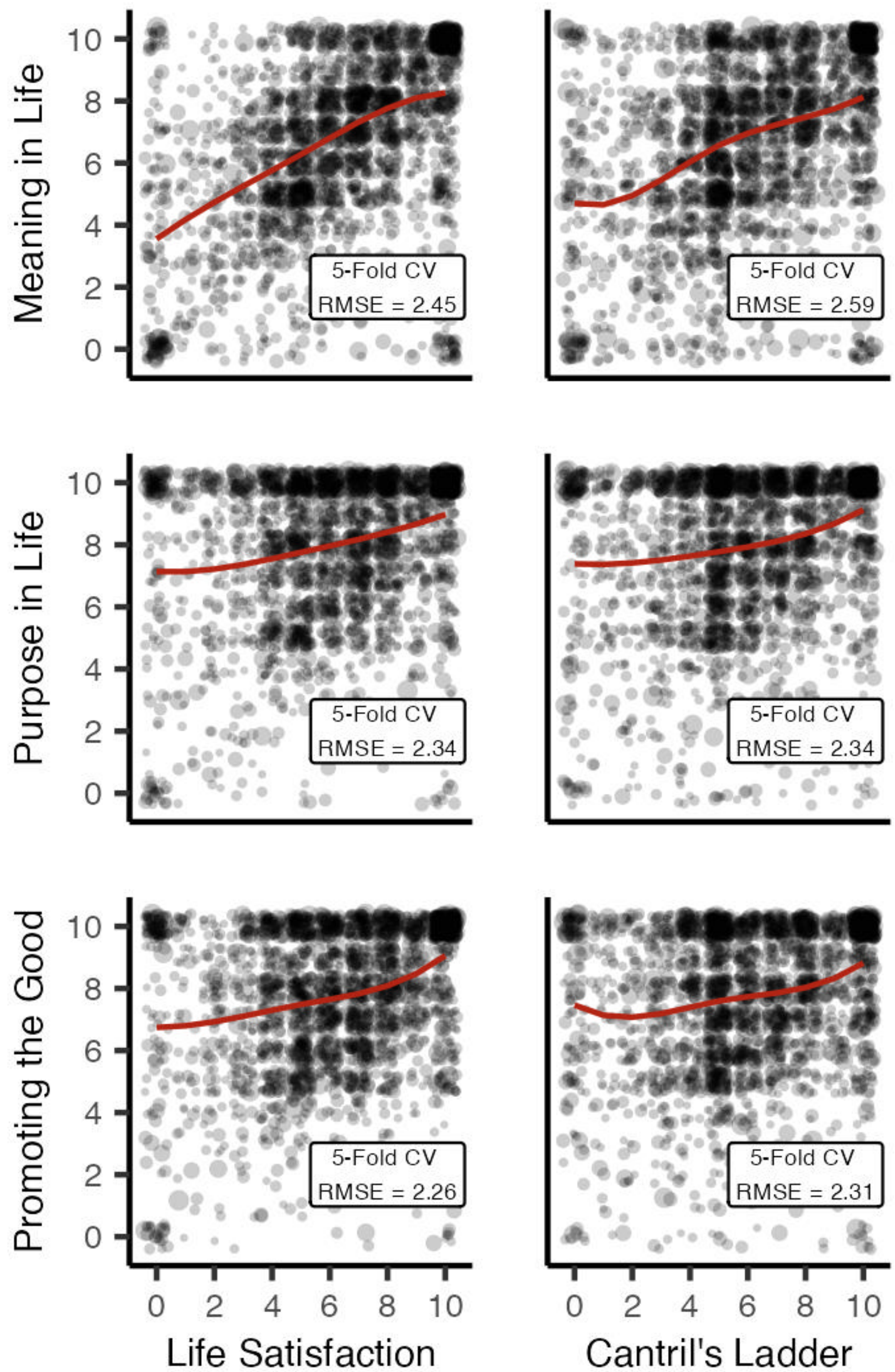

Figure 62: Scatterplots for South Africa, Panel 2. Curves fit using a piecewise polynomial regression with B-splines. Regressions incorporate survey weights, and the cross-validation procedure uses cluster sampling and survey weights to form each fold. See Section 1.1 for more details.

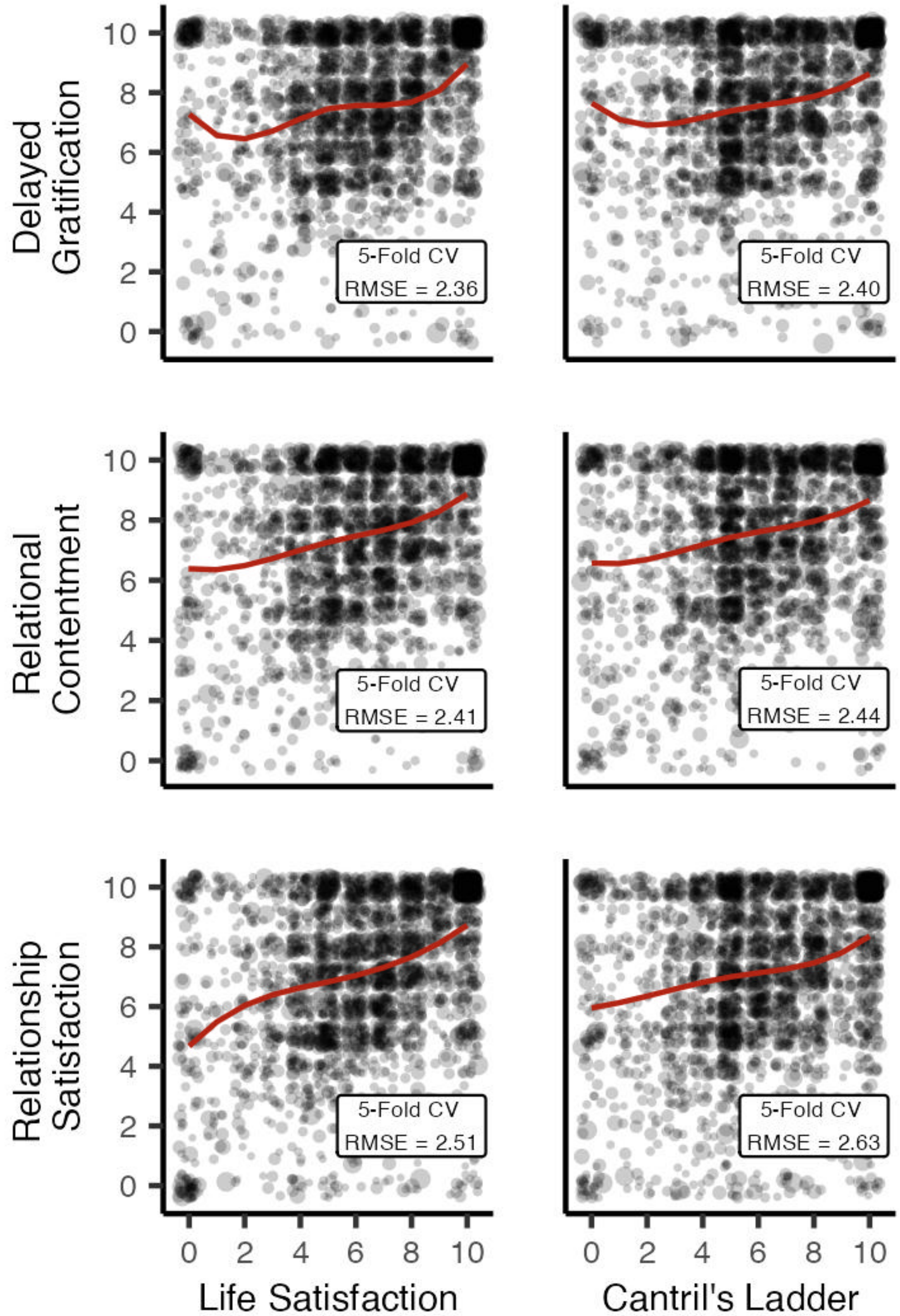

Figure 63: Scatterplots for South Africa, Panel 3. Curves fit using a piecewise polynomial regression with B-splines. Regressions incorporate survey weights, and the cross-validation procedure uses cluster sampling and survey weights to form each fold. See Section 1.1 for more details.

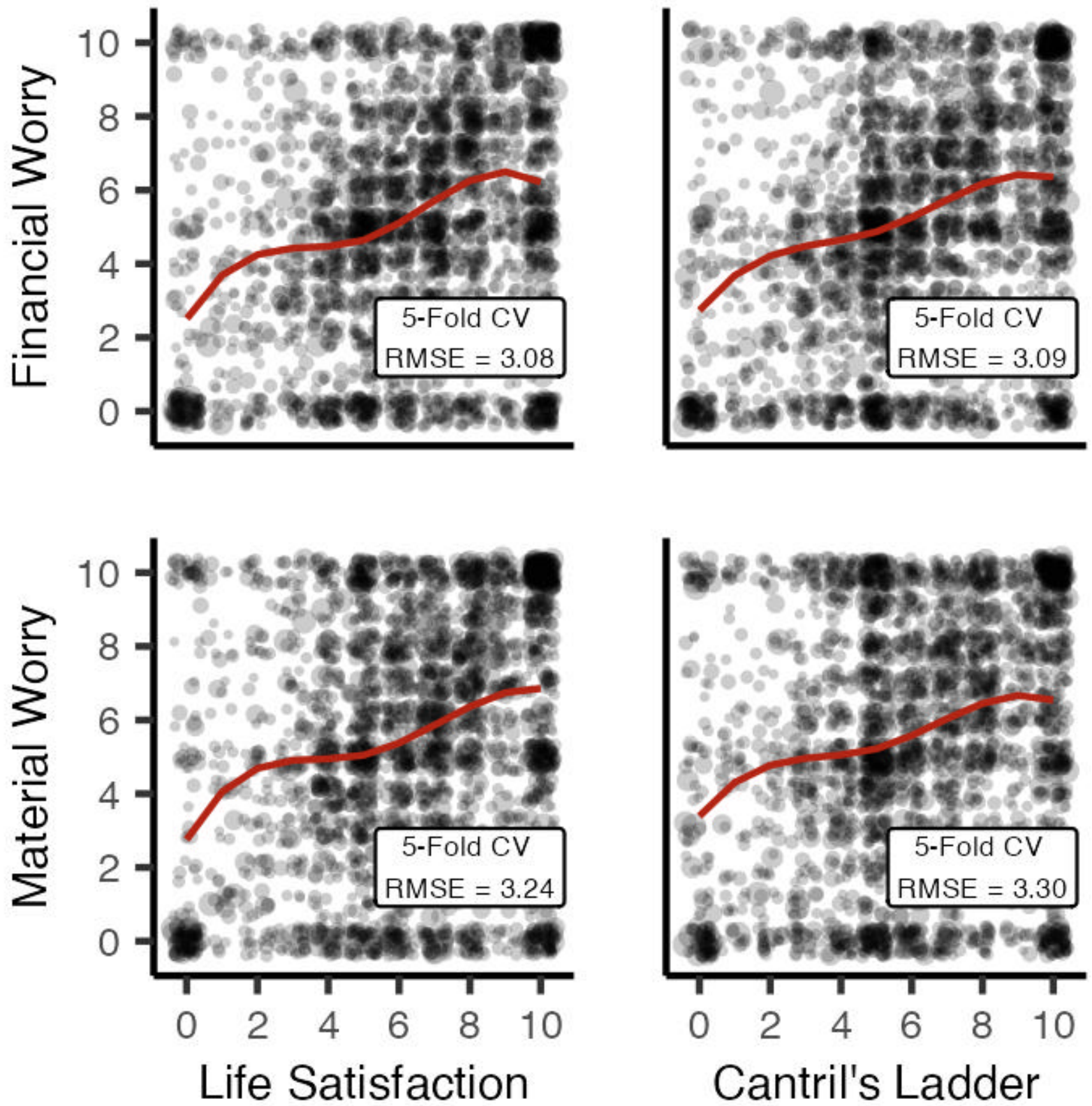

Figure 64: Scatterplots for South Africa, Panel 4. Curves fit using a piecewise polynomial regression with B-splines. Regressions incorporate survey weights, and the cross-validation procedure uses cluster sampling and survey weights to form each fold. See Section 1.1 for more details.

Section 19

Scatterplots for Spain

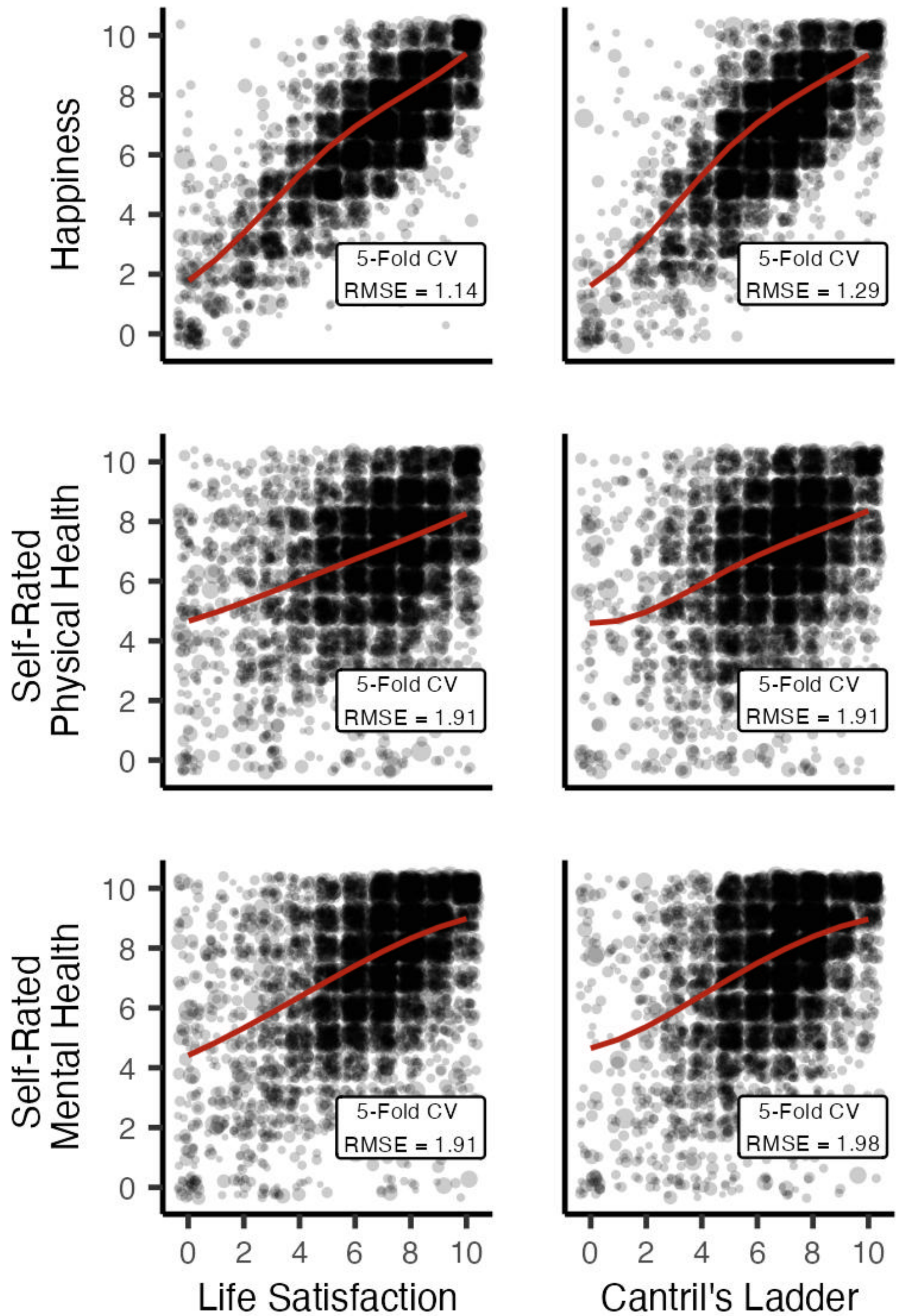

Figure 65: Scatterplots for Spain, Panel 1. Curves fit using a piecewise polynomial regression with B-splines. Regressions incorporate survey weights, and the cross-validation procedure uses cluster sampling and survey weights to form each fold. See Section 1.1 for more details.

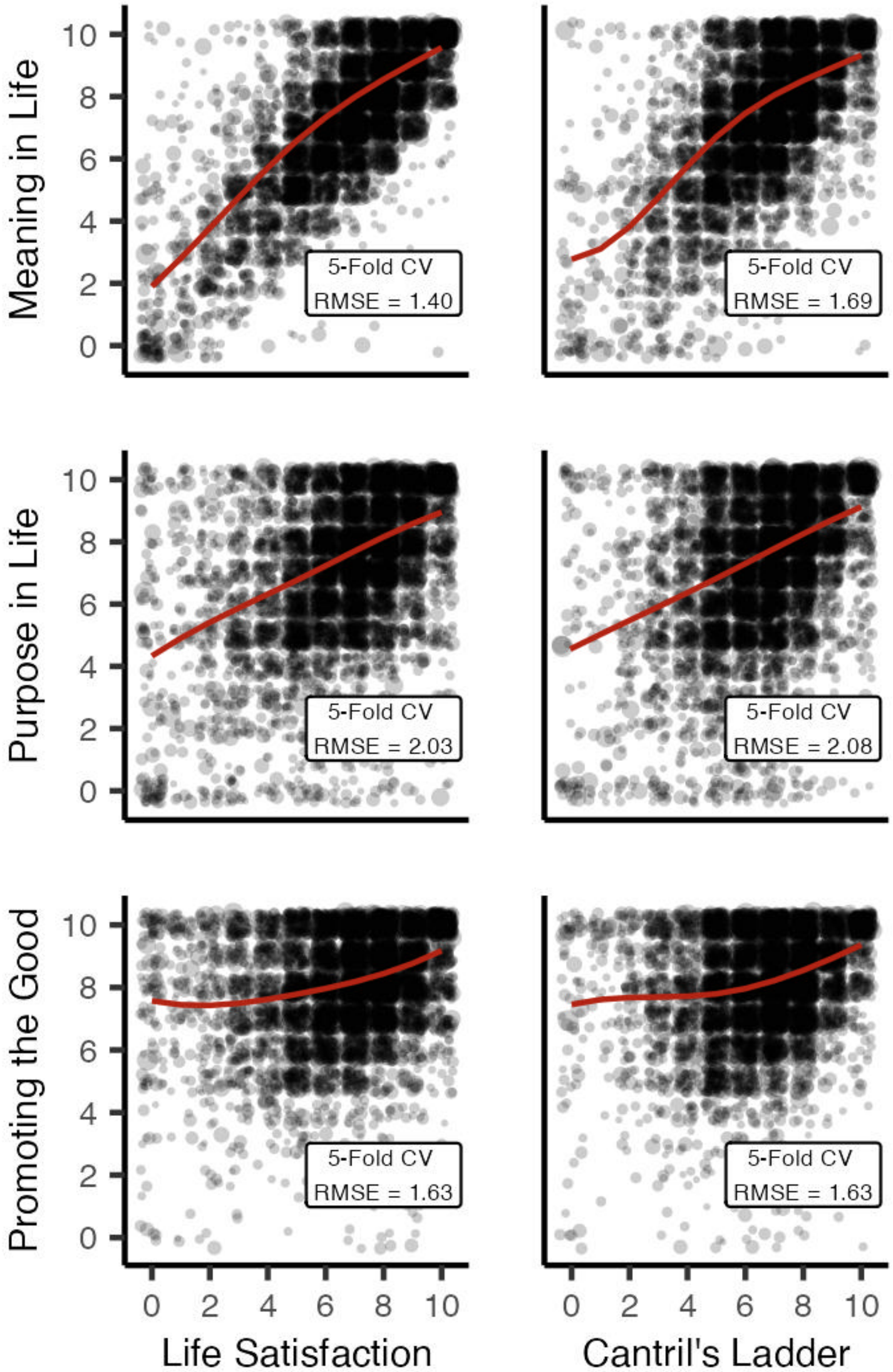

Figure 66: Scatterplots for Spain, Panel 2. Curves fit using a piecewise polynomial regression with B-splines. Regressions incorporate survey weights, and the cross-validation procedure uses cluster sampling and survey weights to form each fold. See Section 1.1 for more details.

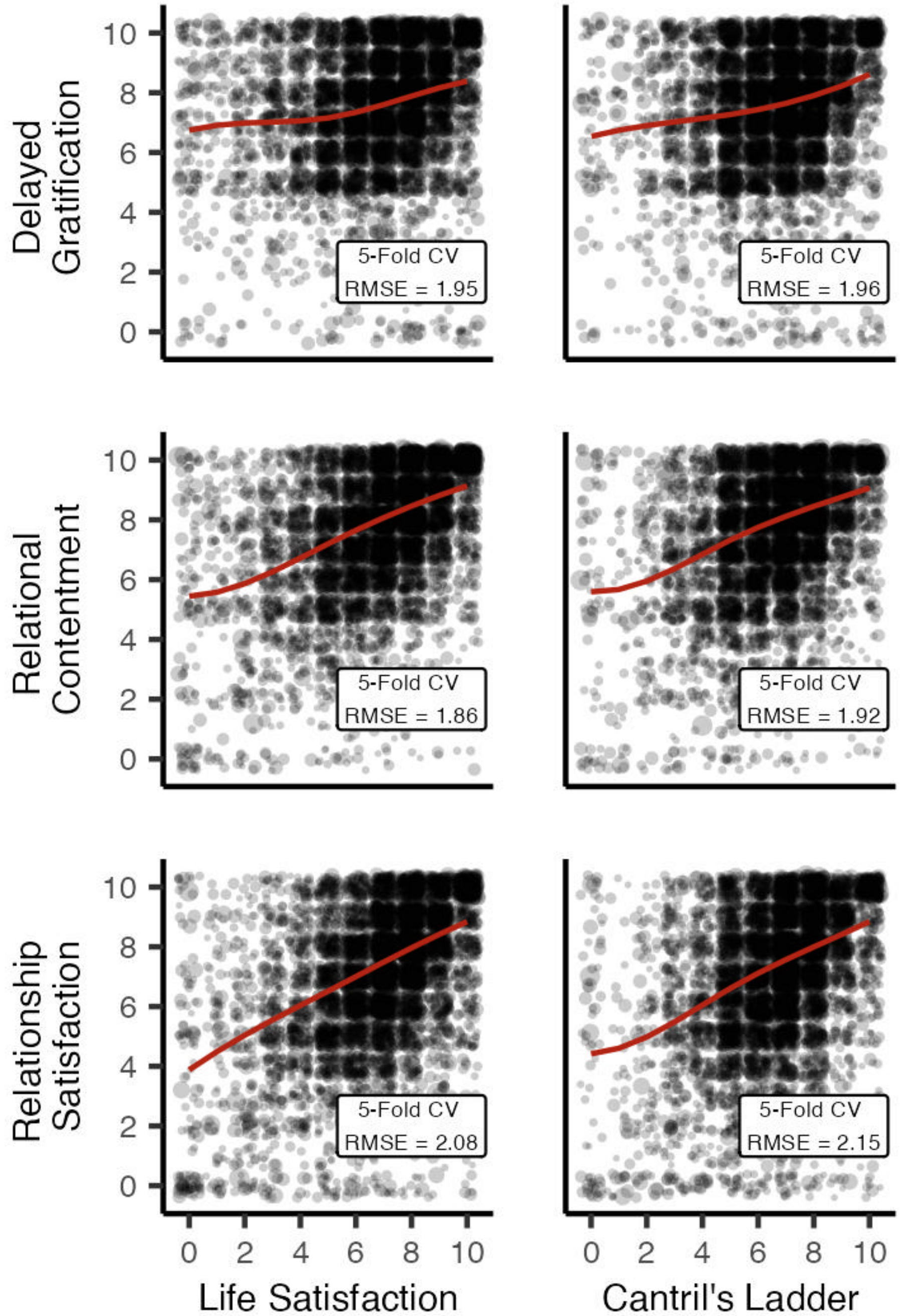

Figure 67: Scatterplots for Spain, Panel 3. Curves fit using a piecewise polynomial regression with B-splines. Regressions incorporate survey weights, and the cross-validation procedure uses cluster sampling and survey weights to form each fold. See Section 1.1 for more details.

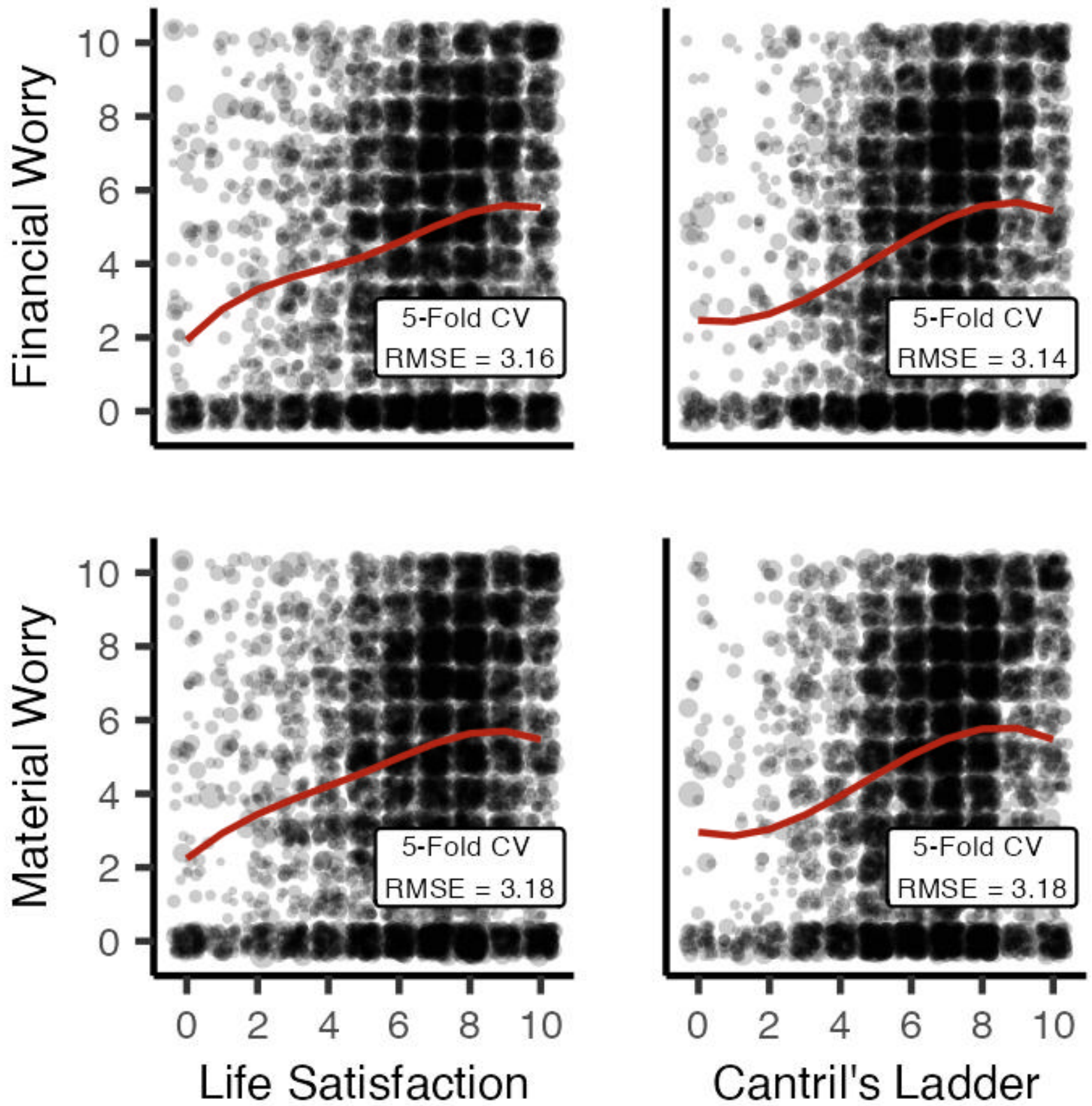

Figure 68: Scatterplots for Spain, Panel 4. Curves fit using a piecewise polynomial regression with B-splines. Regressions incorporate survey weights, and the cross-validation procedure uses cluster sampling and survey weights to form each fold. See Section 1.1 for more details.

Section 20

Scatterplots for Sweden

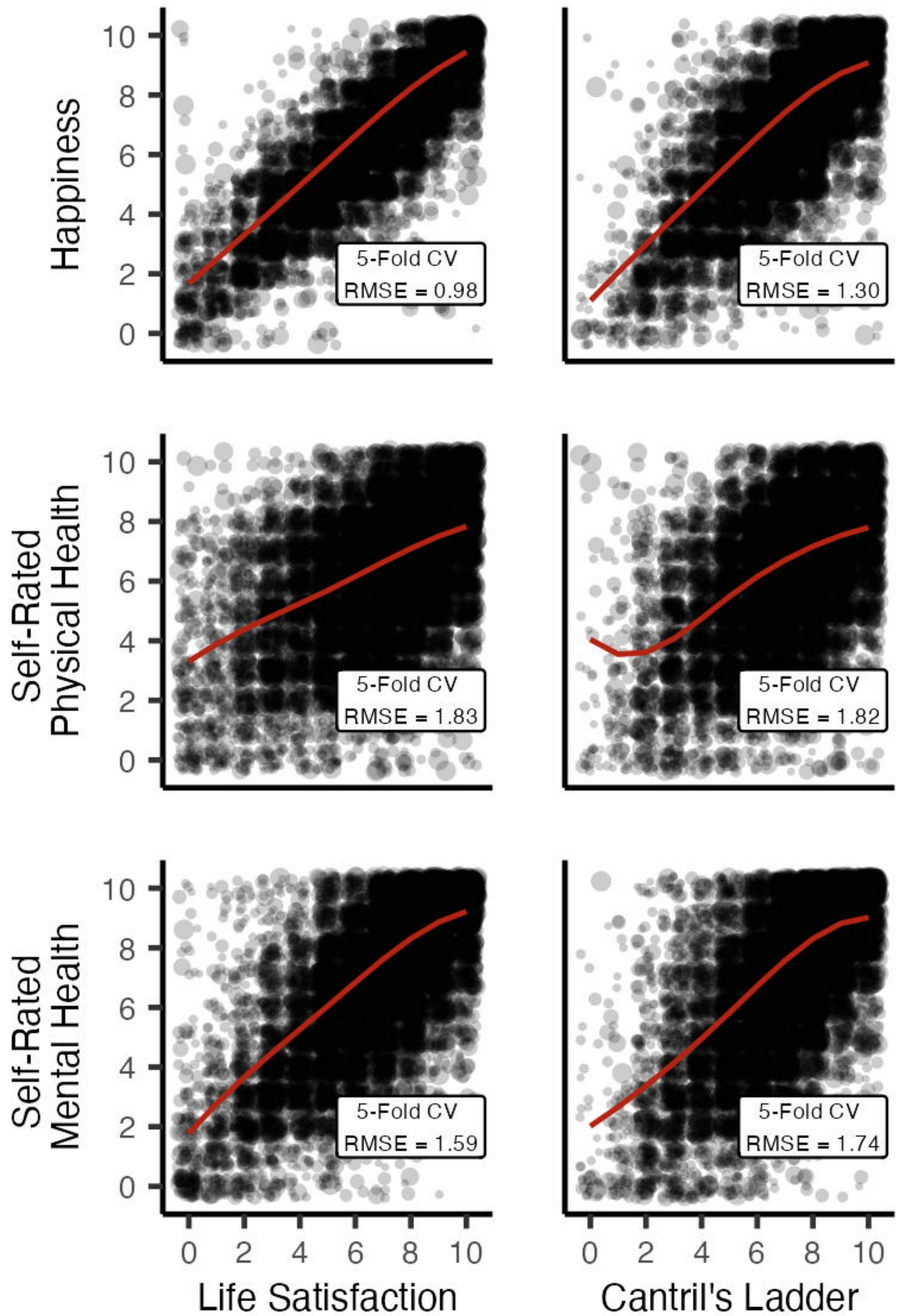

Figure 69: Scatterplots for Sweden, Panel 1. Curves fit using a piecewise polynomial regression with B-splines. Regressions incorporate survey weights, and the cross-validation procedure uses cluster sampling and survey weights to form each fold. See Section 1.1 for more details.

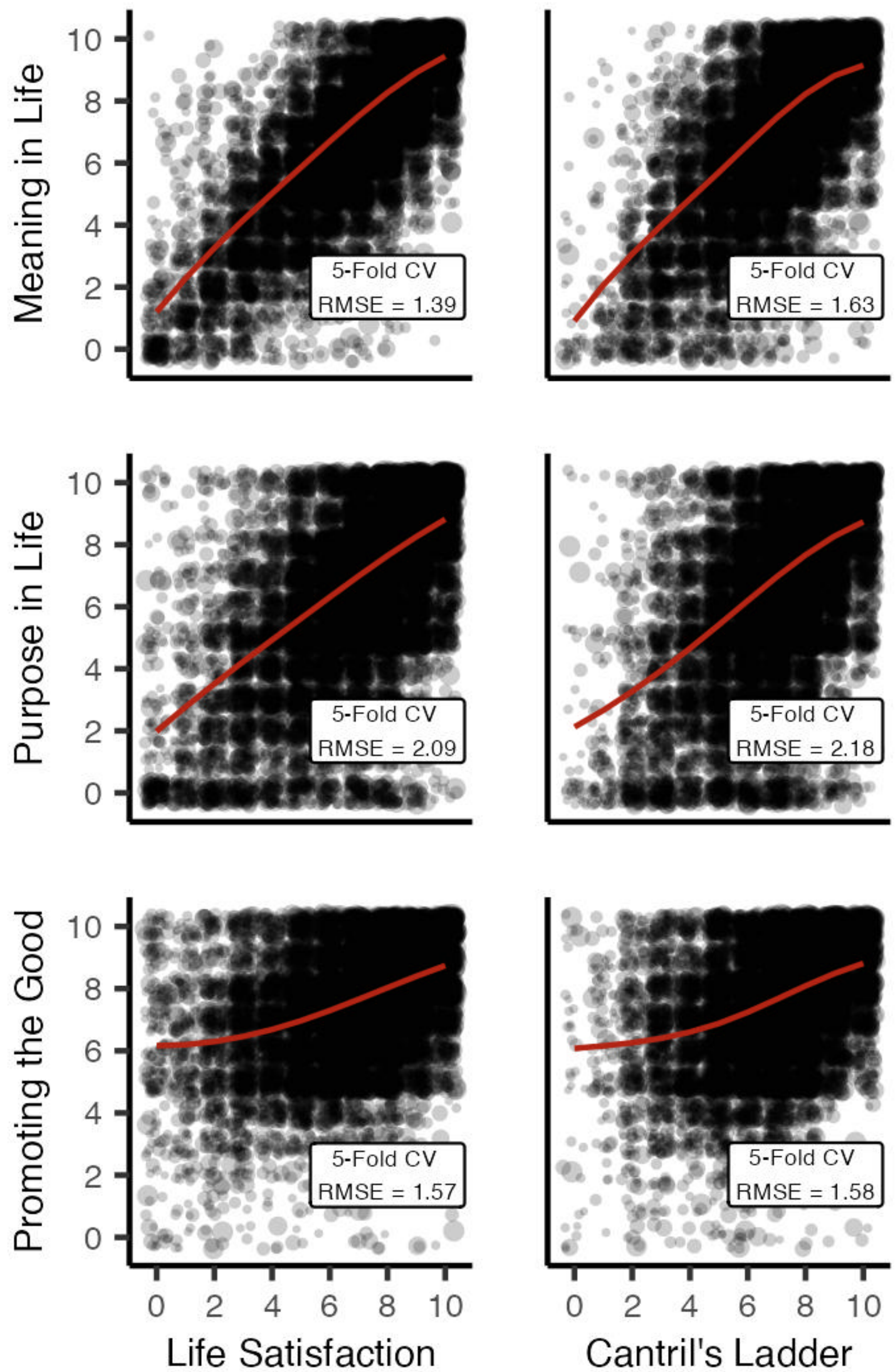

Figure 70: Scatterplots for Sweden, Panel 2. Curves fit using a piecewise polynomial regression with B-splines. Regressions incorporate survey weights, and the cross-validation procedure uses cluster sampling and survey weights to form each fold. See Section 1.1 for more details.

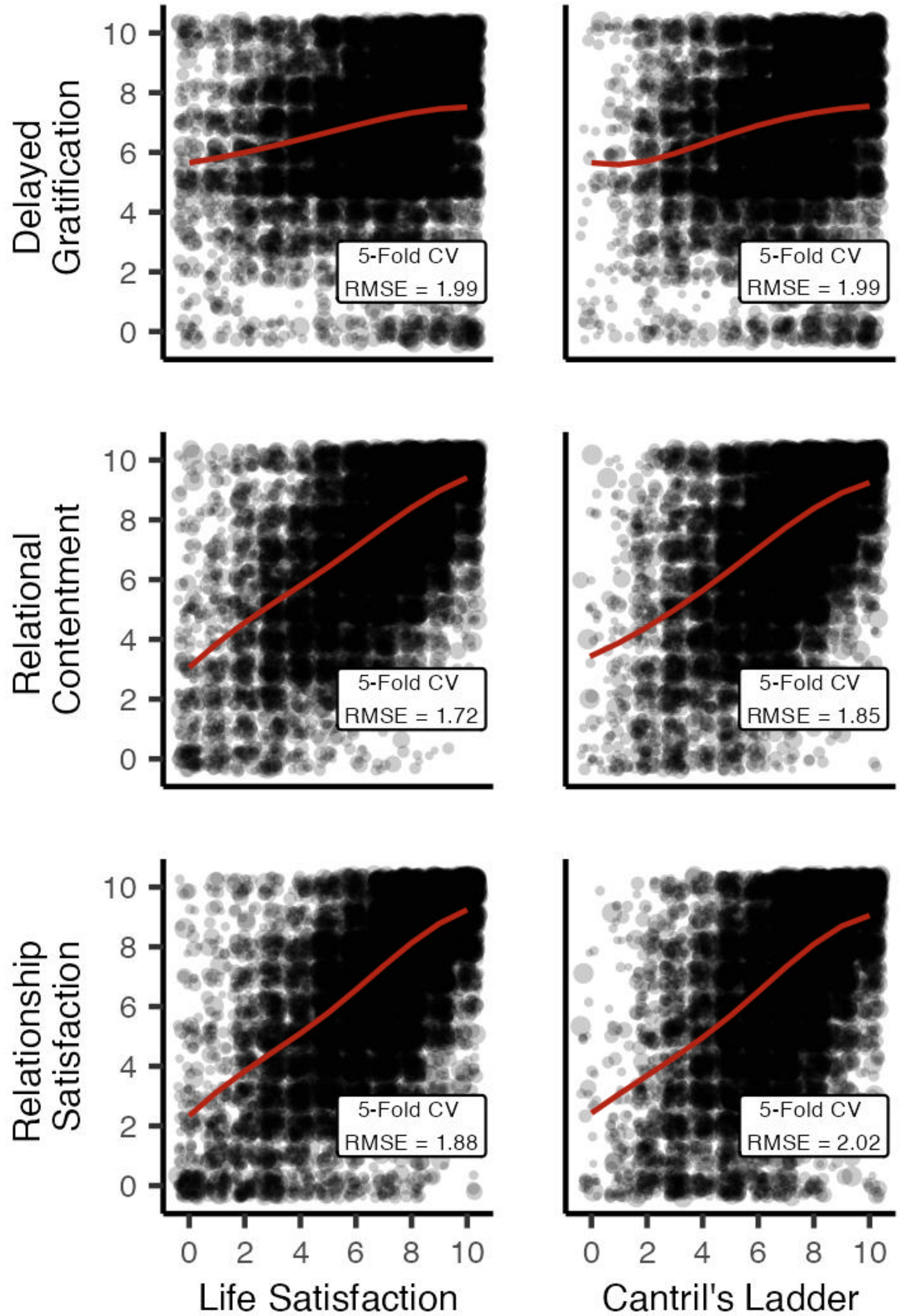

Figure 71: Scatterplots for Sweden, Panel 3. Curves fit using a piecewise polynomial regression with B-splines. Regressions incorporate survey weights, and the cross-validation procedure uses cluster sampling and survey weights to form each fold. See Section 1.1 for more details.

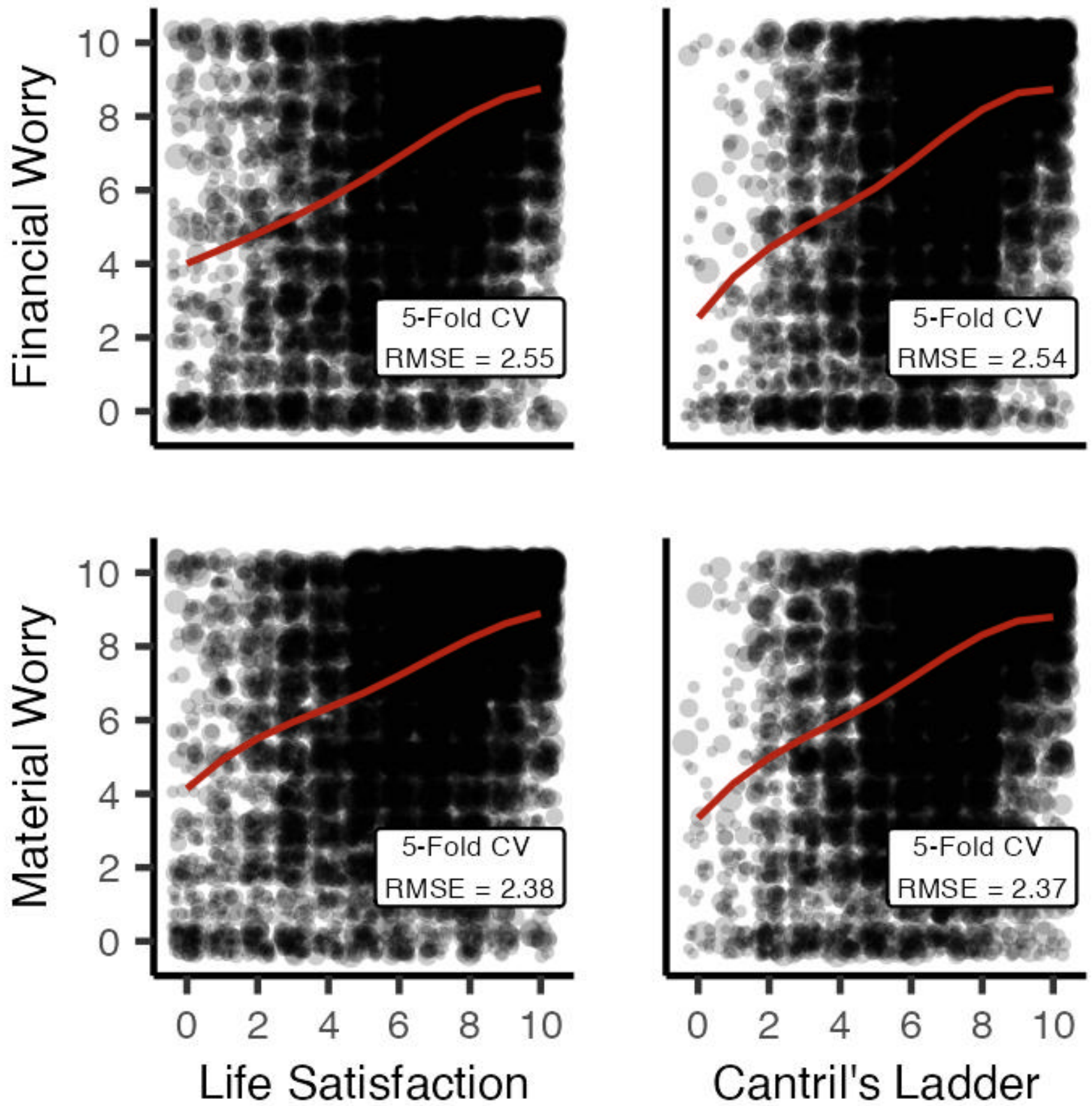

Figure 72: Scatterplots for Sweden, Panel 4. Curves fit using a piecewise polynomial regression with B-splines. Regressions incorporate survey weights, and the cross-validation procedure uses cluster sampling and survey weights to form each fold. See Section 1.1 for more details.

Section 21

Scatterplots for Tanzania

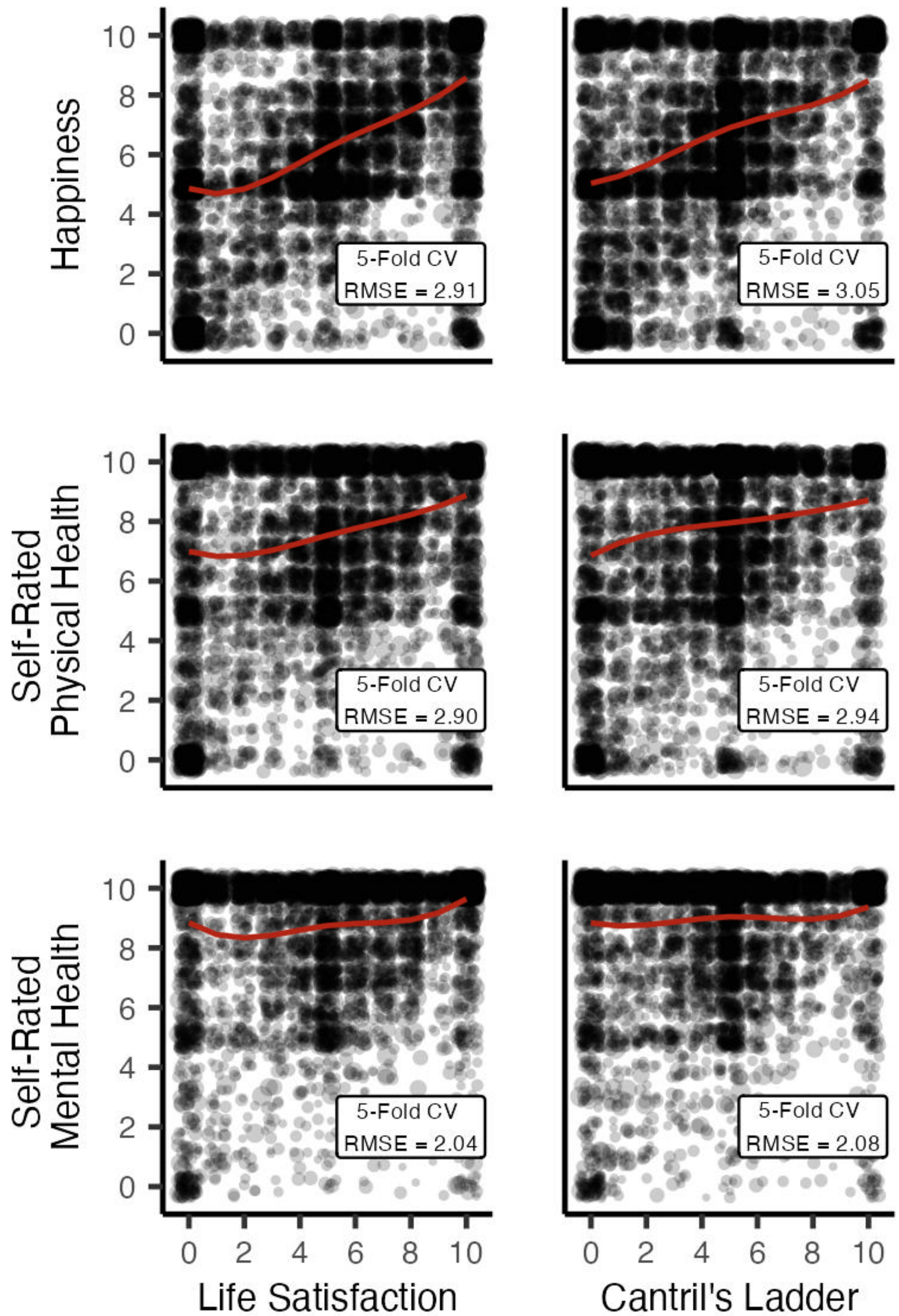

Figure 73: Scatterplots for Tanzania, Panel 1. Curves fit using a piecewise polynomial regression with B-splines. Regressions incorporate survey weights, and the cross-validation procedure uses cluster sampling and survey weights to form each fold. See Section 1.1 for more details.

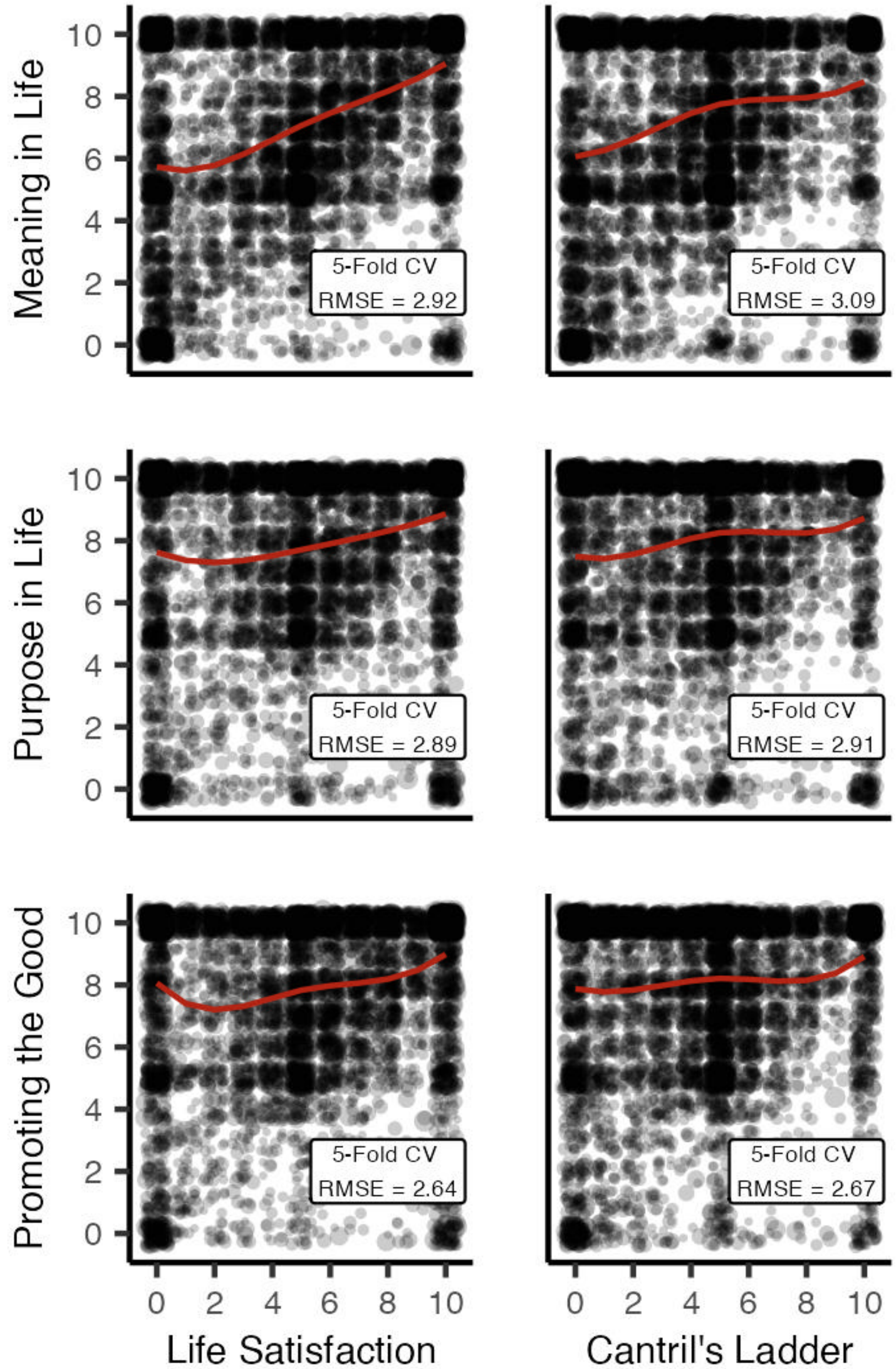

Figure 74: Scatterplots for Tanzania, Panel 2. Curves fit using a piecewise polynomial regression with B-splines. Regressions incorporate survey weights, and the cross-validation procedure uses cluster sampling and survey weights to form each fold. See Section 1.1 for more details.

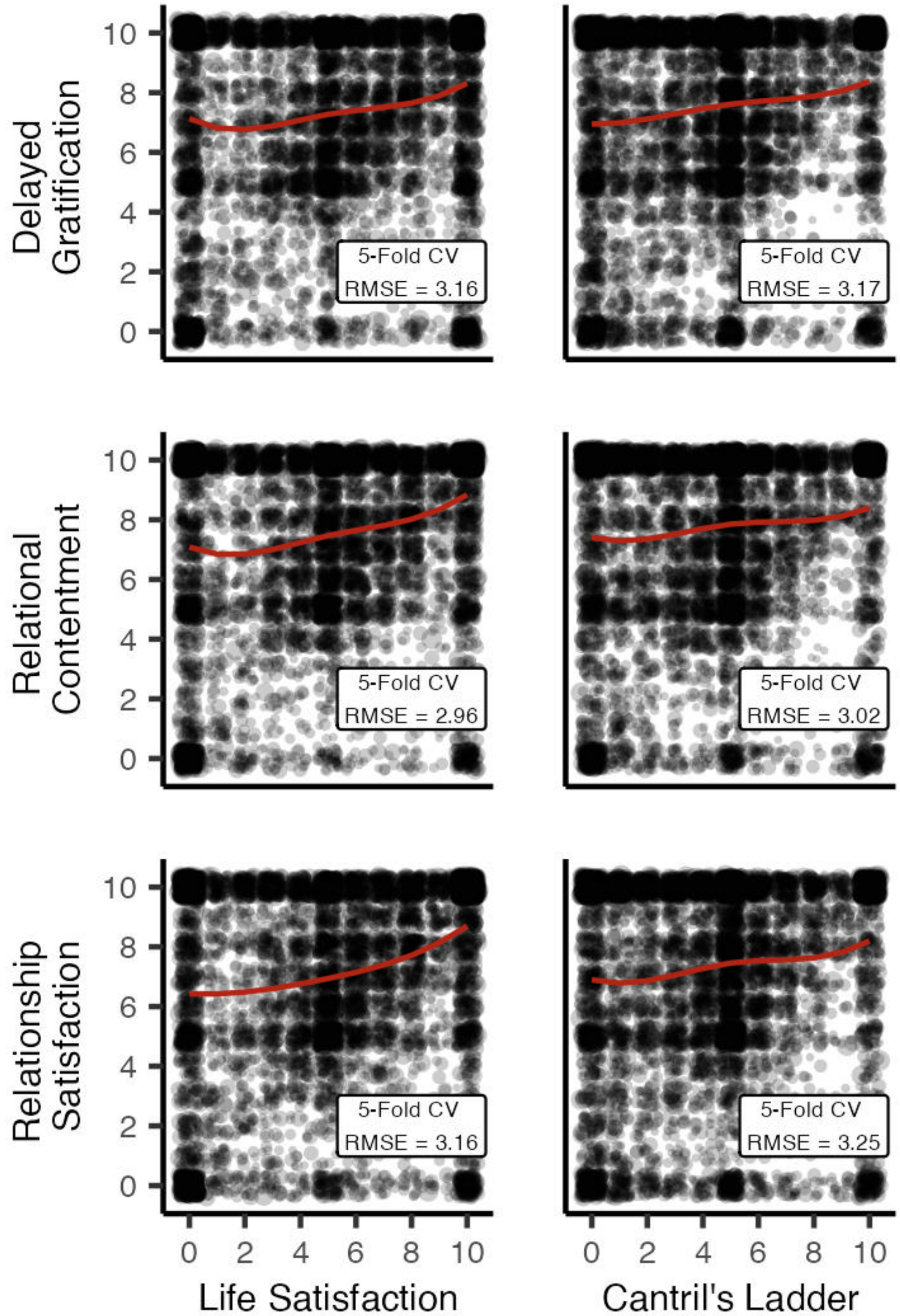

Figure 75: Scatterplots for Tanzania, Panel 3. Curves fit using a piecewise polynomial regression with B-splines. Regressions incorporate survey weights, and the cross-validation procedure uses cluster sampling and survey weights to form each fold. See Section 1.1 for more details.

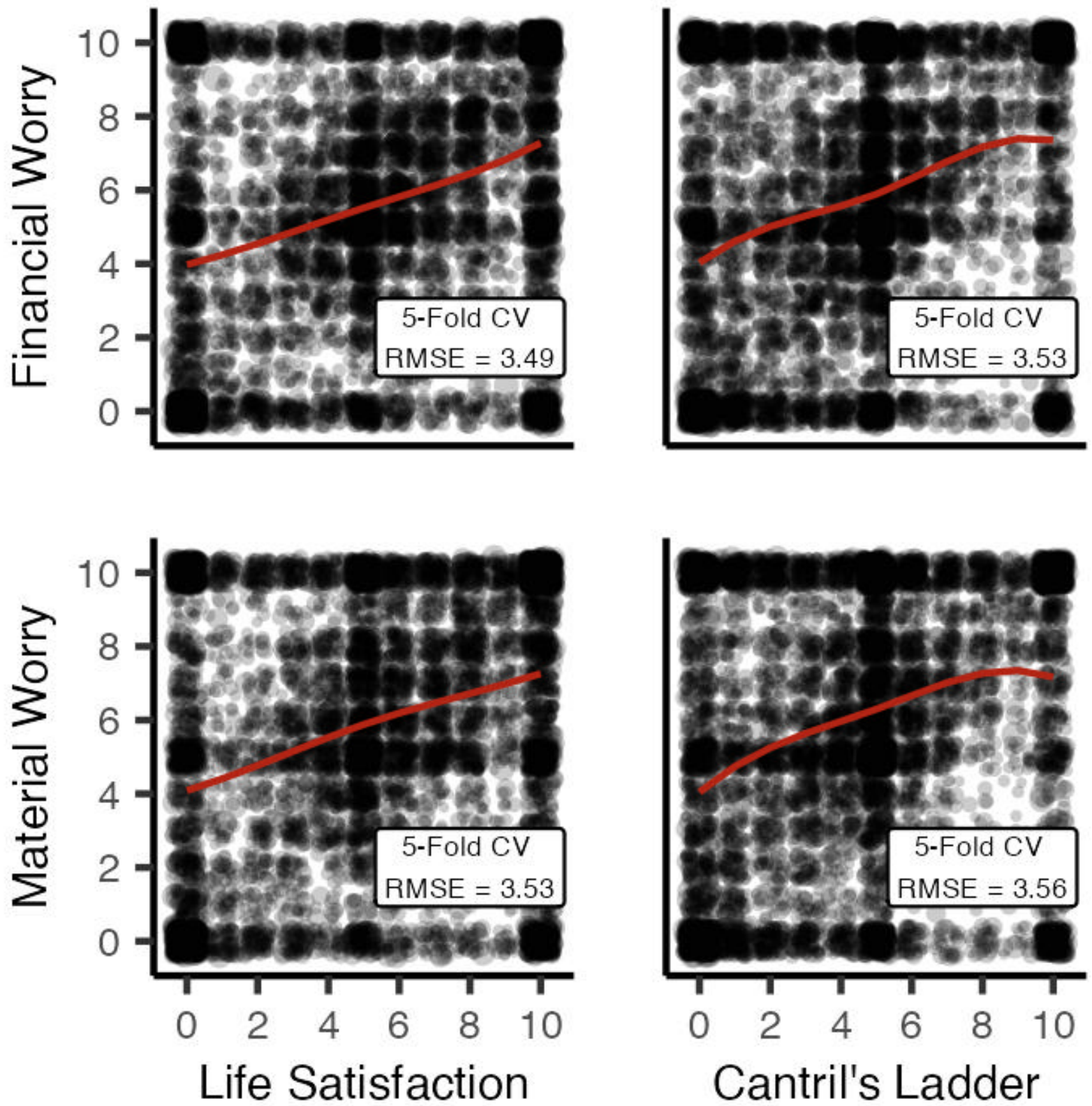

Figure 76: Scatterplots for Tanzania, Panel 4. Curves fit using a piecewise polynomial regression with B-splines. Regressions incorporate survey weights, and the cross-validation procedure uses cluster sampling and survey weights to form each fold. See Section 1.1 for more details.

Section 22

Scatterplots for Turkey

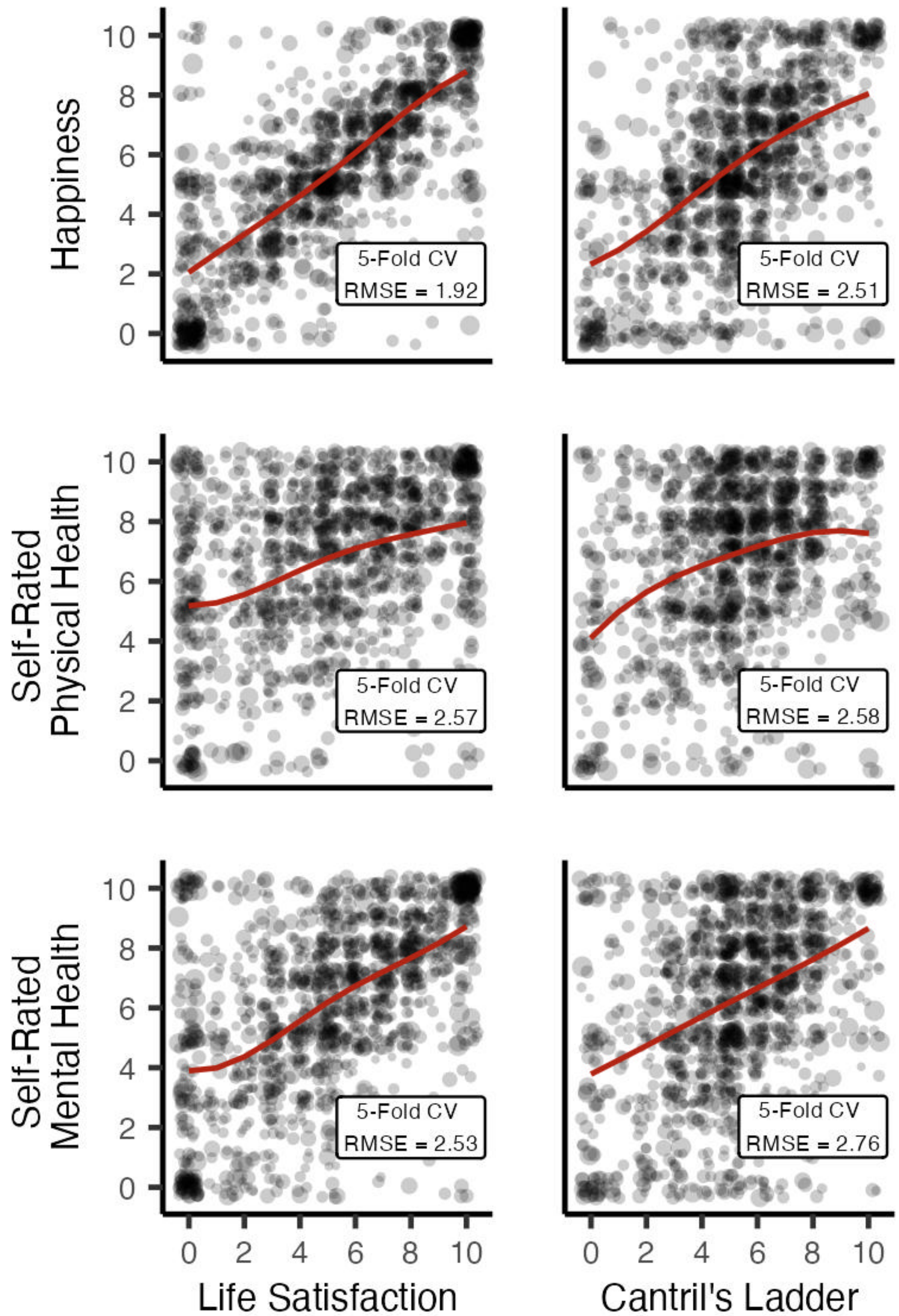

Figure 77: Scatterplots for Turkey, Panel 1. Curves fit using a piecewise polynomial regression with B-splines. Regressions incorporate survey weights, and the cross-validation procedure uses cluster sampling and survey weights to form each fold. See Section 1.1 for more details.

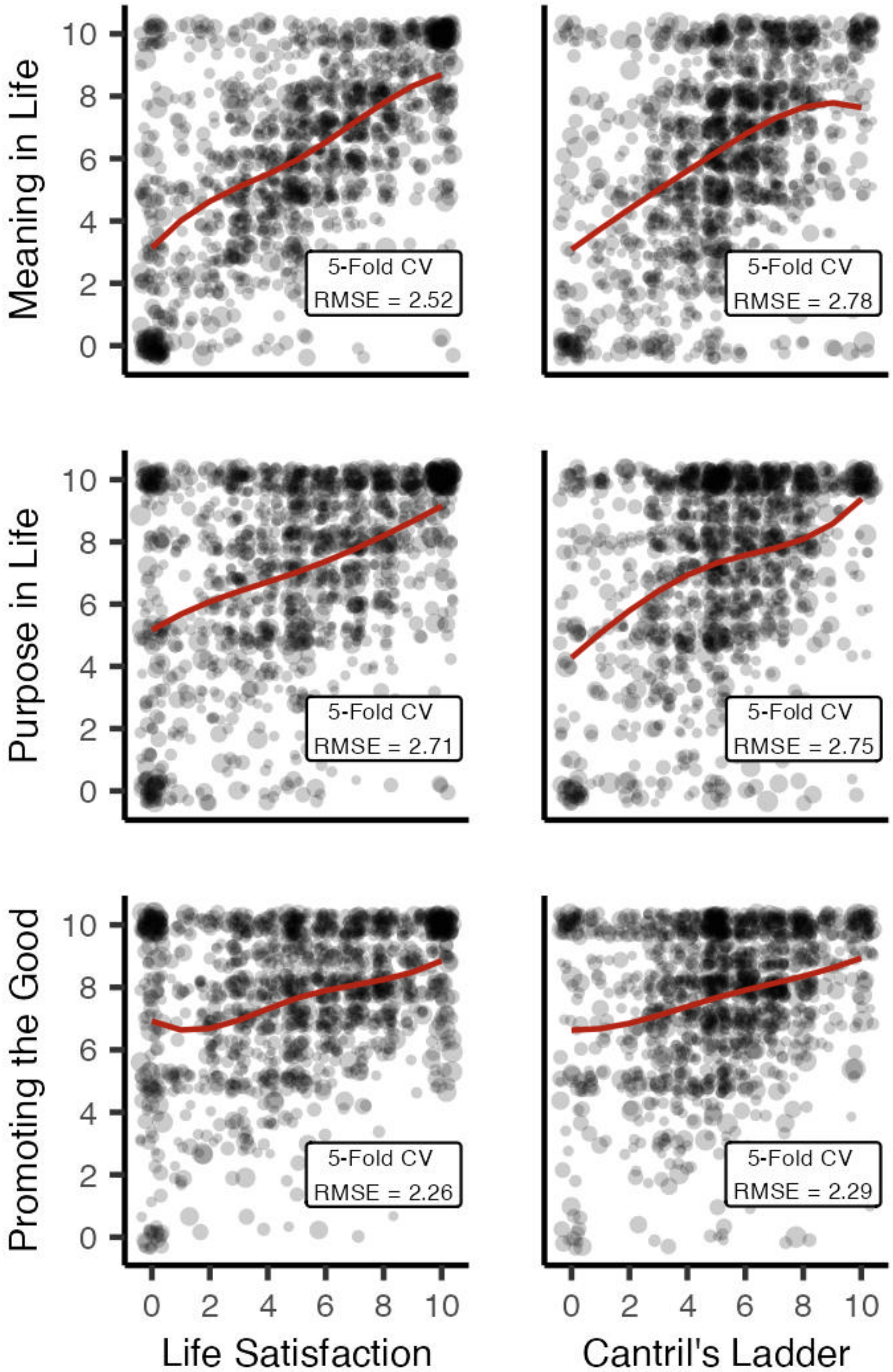

Figure 78: Scatterplots for Turkey, Panel 2. Curves fit using a piecewise polynomial regression with B-splines. Regressions incorporate survey weights, and the cross-validation procedure uses cluster sampling and survey weights to form each fold. See Section 1.1 for more details.

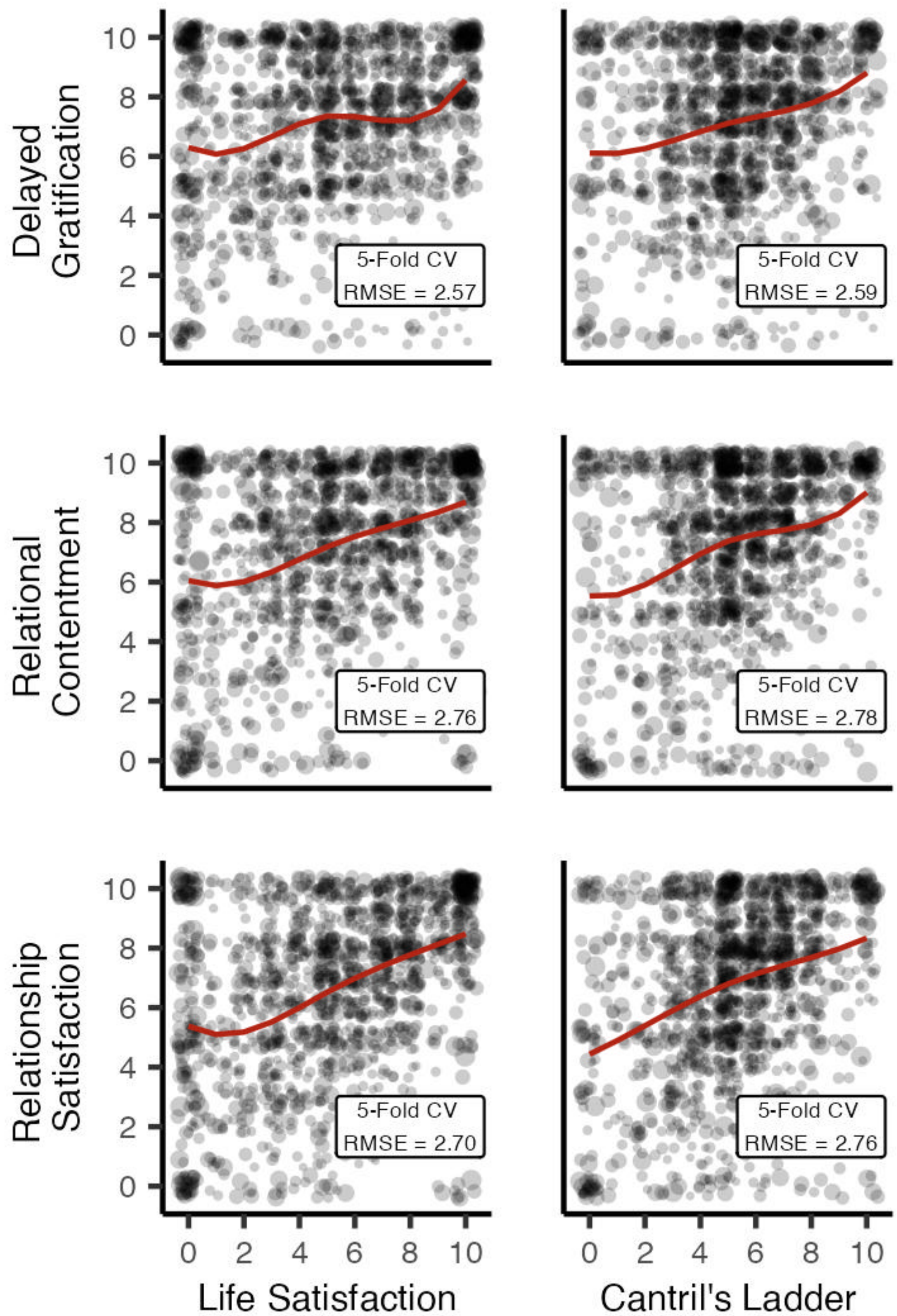

Figure 79: Scatterplots for Turkey, Panel 3. Curves fit using a piecewise polynomial regression with B-splines. Regressions incorporate survey weights, and the cross-validation procedure uses cluster sampling and survey weights to form each fold. See Section 1.1 for more details.

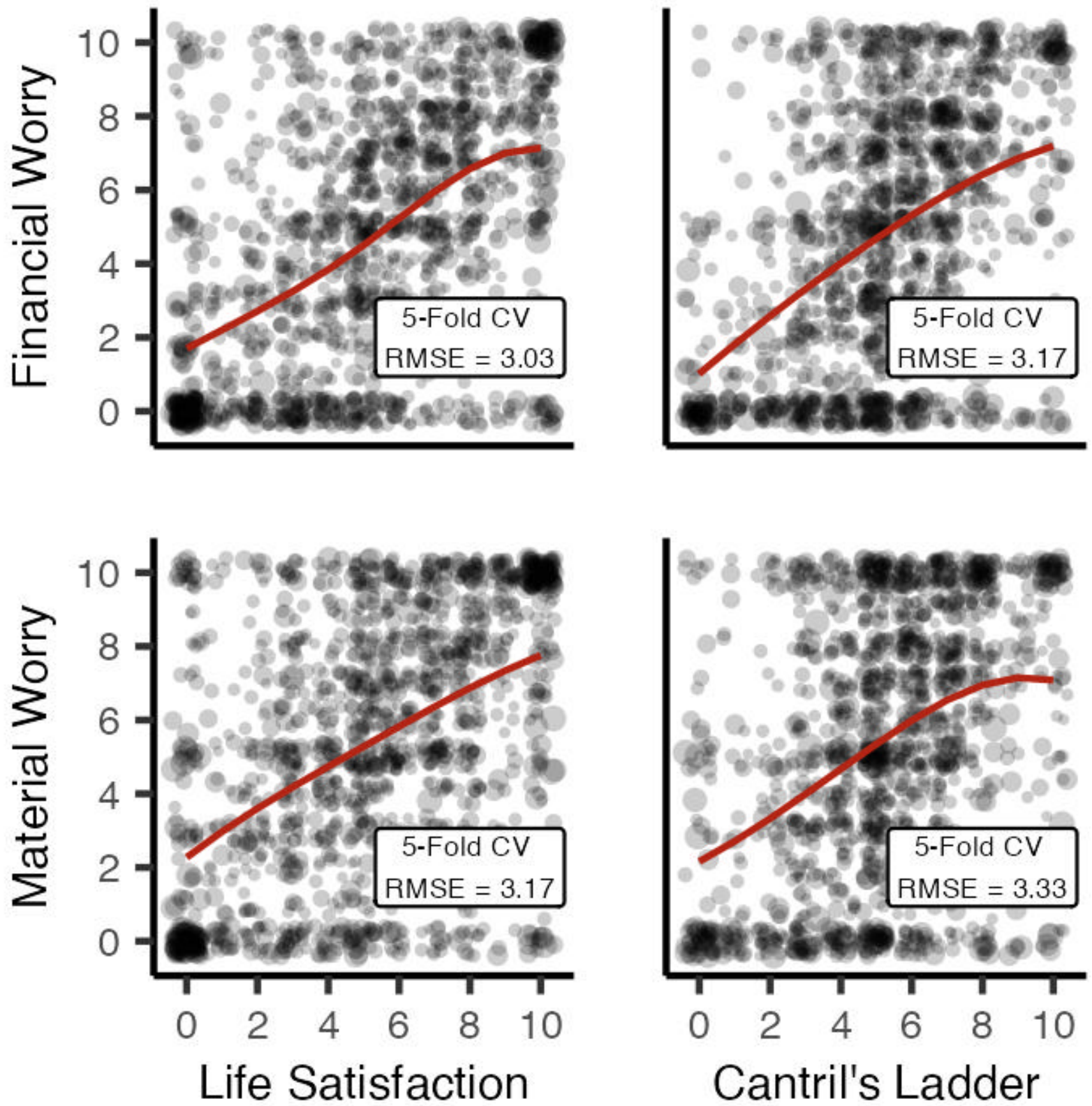

Figure 80: Scatterplots for Turkey, Panel 4. Curves fit using a piecewise polynomial regression with B-splines. Regressions incorporate survey weights, and the cross-validation procedure uses cluster sampling and survey weights to form each fold. See Section 1.1 for more details.

Section 23

Scatterplots for United Kingdom

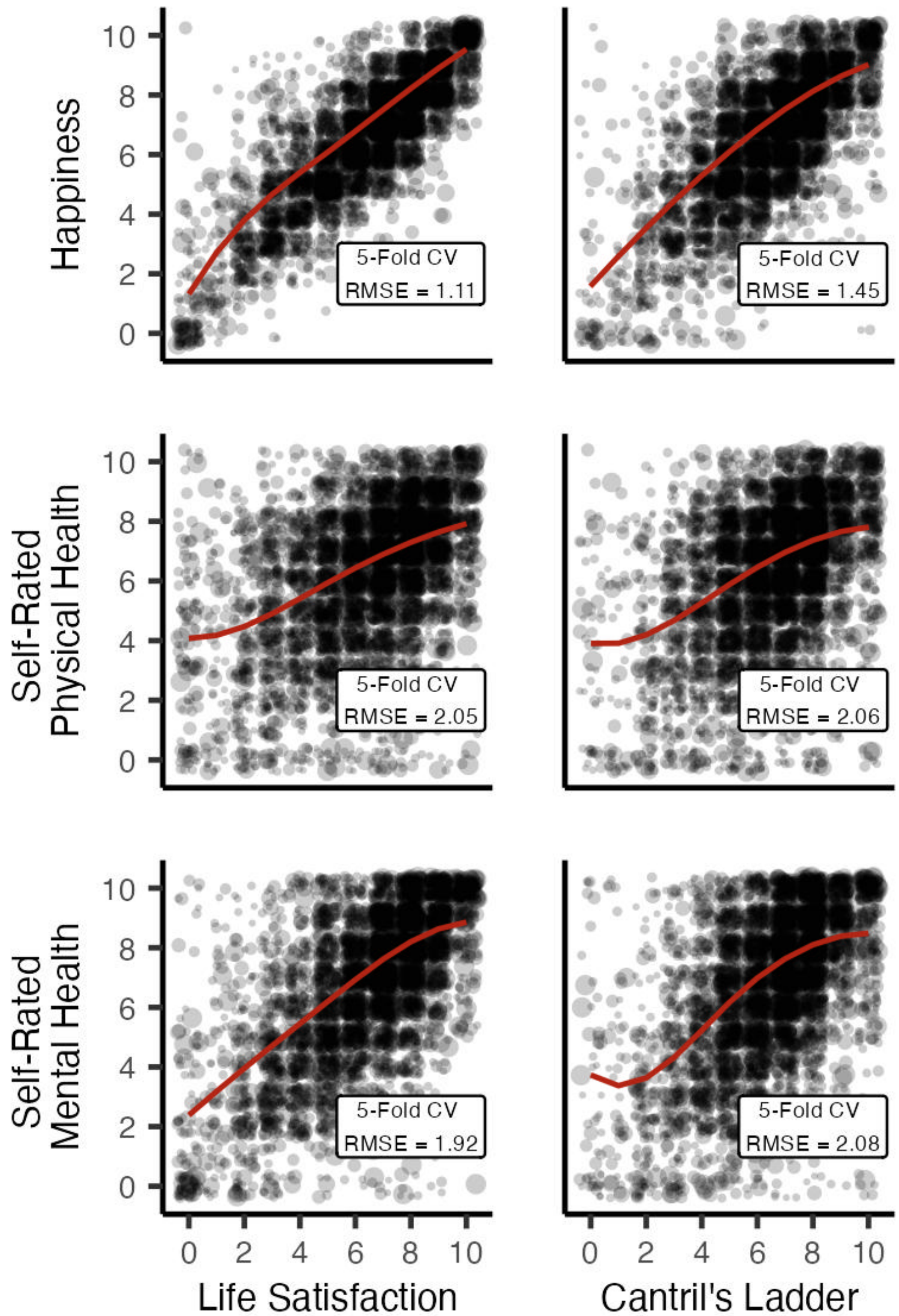

Figure 81: Scatterplots for United Kingdom, Panel 1. Curves fit using a piecewise polynomial regression with B-splines. Regressions incorporate survey weights, and the cross-validation procedure uses cluster sampling and survey weights to form each fold. See Section 1.1 for more details.

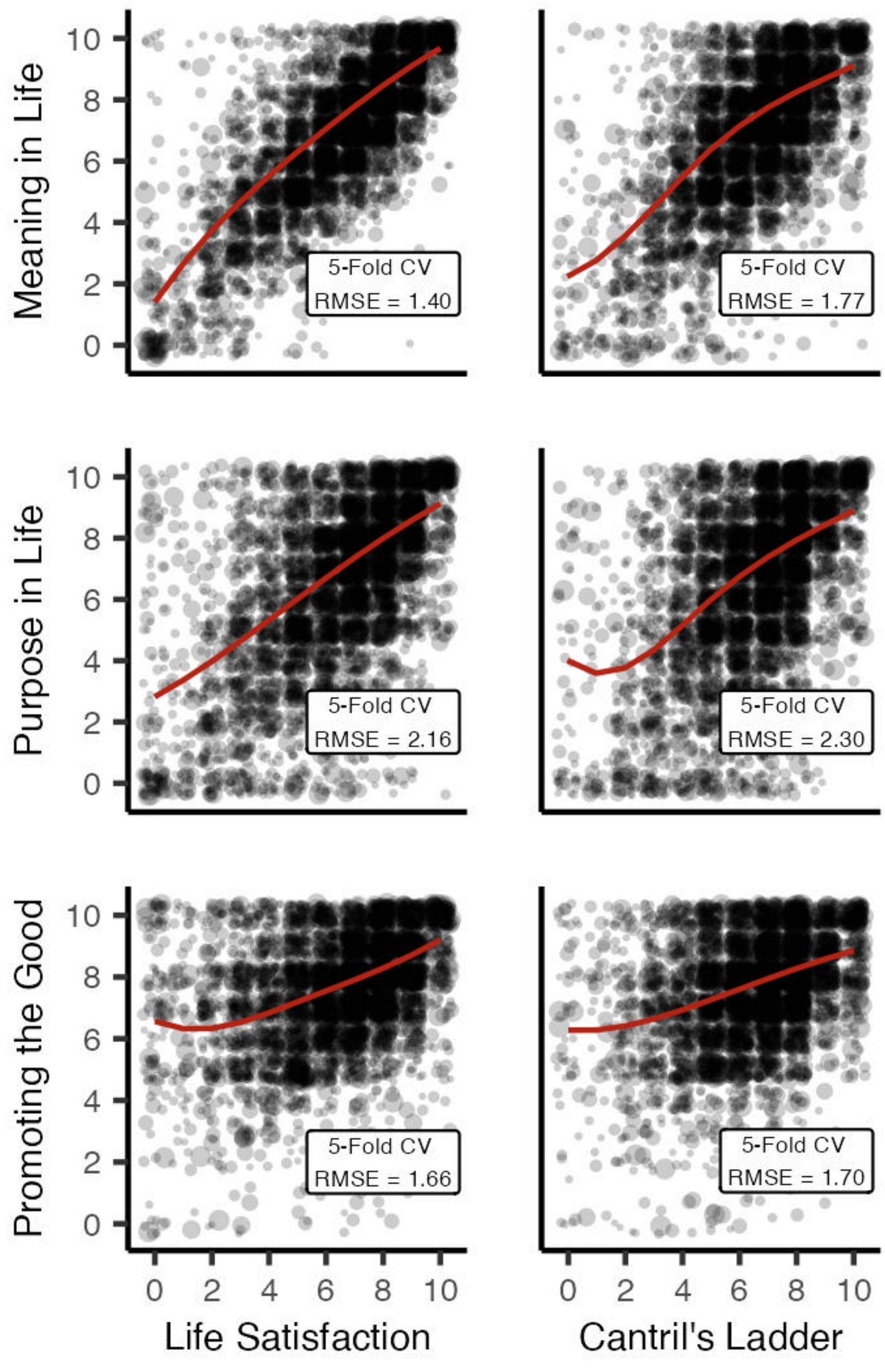

Figure 82: Scatterplots for United Kingdom, Panel 2. Curves fit using a piecewise polynomial regression with B-splines. Regressions incorporate survey weights, and the cross-validation procedure uses cluster sampling and survey weights to form each fold. See Section 1.1 for more details.

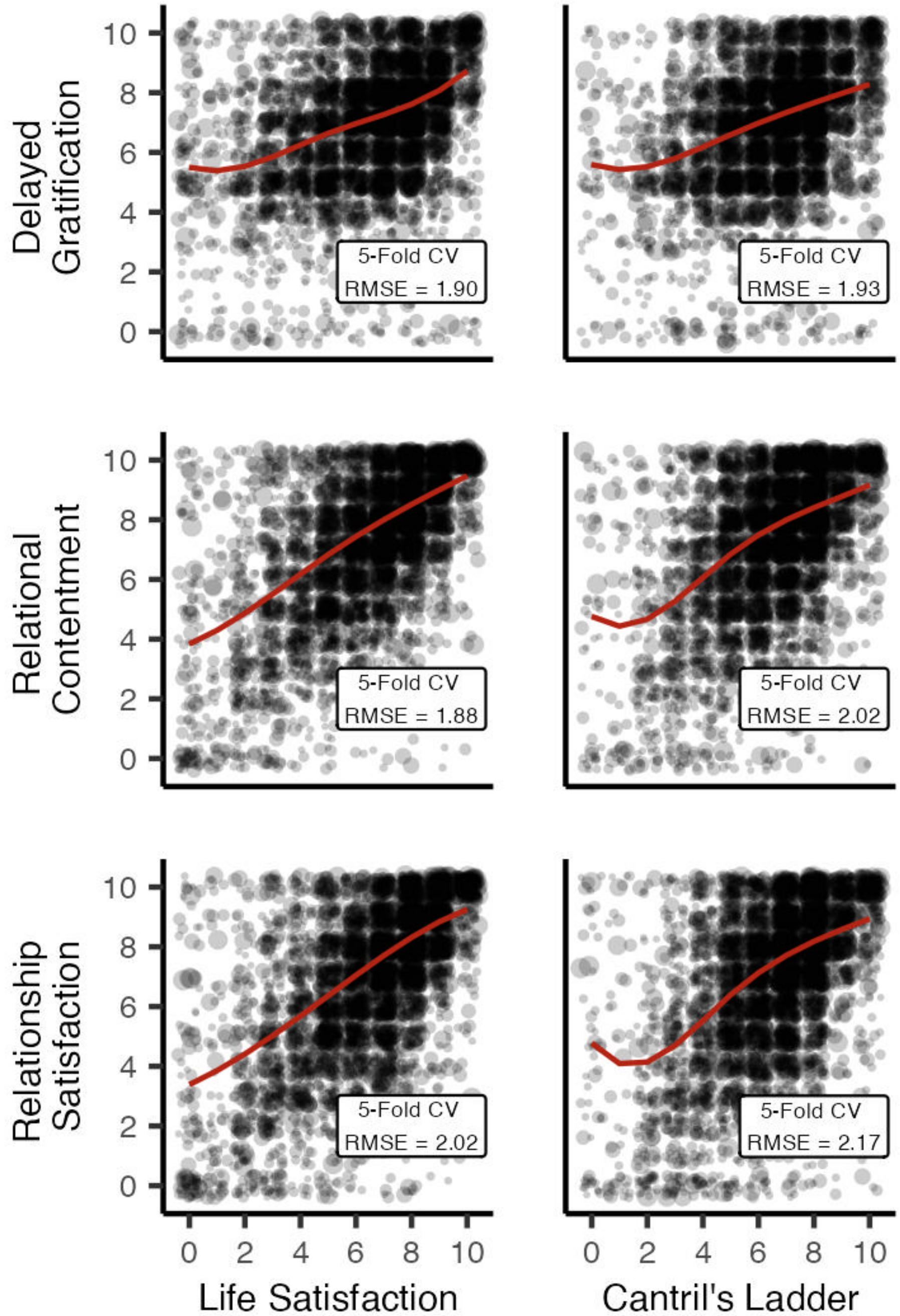

Figure 83: Scatterplots for United Kingdom, Panel 3. Curves fit using a piecewise polynomial regression with B-splines. Regressions incorporate survey weights, and the cross-validation procedure uses cluster sampling and survey weights to form each fold. See Section 1.1 for more details.

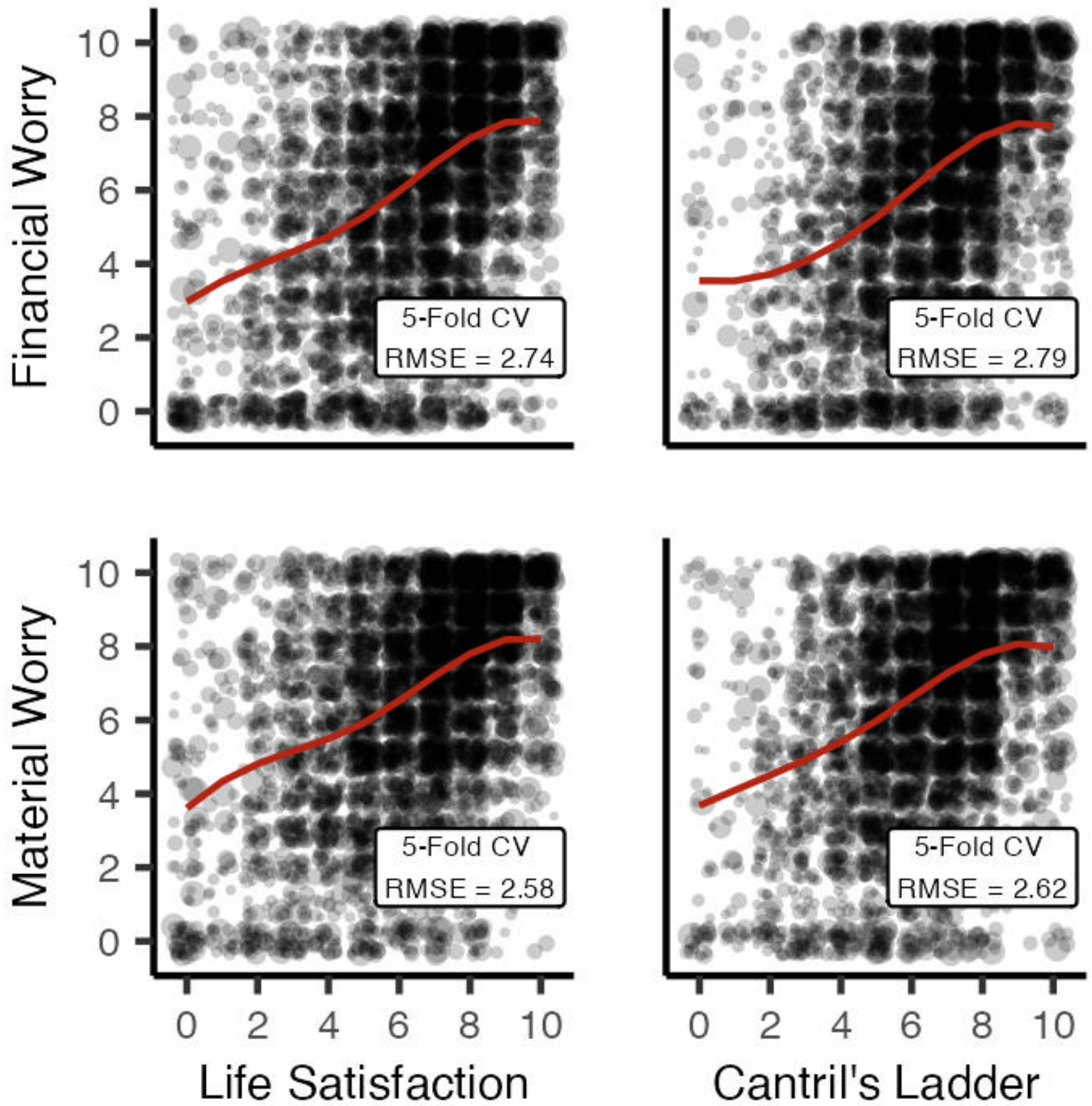

Figure 84: Scatterplots for United Kingdom, Panel 4. Curves fit using a piecewise polynomial regression with B-splines. Regressions incorporate survey weights, and the cross-validation procedure uses cluster sampling and survey weights to form each fold. See Section 1.1 for more details.

Section 24

Scatterplots for United States

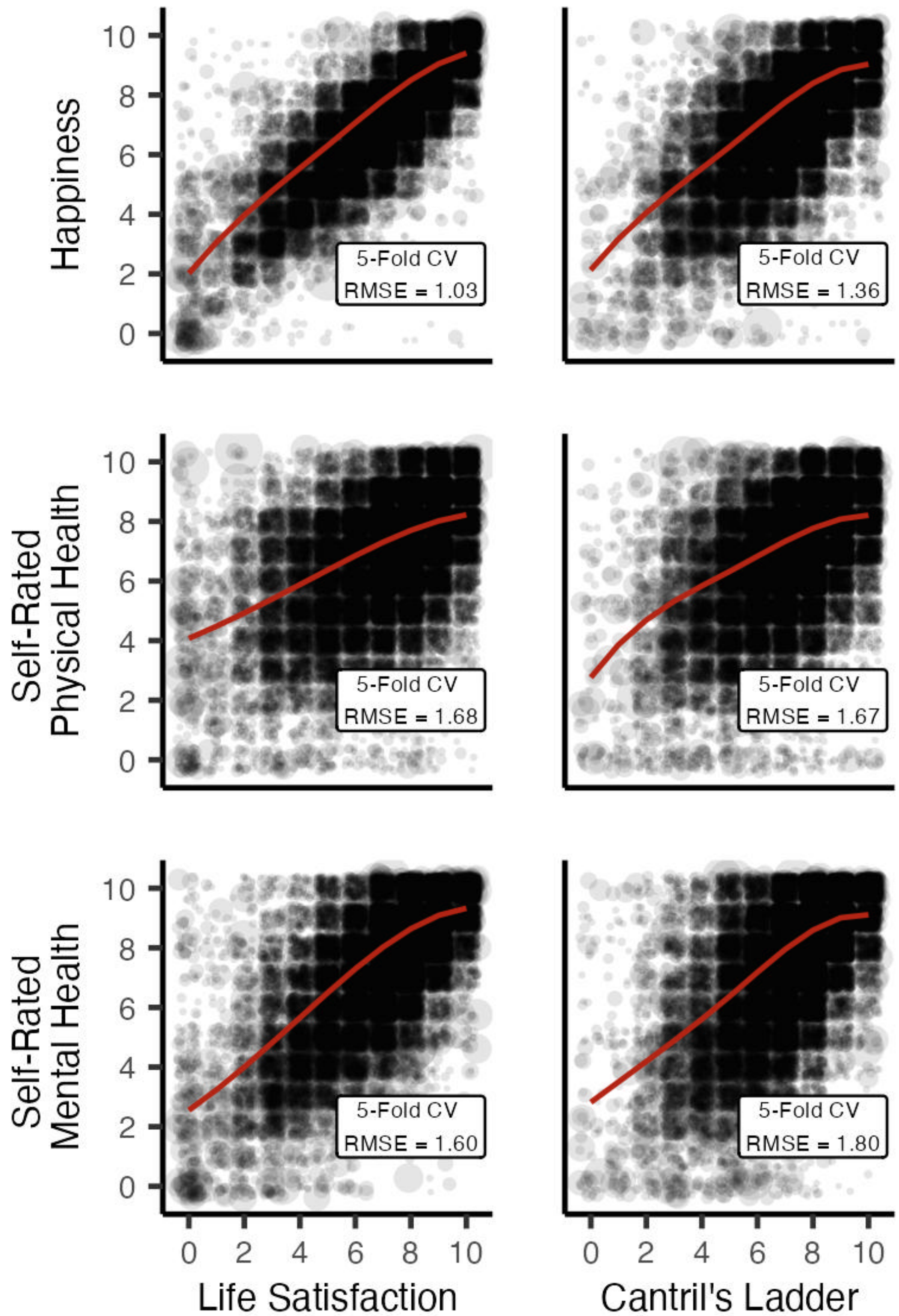

Figure 85: Scatterplots for United States, Panel 1. Curves fit using a piecewise polynomial regression with B-splines. Regressions incorporate survey weights, and the cross-validation procedure uses cluster sampling and survey weights to form each fold. See Section 1.1 for more details.

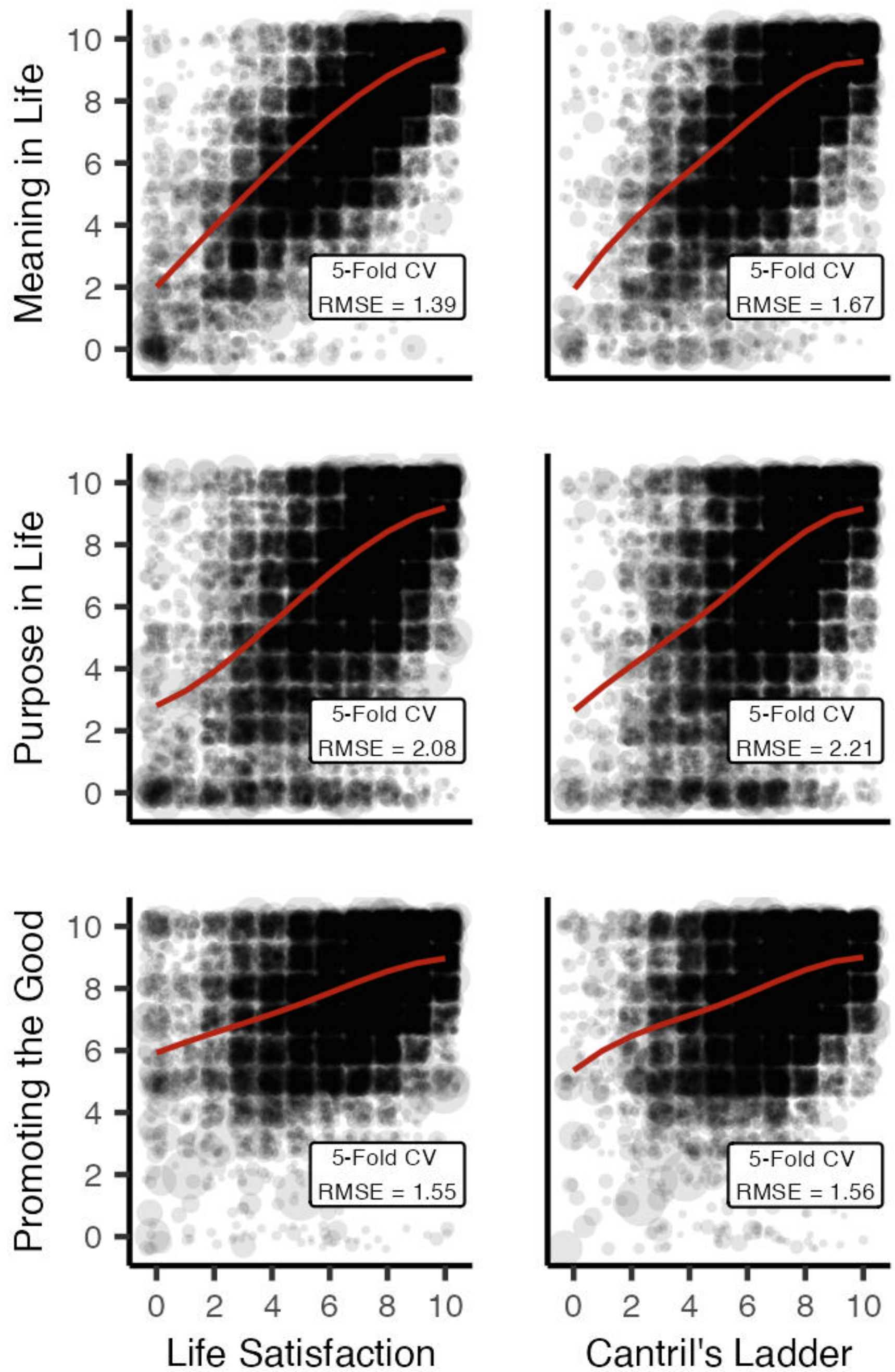

Figure 86: Scatterplots for United States, Panel 2. Curves fit using a piecewise polynomial regression with B-splines. Regressions incorporate survey weights, and the cross-validation procedure uses cluster sampling and survey weights to form each fold. See Section 1.1 for more details.

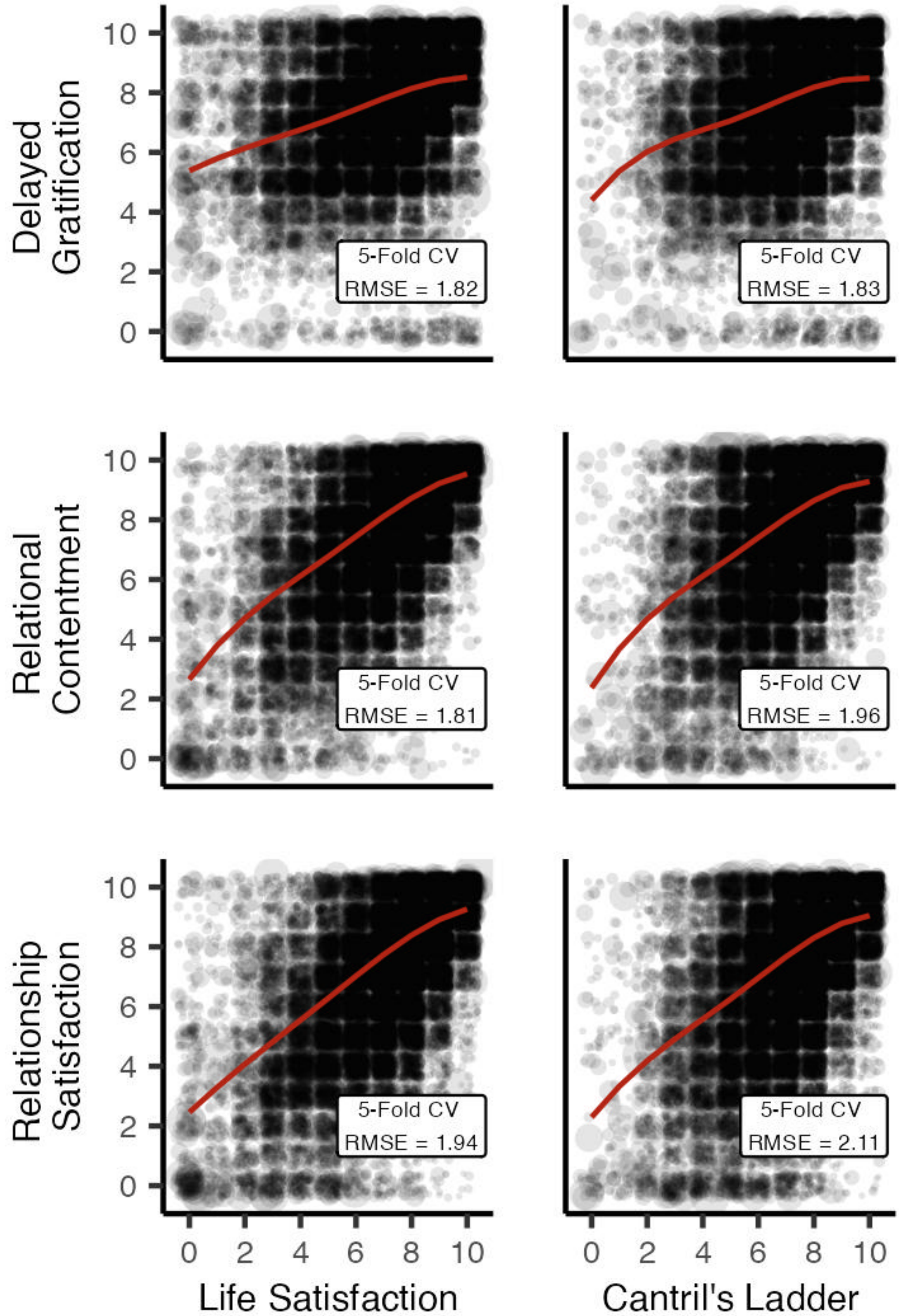

Figure 87: Scatterplots for United States, Panel 3. Curves fit using a piecewise polynomial regression with B-splines. Regressions incorporate survey weights, and the cross-validation procedure uses cluster sampling and survey weights to form each fold. See Section 1.1 for more details.

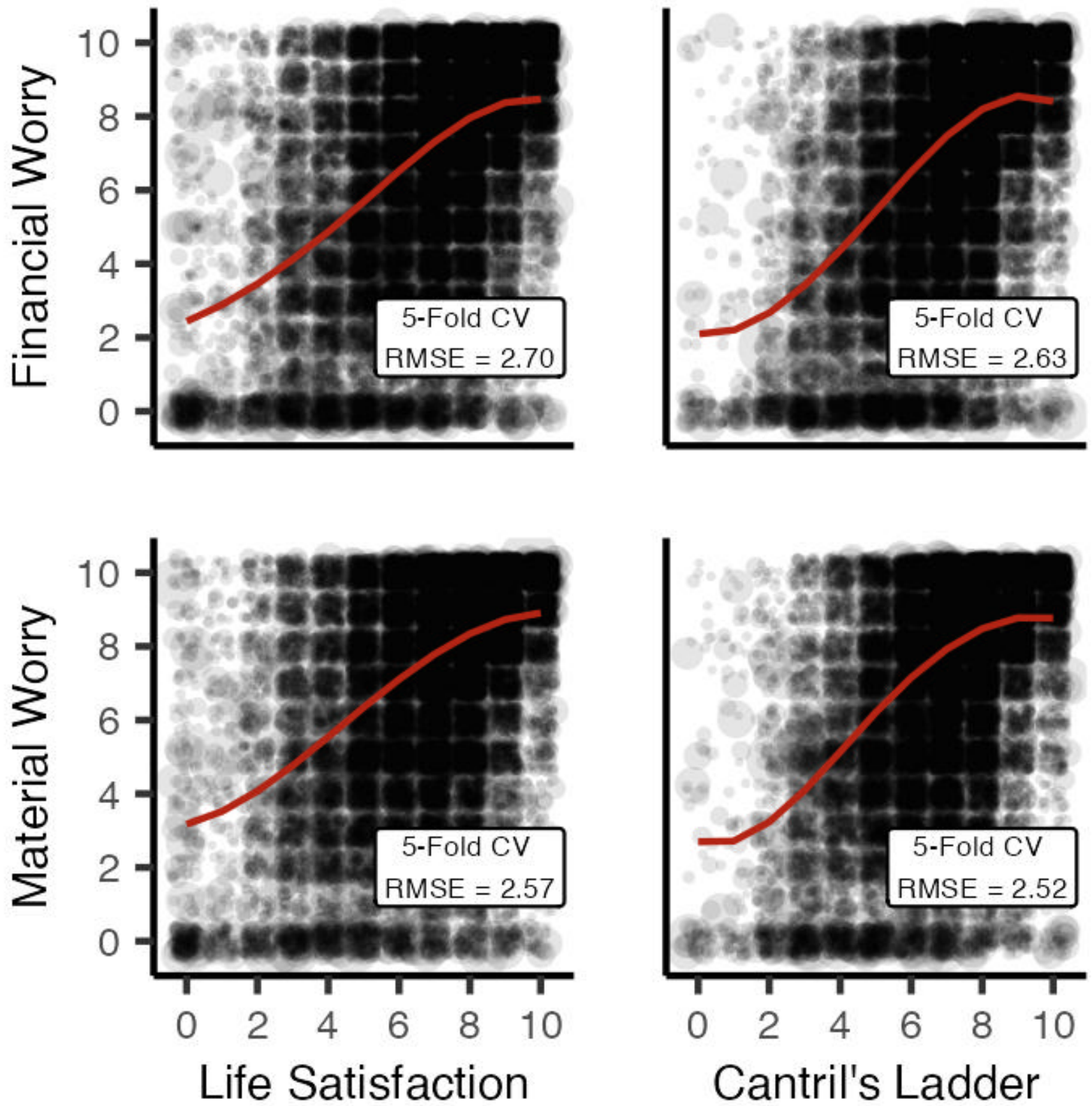

Figure 88: Scatterplots for United States, Panel 4. Curves fit using a piecewise polynomial regression with B-splines. Regressions incorporate survey weights, and the cross-validation procedure uses cluster sampling and survey weights to form each fold. See Section 1.1 for more details.

Section 25

Overfitting with `geom_smooth()`

To test the code, we initially focused on the United States and produced the grid of plots shown in Figure 89. The curve in the *Life Satisfaction–Physical Health* plot is clearly overfit.

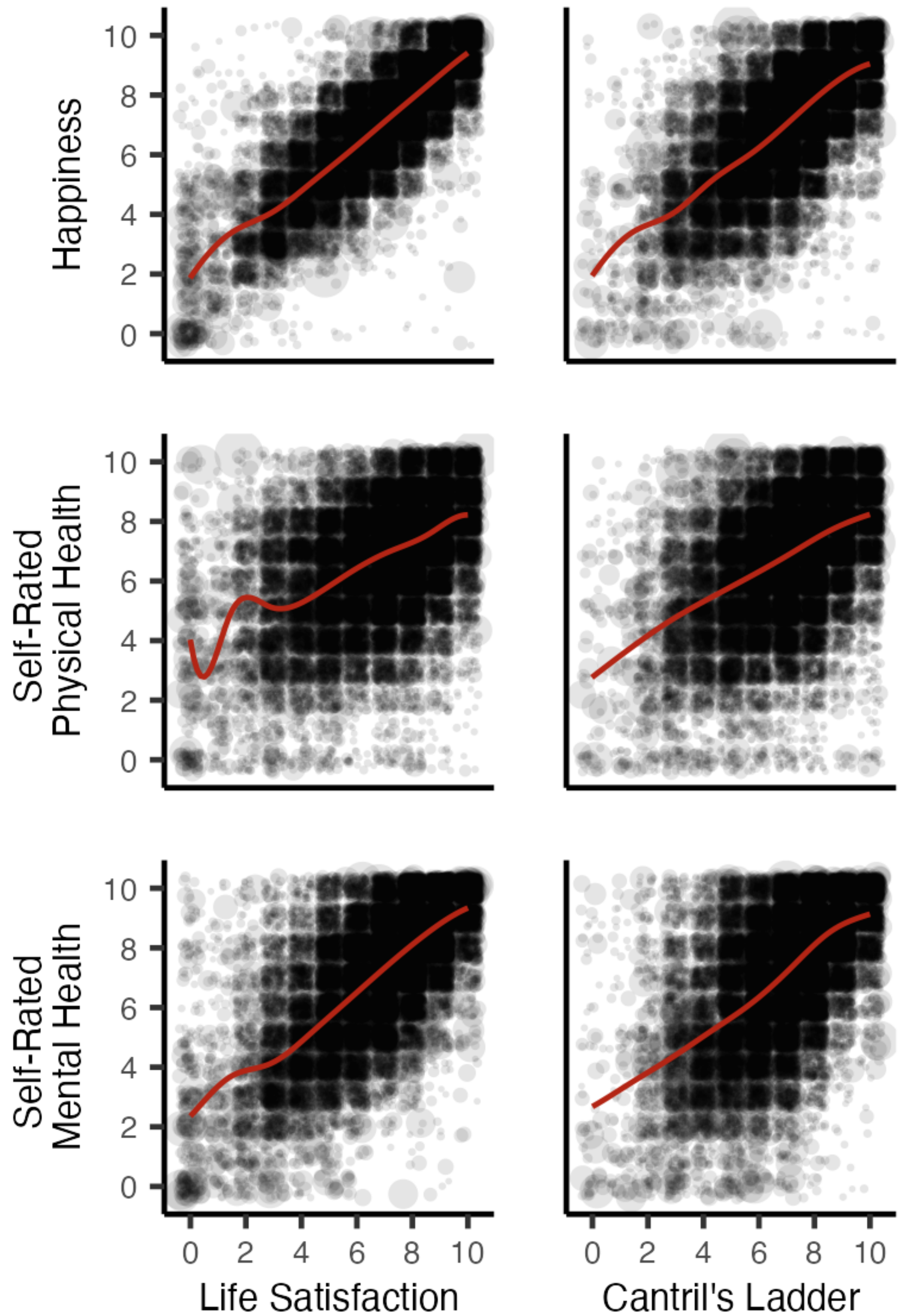

Figure 89: Test scatterplots for United States demonstrating overfitting. Curves fit using survey-weighted P-splines in `geom_smooth()`. The curve in the *LS–Physical Health* plot is clearly overfit. See Section 1.1 for more details.

References

Bates, Stephen, Trevor Hastie and Robert Tibshirani. 2024. "Cross-validation: what does it estimate and how well does it do it?" *Journal of the American Statistical Association* 119(546):1434–1445.

Lumley, Thomas. 2004. "Analysis of Complex Survey Samples." *Journal of Statistical Software* 9(1):1–19. R package verson 2.2.

Wickham, Hadley. 2016. *ggplot2: Elegant Graphics for Data Analysis*. Springer-Verlag New York.
URL: *https://ggplot2.tidyverse.org*

Wieczorek, Jerzy, Cole Guerin and Thomas McMahon. 2022. "K-fold cross-validation for complex sample surveys." *Stat* 11(1):e454.

Wood, Simon and Maintainer Simon Wood. 2015. "Package 'mgcv'." *R package version* 1(29):729.
