# Supplementary material for: Life Satisfaction is More Strongly Correlated with Flourishing Than Cantril’s Ladder
Source: Appl Res Qual Life. 2026 Jun 22;21(5):3337–48. doi: 10.1007/s11482-026-10623-5 (PMC13624039; doi:10.1007/s11482-026-10623-5)
Supplement: Supplementary file 2 [file 11482_2026_10623_MOESM2_ESM.docx]

**Online Supplement**

Table S1. Overall correlation matrix (N=197,036).

| Variable | (1) | (2) | (3) | (4) | (5) | (6) | (7) | (8) | (9) | (10) | (11) | (12) | (13) | Avg.Cor. |
| --- | --- | --- | --- | --- | --- | --- | --- | --- | --- | --- | --- | --- | --- | --- |
| Current life evaluation (1) |  | 0.57 | 0.57 | 0.37 | 0.39 | 0.46 | 0.34 | 0.28 | 0.23 | 0.35 | 0.36 | 0.34 | 0.31 | 0.38 |
| Life satisfaction (2) | 0.57 |  | 0.69 | 0.41 | 0.49 | 0.61 | 0.42 | 0.33 | 0.27 | 0.44 | 0.46 | 0.35 | 0.33 | 0.45 |
| Happiness (3) | 0.57 | 0.69 |  | 0.43 | 0.51 | 0.58 | 0.43 | 0.35 | 0.27 | 0.45 | 0.46 | 0.33 | 0.32 | 0.45 |
| Self-rated physical health (4) | 0.37 | 0.41 | 0.43 |  | 0.53 | 0.40 | 0.35 | 0.32 | 0.26 | 0.32 | 0.33 | 0.26 | 0.24 | 0.35 |
| Self-rated mental health (5) | 0.39 | 0.49 | 0.51 | 0.53 |  | 0.49 | 0.45 | 0.39 | 0.29 | 0.43 | 0.42 | 0.27 | 0.26 | 0.41 |
| Meaningful activities (6) | 0.46 | 0.61 | 0.58 | 0.40 | 0.49 |  | 0.50 | 0.40 | 0.30 | 0.44 | 0.44 | 0.28 | 0.27 | 0.43 |
| Understanding purpose (7) | 0.34 | 0.42 | 0.43 | 0.35 | 0.45 | 0.50 |  | 0.42 | 0.35 | 0.47 | 0.45 | 0.22 | 0.22 | 0.39 |
| Orientation to promote good (8) | 0.28 | 0.33 | 0.35 | 0.32 | 0.39 | 0.40 | 0.42 |  | 0.42 | 0.37 | 0.35 | 0.16 | 0.16 | 0.33 |
| Delayed gratification (9) | 0.23 | 0.27 | 0.27 | 0.26 | 0.29 | 0.30 | 0.35 | 0.42 |  | 0.29 | 0.28 | 0.13 | 0.13 | 0.27 |
| Relationship contentment (10) | 0.35 | 0.44 | 0.45 | 0.32 | 0.43 | 0.44 | 0.47 | 0.37 | 0.29 |  | 0.70 | 0.23 | 0.23 | 0.39 |
| Relationship satisfaction (11) | 0.36 | 0.46 | 0.46 | 0.33 | 0.42 | 0.44 | 0.45 | 0.35 | 0.28 | 0.70 |  | 0.25 | 0.24 | 0.40 |
| Financial security (12) | 0.34 | 0.35 | 0.33 | 0.26 | 0.27 | 0.28 | 0.22 | 0.16 | 0.13 | 0.23 | 0.25 |  | 0.70 | 0.29 |
| Material security (13) | 0.31 | 0.33 | 0.32 | 0.24 | 0.26 | 0.27 | 0.22 | 0.16 | 0.13 | 0.23 | 0.24 | 0.70 |  | 0.28 |

| ***Table 2.***  ***Average correlation of LE and LS with financial domains and other domains for all countries included in the GFS (N=197,036).*** | | | | | |
| --- | --- | --- | --- | --- | --- |
|  | Financial Domain | |  | Non-Financial Domains | |
| Country | LE | LS |  | LE | LS |
| Argentina | 0.22 (0.09) | 0.19 (0.09) |  | 0.35 (0.05) | 0.47 (0.05) |
| Australia | 0.48 (0.10) | 0.47 (0.10) |  | 0.53 (0.05) | 0.59 (0.04) |
| Brazil | 0.23 (0.08) | 0.22 (0.08) |  | 0.40 (0.04) | 0.51 (0.04) |
| Egypt | 0.30 (0.10) | 0.29 (0.10) |  | 0.21 (0.04) | 0.26 (0.05) |
| Germany | 0.36 (0.08) | 0.35 (0.08) |  | 0.35 (0.04) | 0.44 (0.04) |
| Hong Kong | 0.59 (0.10) | 0.60 (0.10) |  | 0.68 (0.05) | 0.74 (0.04) |
| India | 0.11 (0.08) | 0.15 (0.08) |  | 0.26 (0.04) | 0.37 (0.04) |
| Indonesia | 0.21 (0.10) | 0.23 (0.10) |  | 0.27 (0.05) | 0.43 (0.05) |
| Israel | 0.37 (0.13) | 0.39 (0.14) |  | 0.40 (0.06) | 0.43 (0.06) |
| Japan | 0.56 (0.06) | 0.57 (0.06) |  | 0.63 (0.03) | 0.67 (0.03) |
| Kenya | 0.24 (0.08) | 0.28 (0.08) |  | 0.17 (0.04) | 0.22 (0.04) |
| Mexico | 0.25 (0.09) | 0.19 (0.09) |  | 0.36 (0.05) | 0.48 (0.05) |
| Nigeria | 0.18 (0.10) | 0.28 (0.10) |  | 0.20 (0.05) | 0.29 (0.05) |
| Philippines | 0.18 (0.10) | 0.24 (0.10) |  | 0.23 (0.05) | 0.39 (0.05) |
| Poland | 0.32 (0.12) | 0.40 (0.10) |  | 0.37 (0.05) | 0.52 (0.05) |
| South Africa | 0.27 (0.11) | 0.31 (0.12) |  | 0.26 (0.06) | 0.32 (0.06) |
| Spain | 0.24 (0.09) | 0.24 (0.09) |  | 0.42 (0.04) | 0.47 (0.04) |
| Sweden | 0.40 (0.07) | 0.39 (0.07) |  | 0.52 (0.03) | 0.58 (0.03) |
| Tanzania | 0.29 (0.09) | 0.33 (0.09) |  | 0.17 (0.04) | 0.24 (0.04) |
| Turkey | 0.42 (0.13) | 0.51 (0.12) |  | 0.36 (0.06) | 0.43 (0.06) |
| United Kingdom | 0.39 (0.10) | 0.42 (0.10) |  | 0.49 (0.04) | 0.58 (0.04) |
| United States | 0.52 (0.08) | 0.49 (0.08) |  | 0.53 (0.04) | 0.60 (0.04) |
| *Note.* Reported in columns are average correlation (and standard error) of LE/LS with either the items in the Financial and material stability domain or all other domains. Financial Domain includes the financial and material stability indicators; Non-Financial Domains includes the Happiness, Health, Meaning and Purpose, Character and Virtue, and Relationship Domains. | | | | | |

Table S3. Survey weighted correlations of LE and LS with flourishing indicators in Argentina (N=6536)

| Variable | (1) | (2) | (3) | (4) | (5) | (6) | (7) | (8) | (9) | (10) | (11) | (12) | (13) | Avg. Cor. |
| --- | --- | --- | --- | --- | --- | --- | --- | --- | --- | --- | --- | --- | --- | --- |
| Current life evaluation (1) |  | 0.59 | 0.59 | 0.35 | 0.37 | 0.47 | 0.33 | 0.21 | 0.19 | 0.33 | 0.34 | 0.25 | 0.19 | 0.35 |
| Life satisfaction (2) | 0.59 |  | 0.73 | 0.41 | 0.49 | 0.64 | 0.47 | 0.29 | 0.25 | 0.43 | 0.48 | 0.21 | 0.17 | 0.43 |
| Happiness (3) | **0.59** | **0.73** |  | 0.40 | 0.50 | 0.62 | 0.46 | 0.29 | 0.26 | 0.42 | 0.48 | 0.19 | 0.14 | 0.42 |
| Self-rated physical health (4) | **0.35** | **0.41** | 0.40 |  | 0.48 | 0.35 | 0.27 | 0.24 | 0.20 | 0.26 | 0.32 | 0.18 | 0.14 | 0.30 |
| Self-rated mental health (5) | **0.37** | **0.49** | 0.50 | 0.48 |  | 0.49 | 0.47 | 0.36 | 0.28 | 0.39 | 0.43 | 0.17 | 0.14 | 0.38 |
| Meaningful activities (6) | **0.47** | **0.64** | 0.62 | 0.35 | 0.49 |  | 0.51 | 0.36 | 0.27 | 0.43 | 0.47 | 0.15 | 0.11 | 0.41 |
| Understanding purpose (7) | **0.33** | **0.47** | 0.46 | 0.27 | 0.47 | 0.51 |  | 0.39 | 0.32 | 0.40 | 0.42 | 0.12 | 0.08 | 0.35 |
| Orientation to promote good (8) | **0.21** | **0.29** | 0.29 | 0.24 | 0.36 | 0.36 | 0.39 |  | 0.36 | 0.28 | 0.29 | 0.01 | -0.01 | 0.26 |
| Delayed gratification (9) | **0.19** | **0.25** | 0.26 | 0.20 | 0.28 | 0.27 | 0.32 | 0.36 |  | 0.24 | 0.25 | 0.01 | -0.01 | 0.22 |
| Relationship contentment (10) | **0.33** | **0.43** | 0.42 | 0.26 | 0.39 | 0.43 | 0.40 | 0.28 | 0.24 |  | 0.67 | 0.13 | 0.12 | 0.34 |
| Relationship satisfaction (11) | **0.34** | **0.48** | 0.48 | 0.32 | 0.43 | 0.47 | 0.42 | 0.29 | 0.25 | 0.67 |  | 0.15 | 0.12 | 0.37 |
| Financial security (12) | **0.25** | **0.21** | 0.19 | 0.18 | 0.17 | 0.15 | 0.12 | 0.01 | 0.01 | 0.13 | 0.15 |  | 0.79 | 0.20 |
| Material security (13) | **0.19** | **0.17** | 0.14 | 0.14 | 0.14 | 0.11 | 0.08 | -0.01 | -0.01 | 0.12 | 0.12 | 0.79 |  | 0.17 |

Table S4. Survey weighted correlations of LE and LS with flourishing indicators in Australia (N=3791)

| Variable | (1) | (2) | (3) | (4) | (5) | (6) | (7) | (8) | (9) | (10) | (11) | (12) | (13) | Avg. Cor. |
| --- | --- | --- | --- | --- | --- | --- | --- | --- | --- | --- | --- | --- | --- | --- |
| Current life evaluation (1) |  | 0.77 | 0.73 | 0.48 | 0.65 | 0.64 | 0.52 | 0.33 | 0.30 | 0.55 | 0.54 | 0.48 | 0.48 | 0.54 |
| Life satisfaction (2) | 0.77 |  | 0.82 | 0.47 | 0.74 | 0.75 | 0.60 | 0.37 | 0.33 | 0.62 | 0.63 | 0.47 | 0.48 | 0.59 |
| Happiness (3) | **0.73** | **0.82** |  | 0.46 | 0.78 | 0.72 | 0.57 | 0.39 | 0.31 | 0.64 | 0.62 | 0.44 | 0.46 | 0.58 |
| Self-rated physical health (4) | **0.48** | **0.47** | 0.46 |  | 0.51 | 0.45 | 0.37 | 0.27 | 0.30 | 0.33 | 0.33 | 0.35 | 0.36 | 0.39 |
| Self-rated mental health (5) | **0.65** | **0.74** | 0.78 | 0.51 |  | 0.66 | 0.59 | 0.42 | 0.33 | 0.60 | 0.59 | 0.47 | 0.46 | 0.57 |
| Meaningful activities (6) | **0.64** | **0.75** | 0.72 | 0.45 | 0.66 |  | 0.65 | 0.42 | 0.34 | 0.59 | 0.59 | 0.36 | 0.39 | 0.55 |
| Understanding purpose (7) | **0.52** | **0.60** | 0.57 | 0.37 | 0.59 | 0.65 |  | 0.42 | 0.35 | 0.55 | 0.52 | 0.35 | 0.36 | 0.49 |
| Orientation to promote good (8) | **0.33** | **0.37** | 0.39 | 0.27 | 0.42 | 0.42 | 0.42 |  | 0.41 | 0.36 | 0.36 | 0.22 | 0.23 | 0.35 |
| Delayed gratification (9) | **0.30** | **0.33** | 0.31 | 0.30 | 0.33 | 0.34 | 0.35 | 0.41 |  | 0.29 | 0.28 | 0.19 | 0.21 | 0.30 |
| Relationship contentment (10) | **0.55** | **0.62** | 0.64 | 0.33 | 0.60 | 0.59 | 0.55 | 0.36 | 0.29 |  | 0.87 | 0.33 | 0.35 | 0.51 |
| Relationship satisfaction (11) | **0.54** | **0.63** | 0.62 | 0.33 | 0.59 | 0.59 | 0.52 | 0.36 | 0.28 | 0.87 |  | 0.33 | 0.36 | 0.50 |
| Financial security (12) | **0.48** | **0.47** | 0.44 | 0.35 | 0.47 | 0.36 | 0.35 | 0.22 | 0.19 | 0.33 | 0.33 |  | 0.78 | 0.40 |
| Material security (13) | **0.48** | **0.48** | 0.46 | 0.36 | 0.46 | 0.39 | 0.36 | 0.23 | 0.21 | 0.35 | 0.36 | 0.78 |  | 0.41 |

Table S5. Survey weighted correlations of LE and LS with flourishing indicators in Brazil (N=12871)

| Variable | (1) | (2) | (3) | (4) | (5) | (6) | (7) | (8) | (9) | (10) | (11) | (12) | (13) | Avg. Cor. |
| --- | --- | --- | --- | --- | --- | --- | --- | --- | --- | --- | --- | --- | --- | --- |
| Current life evaluation (1) |  | 0.62 | 0.59 | 0.39 | 0.42 | 0.51 | 0.40 | 0.26 | 0.22 | 0.42 | 0.42 | 0.25 | 0.22 | 0.39 |
| Life satisfaction (2) | 0.62 |  | 0.74 | 0.47 | 0.56 | 0.69 | 0.52 | 0.34 | 0.26 | 0.52 | 0.53 | 0.25 | 0.20 | 0.47 |
| Happiness (3) | **0.59** | **0.74** |  | 0.45 | 0.55 | 0.65 | 0.51 | 0.35 | 0.27 | 0.53 | 0.53 | 0.21 | 0.18 | 0.46 |
| Self-rated physical health (4) | **0.39** | **0.47** | 0.45 |  | 0.52 | 0.44 | 0.38 | 0.30 | 0.23 | 0.35 | 0.36 | 0.19 | 0.15 | 0.35 |
| Self-rated mental health (5) | **0.42** | **0.56** | 0.55 | 0.52 |  | 0.55 | 0.52 | 0.39 | 0.28 | 0.47 | 0.47 | 0.21 | 0.18 | 0.43 |
| Meaningful activities (6) | **0.51** | **0.69** | 0.65 | 0.44 | 0.55 |  | 0.56 | 0.38 | 0.31 | 0.52 | 0.52 | 0.19 | 0.16 | 0.46 |
| Understanding purpose (7) | **0.40** | **0.52** | 0.51 | 0.38 | 0.52 | 0.56 |  | 0.44 | 0.36 | 0.49 | 0.48 | 0.17 | 0.11 | 0.41 |
| Orientation to promote good (8) | **0.26** | **0.34** | 0.35 | 0.30 | 0.39 | 0.38 | 0.44 |  | 0.46 | 0.35 | 0.31 | 0.03 | -0.01 | 0.30 |
| Delayed gratification (9) | **0.22** | **0.26** | 0.27 | 0.23 | 0.28 | 0.31 | 0.36 | 0.46 |  | 0.30 | 0.27 | 0.04 | 0.00 | 0.25 |
| Relationship contentment (10) | **0.42** | **0.52** | 0.53 | 0.35 | 0.47 | 0.52 | 0.49 | 0.35 | 0.30 |  | 0.76 | 0.16 | 0.13 | 0.42 |
| Relationship satisfaction (11) | **0.42** | **0.53** | 0.53 | 0.36 | 0.47 | 0.52 | 0.48 | 0.31 | 0.27 | 0.76 |  | 0.18 | 0.15 | 0.41 |
| Financial security (12) | **0.25** | **0.25** | 0.21 | 0.19 | 0.21 | 0.19 | 0.17 | 0.03 | 0.04 | 0.16 | 0.18 |  | 0.76 | 0.22 |
| Material security (13) | **0.22** | **0.20** | 0.18 | 0.15 | 0.18 | 0.16 | 0.11 | -0.01 | 0.00 | 0.13 | 0.15 | 0.76 |  | 0.19 |

Table S6. Survey weighted correlations of LE and LS with flourishing indicators in Egypt (N=4513)

| Variable | (1) | (2) | (3) | (4) | (5) | (6) | (7) | (8) | (9) | (10) | (11) | (12) | (13) | Avg. Cor. |
| --- | --- | --- | --- | --- | --- | --- | --- | --- | --- | --- | --- | --- | --- | --- |
| Current life evaluation (1) |  | 0.30 | 0.44 | 0.26 | 0.16 | 0.24 | 0.20 | 0.11 | 0.10 | 0.17 | 0.18 | 0.31 | 0.30 | 0.23 |
| Life satisfaction (2) | 0.30 |  | 0.43 | 0.27 | 0.24 | 0.38 | 0.26 | 0.18 | 0.15 | 0.24 | 0.22 | 0.28 | 0.29 | 0.27 |
| Happiness (3) | **0.44** | **0.43** |  | 0.34 | 0.25 | 0.31 | 0.24 | 0.13 | 0.14 | 0.26 | 0.26 | 0.33 | 0.33 | 0.29 |
| Self-rated physical health (4) | **0.26** | **0.27** | 0.34 |  | 0.27 | 0.21 | 0.21 | 0.13 | 0.15 | 0.21 | 0.21 | 0.28 | 0.26 | 0.23 |
| Self-rated mental health (5) | **0.16** | **0.24** | 0.25 | 0.27 |  | 0.26 | 0.29 | 0.26 | 0.17 | 0.27 | 0.21 | 0.14 | 0.16 | 0.22 |
| Meaningful activities (6) | **0.24** | **0.38** | 0.31 | 0.21 | 0.26 |  | 0.36 | 0.23 | 0.17 | 0.27 | 0.25 | 0.20 | 0.19 | 0.26 |
| Understanding purpose (7) | **0.20** | **0.26** | 0.24 | 0.21 | 0.29 | 0.36 |  | 0.25 | 0.20 | 0.33 | 0.32 | 0.18 | 0.19 | 0.25 |
| Orientation to promote good (8) | **0.11** | **0.18** | 0.13 | 0.13 | 0.26 | 0.23 | 0.25 |  | 0.22 | 0.20 | 0.20 | 0.08 | 0.09 | 0.17 |
| Delayed gratification (9) | **0.10** | **0.15** | 0.14 | 0.15 | 0.17 | 0.17 | 0.20 | 0.22 |  | 0.16 | 0.13 | 0.07 | 0.11 | 0.15 |
| Relationship contentment (10) | **0.17** | **0.24** | 0.26 | 0.21 | 0.27 | 0.27 | 0.33 | 0.20 | 0.16 |  | 0.52 | 0.22 | 0.20 | 0.25 |
| Relationship satisfaction (11) | **0.18** | **0.22** | 0.26 | 0.21 | 0.21 | 0.25 | 0.32 | 0.20 | 0.13 | 0.52 |  | 0.23 | 0.21 | 0.24 |
| Financial security (12) | **0.31** | **0.28** | 0.33 | 0.28 | 0.14 | 0.20 | 0.18 | 0.08 | 0.07 | 0.22 | 0.23 |  | 0.62 | 0.25 |
| Material security (13) | **0.30** | **0.29** | 0.33 | 0.26 | 0.16 | 0.19 | 0.19 | 0.09 | 0.11 | 0.20 | 0.21 | 0.62 |  | 0.25 |

Table S7. Survey weighted correlations of LE and LS with flourishing indicators in Germany (N=9359)

| Variable | (1) | (2) | (3) | (4) | (5) | (6) | (7) | (8) | (9) | (10) | (11) | (12) | (13) | Avg. Cor. |
| --- | --- | --- | --- | --- | --- | --- | --- | --- | --- | --- | --- | --- | --- | --- |
| Current life evaluation (1) |  | 0.66 | 0.68 | 0.37 | 0.38 | 0.47 | 0.30 | 0.18 | 0.19 | 0.30 | 0.33 | 0.37 | 0.34 | 0.38 |
| Life satisfaction (2) | 0.66 |  | 0.83 | 0.36 | 0.48 | 0.67 | 0.38 | 0.21 | 0.20 | 0.40 | 0.43 | 0.36 | 0.34 | 0.44 |
| Happiness (3) | **0.68** | **0.83** |  | 0.35 | 0.51 | 0.61 | 0.39 | 0.23 | 0.20 | 0.40 | 0.45 | 0.33 | 0.32 | 0.44 |
| Self-rated physical health (4) | **0.37** | **0.36** | 0.35 |  | 0.58 | 0.25 | 0.22 | 0.14 | 0.17 | 0.21 | 0.24 | 0.27 | 0.25 | 0.28 |
| Self-rated mental health (5) | **0.38** | **0.48** | 0.51 | 0.58 |  | 0.39 | 0.38 | 0.23 | 0.18 | 0.35 | 0.37 | 0.31 | 0.29 | 0.37 |
| Meaningful activities (6) | **0.47** | **0.67** | 0.61 | 0.25 | 0.39 |  | 0.45 | 0.28 | 0.21 | 0.38 | 0.38 | 0.24 | 0.26 | 0.38 |
| Understanding purpose (7) | **0.30** | **0.38** | 0.39 | 0.22 | 0.38 | 0.45 |  | 0.30 | 0.24 | 0.37 | 0.35 | 0.21 | 0.21 | 0.32 |
| Orientation to promote good (8) | **0.18** | **0.21** | 0.23 | 0.14 | 0.23 | 0.28 | 0.30 |  | 0.26 | 0.23 | 0.21 | 0.11 | 0.13 | 0.21 |
| Delayed gratification (9) | **0.19** | **0.20** | 0.20 | 0.17 | 0.18 | 0.21 | 0.24 | 0.26 |  | 0.17 | 0.18 | 0.11 | 0.11 | 0.18 |
| Relationship contentment (10) | **0.30** | **0.40** | 0.40 | 0.21 | 0.35 | 0.38 | 0.37 | 0.23 | 0.17 |  | 0.80 | 0.19 | 0.21 | 0.33 |
| Relationship satisfaction (11) | **0.33** | **0.43** | 0.45 | 0.24 | 0.37 | 0.38 | 0.35 | 0.21 | 0.18 | 0.80 |  | 0.20 | 0.20 | 0.35 |
| Financial security (12) | **0.37** | **0.36** | 0.33 | 0.27 | 0.31 | 0.24 | 0.21 | 0.11 | 0.11 | 0.19 | 0.20 |  | 0.75 | 0.29 |
| Material security (13) | **0.34** | **0.34** | 0.32 | 0.25 | 0.29 | 0.26 | 0.21 | 0.13 | 0.11 | 0.21 | 0.20 | 0.75 |  | 0.28 |

Table S8. Survey weighted correlations of LE and LS with flourishing indicators in Hong Kong (N=2845)

| Variable | (1) | (2) | (3) | (4) | (5) | (6) | (7) | (8) | (9) | (10) | (11) | (12) | (13) | Avg. Cor. |
| --- | --- | --- | --- | --- | --- | --- | --- | --- | --- | --- | --- | --- | --- | --- |
| Current life evaluation (1) |  | 0.78 | 0.80 | 0.72 | 0.67 | 0.72 | 0.66 | 0.70 | 0.57 | 0.64 | 0.63 | 0.63 | 0.55 | 0.67 |
| Life satisfaction (2) | 0.78 |  | 0.85 | 0.73 | 0.77 | 0.80 | 0.70 | 0.72 | 0.61 | 0.72 | 0.72 | 0.63 | 0.58 | 0.72 |
| Happiness (3) | **0.80** | **0.85** |  | 0.77 | 0.79 | 0.82 | 0.72 | 0.77 | 0.63 | 0.72 | 0.72 | 0.63 | 0.56 | 0.73 |
| Self-rated physical health (4) | **0.72** | **0.73** | 0.77 |  | 0.75 | 0.71 | 0.68 | 0.73 | 0.61 | 0.67 | 0.68 | 0.58 | 0.52 | 0.68 |
| Self-rated mental health (5) | **0.67** | **0.77** | 0.79 | 0.75 |  | 0.74 | 0.67 | 0.72 | 0.61 | 0.73 | 0.73 | 0.58 | 0.54 | 0.69 |
| Meaningful activities (6) | **0.72** | **0.80** | 0.82 | 0.71 | 0.74 |  | 0.72 | 0.75 | 0.65 | 0.71 | 0.71 | 0.58 | 0.55 | 0.71 |
| Understanding purpose (7) | **0.66** | **0.70** | 0.72 | 0.68 | 0.67 | 0.72 |  | 0.75 | 0.64 | 0.70 | 0.70 | 0.56 | 0.49 | 0.67 |
| Orientation to promote good (8) | **0.70** | **0.72** | 0.77 | 0.73 | 0.72 | 0.75 | 0.75 |  | 0.71 | 0.69 | 0.69 | 0.56 | 0.50 | 0.69 |
| Delayed gratification (9) | **0.57** | **0.61** | 0.63 | 0.61 | 0.61 | 0.65 | 0.64 | 0.71 |  | 0.60 | 0.59 | 0.47 | 0.45 | 0.59 |
| Relationship contentment (10) | **0.64** | **0.72** | 0.72 | 0.67 | 0.73 | 0.71 | 0.70 | 0.69 | 0.60 |  | 0.84 | 0.55 | 0.52 | 0.67 |
| Relationship satisfaction (11) | **0.63** | **0.72** | 0.72 | 0.68 | 0.73 | 0.71 | 0.70 | 0.69 | 0.59 | 0.84 |  | 0.53 | 0.50 | 0.67 |
| Financial security (12) | **0.63** | **0.63** | 0.63 | 0.58 | 0.58 | 0.58 | 0.56 | 0.56 | 0.47 | 0.55 | 0.53 |  | 0.75 | 0.59 |
| Material security (13) | **0.55** | **0.58** | 0.56 | 0.52 | 0.54 | 0.55 | 0.49 | 0.50 | 0.45 | 0.52 | 0.50 | 0.75 |  | 0.54 |

Table S9. Survey weighted correlations of LE and LS with flourishing indicators in India (N=12195)

| Variable | (1) | (2) | (3) | (4) | (5) | (6) | (7) | (8) | (9) | (10) | (11) | (12) | (13) | Avg. Cor. |
| --- | --- | --- | --- | --- | --- | --- | --- | --- | --- | --- | --- | --- | --- | --- |
| Current life evaluation (1) |  | 0.38 | 0.43 | 0.27 | 0.29 | 0.32 | 0.20 | 0.20 | 0.17 | 0.21 | 0.20 | 0.12 | 0.11 | 0.24 |
| Life satisfaction (2) | 0.38 |  | 0.58 | 0.38 | 0.42 | 0.49 | 0.29 | 0.30 | 0.22 | 0.31 | 0.30 | 0.16 | 0.14 | 0.33 |
| Happiness (3) | **0.43** | **0.58** |  | 0.38 | 0.40 | 0.44 | 0.26 | 0.27 | 0.22 | 0.28 | 0.26 | 0.18 | 0.16 | 0.32 |
| Self-rated physical health (4) | **0.27** | **0.38** | 0.38 |  | 0.54 | 0.39 | 0.28 | 0.28 | 0.20 | 0.26 | 0.24 | 0.16 | 0.13 | 0.29 |
| Self-rated mental health (5) | **0.29** | **0.42** | 0.40 | 0.54 |  | 0.39 | 0.33 | 0.36 | 0.20 | 0.32 | 0.28 | 0.14 | 0.10 | 0.31 |
| Meaningful activities (6) | **0.32** | **0.49** | 0.44 | 0.39 | 0.39 |  | 0.31 | 0.31 | 0.22 | 0.29 | 0.28 | 0.18 | 0.14 | 0.31 |
| Understanding purpose (7) | **0.20** | **0.29** | 0.26 | 0.28 | 0.33 | 0.31 |  | 0.38 | 0.26 | 0.34 | 0.31 | 0.06 | 0.04 | 0.26 |
| Orientation to promote good (8) | **0.20** | **0.30** | 0.27 | 0.28 | 0.36 | 0.31 | 0.38 |  | 0.30 | 0.30 | 0.30 | 0.05 | 0.03 | 0.26 |
| Delayed gratification (9) | **0.17** | **0.22** | 0.22 | 0.20 | 0.20 | 0.22 | 0.26 | 0.30 |  | 0.22 | 0.20 | 0.07 | 0.04 | 0.19 |
| Relationship contentment (10) | **0.21** | **0.31** | 0.28 | 0.26 | 0.32 | 0.29 | 0.34 | 0.30 | 0.22 |  | 0.55 | 0.09 | 0.04 | 0.27 |
| Relationship satisfaction (11) | **0.20** | **0.30** | 0.26 | 0.24 | 0.28 | 0.28 | 0.31 | 0.30 | 0.20 | 0.55 |  | 0.04 | 0.01 | 0.25 |
| Financial security (12) | **0.12** | **0.16** | 0.18 | 0.16 | 0.14 | 0.18 | 0.06 | 0.05 | 0.07 | 0.09 | 0.04 |  | 0.59 | 0.15 |
| Material security (13) | **0.11** | **0.14** | 0.16 | 0.13 | 0.10 | 0.14 | 0.04 | 0.03 | 0.04 | 0.04 | 0.01 | 0.59 |  | 0.13 |

Table S10. Survey weighted correlations of LE and LS with flourishing indicators in Indonesia (N=6798)

| Variable | (1) | (2) | (3) | (4) | (5) | (6) | (7) | (8) | (9) | (10) | (11) | (12) | (13) | Avg. Cor. |
| --- | --- | --- | --- | --- | --- | --- | --- | --- | --- | --- | --- | --- | --- | --- |
| Current life evaluation (1) |  | 0.42 | 0.44 | 0.30 | 0.27 | 0.34 | 0.21 | 0.23 | 0.22 | 0.18 | 0.22 | 0.23 | 0.19 | 0.27 |
| Life satisfaction (2) | 0.42 |  | 0.57 | 0.46 | 0.44 | 0.55 | 0.36 | 0.36 | 0.35 | 0.34 | 0.40 | 0.26 | 0.19 | 0.39 |
| Happiness (3) | **0.44** | **0.57** |  | 0.45 | 0.47 | 0.50 | 0.32 | 0.35 | 0.34 | 0.33 | 0.37 | 0.25 | 0.20 | 0.38 |
| Self-rated physical health (4) | **0.30** | **0.46** | 0.45 |  | 0.55 | 0.43 | 0.37 | 0.41 | 0.36 | 0.36 | 0.38 | 0.20 | 0.16 | 0.37 |
| Self-rated mental health (5) | **0.27** | **0.44** | 0.47 | 0.55 |  | 0.47 | 0.40 | 0.43 | 0.39 | 0.41 | 0.43 | 0.19 | 0.18 | 0.39 |
| Meaningful activities (6) | **0.34** | **0.55** | 0.50 | 0.43 | 0.47 |  | 0.43 | 0.44 | 0.39 | 0.37 | 0.40 | 0.17 | 0.12 | 0.38 |
| Understanding purpose (7) | **0.21** | **0.36** | 0.32 | 0.37 | 0.40 | 0.43 |  | 0.44 | 0.39 | 0.49 | 0.49 | 0.13 | 0.10 | 0.34 |
| Orientation to promote good (8) | **0.23** | **0.36** | 0.35 | 0.41 | 0.43 | 0.44 | 0.44 |  | 0.52 | 0.43 | 0.41 | 0.14 | 0.12 | 0.36 |
| Delayed gratification (9) | **0.22** | **0.35** | 0.34 | 0.36 | 0.39 | 0.39 | 0.39 | 0.52 |  | 0.35 | 0.35 | 0.14 | 0.11 | 0.33 |
| Relationship contentment (10) | **0.18** | **0.34** | 0.33 | 0.36 | 0.41 | 0.37 | 0.49 | 0.43 | 0.35 |  | 0.60 | 0.12 | 0.10 | 0.34 |
| Relationship satisfaction (11) | **0.22** | **0.40** | 0.37 | 0.38 | 0.43 | 0.40 | 0.49 | 0.41 | 0.35 | 0.60 |  | 0.17 | 0.15 | 0.36 |
| Financial security (12) | **0.23** | **0.26** | 0.25 | 0.20 | 0.19 | 0.17 | 0.13 | 0.14 | 0.14 | 0.12 | 0.17 |  | 0.63 | 0.22 |
| Material security (13) | **0.19** | **0.19** | 0.20 | 0.16 | 0.18 | 0.12 | 0.10 | 0.12 | 0.11 | 0.10 | 0.15 | 0.63 |  | 0.19 |

Table S11. Survey weighted correlations of LE and LS with flourishing indicators in Israel (N=3573)

| Variable | (1) | (2) | (3) | (4) | (5) | (6) | (7) | (8) | (9) | (10) | (11) | (12) | (13) | Avg. Cor. |
| --- | --- | --- | --- | --- | --- | --- | --- | --- | --- | --- | --- | --- | --- | --- |
| Current life evaluation (1) |  | 0.70 | 0.61 | 0.40 | 0.49 | 0.47 | 0.35 | 0.30 | 0.17 | 0.41 | 0.40 | 0.37 | 0.38 | 0.42 |
| Life satisfaction (2) | 0.70 |  | 0.64 | 0.39 | 0.48 | 0.52 | 0.40 | 0.33 | 0.25 | 0.44 | 0.42 | 0.37 | 0.40 | 0.45 |
| Happiness (3) | **0.61** | **0.64** |  | 0.45 | 0.58 | 0.65 | 0.51 | 0.41 | 0.27 | 0.54 | 0.55 | 0.48 | 0.46 | 0.51 |
| Self-rated physical health (4) | **0.40** | **0.39** | 0.45 |  | 0.60 | 0.43 | 0.41 | 0.35 | 0.19 | 0.37 | 0.35 | 0.33 | 0.30 | 0.38 |
| Self-rated mental health (5) | **0.49** | **0.48** | 0.58 | 0.60 |  | 0.51 | 0.49 | 0.43 | 0.22 | 0.45 | 0.44 | 0.44 | 0.39 | 0.46 |
| Meaningful activities (6) | **0.47** | **0.52** | 0.65 | 0.43 | 0.51 |  | 0.62 | 0.46 | 0.33 | 0.56 | 0.54 | 0.46 | 0.44 | 0.50 |
| Understanding purpose (7) | **0.35** | **0.40** | 0.51 | 0.41 | 0.49 | 0.62 |  | 0.47 | 0.32 | 0.52 | 0.49 | 0.38 | 0.38 | 0.45 |
| Orientation to promote good (8) | **0.30** | **0.33** | 0.41 | 0.35 | 0.43 | 0.46 | 0.47 |  | 0.38 | 0.39 | 0.38 | 0.30 | 0.28 | 0.37 |
| Delayed gratification (9) | **0.17** | **0.25** | 0.27 | 0.19 | 0.22 | 0.33 | 0.32 | 0.38 |  | 0.26 | 0.30 | 0.17 | 0.16 | 0.25 |
| Relationship contentment (10) | **0.41** | **0.44** | 0.54 | 0.37 | 0.45 | 0.56 | 0.52 | 0.39 | 0.26 |  | 0.85 | 0.32 | 0.35 | 0.46 |
| Relationship satisfaction (11) | **0.40** | **0.42** | 0.55 | 0.35 | 0.44 | 0.54 | 0.49 | 0.38 | 0.30 | 0.85 |  | 0.36 | 0.36 | 0.45 |
| Financial security (12) | **0.37** | **0.37** | 0.48 | 0.33 | 0.44 | 0.46 | 0.38 | 0.30 | 0.17 | 0.32 | 0.36 |  | 0.77 | 0.40 |
| Material security (13) | **0.38** | **0.40** | 0.46 | 0.30 | 0.39 | 0.44 | 0.38 | 0.28 | 0.16 | 0.35 | 0.36 | 0.77 |  | 0.39 |

Table S12. Survey weighted correlations of LE and LS with flourishing indicators in Japan (N=19566)

| Variable | (1) | (2) | (3) | (4) | (5) | (6) | (7) | (8) | (9) | (10) | (11) | (12) | (13) | Avg. Cor. |
| --- | --- | --- | --- | --- | --- | --- | --- | --- | --- | --- | --- | --- | --- | --- |
| Current life evaluation (1) |  | 0.84 | 0.82 | 0.57 | 0.70 | 0.72 | 0.60 | 0.62 | 0.37 | 0.64 | 0.65 | 0.55 | 0.56 | 0.64 |
| Life satisfaction (2) | 0.84 |  | 0.88 | 0.60 | 0.76 | 0.78 | 0.62 | 0.64 | 0.37 | 0.68 | 0.70 | 0.56 | 0.58 | 0.67 |
| Happiness (3) | **0.82** | **0.88** |  | 0.60 | 0.75 | 0.77 | 0.62 | 0.66 | 0.36 | 0.68 | 0.70 | 0.50 | 0.54 | 0.66 |
| Self-rated physical health (4) | **0.57** | **0.60** | 0.60 |  | 0.73 | 0.58 | 0.50 | 0.56 | 0.36 | 0.53 | 0.54 | 0.39 | 0.43 | 0.53 |
| Self-rated mental health (5) | **0.70** | **0.76** | 0.75 | 0.73 |  | 0.71 | 0.63 | 0.69 | 0.39 | 0.68 | 0.69 | 0.48 | 0.52 | 0.64 |
| Meaningful activities (6) | **0.72** | **0.78** | 0.77 | 0.58 | 0.71 |  | 0.71 | 0.70 | 0.41 | 0.68 | 0.69 | 0.46 | 0.50 | 0.64 |
| Understanding purpose (7) | **0.60** | **0.62** | 0.62 | 0.50 | 0.63 | 0.71 |  | 0.70 | 0.48 | 0.66 | 0.66 | 0.42 | 0.44 | 0.59 |
| Orientation to promote good (8) | **0.62** | **0.64** | 0.66 | 0.56 | 0.69 | 0.70 | 0.70 |  | 0.48 | 0.65 | 0.65 | 0.42 | 0.45 | 0.60 |
| Delayed gratification (9) | **0.37** | **0.37** | 0.36 | 0.36 | 0.39 | 0.41 | 0.48 | 0.48 |  | 0.39 | 0.39 | 0.28 | 0.27 | 0.38 |
| Relationship contentment (10) | **0.64** | **0.68** | 0.68 | 0.53 | 0.68 | 0.68 | 0.66 | 0.65 | 0.39 |  | 0.90 | 0.43 | 0.49 | 0.62 |
| Relationship satisfaction (11) | **0.65** | **0.70** | 0.70 | 0.54 | 0.69 | 0.69 | 0.66 | 0.65 | 0.39 | 0.90 |  | 0.45 | 0.50 | 0.63 |
| Financial security (12) | **0.55** | **0.56** | 0.50 | 0.39 | 0.48 | 0.46 | 0.42 | 0.42 | 0.28 | 0.43 | 0.45 |  | 0.80 | 0.48 |
| Material security (13) | **0.56** | **0.58** | 0.54 | 0.43 | 0.52 | 0.50 | 0.44 | 0.45 | 0.27 | 0.49 | 0.50 | 0.80 |  | 0.51 |

Table S13. Survey weighted correlations of LE and LS with flourishing indicators in Kenya (N=11210)

| Variable | (1) | (2) | (3) | (4) | (5) | (6) | (7) | (8) | (9) | (10) | (11) | (12) | (13) | Avg. Cor. |
| --- | --- | --- | --- | --- | --- | --- | --- | --- | --- | --- | --- | --- | --- | --- |
| Current life evaluation (1) |  | 0.32 | 0.33 | 0.20 | 0.13 | 0.24 | 0.10 | 0.09 | 0.13 | 0.13 | 0.15 | 0.25 | 0.24 | 0.19 |
| Life satisfaction (2) | 0.32 |  | 0.36 | 0.23 | 0.16 | 0.35 | 0.15 | 0.16 | 0.14 | 0.18 | 0.24 | 0.29 | 0.27 | 0.24 |
| Happiness (3) | **0.33** | **0.36** |  | 0.32 | 0.25 | 0.33 | 0.21 | 0.20 | 0.18 | 0.23 | 0.26 | 0.22 | 0.21 | 0.26 |
| Self-rated physical health (4) | **0.20** | **0.23** | 0.32 |  | 0.39 | 0.32 | 0.25 | 0.25 | 0.22 | 0.23 | 0.22 | 0.17 | 0.16 | 0.25 |
| Self-rated mental health (5) | **0.13** | **0.16** | 0.25 | 0.39 |  | 0.27 | 0.29 | 0.24 | 0.24 | 0.28 | 0.24 | 0.08 | 0.09 | 0.22 |
| Meaningful activities (6) | **0.24** | **0.35** | 0.33 | 0.32 | 0.27 |  | 0.22 | 0.23 | 0.21 | 0.23 | 0.25 | 0.18 | 0.19 | 0.25 |
| Understanding purpose (7) | **0.10** | **0.15** | 0.21 | 0.25 | 0.29 | 0.22 |  | 0.31 | 0.28 | 0.32 | 0.28 | 0.10 | 0.09 | 0.22 |
| Orientation to promote good (8) | **0.09** | **0.16** | 0.20 | 0.25 | 0.24 | 0.23 | 0.31 |  | 0.35 | 0.26 | 0.24 | 0.08 | 0.09 | 0.21 |
| Delayed gratification (9) | **0.13** | **0.14** | 0.18 | 0.22 | 0.24 | 0.21 | 0.28 | 0.35 |  | 0.25 | 0.22 | 0.08 | 0.08 | 0.20 |
| Relationship contentment (10) | **0.13** | **0.18** | 0.23 | 0.23 | 0.28 | 0.23 | 0.32 | 0.26 | 0.25 |  | 0.45 | 0.12 | 0.12 | 0.23 |
| Relationship satisfaction (11) | **0.15** | **0.24** | 0.26 | 0.22 | 0.24 | 0.25 | 0.28 | 0.24 | 0.22 | 0.45 |  | 0.19 | 0.17 | 0.24 |
| Financial security (12) | **0.25** | **0.29** | 0.22 | 0.17 | 0.08 | 0.18 | 0.10 | 0.08 | 0.08 | 0.12 | 0.19 |  | 0.56 | 0.19 |
| Material security (13) | **0.24** | **0.27** | 0.21 | 0.16 | 0.09 | 0.19 | 0.09 | 0.09 | 0.08 | 0.12 | 0.17 | 0.56 |  | 0.19 |

Table S14. Survey weighted correlations of LE and LS with flourishing indicators in Mexico (N=5576)

| Variable | (1) | (2) | (3) | (4) | (5) | (6) | (7) | (8) | (9) | (10) | (11) | (12) | (13) | Avg. Cor. |
| --- | --- | --- | --- | --- | --- | --- | --- | --- | --- | --- | --- | --- | --- | --- |
| Current life evaluation (1) |  | 0.52 | 0.54 | 0.39 | 0.36 | 0.43 | 0.32 | 0.29 | 0.19 | 0.33 | 0.35 | 0.26 | 0.24 | 0.35 |
| Life satisfaction (2) | 0.52 |  | 0.72 | 0.47 | 0.53 | 0.66 | 0.45 | 0.33 | 0.22 | 0.47 | 0.50 | 0.21 | 0.18 | 0.44 |
| Happiness (3) | **0.54** | **0.72** |  | 0.46 | 0.52 | 0.60 | 0.43 | 0.34 | 0.22 | 0.42 | 0.46 | 0.21 | 0.18 | 0.43 |
| Self-rated physical health (4) | **0.39** | **0.47** | 0.46 |  | 0.54 | 0.42 | 0.36 | 0.35 | 0.23 | 0.32 | 0.36 | 0.17 | 0.14 | 0.35 |
| Self-rated mental health (5) | **0.36** | **0.53** | 0.52 | 0.54 |  | 0.53 | 0.50 | 0.42 | 0.26 | 0.44 | 0.45 | 0.16 | 0.13 | 0.40 |
| Meaningful activities (6) | **0.43** | **0.66** | 0.60 | 0.42 | 0.53 |  | 0.52 | 0.37 | 0.25 | 0.47 | 0.45 | 0.17 | 0.13 | 0.42 |
| Understanding purpose (7) | **0.32** | **0.45** | 0.43 | 0.36 | 0.50 | 0.52 |  | 0.43 | 0.31 | 0.47 | 0.45 | 0.14 | 0.10 | 0.37 |
| Orientation to promote good (8) | **0.29** | **0.33** | 0.34 | 0.35 | 0.42 | 0.37 | 0.43 |  | 0.41 | 0.37 | 0.36 | 0.05 | 0.02 | 0.31 |
| Delayed gratification (9) | **0.19** | **0.22** | 0.22 | 0.23 | 0.26 | 0.25 | 0.31 | 0.41 |  | 0.28 | 0.26 | 0.07 | 0.05 | 0.23 |
| Relationship contentment (10) | **0.33** | **0.47** | 0.42 | 0.32 | 0.44 | 0.47 | 0.47 | 0.37 | 0.28 |  | 0.67 | 0.17 | 0.13 | 0.38 |
| Relationship satisfaction (11) | **0.35** | **0.50** | 0.46 | 0.36 | 0.45 | 0.45 | 0.45 | 0.36 | 0.26 | 0.67 |  | 0.21 | 0.17 | 0.39 |
| Financial security (12) | **0.26** | **0.21** | 0.21 | 0.17 | 0.16 | 0.17 | 0.14 | 0.05 | 0.07 | 0.17 | 0.21 |  | 0.76 | 0.21 |
| Material security (13) | **0.24** | **0.18** | 0.18 | 0.14 | 0.13 | 0.13 | 0.10 | 0.02 | 0.05 | 0.13 | 0.17 | 0.76 |  | 0.19 |

Table S15. Survey weighted correlations of LE and LS with flourishing indicators in Nigeria (N=6633)

| Variable | (1) | (2) | (3) | (4) | (5) | (6) | (7) | (8) | (9) | (10) | (11) | (12) | (13) | Avg. Cor. |
| --- | --- | --- | --- | --- | --- | --- | --- | --- | --- | --- | --- | --- | --- | --- |
| Current life evaluation (1) |  | 0.36 | 0.35 | 0.20 | 0.14 | 0.24 | 0.18 | 0.16 | 0.15 | 0.20 | 0.21 | 0.18 | 0.18 | 0.21 |
| Life satisfaction (2) | 0.36 |  | 0.50 | 0.26 | 0.23 | 0.42 | 0.20 | 0.21 | 0.22 | 0.27 | 0.31 | 0.30 | 0.26 | 0.29 |
| Happiness (3) | **0.35** | **0.50** |  | 0.31 | 0.25 | 0.34 | 0.26 | 0.24 | 0.26 | 0.27 | 0.29 | 0.22 | 0.19 | 0.29 |
| Self-rated physical health (4) | **0.20** | **0.26** | 0.31 |  | 0.50 | 0.38 | 0.34 | 0.36 | 0.37 | 0.26 | 0.26 | 0.08 | 0.08 | 0.28 |
| Self-rated mental health (5) | **0.14** | **0.23** | 0.25 | 0.50 |  | 0.33 | 0.38 | 0.33 | 0.38 | 0.27 | 0.25 | 0.03 | 0.05 | 0.26 |
| Meaningful activities (6) | **0.24** | **0.42** | 0.34 | 0.38 | 0.33 |  | 0.34 | 0.28 | 0.27 | 0.28 | 0.26 | 0.18 | 0.17 | 0.29 |
| Understanding purpose (7) | **0.18** | **0.20** | 0.26 | 0.34 | 0.38 | 0.34 |  | 0.39 | 0.41 | 0.39 | 0.33 | 0.05 | 0.04 | 0.28 |
| Orientation to promote good (8) | **0.16** | **0.21** | 0.24 | 0.36 | 0.33 | 0.28 | 0.39 |  | 0.52 | 0.32 | 0.33 | 0.04 | -0.01 | 0.26 |
| Delayed gratification (9) | **0.15** | **0.22** | 0.26 | 0.37 | 0.38 | 0.27 | 0.41 | 0.52 |  | 0.32 | 0.32 | 0.01 | 0.02 | 0.27 |
| Relationship contentment (10) | **0.20** | **0.27** | 0.27 | 0.26 | 0.27 | 0.28 | 0.39 | 0.32 | 0.32 |  | 0.59 | 0.14 | 0.09 | 0.28 |
| Relationship satisfaction (11) | **0.21** | **0.31** | 0.29 | 0.26 | 0.25 | 0.26 | 0.33 | 0.33 | 0.32 | 0.59 |  | 0.16 | 0.11 | 0.28 |
| Financial security (12) | **0.18** | **0.30** | 0.22 | 0.08 | 0.03 | 0.18 | 0.05 | 0.04 | 0.01 | 0.14 | 0.16 |  | 0.62 | 0.17 |
| Material security (13) | **0.18** | **0.26** | 0.19 | 0.08 | 0.05 | 0.17 | 0.04 | -0.01 | 0.02 | 0.09 | 0.11 | 0.62 |  | 0.15 |

Table S16. Survey weighted correlations of LE and LS with flourishing indicators in the Philippines (N=5246)

| Variable | (1) | (2) | (3) | (4) | (5) | (6) | (7) | (8) | (9) | (10) | (11) | (12) | (13) | Avg. Cor. |
| --- | --- | --- | --- | --- | --- | --- | --- | --- | --- | --- | --- | --- | --- | --- |
| Current life evaluation (1) |  | 0.31 | 0.36 | 0.29 | 0.26 | 0.23 | 0.17 | 0.21 | 0.20 | 0.20 | 0.20 | 0.19 | 0.18 | 0.23 |
| Life satisfaction (2) | 0.31 |  | 0.58 | 0.41 | 0.44 | 0.42 | 0.31 | 0.32 | 0.33 | 0.37 | 0.38 | 0.24 | 0.24 | 0.36 |
| Happiness (3) | **0.36** | **0.58** |  | 0.42 | 0.42 | 0.37 | 0.30 | 0.29 | 0.31 | 0.35 | 0.38 | 0.23 | 0.24 | 0.35 |
| Self-rated physical health (4) | **0.29** | **0.41** | 0.42 |  | 0.54 | 0.35 | 0.35 | 0.34 | 0.32 | 0.34 | 0.34 | 0.18 | 0.18 | 0.34 |
| Self-rated mental health (5) | **0.26** | **0.44** | 0.42 | 0.54 |  | 0.41 | 0.41 | 0.39 | 0.39 | 0.37 | 0.38 | 0.17 | 0.18 | 0.36 |
| Meaningful activities (6) | **0.23** | **0.42** | 0.37 | 0.35 | 0.41 |  | 0.41 | 0.41 | 0.38 | 0.36 | 0.36 | 0.15 | 0.16 | 0.33 |
| Understanding purpose (7) | **0.17** | **0.31** | 0.30 | 0.35 | 0.41 | 0.41 |  | 0.44 | 0.45 | 0.49 | 0.46 | 0.12 | 0.15 | 0.34 |
| Orientation to promote good (8) | **0.21** | **0.32** | 0.29 | 0.34 | 0.39 | 0.41 | 0.44 |  | 0.58 | 0.39 | 0.41 | 0.11 | 0.11 | 0.33 |
| Delayed gratification (9) | **0.20** | **0.33** | 0.31 | 0.32 | 0.39 | 0.38 | 0.45 | 0.58 |  | 0.40 | 0.43 | 0.11 | 0.14 | 0.34 |
| Relationship contentment (10) | **0.20** | **0.37** | 0.35 | 0.34 | 0.37 | 0.36 | 0.49 | 0.39 | 0.40 |  | 0.63 | 0.13 | 0.14 | 0.35 |
| Relationship satisfaction (11) | **0.20** | **0.38** | 0.38 | 0.34 | 0.38 | 0.36 | 0.46 | 0.41 | 0.43 | 0.63 |  | 0.15 | 0.15 | 0.36 |
| Financial security (12) | **0.19** | **0.24** | 0.23 | 0.18 | 0.17 | 0.15 | 0.12 | 0.11 | 0.11 | 0.13 | 0.15 |  | 0.61 | 0.20 |
| Material security (13) | **0.18** | **0.24** | 0.24 | 0.18 | 0.18 | 0.16 | 0.15 | 0.11 | 0.14 | 0.14 | 0.15 | 0.61 |  | 0.21 |

Table S17. Survey weighted correlations of LE and LS with flourishing indicators in Poland (N=9855)

| Variable | (1) | (2) | (3) | (4) | (5) | (6) | (7) | (8) | (9) | (10) | (11) | (12) | (13) | Avg. Cor. |
| --- | --- | --- | --- | --- | --- | --- | --- | --- | --- | --- | --- | --- | --- | --- |
| Current life evaluation (1) |  | 0.53 | 0.57 | 0.35 | 0.34 | 0.47 | 0.32 | 0.34 | 0.20 | 0.38 | 0.39 | 0.33 | 0.30 | 0.38 |
| Life satisfaction (2) | 0.53 |  | 0.75 | 0.52 | 0.61 | 0.64 | 0.46 | 0.43 | 0.19 | 0.55 | 0.56 | 0.40 | 0.41 | 0.50 |
| Happiness (3) | **0.57** | **0.75** |  | 0.56 | 0.59 | 0.64 | 0.45 | 0.45 | 0.19 | 0.56 | 0.56 | 0.41 | 0.41 | 0.51 |
| Self-rated physical health (4) | **0.35** | **0.52** | 0.56 |  | 0.63 | 0.44 | 0.30 | 0.37 | 0.09 | 0.40 | 0.39 | 0.37 | 0.37 | 0.40 |
| Self-rated mental health (5) | **0.34** | **0.61** | 0.59 | 0.63 |  | 0.53 | 0.41 | 0.43 | 0.09 | 0.50 | 0.50 | 0.36 | 0.40 | 0.45 |
| Meaningful activities (6) | **0.47** | **0.64** | 0.64 | 0.44 | 0.53 |  | 0.58 | 0.51 | 0.23 | 0.52 | 0.52 | 0.37 | 0.39 | 0.49 |
| Understanding purpose (7) | **0.32** | **0.46** | 0.45 | 0.30 | 0.41 | 0.58 |  | 0.46 | 0.32 | 0.53 | 0.51 | 0.30 | 0.33 | 0.41 |
| Orientation to promote good (8) | **0.34** | **0.43** | 0.45 | 0.37 | 0.43 | 0.51 | 0.46 |  | 0.30 | 0.45 | 0.43 | 0.25 | 0.29 | 0.39 |
| Delayed gratification (9) | **0.20** | **0.19** | 0.19 | 0.09 | 0.09 | 0.23 | 0.32 | 0.30 |  | 0.24 | 0.25 | 0.09 | 0.09 | 0.19 |
| Relationship contentment (10) | **0.38** | **0.55** | 0.56 | 0.40 | 0.50 | 0.52 | 0.53 | 0.45 | 0.24 |  | 0.79 | 0.35 | 0.39 | 0.47 |
| Relationship satisfaction (11) | **0.39** | **0.56** | 0.56 | 0.39 | 0.50 | 0.52 | 0.51 | 0.43 | 0.25 | 0.79 |  | 0.31 | 0.35 | 0.46 |
| Financial security (12) | **0.33** | **0.40** | 0.41 | 0.37 | 0.36 | 0.37 | 0.30 | 0.25 | 0.09 | 0.35 | 0.31 |  | 0.80 | 0.36 |
| Material security (13) | **0.30** | **0.41** | 0.41 | 0.37 | 0.40 | 0.39 | 0.33 | 0.29 | 0.09 | 0.39 | 0.35 | 0.80 |  | 0.38 |

Table S18. Survey weighted correlations of LE and LS with flourishing indicators in South Africa (N=2586)

| Variable | (1) | (2) | (3) | (4) | (5) | (6) | (7) | (8) | (9) | (10) | (11) | (12) | (13) | Avg. Cor. |
| --- | --- | --- | --- | --- | --- | --- | --- | --- | --- | --- | --- | --- | --- | --- |
| Current life evaluation (1) |  | 0.43 | 0.47 | 0.23 | 0.19 | 0.37 | 0.22 | 0.20 | 0.17 | 0.25 | 0.24 | 0.30 | 0.25 | 0.28 |
| Life satisfaction (2) | 0.43 |  | 0.51 | 0.25 | 0.23 | 0.48 | 0.24 | 0.28 | 0.24 | 0.29 | 0.38 | 0.31 | 0.30 | 0.33 |
| Happiness (3) | **0.47** | **0.51** |  | 0.28 | 0.26 | 0.39 | 0.26 | 0.27 | 0.28 | 0.31 | 0.36 | 0.29 | 0.30 | 0.33 |
| Self-rated physical health (4) | **0.23** | **0.25** | 0.28 |  | 0.48 | 0.32 | 0.27 | 0.28 | 0.28 | 0.18 | 0.21 | 0.12 | 0.09 | 0.25 |
| Self-rated mental health (5) | **0.19** | **0.23** | 0.26 | 0.48 |  | 0.25 | 0.31 | 0.30 | 0.27 | 0.25 | 0.23 | 0.08 | 0.07 | 0.24 |
| Meaningful activities (6) | **0.37** | **0.48** | 0.39 | 0.32 | 0.25 |  | 0.29 | 0.37 | 0.24 | 0.24 | 0.30 | 0.24 | 0.23 | 0.31 |
| Understanding purpose (7) | **0.22** | **0.24** | 0.26 | 0.27 | 0.31 | 0.29 |  | 0.38 | 0.37 | 0.37 | 0.28 | 0.08 | 0.09 | 0.26 |
| Orientation to promote good (8) | **0.20** | **0.28** | 0.27 | 0.28 | 0.30 | 0.37 | 0.38 |  | 0.42 | 0.25 | 0.22 | 0.11 | 0.10 | 0.27 |
| Delayed gratification (9) | **0.17** | **0.24** | 0.28 | 0.28 | 0.27 | 0.24 | 0.37 | 0.42 |  | 0.33 | 0.28 | 0.07 | 0.11 | 0.25 |
| Relationship contentment (10) | **0.25** | **0.29** | 0.31 | 0.18 | 0.25 | 0.24 | 0.37 | 0.25 | 0.33 |  | 0.57 | 0.14 | 0.15 | 0.28 |
| Relationship satisfaction (11) | **0.24** | **0.38** | 0.36 | 0.21 | 0.23 | 0.30 | 0.28 | 0.22 | 0.28 | 0.57 |  | 0.20 | 0.21 | 0.29 |
| Financial security (12) | **0.30** | **0.31** | 0.29 | 0.12 | 0.08 | 0.24 | 0.08 | 0.11 | 0.07 | 0.14 | 0.20 |  | 0.63 | 0.21 |
| Material security (13) | **0.25** | **0.30** | 0.30 | 0.09 | 0.07 | 0.23 | 0.09 | 0.10 | 0.11 | 0.15 | 0.21 | 0.63 |  | 0.21 |

Table S19. Survey weighted correlations of LE and LS with flourishing indicators in Spain (N=6152)

| Variable | (1) | (2) | (3) | (4) | (5) | (6) | (7) | (8) | (9) | (10) | (11) | (12) | (13) | Avg. Cor. |
| --- | --- | --- | --- | --- | --- | --- | --- | --- | --- | --- | --- | --- | --- | --- |
| Current life evaluation (1) |  | 0.72 | 0.76 | 0.39 | 0.42 | 0.63 | 0.40 | 0.22 | 0.19 | 0.37 | 0.40 | 0.26 | 0.23 | 0.42 |
| Life satisfaction (2) | 0.72 |  | 0.82 | 0.39 | 0.48 | 0.76 | 0.45 | 0.23 | 0.20 | 0.43 | 0.46 | 0.24 | 0.23 | 0.45 |
| Happiness (3) | **0.76** | **0.82** |  | 0.40 | 0.50 | 0.74 | 0.45 | 0.26 | 0.21 | 0.45 | 0.48 | 0.21 | 0.18 | 0.46 |
| Self-rated physical health (4) | **0.39** | **0.39** | 0.40 |  | 0.56 | 0.34 | 0.33 | 0.22 | 0.21 | 0.29 | 0.30 | 0.17 | 0.16 | 0.31 |
| Self-rated mental health (5) | **0.42** | **0.48** | 0.50 | 0.56 |  | 0.47 | 0.42 | 0.30 | 0.25 | 0.40 | 0.40 | 0.19 | 0.19 | 0.38 |
| Meaningful activities (6) | **0.63** | **0.76** | 0.74 | 0.34 | 0.47 |  | 0.49 | 0.28 | 0.24 | 0.43 | 0.43 | 0.16 | 0.16 | 0.43 |
| Understanding purpose (7) | **0.40** | **0.45** | 0.45 | 0.33 | 0.42 | 0.49 |  | 0.32 | 0.29 | 0.42 | 0.42 | 0.14 | 0.13 | 0.35 |
| Orientation to promote good (8) | **0.22** | **0.23** | 0.26 | 0.22 | 0.30 | 0.28 | 0.32 |  | 0.37 | 0.31 | 0.27 | 0.01 | 0.02 | 0.23 |
| Delayed gratification (9) | **0.19** | **0.20** | 0.21 | 0.21 | 0.25 | 0.24 | 0.29 | 0.37 |  | 0.23 | 0.22 | 0.06 | 0.04 | 0.21 |
| Relationship contentment (10) | **0.37** | **0.43** | 0.45 | 0.29 | 0.40 | 0.43 | 0.42 | 0.31 | 0.23 |  | 0.73 | 0.14 | 0.15 | 0.36 |
| Relationship satisfaction (11) | **0.40** | **0.46** | 0.48 | 0.30 | 0.40 | 0.43 | 0.42 | 0.27 | 0.22 | 0.73 |  | 0.17 | 0.15 | 0.37 |
| Financial security (12) | **0.26** | **0.24** | 0.21 | 0.17 | 0.19 | 0.16 | 0.14 | 0.01 | 0.06 | 0.14 | 0.17 |  | 0.72 | 0.21 |
| Material security (13) | **0.23** | **0.23** | 0.18 | 0.16 | 0.19 | 0.16 | 0.13 | 0.02 | 0.04 | 0.15 | 0.15 | 0.72 |  | 0.20 |

Table S20. Survey weighted correlations of LE and LS with flourishing indicators in Sweden (N=14523)

| Variable | (1) | (2) | (3) | (4) | (5) | (6) | (7) | (8) | (9) | (10) | (11) | (12) | (13) | Avg. Cor. |
| --- | --- | --- | --- | --- | --- | --- | --- | --- | --- | --- | --- | --- | --- | --- |
| Current life evaluation (1) |  | 0.79 | 0.75 | 0.47 | 0.63 | 0.67 | 0.52 | 0.37 | 0.21 | 0.54 | 0.55 | 0.41 | 0.39 | 0.52 |
| Life satisfaction (2) | 0.79 |  | 0.86 | 0.47 | 0.70 | 0.78 | 0.57 | 0.38 | 0.21 | 0.62 | 0.63 | 0.40 | 0.38 | 0.57 |
| Happiness (3) | **0.75** | **0.86** |  | 0.44 | 0.72 | 0.75 | 0.58 | 0.40 | 0.22 | 0.62 | 0.64 | 0.38 | 0.37 | 0.56 |
| Self-rated physical health (4) | **0.47** | **0.47** | 0.44 |  | 0.48 | 0.42 | 0.35 | 0.28 | 0.21 | 0.33 | 0.35 | 0.30 | 0.28 | 0.37 |
| Self-rated mental health (5) | **0.63** | **0.70** | 0.72 | 0.48 |  | 0.64 | 0.53 | 0.39 | 0.20 | 0.53 | 0.54 | 0.41 | 0.39 | 0.51 |
| Meaningful activities (6) | **0.67** | **0.78** | 0.75 | 0.42 | 0.64 |  | 0.65 | 0.43 | 0.24 | 0.57 | 0.57 | 0.34 | 0.33 | 0.53 |
| Understanding purpose (7) | **0.52** | **0.57** | 0.58 | 0.35 | 0.53 | 0.65 |  | 0.43 | 0.25 | 0.53 | 0.52 | 0.27 | 0.28 | 0.46 |
| Orientation to promote good (8) | **0.37** | **0.38** | 0.40 | 0.28 | 0.39 | 0.43 | 0.43 |  | 0.38 | 0.39 | 0.36 | 0.20 | 0.22 | 0.35 |
| Delayed gratification (9) | **0.21** | **0.21** | 0.22 | 0.21 | 0.20 | 0.24 | 0.25 | 0.38 |  | 0.20 | 0.19 | 0.10 | 0.11 | 0.21 |
| Relationship contentment (10) | **0.54** | **0.62** | 0.62 | 0.33 | 0.53 | 0.57 | 0.53 | 0.39 | 0.20 |  | 0.86 | 0.29 | 0.31 | 0.48 |
| Relationship satisfaction (11) | **0.55** | **0.63** | 0.64 | 0.35 | 0.54 | 0.57 | 0.52 | 0.36 | 0.19 | 0.86 |  | 0.30 | 0.32 | 0.49 |
| Financial security (12) | **0.41** | **0.40** | 0.38 | 0.30 | 0.41 | 0.34 | 0.27 | 0.20 | 0.10 | 0.29 | 0.30 |  | 0.73 | 0.34 |
| Material security (13) | **0.39** | **0.38** | 0.37 | 0.28 | 0.39 | 0.33 | 0.28 | 0.22 | 0.11 | 0.31 | 0.32 | 0.73 |  | 0.34 |

Table S21. Survey weighted correlations of LE and LS with flourishing indicators in Tanzania (N=8829)

| Variable | (1) | (2) | (3) | (4) | (5) | (6) | (7) | (8) | (9) | (10) | (11) | (12) | (13) | Avg. Cor. |
| --- | --- | --- | --- | --- | --- | --- | --- | --- | --- | --- | --- | --- | --- | --- |
| Current life evaluation (1) |  | 0.36 | 0.35 | 0.19 | 0.08 | 0.26 | 0.14 | 0.12 | 0.14 | 0.11 | 0.13 | 0.30 | 0.29 | 0.21 |
| Life satisfaction (2) | 0.36 |  | 0.44 | 0.25 | 0.15 | 0.41 | 0.17 | 0.15 | 0.15 | 0.22 | 0.26 | 0.33 | 0.32 | 0.27 |
| Happiness (3) | **0.35** | **0.44** |  | 0.37 | 0.25 | 0.43 | 0.25 | 0.24 | 0.20 | 0.25 | 0.26 | 0.32 | 0.33 | 0.31 |
| Self-rated physical health (4) | **0.19** | **0.25** | 0.37 |  | 0.42 | 0.38 | 0.30 | 0.30 | 0.25 | 0.24 | 0.24 | 0.26 | 0.26 | 0.29 |
| Self-rated mental health (5) | **0.08** | **0.15** | 0.25 | 0.42 |  | 0.31 | 0.30 | 0.31 | 0.23 | 0.26 | 0.22 | 0.16 | 0.17 | 0.24 |
| Meaningful activities (6) | **0.26** | **0.41** | 0.43 | 0.38 | 0.31 |  | 0.34 | 0.29 | 0.25 | 0.27 | 0.27 | 0.27 | 0.28 | 0.31 |
| Understanding purpose (7) | **0.14** | **0.17** | 0.25 | 0.30 | 0.30 | 0.34 |  | 0.34 | 0.28 | 0.33 | 0.29 | 0.17 | 0.21 | 0.26 |
| Orientation to promote good (8) | **0.12** | **0.15** | 0.24 | 0.30 | 0.31 | 0.29 | 0.34 |  | 0.36 | 0.29 | 0.24 | 0.15 | 0.18 | 0.25 |
| Delayed gratification (9) | **0.14** | **0.15** | 0.20 | 0.25 | 0.23 | 0.25 | 0.28 | 0.36 |  | 0.23 | 0.20 | 0.16 | 0.15 | 0.22 |
| Relationship contentment (10) | **0.11** | **0.22** | 0.25 | 0.24 | 0.26 | 0.27 | 0.33 | 0.29 | 0.23 |  | 0.52 | 0.24 | 0.19 | 0.26 |
| Relationship satisfaction (11) | **0.13** | **0.26** | 0.26 | 0.24 | 0.22 | 0.27 | 0.29 | 0.24 | 0.20 | 0.52 |  | 0.23 | 0.21 | 0.26 |
| Financial security (12) | **0.30** | **0.33** | 0.32 | 0.26 | 0.16 | 0.27 | 0.17 | 0.15 | 0.16 | 0.24 | 0.23 |  | 0.57 | 0.26 |
| Material security (13) | **0.29** | **0.32** | 0.33 | 0.26 | 0.17 | 0.28 | 0.21 | 0.18 | 0.15 | 0.19 | 0.21 | 0.57 |  | 0.26 |

Table S22. Survey weighted correlations of LE and LS with flourishing indicators in Turkey (N=1448)

| Variable | (1) | (2) | (3) | (4) | (5) | (6) | (7) | (8) | (9) | (10) | (11) | (12) | (13) | Avg. Cor. |
| --- | --- | --- | --- | --- | --- | --- | --- | --- | --- | --- | --- | --- | --- | --- |
| Current life evaluation (1) |  | 0.57 | 0.53 | 0.33 | 0.41 | 0.42 | 0.38 | 0.26 | 0.25 | 0.30 | 0.34 | 0.46 | 0.39 | 0.39 |
| Life satisfaction (2) | 0.57 |  | 0.76 | 0.35 | 0.54 | 0.58 | 0.42 | 0.29 | 0.24 | 0.32 | 0.39 | 0.53 | 0.49 | 0.46 |
| Happiness (3) | **0.53** | **0.76** |  | 0.37 | 0.53 | 0.60 | 0.42 | 0.31 | 0.22 | 0.32 | 0.39 | 0.45 | 0.43 | 0.44 |
| Self-rated physical health (4) | **0.33** | **0.35** | 0.37 |  | 0.52 | 0.39 | 0.35 | 0.32 | 0.27 | 0.25 | 0.23 | 0.34 | 0.28 | 0.33 |
| Self-rated mental health (5) | **0.41** | **0.54** | 0.53 | 0.52 |  | 0.49 | 0.46 | 0.29 | 0.25 | 0.38 | 0.39 | 0.38 | 0.33 | 0.41 |
| Meaningful activities (6) | **0.42** | **0.58** | 0.60 | 0.39 | 0.49 |  | 0.51 | 0.39 | 0.29 | 0.30 | 0.35 | 0.41 | 0.39 | 0.43 |
| Understanding purpose (7) | **0.38** | **0.42** | 0.42 | 0.35 | 0.46 | 0.51 |  | 0.42 | 0.39 | 0.40 | 0.43 | 0.33 | 0.34 | 0.40 |
| Orientation to promote good (8) | **0.26** | **0.29** | 0.31 | 0.32 | 0.29 | 0.39 | 0.42 |  | 0.46 | 0.32 | 0.27 | 0.25 | 0.25 | 0.32 |
| Delayed gratification (9) | **0.25** | **0.24** | 0.22 | 0.27 | 0.25 | 0.29 | 0.39 | 0.46 |  | 0.24 | 0.22 | 0.19 | 0.19 | 0.27 |
| Relationship contentment (10) | **0.30** | **0.32** | 0.32 | 0.25 | 0.38 | 0.30 | 0.40 | 0.32 | 0.24 |  | 0.66 | 0.26 | 0.23 | 0.33 |
| Relationship satisfaction (11) | **0.34** | **0.39** | 0.39 | 0.23 | 0.39 | 0.35 | 0.43 | 0.27 | 0.22 | 0.66 |  | 0.28 | 0.32 | 0.36 |
| Financial security (12) | **0.46** | **0.53** | 0.45 | 0.34 | 0.38 | 0.41 | 0.33 | 0.25 | 0.19 | 0.26 | 0.28 |  | 0.69 | 0.38 |
| Material security (13) | **0.39** | **0.49** | 0.43 | 0.28 | 0.33 | 0.39 | 0.34 | 0.25 | 0.19 | 0.23 | 0.32 | 0.69 |  | 0.36 |

Table S23. Survey weighted correlations of LE and LS with flourishing indicators in the United Kingdom (N=5204)

| Variable | (1) | (2) | (3) | (4) | (5) | (6) | (7) | (8) | (9) | (10) | (11) | (12) | (13) | Avg. Cor. |
| --- | --- | --- | --- | --- | --- | --- | --- | --- | --- | --- | --- | --- | --- | --- |
| Current life evaluation (1) |  | 0.73 | 0.71 | 0.43 | 0.53 | 0.64 | 0.49 | 0.34 | 0.33 | 0.49 | 0.48 | 0.40 | 0.38 | 0.50 |
| Life satisfaction (2) | 0.73 |  | 0.84 | 0.44 | 0.63 | 0.79 | 0.57 | 0.39 | 0.37 | 0.58 | 0.58 | 0.43 | 0.41 | 0.56 |
| Happiness (3) | **0.71** | **0.84** |  | 0.43 | 0.66 | 0.77 | 0.58 | 0.43 | 0.38 | 0.58 | 0.58 | 0.40 | 0.38 | 0.56 |
| Self-rated physical health (4) | **0.43** | **0.44** | 0.43 |  | 0.54 | 0.40 | 0.37 | 0.30 | 0.27 | 0.31 | 0.31 | 0.26 | 0.27 | 0.36 |
| Self-rated mental health (5) | **0.53** | **0.63** | 0.66 | 0.54 |  | 0.59 | 0.53 | 0.37 | 0.33 | 0.52 | 0.51 | 0.42 | 0.43 | 0.51 |
| Meaningful activities (6) | **0.64** | **0.79** | 0.77 | 0.40 | 0.59 |  | 0.63 | 0.44 | 0.38 | 0.57 | 0.54 | 0.36 | 0.34 | 0.54 |
| Understanding purpose (7) | **0.49** | **0.57** | 0.58 | 0.37 | 0.53 | 0.63 |  | 0.44 | 0.41 | 0.55 | 0.53 | 0.31 | 0.30 | 0.48 |
| Orientation to promote good (8) | **0.34** | **0.39** | 0.43 | 0.30 | 0.37 | 0.44 | 0.44 |  | 0.51 | 0.40 | 0.36 | 0.23 | 0.22 | 0.37 |
| Delayed gratification (9) | **0.33** | **0.37** | 0.38 | 0.27 | 0.33 | 0.38 | 0.41 | 0.51 |  | 0.36 | 0.34 | 0.17 | 0.17 | 0.34 |
| Relationship contentment (10) | **0.49** | **0.58** | 0.58 | 0.31 | 0.52 | 0.57 | 0.55 | 0.40 | 0.36 |  | 0.84 | 0.30 | 0.31 | 0.48 |
| Relationship satisfaction (11) | **0.48** | **0.58** | 0.58 | 0.31 | 0.51 | 0.54 | 0.53 | 0.36 | 0.34 | 0.84 |  | 0.31 | 0.31 | 0.47 |
| Financial security (12) | **0.40** | **0.43** | 0.40 | 0.26 | 0.42 | 0.36 | 0.31 | 0.23 | 0.17 | 0.30 | 0.31 |  | 0.77 | 0.36 |
| Material security (13) | **0.38** | **0.41** | 0.38 | 0.27 | 0.43 | 0.34 | 0.30 | 0.22 | 0.17 | 0.31 | 0.31 | 0.77 |  | 0.36 |

Table S24. Survey weighted correlations of LE and LS with flourishing indicators in the United States (N=37727)

| Variable | (1) | (2) | (3) | (4) | (5) | (6) | (7) | (8) | (9) | (10) | (11) | (12) | (13) | Avg. Cor. |
| --- | --- | --- | --- | --- | --- | --- | --- | --- | --- | --- | --- | --- | --- | --- |
| Current life evaluation (1) |  | 0.76 | 0.70 | 0.50 | 0.60 | 0.65 | 0.52 | 0.40 | 0.36 | 0.54 | 0.52 | 0.53 | 0.51 | 0.55 |
| Life satisfaction (2) | 0.76 |  | 0.84 | 0.50 | 0.70 | 0.77 | 0.59 | 0.41 | 0.37 | 0.63 | 0.62 | 0.49 | 0.49 | 0.60 |
| Happiness (3) | **0.70** | **0.84** |  | 0.48 | 0.75 | 0.76 | 0.61 | 0.45 | 0.39 | 0.64 | 0.63 | 0.45 | 0.44 | 0.60 |
| Self-rated physical health (4) | **0.50** | **0.50** | 0.48 |  | 0.53 | 0.46 | 0.38 | 0.35 | 0.32 | 0.38 | 0.38 | 0.34 | 0.34 | 0.41 |
| Self-rated mental health (5) | **0.60** | **0.70** | 0.75 | 0.53 |  | 0.67 | 0.61 | 0.47 | 0.41 | 0.60 | 0.56 | 0.43 | 0.41 | 0.56 |
| Meaningful activities (6) | **0.65** | **0.77** | 0.76 | 0.46 | 0.67 |  | 0.67 | 0.46 | 0.40 | 0.62 | 0.59 | 0.40 | 0.41 | 0.57 |
| Understanding purpose (7) | **0.52** | **0.59** | 0.61 | 0.38 | 0.61 | 0.67 |  | 0.48 | 0.42 | 0.61 | 0.58 | 0.33 | 0.32 | 0.51 |
| Orientation to promote good (8) | **0.40** | **0.41** | 0.45 | 0.35 | 0.47 | 0.46 | 0.48 |  | 0.52 | 0.42 | 0.39 | 0.23 | 0.24 | 0.40 |
| Delayed gratification (9) | **0.36** | **0.37** | 0.39 | 0.32 | 0.41 | 0.40 | 0.42 | 0.52 |  | 0.38 | 0.34 | 0.22 | 0.21 | 0.36 |
| Relationship contentment (10) | **0.54** | **0.63** | 0.64 | 0.38 | 0.60 | 0.62 | 0.61 | 0.42 | 0.38 |  | 0.86 | 0.32 | 0.34 | 0.53 |
| Relationship satisfaction (11) | **0.52** | **0.62** | 0.63 | 0.38 | 0.56 | 0.59 | 0.58 | 0.39 | 0.34 | 0.86 |  | 0.32 | 0.33 | 0.51 |
| Financial security (12) | **0.53** | **0.49** | 0.45 | 0.34 | 0.43 | 0.40 | 0.33 | 0.23 | 0.22 | 0.32 | 0.32 |  | 0.81 | 0.41 |
| Material security (13) | **0.51** | **0.49** | 0.44 | 0.34 | 0.41 | 0.41 | 0.32 | 0.24 | 0.21 | 0.34 | 0.33 | 0.81 |  | 0.40 |

Table S25. Summary of missing data across flourishing indicators.

| COUNTRY | Current life evaluation | Life satisfaction | Happiness | Self-rated physical health | Self-rated mental health | Meaningful activities | Understanding purpose | Orientation to promote good | Delayed gratification | Relationship contentment | Relationship satisfaction | Financial security | Material security |
| --- | --- | --- | --- | --- | --- | --- | --- | --- | --- | --- | --- | --- | --- |
| Argentina | 0.06 | 0.22 | 0.22 | 0.09 | 0.16 | 0.21 | 0.39 | 0.27 | 0.62 | 0.27 | 0.43 | 0.19 | 0.19 |
| Australia | 0.13 | 0.23 | 0.21 | 0.10 | 0.13 | 0.05 | 0.13 | 0.16 | 0.13 | 0.18 | 0.18 | 0.10 | 0.10 |
| Brazil | 0.08 | 0.24 | 0.33 | 0.22 | 0.14 | 0.14 | 0.30 | 0.39 | 0.23 | 0.33 | 0.42 | 0.20 | 0.23 |
| Egypt | 0.66 | 0.53 | 0.32 | 0.27 | 0.72 | 0.44 | 0.61 | 1.10 | 0.97 | 0.21 | 0.23 | 0.19 | 0.25 |
| Germany | 0.09 | 0.35 | 0.16 | 0.09 | 0.14 | 0.09 | 0.17 | 0.11 | 0.13 | 0.26 | 0.28 | 0.08 | 0.06 |
| Hong Kong | 0.07 | 1.69 | 0.13 | 0.00 | 0.53 | 0.93 | 0.07 | 0.03 | 0.50 | 1.00 | 1.46 | 0.07 | 1.16 |
| India | 1.06 | 0.43 | 0.27 | 0.22 | 0.24 | 1.07 | 0.87 | 0.57 | 0.74 | 0.27 | 0.52 | 0.26 | 0.15 |
| Indonesia | 0.57 | 0.30 | 0.40 | 0.27 | 0.36 | 0.40 | 0.31 | 0.43 | 0.60 | 0.33 | 0.44 | 0.26 | 0.11 |
| Israel | 0.11 | 0.25 | 0.08 | 0.05 | 0.08 | 0.16 | 0.46 | 0.30 | 0.52 | 0.08 | 0.46 | 0.35 | 0.19 |
| Japan | 0.35 | 0.60 | 0.39 | 0.29 | 0.17 | 0.42 | 0.53 | 0.50 | 0.35 | 0.66 | 0.92 | 0.35 | 0.39 |
| Kenya | 0.15 | 0.11 | 0.14 | 0.13 | 0.11 | 0.14 | 0.20 | 0.09 | 0.11 | 0.21 | 0.20 | 0.12 | 0.11 |
| Mexico | 0.24 | 0.24 | 0.36 | 0.24 | 0.28 | 0.31 | 0.33 | 0.52 | 0.74 | 0.43 | 0.42 | 0.26 | 0.33 |
| Nigeria | 0.25 | 0.25 | 0.15 | 0.16 | 0.26 | 0.40 | 0.29 | 0.35 | 0.56 | 0.35 | 0.25 | 0.22 | 0.18 |
| Philippines | 0.08 | 0.11 | 0.06 | 0.06 | 0.09 | 0.06 | 0.13 | 0.06 | 0.13 | 0.13 | 0.17 | 0.06 | 0.17 |
| Poland | 0.14 | 0.99 | 0.17 | 0.16 | 0.21 | 0.46 | 0.33 | 0.49 | 0.40 | 1.31 | 1.16 | 0.35 | 0.84 |
| South Africa | 0.26 | 0.38 | 0.04 | 0.11 | 0.23 | 0.34 | 0.15 | 0.08 | 0.38 | 0.23 | 0.34 | 0.38 | 0.23 |
| Spain | 0.08 | 0.22 | 0.21 | 0.32 | 0.22 | 0.19 | 0.40 | 0.24 | 0.13 | 0.25 | 0.27 | 0.11 | 0.14 |
| Sweden | 0.30 | 0.33 | 0.29 | 0.27 | 0.19 | 0.29 | 0.71 | 0.54 | 0.69 | 0.33 | 0.41 | 0.23 | 0.36 |
| Tanzania | 0.15 | 0.10 | 0.17 | 0.14 | 0.33 | 0.31 | 1.09 | 0.32 | 0.33 | 0.23 | 0.30 | 0.14 | 0.11 |
| Turkey | 0.14 | 0.20 | 0.07 | 0.07 | 0.00 | 0.20 | 0.27 | 0.00 | 0.07 | 0.07 | 0.34 | 0.14 | 0.14 |
| United Kingdom | 0.20 | 0.60 | 0.26 | 0.07 | 0.26 | 0.19 | 0.20 | 0.28 | 0.56 | 0.48 | 0.39 | 0.11 | 0.37 |
| United States | 0.06 | 0.14 | 0.09 | 0.10 | 0.09 | 0.08 | 0.21 | 0.16 | 0.33 | 0.17 | 0.22 | 0.06 | 0.11 |

| **`**  *Gender subgroup analysis--Average correlation of LS with all other flourishing indicators is greater than LE for all countries included in the GFS (Male N=92,484; Female N=103,748).* | | | | | |
| --- | --- | --- | --- | --- | --- |
|  | Male | |  | Female | |
| Country | LS | LE |  | LS | LE |
| Argentina | 0.43 (0.05) | 0.33 (0.05) |  | 0.44 (0.05) | 0.36 (0.05) |
| Australia | 0.58 (0.05) | 0.53 (0.05) |  | 0.59 (0.05) | 0.55 (0.05) |
| Brazil | 0.48 (0.04) | 0.40 (0.04) |  | 0.47 (0.04) | 0.39 (0.04) |
| Egypt | 0.27 (0.05) | 0.22 (0.05) |  | 0.26 (0.05) | 0.24 (0.05) |
| Germany | 0.45 (0.04) | 0.38 (0.04) |  | 0.44 (0.04) | 0.38 (0.04) |
| Hong Kong | 0.72 (0.04) | 0.67 (0.05) |  | 0.72 (0.04) | 0.67 (0.05) |
| India | 0.32 (0.04) | 0.23 (0.04) |  | 0.35 (0.04) | 0.25 (0.04) |
| Indonesia | 0.37 (0.05) | 0.26 (0.05) |  | 0.41 (0.05) | 0.28 (0.05) |
| Israel | 0.46 (0.06) | 0.42 (0.06) |  | 0.43 (0.06) | 0.42 (0.05) |
| Japan | 0.67 (0.03) | 0.63 (0.03) |  | 0.67 (0.03) | 0.64 (0.03) |
| Kenya | 0.24 (0.04) | 0.20 (0.04) |  | 0.23 (0.04) | 0.19 (0.04) |
| Mexico | 0.44 (0.05) | 0.36 (0.05) |  | 0.44 (0.04) | 0.35 (0.04) |
| Nigeria | 0.30 (0.05) | 0.22 (0.05) |  | 0.29 (0.05) | 0.21 (0.05) |
| Philippines | 0.36 (0.05) | 0.24 (0.05) |  | 0.37 (0.04) | 0.22 (0.04) |
| Poland | 0.52 (0.05) | 0.39 (0.05) |  | 0.49 (0.05) | 0.36 (0.05) |
| South Africa | 0.31 (0.06) | 0.25 (0.06) |  | 0.34 (0.06) | 0.30 (0.06) |
| Spain | 0.45 (0.04) | 0.42 (0.04) |  | 0.45 (0.04) | 0.41 (0.04) |
| Sweden | 0.57 (0.03) | 0.52 (0.03) |  | 0.57 (0.03) | 0.53 (0.03) |
| Tanzania | 0.25 (0.04) | 0.20 (0.04) |  | 0.28 (0.04) | 0.21 (0.04) |
| Turkey | 0.44 (0.06) | 0.35 (0.06) |  | 0.48 (0.06) | 0.43 (0.06) |
| United Kingdom | 0.58 (0.04) | 0.52 (0.04) |  | 0.55 (0.04) | 0.47 (0.05) |
| United States | 0.58 (0.04) | 0.53 (0.04) |  | 0.61 (0.04) | 0.57 (0.04) |
